# Synthesis, anticancer evaluation and molecular docking studies of new benzimidazole- 1,3,4-oxadiazole derivatives as human topoisomerase types I poison

**DOI:** 10.1080/14756366.2020.1806831

**Published:** 2020-08-19

**Authors:** Ulviye Acar Çevik, Begüm Nurpelin Sağlık, Derya Osmaniye, Serkan Levent, Betül Kaya Çavuşoğlu, Abdullah Burak Karaduman, Özlem Atlı Eklioğlu, Yusuf Özkay, Zafer Asım Kaplancıklı

**Affiliations:** a Department of Pharmaceutical Chemistry, Faculty of Pharmacy, Anadolu University, Eskişehir, Turkey; b Doping and Narcotic Compounds Analysis Laboratory, Faculty of Pharmacy, Anadolu University, Eskişehir, Turkey; c Department of Pharmaceutical Chemistry, Faculty of Pharmacy, Zonguldak Bülent Ecevit University, Zonguldak, Turkey; d Department of Pharmaceutical Toxicology, Faculty of Pharmacy, Anadolu University, Eskişehir, Turkey

**Keywords:** Benzimidazole, 1,3,4-oxadiazole, anticancer, DNA Topo I, Hoechst 33342

## Abstract

In this study, some benzimidazole-oxadiazole derivatives were synthesised and tested for their *in vitro* anticancer activities on five cancer cell lines, including HeLa, MCF7, A549, HepG2 and C6. Their structures were elucidated by IR, ^1^H-NMR, ^13^C-NMR, 2 D-NMR and HRMS spectroscopic methods. Among all screened compounds; **5a**, **5b**, **5d**, **5e**, **5k**, **5l**, **5n** and **5o** exhibited potent selective cytotoxic activities against various tested cancer cell lines. Especially, compounds **5l** and **5n** exhibited the most antiproliferative activity than Hoechst 33342 and doxorubicin against HeLa cell line, with IC_50_ of 0.224 ± 0.011 µM and 0.205 ± 0.010 µM, respectively. Furthermore, these potent lead cytotoxic agents were evaluated in terms of their inhibition potency against Topoisomerase I and it was determined that selected compounds inhibited the Topoisomerase I. Docking studies were performed and probable interactions in the DNA-Topo I enzyme complex was determined.

## 1. Introduction

DNA topoisomerases (Topo) are ubiquitous enzymes that are required for proper chromosome structure and segregation. These enzymes play important roles in triggering, controlling, and modifying topological DNA problems during cell proliferation, differentiation, and survival. Thus, topo enzymes possesses immense importance in almost all stages of the cell cycle. As a result, the role of mammalian DNA topoisomerases as molecular targets for anticancer drugs has been explored, and it was found that topoisomerase inhibition could curb cancerous cell growth across a variety of cell lines^1–8^.

Human Topo are divided into two classes: Topoisomerase I (topo I), breaks only one strand of DNA and Topoisomerase II (topo II), breaks both strands of DNA^9–11^. Several Topo poisons have been approved for the chemotherapeutic treatment of different cancer types in recent decades, including the Topo I inhibitors camptothecin, topotecan and the Topo-II-targeting drugs etoposide and amsacrin^12^^,^^13^.

In the field of anticancer drugs, DNA topoisomerase I has been reported to be an important target for anticancer agents but topoisomerase inhibitors have several barriers and limitations. These include poor solubility, accumulation in target tissues or organs and prolonged half-life in plasma as well as drug efflux from the target cells and cancer cell resistance^14–17^.

**Figure 1.**
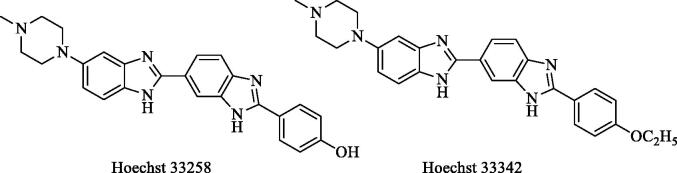
Hoechst 33258 and Hoechst 33342 compounds.

Benzimidazoles represent a structurally unique class of Topo I poisons that act as DNA minor groove binders such as Hoescht 33258 and Hoechst 33342 (Figure 1)^18–20^. These drugs are known to hinder the breakage/reunion reaction of topoisomerase I, in which the enzyme is reversibly trapped in a state where the DNA is cleave^6^. Furthermore, benzimidazole is known to be a versatile scaffold that possess potential anticancer, antitumor and antiproliferative activities^21–27^. Similarly, oxadiazole has engrossed significant attention of medicinal chemists owing to their wide range of useful pharmacological actions particularly cytotoxic activities against DNA topoisomerase I^28–31^.

In the light of this information, it is aimed to develop new anticancer effective compounds in this research. To this aim, Hoechst compounds have been identified as precursor compounds. In addition to the hydroxy (–OH) and ethoxy (–OC_2_H_5_) substituents in the para position of the phenyl ring in the Hoechst compounds, the methoxy (–OCH_3_) substituent was also used for synthesised compounds. Instead of bisbenzimidazole, the 1,3,4-oxadiazole ring attached to the benzimidazole ring was synthesised. Methyl, ethyl, pyridine, pyrimidine and phenyl substituents were used for the fourth position of piperazine. The cytotoxicity of these synthesised compounds to different cell lines, and Topo I inhibitory activities were evaluated. Besides different mechanisms such as Flow cytometric analysis, DNA synthesis inhibition assay was also studied in our research. In order to determine the possible interactions of compound, showed high activity, docking studies have been performed.

## 2. Experimental

### 2.1. Chemistry

Whole chemicals employed in the synthetic procedure were purchased from Sigma-Aldrich Chemicals (Sigma-Aldrich Corp., St. Louis, MO, USA) or Merck Chemicals (Merck KGaA, Darmstadt, Germany). Melting points of the obtained compounds were determined by MP90 digital melting point apparatus (Mettler Toledo, OH, USA) and were uncorrected. The IR spectra were obtained on a Shimadzu, IR Prestige-21 (Shimadzu, Tokyo, Japan). ^1^H NMR, HSQC, HMBC, COSY and ^13^C NMR spectra of the synthesised compounds were registered by a Bruker 300 MHz and 75 MHz digital FT-NMR spectrometer (Bruker Bioscience, Billerica, MA, USA) in DMSO-*d_6_*, respectively. Splitting patterns were designated as follows: s: singlet; d: doublet; t: triplet; m: multiplet in the NMR spectra. Coupling constants (J) were reported as Hertz. M + 1 peaks were determined by Shimadzu LC/MS ITTOF system (Shimadzu, Tokyo, Japan). All reactions were monitored by thin-layer chromatography (TLC) using Silica Gel 60 F254 TLC plates (Merck KGaA, Darmstadt, Germany).

#### 2.1.1. General procedure for the synthesis of the compounds

##### 2.1.1.1. Synthesis of 2-(4-substitutedphenyl)-1H-benzo[d]imidazole-6-carboxylic acid derivatives (1a-1c)

A mixture of 4-substituted benzaldehyde (0.03 mol) and sodium disulphide (0.03 mol, 5.7 g) in DMF were treated under microwave irradiation (Anton-Paar Monowave 300) at 240 °C and 10 bar for 5 min. After cooling the mixture, 3,4-diamino benzoic acid (0.03 mol, 4.56 g) was added and kept under the same reaction conditions. The progress of the reaction was monitored by TLC. After completion of the reaction, the mixture was poured into crushed ice. The solid obtained was filtered, washed with water, dried and recrystallized from EtOH.

##### 2.1.1.2. Synthesis of 2-chloro-1-(4-sübstitüepiperaz-1-yl) ethan-1-one derivatives (1d-1h)

Chloroacetyl chloride (0.014 mol, 1.056 ml) in THF (15 ml) was taken into ice bath. 4-Substituted piperazine derivatives (0.012 mol) and TEA (0.0132 mol, 1.90 ml) in THF (50 ml) were added dropwise to this solution. After completion of dropping, the reaction mixture was stirred at room temperature for 1 h. The precipitated product was filtered washed with water and dried.

##### 2.1.1.3. Synthesis of Methyl 2-(4-substitutedphenyl)-1H-benzo[d]imidazole-6-carboxylate derivatives (2a-2c)

2–(4-Substitutedphenyl)-1*H*-benzo[*d*]imidazole-6-carboxylic acid (**1a**-**1c**) derivative compound (0.025 mol) was dissolved in methanol and several drops of sulphuric acid were added into the mixture. The mixture was refluxed for 72 h, and the precipitate was filtered off.

##### 2.1.1.4. Synthesis of 2-(4-Substitutedphenyl)-1H-benzo[d]imidazole-6-carbohydrazide derivatives (3a-3c)

Methyl 2–(4-substitutedphenyl)-1*H*-benzo[*d*]imidazole-6-carboxylate (**2a**-**2c**) (0.018 mol) and excess of hydrazine hydrate (5 ml) in EtOH (15 ml) were mixed in a vial (30 ml) of microwave synthesis reactor (Anton-Paar Monowave 300). The reaction mixture was heated at 240 °C and 10 bar for 10 min. After cooling, the mixture was poured into iced water, the product was filtered, washed with water, dried, and recrystallized from EtOH.

##### 2.1.1.5. Synthesis of 2-((4-Substituted phenyl)-(6-(5-mercapto-1,3,4-oxadiazol-2-yl)-1H-benzo [d]imidazole derivatives (4a-4c)

2–(4-Substitutedphenyl)-1*H*-benzo[*d*]imidazole-6-carbohydrazide (**3a**-**3c**) derivative (0.01 mol) was dissolved in a solution of NaOH (0.01 mol, 0.4 g) in ethanol and carbon disulphide (0.01 mol, 0.60 ml) was added in the mixture. The mixture was refluxed for 8 h. When the reaction was finished, the solution was cooled and acidified to pH 4–5 with concentrated hydrochloric acid solution to obtained target compound.

##### 2.1.1.6. General synthesis method of target compounds (5a-5o)

2-((4-Substitutedphenyl)-(6–(5-mercapto-1,3,4-oxadiazol-2-yl)-1*H*-benzo[*d*]imidazole (**4a**-**4c**) (0.001 mol), potassium carbonate (0.001 mol, 0.138 g) and 2-chloro-1-(4-substitutedepiperazin-1-yl) -ethane-1-on (0.0015 mol) derivative were dissolved in acetone and refluxed for 6 h. After TLC control, the solvent was evaporated, the residue was washed with water, dried and then recrystallized from ethanol to afford final compounds (**5a**-**5o**).

###### Methyl 2-(4-hydroxyphenyl)-1H-benzo[d]imidazole-6-carboxylate (2a)

Yield: 75%, M.P. = 306.1–307.5 °C. FTIR (ATR, cm^−1^): 3334 (N-H), 1687 (C=O), 844 (1,4-disubstituted benzene). ^1^H-NMR (300 MHz, DMSO-*d_6_*): δ = 3.88 (3H, *s*, –CH_3_), 6.95 (2H, d, *J* = 8.70 Hz, 1,4-disubstituted benzene), 7.62 (1H, d, *J* = 8.34 Hz, benzimidazole-C_4_), 7.82 (1H, dd, *J_1_*=8.43 Hz, *J_2_*=1.26 Hz, benzimidazole-C_5_), 8.04 (2H, d, *J* = 8.64 Hz, 1,4-disubstituted benzene), 8.14 (1H, *s*, benzimidazole-C_7_), 10.05 (1H, *s*, N–H). ^13^C-NMR (75 MHz, DMSO-*d_6_*): δ = 52.41, 107.48, 111.94, 116.26 (2C), 120.89, 123.40, 126.28, 129.02, 130.54 (2C), 134.46, 154.79, 160.14, 167.28. HRMS (*m*/*z*): [M + H]^+^ calcd for C_15_H_13_N_2_O_3_: 269.0910; found: 269.0921.

###### Methyl 2-(4-methoxyphenyl)-1H-benzo[d]imidazole-6-carboxylate (2 b)

Yield: 68%, M.P.=230.4–231.7 °C. FTIR (ATR, cm^−1^): 3250 (N–H), 1695 (C=O), 839 (1,4-disubstituted benzene). ^1^H-NMR (300 MHz, DMSO-*d_6_*): δ = 3.85 (3H, *s*, –OCH_3_), 3.87 (3H, *s*, –COOCH_3_), 7.13 (2H, d, *J* = 8.85 Hz, 1,4-disubstituted benzene), 7.65 (1H, br.s., benzimidazole-C–H), 7.83 (1H, d, *J* = 7.95 Hz, benzimidazole C–H), 8.14 (2H, d, *J* = 8.85 Hz, 1,4-disubstituted benzene), 8.22 (1H, br.s., benzimidazole, C-H). ^13^C-NMR (75 MHz, DMSO-*d_6_*): δ = 52.43, 55.87, 111.49, 113.08, 115.31 (2C), 118.69, 120.49, 122.45, 123.29, 123.56, 128.88 (2C), 130.43, 161.55, 167.26. HRMS (*m*/*z*): [M + H]^+^ calcd for C_16_H_15_N_2_O_3_: 283.1083; found: 283.1077.

###### Methyl 2-(4-ethoxyphenyl)-1H-benzo[d]imidazole-6-carboxylate (2c)

Yield: 60%, M.P.= 252.8–254.4 °C. FTIR (ATR, cm^−1^): 3477 (N-H), 1645 (C=O), 844 (1,4-disubstituted benzene). ^1^H-NMR (300 MHz, DMSO-*d_6_*): δ = 1.36 (3H, *t*, *J* = 6.96 Hz, –CH_3_), 3.87 (3H, *s*, –CH_3_), 4.11 (2H, *q*, *J* = 6.96 Hz, -CH_2_), 7.11 (2H, d, *J* = 8.88 Hz, 1,4-disubstituted benzene), 7.63 (1H, d, *J* = 8.43 Hz, benzimidazole-C_4_), 7.83 (1H, dd, *J_1_*=8.40 Hz, *J_2_*=1.59 Hz, benzimidazole-C_5_), 8.12 (2H, d, *J* = 8.88 Hz, 1,4-disubstituted benzene), 8.1 (1H, *s*, benzmidazole-C_7_), 13.07 (1H, *s*, N-H). ^13^C-NMR (75 MHz, DMSO-*d_6_*): δ = 15.04, 52.38, 63.81, 114.39, 115.33, 116.22 (2C), 118.31, 119.34, 122.31, 123.53, 128.49 (2C), 128.88, 154.40, 160.84, 167.26. HRMS (m/z): [M + H]^+^ calcd for C_17_H_17_N_2_O_3_: 297.1232; found: 297.1234.

###### 2-((4-Hydroxyphenyl)-(6-(5-mercapto-1,3,4-oxadiazol-2-yl)-1H-benzo[d]imidazole (4a)

Yield: 81%, M.P.= 226.8–228.3 °C. FTIR (ATR, cm^−1^): 3483 (N-H), 1645 (C=O), 844 (1,4-disubstituted benzene). ^1^H-NMR (300 MHz, DMSO-*d_6_*):δ = 7.06 (2H, d, *J* = 8.76 Hz, 1,4-disubstituted benzene), 7.89 (2H, *m*, benzimidazole-C–H), 8.10 (1H, *s*, benzimidazole-C_-_H), 8.24 (2H, d, *J* = 8.70 Hz, 1,4-disubstituted benzene), 10.75 (1H, *s*, O-H). ^13^C-NMR (75 MHz, DMSO-*d_6_*): δ = 112.11, 115.27, 115.48, 116.86 (2C), 118.85, 122.86, 130.64 (2C), 134.41, 136.72, 152.40, 160.92, 162.58, 177.83. HRMS (*m*/*z*): [M + H]^+^ calcd for C_15_H_11_N_4_O_2_S: 311.0595; found: 311.0597.

###### 2-((4-Methoxyphenyl)-(6-(5-mercapto-1,3,4-oxadiazol-2-yl)-1H-benzo[d]imidazole (4b)

Yield: 78%, M.P.= 274.4–276.5 °C. FTIR (ATR, cm^−1^): 3381 (N-H), 1633 (C=O), 837 (1,4-disubstituted benzene). ^1^H-NMR (300 MHz, DMSO-*d_6_*): δ = 3.90 (3H, *s*, –OCH_3_), 7.24–7.28 (2H, *m*, 1,4-disubstituted benzene), 7.89 (1H, *s*, benzimidazole C-H), 8.12 (1H, *s*, benzimidazole C–H), 8.32 (2H, d, *J* = 8.70 Hz, 1,4-disubstituted benzene), 8.40 (1H, *s*, benzimidazole C-H). ^13^C-NMR (75 MHz, DMSO-*d_6_*): δ = 56.25, 111.07, 114.23, 115.49, 115.76 (2C), 119.97, 122.60, 130.24 (2C), 130.49, 133.74, 152.25, 160.86, 162.73, 177.22. HRMS (*m*/*z*): [M + H]^+^ calcd for C_16_H_13_N_4_O_2_S: 325.0748; found: 325.0754.

###### 2-((4-Ethoxyphenyl)-(6–(5-mercapto-1,3,4-oxadiazol-2-yl)-1H-benzo[d]imidazole (4c)

Yield: 84%, M.P.= 288.2–289.9 °C. FTIR (ATR, cm^−1^): 3402 (N-H), 1624 (C=O), 846 (1,4-disubstituted benzene). ^1^H-NMR (300 MHz, DMSO-*d_6_*): δ = 1.38 (3H, *t*, *J* = 6.90 Hz, -CH_3_), 4.17 (2H, q, *J* = 6.96 Hz, -CH_2_), 7.23 (2H, d, *J* = 8.28 Hz, 1,4-disubstituted benzene), 7.91–7.93 (2H, *m*, benzimidazole-C_-_H), 8.12 (1H, *s*, benzimidazole-C_-_H), 8.24 (2H, d, *J* = 7.47 Hz, 1,4-disubstituted benzene). ^13^C-NMR (75 MHz, DMSO-*d_6_*): δ = 14.92, 64.35, 112.17, 115.55, 115.96, 116.14 (2C), 119.30, 123.28, 130.57 (2C), 133.78, 135.98, 151.83, 155.14, 162.99, 177.88. HRMS (m/z): [M + H]^+^ calcd for C_17_H_15_N_4_O_2_S: 339.0910; found: 339.0910.

###### 2-((5–(2-(4-Hydroxyphenyl)-1H-benzo[d]imidazol-6-yl)-1,3,4-oxadiazol-2-yl)thio)-1–(4-methyl- piperazin-1-yl) -ethane-1-on (5a)

Yield: 65%, M.P.= 161.3–163.4 °C. FTIR (ATR, cm^−1^): 3618 (N-H), 1645 (C=O), 840 (1,4-disubstituted benzene). ^1^H-NMR (300 MHz, DMSO-*d_6_*): δ = 2.76 (3H, *s*, -CH_3_), 3.35 (8H, br.s., piperazine), 4.64 (2H, *s*, -CH_2_), 6.96 (2H, d, *J* = 8.64 Hz, 1,4-disubstituted benzene), 7.71 (1H, d, *J* = 8.28 Hz, benzimidazole-C_4_), 7.80 (1H, dd, *J_1_*=8.40 Hz, *J_2_*=1.15 Hz, benzimidazole-C_5_), 8.09 (2H, d, *J* = 8.61 Hz, 1,4-disubstituted benzene), 8.13 (1H, *s*, benzimidazole-C_7_), 10.20 (1H, *s*, O-H). ^13^C-NMR (75 MHz, DMSO-*d_6_*): δ = 36.76, 42.47, 45.75, 52.23, 52.44, 60.63, 112.96, 115.85, 116.47 (2C), 117.52, 118.66, 121.59, 129.67 (2C), 153.79, 161.14, 163.26, 165.68, 166.19, 167.30, 169.40. HRMS (m/z): [M + H]^+^/2 calcd for C_22_H_23_N_6_O_3_S: 226.0804; found: 226.0810.

###### 2-((5–(2-(4-Hydroxyphenyl)-1H-benzo[d]imidazol-6-yl)-1,3,4-oxadiazol-2-yl)thio)-1–(4-ethyl- piperazin-1-yl) -ethane-1-on (5 b)

Yield: 78%, M.P.= 199.9–201.2 °C. FTIR (ATR, cm^−1^): 3311 (N-H), 1645 (C=O), 839 (1,4-disubstituted benzene). ^1^H-NMR (300 MHz, DMSO-*d_6_*): δ = 1.01 (3H, *t*, *J* = 7.16, CH_3_), 2.34–2.51 (6H, *m*, CH_2_, piperazine), 3.48–3.50 (4H, *m*, piperazine), 4.56 (2H, *s*, CH_2_), 6.90 (2H, d, *J* = 8.70 Hz, 1,4-disubstituted benzene), 7.67 (1H, d, *J* = 8.46 Hz, benzimidazole-C_4_), 7.74 (1H, dd, *J_1_*=8.39 Hz, *J_2_*:1.65 Hz, benzimidazole-C_5_), 8.06 (2H, d, *J* = 8.70 Hz, 1,4-disubstituted benzene), 8.09 (1H, *s*, benzimidazole-C_7_). ^13^C-NMR (75 MHz, DMSO-*d_6_*): δ = 12.32, 37.04, 42.23, 45.85, 51.91, 52.31, 52.79, 113.41, 116.30, 116.45, 116.70 (2C), 120.38, 129.02 (2C), 155.20, 155.45, 161.21, 163.08, 165.06, 165.89, 166.58, 168.44. HRMS (*m*/*z*): [M + H]^+^ calcd for C_23_H_25_N_6_O_3_S: 465.1692; found: 465.1703.

###### 2-((5–(2-(4-Hydroxyphenyl)-1H-benzo[d]imidazol-6-yl)-1,3,4-oxadiazol-2-yl) thio)-1–(4-(phenyl) -piperazin-1-yl) -ethane-1-on (5c)

Yield: 72%, M.P.= 271.1–273.4 °C. FTIR (ATR, cm^−1^): 3365 (N-H), 1645 (C=O), 840 (1,4-disubstituted benzene). ^1^H-NMR (300 MHz, DMSO-*d_6_*): δ = 3.13–3.26 (4H, *m*, piperazine), 3.63–3.70 (4H, *m*, piperazine), 4.64 (2H, *s*, -CH_2_), 6.82 (1H, *t*, *J* = 7.23 Hz, phenyl C-H), 6.99–6.93 (4H, *m*, 1,4-disubstituted benzene, phenyl C-H), 7.24 (2H, *t*, *J* = 7.29 Hz, phenyl C-H), 7.66 (1H, br.s., benzimidazole-C_4_), 7.81 (1H, d, *J* = 8.34 Hz, benzimidazole-C_5_), 8.01–8.06 (3H, *m*, 1,4-disubstituted benzene, benzimidazole-C_7_), 10.10 (1H, *s*, O-H), 13.09 (1H, *s*, N-H).^13^C-NMR (75 MHz, DMSO-*d_6_*): δ = 37.03, 42.06, 45.66, 48.63, 48.97, 116.21 (2C), 116.28, 116.37, 116.77 (2C), 119.18, 119.85, 120.83, 121.11, 128.86, 129.03 (2C), 129.48 (2C), 151.15, 159.93, 160.18, 162.08, 163.22, 165.26, 166.46. HRMS (*m*/*z*): [M + H]^+^ calcd for C_27_H_25_N_6_O_3_S: 513.1699; found: 513.1703.

###### 2-((5-(2-(4-Hydroxyphenyl)-1H-benzo[d]imidazol-6-yl)-1,3,4-oxadiazol-2-yl)thio)-1-(4-(pyridin-2-yl)-piperazin-1-yl)-ethane-1-on (5d)

Yield: 69%, M.P.= 247.6–248.9 °C. FTIR (ATR, cm^−1^): 3628 (N-H), 1645 (C=O), 840 (1,4-disubstituted benzene). ^1^H-NMR (300 MHz, DMSO-*d_6_*): δ = 3.56–3.66 (8H, *m*, piperazine), 4.65 (2H, *s*, CH_2_), 6.68–6.69 (2H, *m*, pyridine C-H), 6.99 (2H, d, *J* = 8.73 Hz, 1,4-disubstituted benzene), 7.57–7.58 (2H, *m*, pyridine C-H), 7.72 (1H, d, *J* = 8.37 Hz, benzimidazole-C_4_), 7.82 (1H, dd, *J_1_*=5.40 Hz, *J_2_*=1.53 Hz, benzimidazol-C_5_), 8.11–8.14 (2H, *m*, 1,4-disubstituted benzene, benzimidazole-C_7_), 10.26 (1H, *s*, O-H). ^13^C-NMR (75 MHz, DMSO-*d_6_*): δ = 37.17, 41.83, 44.74, 44.99, 45.41, 107.88, 113.21, 113.79, 114.59, 116.27 (2C), 116.73, 117.11, 120.67, 120.74, 129.13 (2C), 138.24, 147.83, 154.66, 158.94, 160.34, 163.22, 165.40, 165.49, 166.47. HRMS (m/z): [M + H]^+^ calcd for C_26_H_24_N_7_O_3_S: 514.1636; found: 514.1656.

###### 2-((5–(2-(4-Hydroxyphenyl)-1H-benzo[d]imidazol-6-yl)-1,3,4-oxadiazol-2-yl)thio)-1-(4-(pyrimidine-2-yl)-piperazin-1-yl)-ethane-1-on (5e)

Yield: 78%, M.P.= 181.4–183.2 °C. FTIR (ATR, cm^−1^): 3587 (N-H), 1645 (C=O), 840 (1,4-disubstituted benzene). ^1^H-NMR (300 MHz, DMSO-*d_6_*): δ = 3.60 (2H, br.s, piperazine), 3.67 (2H, br.s., piperazine), 3.79 (2H, br.s., piperazine), 3.90 (2H, brs., piperazine), 4.64 (2H, *s*, CH_2_), 6.74 (1H, *J* = 4.80, pyrimidine), 7.09 (2H, *J* = 8.76 Hz, 1,4-disubstituted benzene), 7.92 (1H, d, *J* = 8.55 Hz, benzimidazole-C_4_), 8.03, (1H, dd, *J_1_*=8.60 Hz, *J_2_*=1.17 Hz, benzimidazole-C_5_), 8.21 (1H, *s*, benzimidazole-C_7_), 8.35 (2H, d, *J* = 8.73 Hz, 1,4-disubstituted benzene), 8.44 (2H, d, *J* = 4.83 Hz, pyrimidine). ^13^C-NMR (75 MHz, DMSO-*d_6_*): δ = 37.43, 41.82, 43.67, 43.95, 45.37, 110.86, 111.87, 113.24, 115.16, 117.01 (2C), 120.25, 124.09, 131.19 (2C), 132.41, 134.34, 151.27, 158.26, 159.99, 163.40, 164.30, 165.12, 165.37. HRMS (*m/z*): [M + H]^+^ calcd for C_25_H_23_N_8_O_3_S: 515.1610; found: 515.1608.

###### 2-((5-(2-(4-Methoxyphenyl)-1H-benzo[d]imidazol-6-yl)-1,3,4-oxadiazol-2-yl) thio)-1–(4-methyl- piperazin-1-yl)-ethane-1-on (5f)

Yield: 74%, M.P.= 156.6–158.7 °C. FTIR (ATR, cm^−1^): 3446 (N-H), 1645 (C=O), 835 (1,4-disubstituted benzene). ^1^H-NMR (300 MHz, DMSO-*d_6_*): δ = 2.78 (3H, *s*, -CH_3_), 3.43 (6H, br.s., piperazine), 3.65 (2H, br.s., piperazine), 3.87 (3H, *s*, –OCH_3_), 4.64 (2H, *s*, -CH_2_), 7.17 (2H, d, *J* = 8.91 Hz, 1,4-disubstituted benzene), 7.78 (1H, d, *J* = 8.46 Hz, benzimidazole-C_4_), 7.87 (1H, dd, *J_1_*= 8.43 Hz, *J_2_*= 1.50 Hz benzimidazole-C_5_), 8.16 (1H, br.s. benzimidazole-C_7_), 8.25 (2H, d, *J* = 8.82 Hz, 1,4-disubstituted benzene), 11.38 (1H, *s*, N-H).^13^C-NMR (75 MHz, DMSO-*d_6_*): δ = 36.82, 42.40, 42.79, 45.66, 52.19, 52.42, 56.00, 113.15, 113.44, 115.09 (2C), 115.98, 117.70, 120.46, 121.70, 124.59, 129.56 (2C), 153.34, 162.18, 163.31, 165.67, 166.15. HRMS (*m*/*z*): [M + H]^+^ calcd for C_23_H_25_N_6_O_3_S: 465.1703; found: 465.170.

###### 2-((5-(2-(4-Methoxyphenyl)-1H-benzo[d]imidazol-6-yl)-1,3,4-oxadiazol-2-yl)thio)-1–(4-ethyl-piperazin-1-yl)-ethane-1-on (5 g)

Yield: 66%, M.P.= 180.4–181.9 °C. FTIR (ATR, cm^−1^): 3334 (N-H), 1645 (C=O), 840 (1,4-disubstituted benzene). ^1^H-NMR (300 MHz, DMSO-*d_6_*): δ = 1.01 (3H, *t*, *J* = 7.08 Hz, CH_3_), 2.34 (2H, *m*, CH_2_), 3.85 (3H, *s*, OCH_3_), 3.47–3.53 (8H, *m*, piperazine), 4.56 (2H, *s*, CH_2_), 7.13 (2H, d, *J* = 8.79 Hz, 1,4-disubstituted benzene), 7.71 (1H, d, *J* = 8.40 Hz, benzimidazole-C_4_), 7.80 (1H, dd, *J_1_*=8.42 Hz, *J_2_*=1.44 Hz benzimidazole-C_5_), 8.13 (1H, br.s., benzimidazole-C_7_), 8.17 (2H, d, *J* = 8.88 Hz, 1,4-disubstituted benzene). ^13^C-NMR (75 MHz, DMSO-*d_6_*): δ = 12.36, 37.10, 42.23, 45.85, 51.92, 52.32, 52.80, 55.85, 114.88 (2C), 119.77, 120.02, 120.63, 122.75, 128.71, 128.93 (2C), 153.30, 154.66, 161.25, 161.42, 163.19, 165.05, 166.48. HRMS (m/z): [M + H]^+^ calcd for C_24_H_27_N_6_O_3_S: 479.1872; found: 479.1860.

###### 2-((5-(2-(4-Methoxyphenyl)-1H-benzo[d]imidazol-6-yl)-1,3,4-oxadiazol-2-yl)thio)-1–(4-(phenyl)-piperazin-1-yl)-ethane-1-on (5 h)

Yield: 77%, M.P.= 166.7–169.2 °C. FTIR (ATR, cm^−1^): 3367 (N-H), 1645 (C=O), 840 (1,4-disubstituted benzene). ^1^H-NMR (300 MHz, DMSO-*d_6_*): δ = 3.19 (4H, br.s., piperazine), 3.67 (4H, br.s., piperazine), 3.85 (3H, *s*, -OCH_3_), 4.63 (2H, *s*, -CH_2_), 6.81 (1H, *t*, *J* = 7.14 Hz, phenyl C-H), 6.97 (2H, d, *J* = 7.92 Hz, phenyl C-H), 7.14 (2H, d, *J* = 8.79 Hz, 1,4-disubstituted benzene), 7.23 (2H, d, *J* = 7.47 Hz, phenyl C-H), 7.76 (1H, d, *J* = 8.46 Hz, benzimidazole C_4_), 7.80 (1H, dd, *J_1_*= 8.37 Hz, *J_2_*= 1.20 Hz, benzimidazole-C_5_), 8.16 (1H, br.s. benzimidazole-C_7_), 8.29 (2H, d, *J* = 8.70 Hz, 1,4-disubstituted benzene), 10.72 (1H, *s*, N-H). ^13^C-NMR (75 MHz, DMSO-*d_6_*): δ = 42.23, 45.85, 51.92, 52.32, 52.80, 55.85, 114.88 (2C), 116.12, 116.63 (2C), 117.06, 119.77, 120.02, 120.63, 122.75, 122.86 128.71 (2C), 128.93 (2C), 153.30, 154.66, 161.25, 161.42, 163.19 165.05, 166.48. HRMS (*m*/*z*): [M + H]^+^ calcd for C_28_H_27_N_6_O_3_S: 527.1849; found: 527.1860.

###### 2-((5-(2-(4-Methoxyphenyl)-1H-benzo[d]imidazol-6-yl)-1,3,4-oxadiazol-2-yl)thio)-1–(4-(pyridin-2-yl)-piperazin-1-yl)-ethane-1-on (5ı)

Yield: 72%, M.P.= 168.3–170.1 °C. FTIR (ATR, cm^−1^): 3350 (N-H), 1651 (C=O), 829 (1,4-disubstituted benzene). ^1^H-NMR (300 MHz, DMSO-*d_6_*): δ = 3.52–3.54 (2H, m, piperazine), 3.61–3.65 (6H, *m*, piperazine), 3.83 (3H, *s*, -OCH_3_), 4.62 (2H, *s*, CH_2_), 6.65–6.69 (1H, *m*, pyridine C-H), 6.85–6.87 (1H, *m*, pyridine C-H), 7.07 (2H, d, *J* = 8.91 Hz, 1,4-disubstituted benzene), 7.53–7.58 (1H, *m*, pyridine C-H), 7.64 (1H, *s*, benzimidazole-C_4_), 7.70 (1H, dd, *J_1_*=8.37 Hz, *J_2_*=1.50 Hz, benzimidazole-C_5_), 8.09 (1H, *s*, benzimidazole-C_7_), 8.12–8.14 (1H, *m*, pyridine C-H), 8.20 (2H, d, *J* = 8.85 Hz, 1,4-disubstituted benzene). ^13^C-NMR (75 MHz, DMSO-*d_6_*): δ = 37.12, 41.83 44.72, 44.97, 45.42, 55.75, 107.77, 113.80, 114.03, 114.61, 114.82, 115.23 (2C), 116.07, 119.53, 124.75, 128.70 (2C), 128.80, 138.12, 142.46, 148.04, 159.09, 160.87, 162.71, 165.43, 167.01. HRMS (*m*/*z*): [M + H]^+^ calcd for C_27_H_26_N_7_O_3_S: 528.1825; found: 528.1812.

###### 2-((5-(2-(4-Methoxyphenyl)-1H-benzo[d]imidazol-6-yl)-1,3,4-oxadiazol-2-yl)thio)-1–(4-(pyrimidin-2-yl)-piperazin-1-yl)-ethane-1-on (5j)

Yield: 80%, M.P.= 118.7–120.3 °C. FTIR (ATR, cm^−1^): 3406 (N-H), 1627 (C=O), 839 (1,4-disubstituted benzene). ^1^H-NMR (300 MHz, DMSO-*d_6_*): δ = 3.64 (2H, br.s, piperazine), 3.72–3.76 (6H, *m*, piperazine), 3.82 (3H, *s*, -OCH_3_), 4.62 (2H, *s*, CH_2_), 6.68 (1H, *t*, *J* = 4.71, pyrimidine), 7.12 (2H, *J* = 8.91 Hz, 1,4-disubstituted benzene), 7.70 (1H, d, *J* = 8.31 Hz, benzimidazole C_4_), 7.79 (1H, dd, *J_1_*= 8.43 Hz, *J_2_*= 1.47 Hz, benzimidazole-C_5_), 8.13 (1H, br.s. benzimidazole-C_7_), 8.20 (2H, *J* = 8.85 Hz, 1,4-disubstituted benzene), 8.40 (2H, d, *J* = 4.74 Hz, pyrimidine). ^13^C-NMR (75 MHz, DMSO-*d_6_*): δ = 37.22, 41.94, 43.77, 45.53, 52.71, 55.71, 110.54, 110.91, 110.96, 114.09, 114.52 (2C), 116.10, 119.28, 125.13, 128.82 (2C), 158.43, 158.46, 160.74, 161.53, 161.62, 162.61, 165.49, 167.55, 168.04. HRMS (*m*/*z*): [M + H]^+^ calcd for C_26_H_25_N_8_O_3_S: 529.1755; found: 529.1765.

###### 2-((5-(2-(4-Ethoxyphenyl)-1H-benzo[d]imidazol-6-yl)-1,3,4-oxadiazol-2-yl)thio)-1–(4-methyl-piperazin-1-yl)-ethane-1-on (5k)

Yield: 79%, M.P.= 181.2–183.5 °C. FTIR (ATR, cm^−1^): 3360 (N-H), 1645 (C=O), 833 (1,4-disubstituted benzene). ^1^H-NMR (300 MHz, DMSO-*d_6_*): δ = 1.37 (3H, *t*, *J* = 6.81 Hz, -CH_3_), 2.75 (3H, *s*, -CH_3_), 3.39 (4H, br.s., piperazine), 3.60 (4H, br.s., piperazine), 4.47–4.50 (2H, *m*, -CH_2_), 4.66 (2H, *s*, -CH_2_), 7.21 (2H, d, *J* = 8.67 Hz, 1,4-disubstituted benzene), 7.88 (1H, d, *J* = 8.67 Hz, benzimidazole-C_4_), 7.99 (1H, d, *J* = 8.55 Hz, benzimidazole-C_5_), 8.22 (1H, *s*, benzimidazole-C_7_), 8.38 (2H, d, *J* = 8.16 Hz, 1,4-disubstituted benzene), 11.55 (1H, *s*, N-H). ^13^C-NMR (75 MHz, DMSO-*d_6_*): δ = 14.96, 36.87, 39.11, 42.38, 42.74, 45.66, 52.18, 64.25, 112.48, 115.79 (2C), 117.36, 119.21, 121.51, 123.22, 126.03 (2C), 128.45, 130.51, 151.88, 162.62, 163.81, 165.63, 165.80. HRMS (m/z): [M + H]^+^ calcd for C_24_H_27_N_6_O_3_S: 479.1873; found: 479.1860.

###### 2-((5-(2-(4-Ethoxyphenyl)-1H-benzo[d]imidazol-6-yl)-1,3,4-oxadiazol-2-yl)thio)-1–(4-ethyl-piperazin-1-yl)-ethane-1-on (5 l)

Yield: 77%, M.P.= 109.4–110.8 °C. FTIR (ATR, cm^−1^): 3429 (N-H), 1653 (C=O), 827 (1,4-disubstituted benzene). ^1^H-NMR (300 MHz, DMSO-*d_6_*): δ = 1.19–1.21 (3H, *m*, -CH_3_), 1.36 (3H, *t*, *J* = 6.90 Hz, -CH_3_), 2.93–2.98 (2H, *m*, -CH_2_), 3.80–3.90 (8H, *m*, piperazine), 4.10–4.13 (2H, *m*, -CH_2_), 4.62 (2H, *s*, -CH_2_), 7.10 (2H, d, *J* = 8.91 Hz, 1,4-disubstituted benzene), 7.73 (1H, d, *J* = 8.40 Hz, benzimidazole-C_4_), 7.81 (1H, dd, *J_1_*=8.37 Hz, *J_2_*=1.50 Hz, benzimidazole-C_5_), 8.13 (1H, *s*, benzimidazole-C_7_), 8.22 (2H, d, *J* = 8.82 Hz, 1,4-disubstituted benzene). ^13^C-NMR (75 MHz, DMSO-*d_6_*): δ = 15.06, 36.81, 37.88, 43.45, 50.57, 50.98, 51.22, 60.65, 63.81, 113.08, 115.28, 116.74 (2C), 120.76, 122.30, 125.99, 127.43, 129.02 (2C), 132.31, 154.34, 160.80, 163.07, 165.51, 165.61. HRMS (*m*/*z*): [M + H]^+^ calcd for C_25_H_29_N_6_O_3_S: 493.2019; found: 493.2016.

###### 2-((5-(2-(4-Ethoxyphenyl)-1H-benzo [d] imidazol-6-yl)-1,3,4-oxadiazol-2-yl) thio)-1–(4-(phenyl)-piperazin-1-yl)-ethane-1-on (5 m)

Yield: 70%, M.P.= 262.5–263.9 °C. FTIR (ATR, cm^−1^): 3367 (N-H), 1645 (C=O), 840 (1,4-disubstituted benzene). ^1^H-NMR (300 MHz, DMSO-*d_6_*): δ = 1.35 (3H, *t*, *J* = 6.93 Hz, -CH_3_), 2.32 (2H, br.s., piperazine), 2.41 (2H, br.s., piperazine), 3.52 (4H, br.s., piperazine), 4.11 (2H, q, *J* = 6.96 Hz, –CH_2_), 4.53 (2H, *s*, –CH_2_), 7.15 (2H, d, *J* = 8.94 Hz, 1,4-disubstituted benzene), 7.44–7.47 (3H, *m*, phenyl C-H), 7.66–7.69 (2H, *m*, phenyl C-H), 7.76 (1H, d, *J* = 8.46 Hz, benzimidazole-C_4_), 7.81–7.85 (1H, *m*, benzimidazole-C_5_), 8.15 (1H, br.s. benzimidazole-C_7_), 8.28 (2H, d, *J* = 8.85 Hz, 1,4-disubstituted benzene), 10.70 (1H, *s*, N-H). ^13^C-NMR (75 MHz, DMSO-*d_6_*): δ = 36.75, 42.72, 45.68, 50.38, 50.84, 55.91, 59.07, 114.95, 115.83 (2C), 117.01, 121.02, 121.93, 128.76 (2C), 129.03, 129.09, 129.18, 129.83, 130.20, 131.01, 131.89, 154.05, 161.69, 162.99, 163.10, 165.59, 166.41, 169.44. HRMS (*m*/*z*): [M + H]^+^ calcd for C_29_H_29_N_6_O_3_S: 541.2002; found: 541.2016.

###### 2-((5-(2-(4-Ethoxyphenyl)-1H-benzo[d]imidazol-6-yl)-1,3,4-oxadiazol-2-yl) thio-1–(4-(pyridin-2-yl)-piperazin-1-yl)-ethane-1-on (5n)

Yield: 71%, M.P. = 168.3–170.4 °C. FTIR (ATR, cm^−1^): 3361 (N-H), 1635 (C=O), 840 (1,4-disubstituted benzene). ^1^H-NMR (300 MHz, DMSO-*d_6_*): δ = 1.37 (3H, *t*, *J* = 6.93 Hz, –CH_3_), 3.52–3.54 (2H, *m*, piperazine), 3.59–3.65 (6H, *m*, piperazine), 4.13 (2H, *q*, *J* = 6.99 Hz, -CH_2_), 4.65 (2H, *s*, –CH_2_), 6.66–6.70 (1H, *m*, pyridine C–H), 6.88 (1H, d, *J* = 8.61 Hz, pyridine-C–H), 7.11 (2H, d, *J* = 8.85 Hz, 1,4-disubstituted benzene), 7.54–7.60 (1H, *m*, benzimidazole-C_4_), 7.80 (2H, *m*, pyridine C–H, benzimidazole C–H), 8.06–8.15 (2H, *m*, pyridine C–H, benzimidazole C–H), 8.18 (2H, d, *J* = 8.49 Hz, 1,4-disubstituted benzene), 13.50 (1H, *s*, N–H). ^13^C-NMR (75 MHz, DMSO-*d_6_*): δ = 15.07, 37.18, 41.84, 44.72, 44.98, 45.42, 63.81, 107.80, 113.82, 114.63 (2C), 115.31, 116.84, 119.76, 122.26, 123.67, 125.16, 128.96, 130.02 (2C), 138.15, 141.90, 148.04, 159.08, 160.83, 164.04, 165.40, 166.45. HRMS (*m*/*z*): [M + H]^+^ calcd for C_28_H_28_N_7_O_3_S: 542.1982; found: 542.1969.

###### 2-((5-(2-(4-Ethoxyphenyl)-1H-benzo[d]imidazol-6-yl)-1,3,4-oxadiazol-2-yl)thio)-1–(4-(pyrimidine-2-yl)-piperazin-1-yl)-ethane-1-on (5o)

Yield: 67%, M.P. = 118.7–120.5 °C. FTIR (ATR, cm^−1^): 3334 (N-H), 1645 (C=O), 871 (1,4-disubstituted benzene). ^1^H-NMR (300 MHz, DMSO-*d_6_*): δ = 1.37 (3H, *t*, *J* = 6.90 Hz, -CH_3_), 3.59 (2H, br.s., piperazine), 3.65 (2H, br.s., piperazine), 3.75–3.76 (4H, *m*, piperazine), 4.11 (2H, *q*, *J* = 6.99 Hz, –CH_2_), 4.64 (2H, *s*, –CH_2_), 6.67 (1H, *t*, *J* = 4.77 Hz, pyrimidine), 8.09 (2H, d, *J* = 8.73 Hz, 1,4-disubstituted benzene), 7.70 (1H, d, *J* = 7.70 Hz, benzimidazole-C_4_), 7.79 (1H, dd, *J_1_* = 8.40 Hz, *J_2_* = 1.59 Hz, benzimidazole-C_5_), 7.89 (1H, *s*, benzimidazole-C_7_), 8.15 (2H, d, *J* = 8.73 Hz, 1,4-disubstituted benzene), 8.40 (2H, d, *J* = 4.71 Hz, pyrimidine). ^13^C-NMR (75 MHz, DMSO-*d_6_*): δ = 15.08, 37.25, 41.95, 43.41, 43.68, 45.53, 63.76, 110.99, 115.23 (2C), 116.27, 119.73, 119.96, 120.37, 122.71, 123.06, 128.72 (2C), 128.91, 153.41, 158.48, 160.54, 162.13, 163.06, 165.46, 166.66, 179.63. HRMS (*m*/*z*): [M + H]^+^ calcd for C_27_H_27_N_8_O_3_S: 543.1907; found: 543.1921.

### 2.2. 2 D NMR

2D NMR studies including HSQC (Heteronuclear single-quantum correlation spectroscopy), HMBC (Heteronuclear multiple-bond correlation spectroscopy) and COSY (Correlation Spectroscopy) were performed for compound **5e** (Table 1 and Supporting information).

**Table 1.** Hydrogen and carbon values of compound **5e** detected by 2 D NMR.


Position	^1^H	^13^C
**1**	6.68	111.0
**2**	8.39	158.5
**3**	–	161.4
**4**	3.60–3.86	42.0–45.5
**5**	3.65–3.77	43.4–43.7
**6**	–	165.4
**7**	4.62	37.3
**8**	–	152.3
**9**	–	165.3
**10**	–	136.0
**11**	7.98	123.6
**12**	7.86	115.4
**13**	8.15	112.3
**14**	–	119.7
**15**	–	134.0
**16**	–	164.2
**17**	–	114.8
**18**	8.22	130.7
**19**	7.06	116.9
**20**	–	162.8
**21**	10.88	–
**22**	12.69	–

### 2.3. Biological activity

#### 2.3.1. Cytotoxicity test

The anticancer activity of compounds **5a**–**5o** were screened according to the MTT assays. The MTT assays were performed as previously described^32^^,^^33^. Anticancer activity of final compounds was assessed against five different cancer cell lines A549 (lung carcinoma cell line), HeLa (cervical cell line), MCF-7 (human breast adenocarcinoma cell line), HepG2 (human liver carcinoma cell line) and C6 (rat glioma cell line) cell lines as well as NIH3T3 (mouse embryo fibroblast cell line). Doxorubicin and Hoechst 33342 were used as the reference drugs in the MTT assays.

#### 2.2.2. DNA synthesis inhibition assay

The BrdU cell proliferation method was performed to analyse the effects of the active compounds on proliferation of cancer cells. Cancer cells were seeded into the 96-well plates at a density of 1 × 10^4^ cells. Compounds were added into the each well at three different concentrations (IC_50/2_, IC_50_ and 2xIC_50_) and the plates were incubated for 24 h. At the end of the incubation period, BrdU solution was added and cells were reincubated for 2 h at 37 °C. Anti-BrdU-POD (100 ml) was added and the mixture was incubated for 90 min. Microplates were washed with PBS for three times. After adding substrate solution, the mixture was incubated for 15 min. OD of the wells were determined at 492 nm. Proliferation of control cells was assessed as 100% and growth inhibition % of cells, treated with test compounds and cisplatin were calculated^34^.

#### 2.2.3. Flow cytometric analysis

Death pathway of the carcinogenic cell lines was detected by Annexin V-FITC Apoptosis Detection Kit (BD, Pharmingen) as reported previously described^35^. Doxorubicin and compounds, which possess the highest cytotoxic activity, were used at their IC_50_ concentration. FCS Express software was used to display the percent of normal and apoptotic cells at different stages. In the diagrams, Q1, Q2, Q3, and Q4 demonstrates the necrotic cells (positive for PI and negative for annexin/FITC), late apoptotic or necrotic cells (positive for annexin and PI), live cells (negative for annexin and PI), and apoptotic cells (negative for PI and positive for annexin), respectively. The experiments were carried out in triplicates.

#### 2.2.4. DNA topoisomerase I assay

In this study, topoisomerase I assay kit (TG1018‐2; TopoGen) was used to determine if synthesised compounds showed topoisomerase I inhibition. The topoisomerase I inhibition activities of final compounds were measured as relaxation of supercoiled plasmid DNA using agarose gel electrophoresis and camptothecin was used as a positive control. The assay was carried out in a final volume of 20 µl reaction volume containing 2 µl of 10xTGS Buffer, 6 µl water, 2 µl supercoiled plasmid DNA, 2 µl test compound, 2 µl of Top1, 2 ul 10% SDS, 2 µl proteinase K, 2 µl DNA loading dye. After incubation of reaction mixtures at 37 °C for 30 min, electrophoresis was done on a 1% agarose gel at a potential 50 V for 75 min using 1xTAE buffer.

### 2.3. Molecular docking

A structure-based *in silico* docking method was applied to determine the binding and interaction modes of compound **5n** that show significant anticancer activity in the series, in the active region of the DNA-Topoisomerase I enzyme complex. A protein–ligand interaction analysis was performed on the DNA-Topoisomerase I enzyme complex by using X-ray crystal structure retrieved from Protein Data Bank server (www.pdb.org) (PDB Code: 1T8I)^35^.

The chemical structures of ligands were drawn using the Schrödinger Maestro^36^ interface and then were submitted to the Protein Preparation Wizard protocol of the Schrödinger Suite 2016 Update 2^37^. Then the ligands were prepared by the LigPrep 3.8^38^ to set to protonation states at pH 7.4 ± 1.0 and the atom types, correctly. Bond orders were assigned, and hydrogen atoms were added to the structures. The grid generation was formed using Glide 7.1^39^. Flexible docking runs were performed with single precision docking mode (SP).

## 3. Result and discussion

### 3.1. Chemistry

**Scheme 1.**
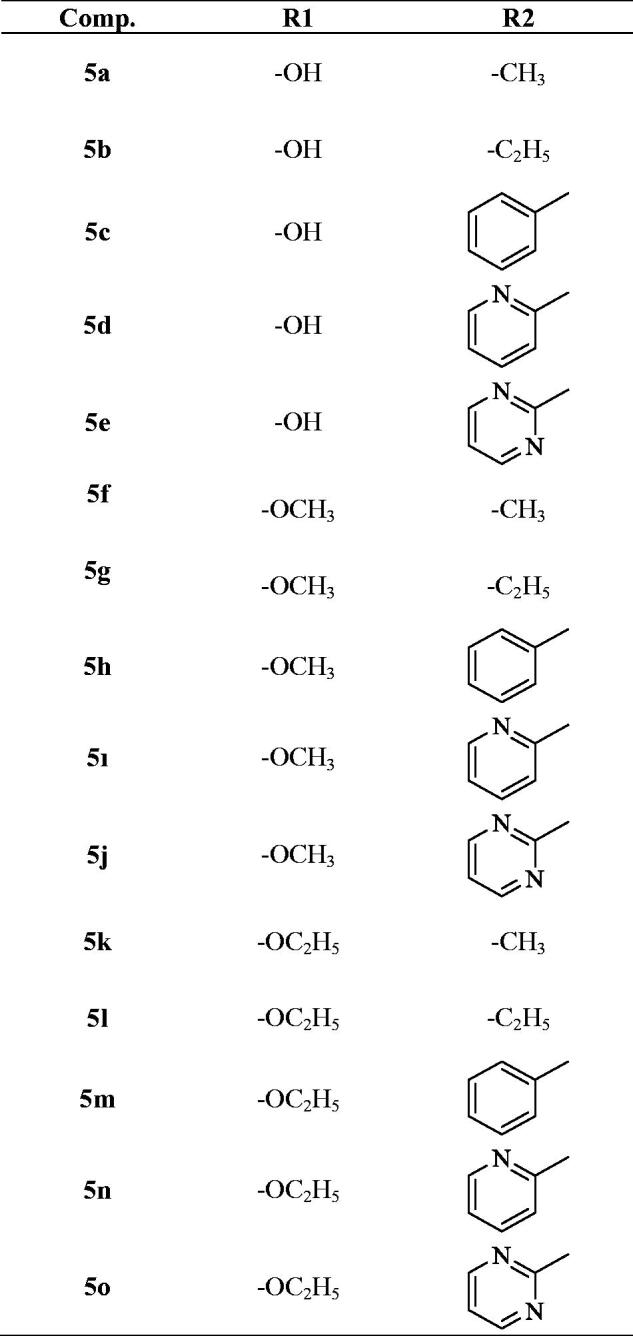
Continued.

The protocol adopted for synthesis of compounds (**5a**-**5o**) was shown in Scheme 1. In the first step, 2–(4-substitutedphenyl)-1*H*-benzo[*d*]imidazole-6-carboxylic acid (**1a**-**1c**) derivatives were obtained by the reaction of 4-substituted benzaldehyde with 3,4-diamino benzoic acid using sodium bisulphite in DMF. The compound (**1a-1c**) was converted to a methyl ester (**2a**-**2c**) by simple esterification reaction followed by treatment with hydrazine hydrate to obtain 2–(4-substitutedphenyl-1*H*-benzo[*d*]imidazole-6-carbohydrazide (**3a**-**3c**). The reaction of the hydrazide derivative (**3a**-**3c**) with carbon disulfide in boiling ethanol and KOH gave 2-((4-substitutedphenyl)-(6–(5-mercapto-1,3,4-oxadiazol-2-yl)-1*H*-benzo[*d*]imidazole derivative (**4a**-**4c**). At the last reaction step, the compound (**4a**-**4c**) was reacted with acetylated piperazine derivatives in acetone to produce 2-((5–(2-(4-substitutedphenyl)-1*H*-benzo[*d*]imidazol-6-yl)-1,3,4-oxadiazol-2-yl) thio)-1– (4-sübstitüepiperazin- 1-yl)-ethane-1-on derivatives (**5a**-**5o**).

After the isolation and purification, the structures of newly synthesised compounds (**5a**-**5o**) were characterised by using various modern analytical techniques like IR, ^1^H NMR, ^13^C NMR, 2 D-NMR and HRMS.

In general, for all the synthesised compounds, the stretching bands for C=O and N-H were observed between 1627–1653 cm^−1^ and 3311–3628 cm^−1^, respectively. The stretching absorption belonging to 1,4-disubstituted benzene was determined at 827–871 cm^−1^.

In the ^1^H-NMR spectra, protons of piperazine was seen between 2.32–3.90 ppm. The –CH_3_ group protons of compounds **5a**, **5f** and **5k** on para position of piperazine ring were observed at 2.75–2.78 ppm as asinglet. Again, in position para of the piperazine ring, methyl protons from the -C_2_H_5_ protons of compounds **5b**, **5g** and **5l** were observed in the range of 1.01–1.19 ppm, and -CH_2_ protons in the range of 2.34–2.78 ppm. The aromatic protons of the phenyl (**5d**, **5l** and **5n**), pyridine (**5d**, **5l** ve **5n**) and pyrimidine (**5e**, **5j** and **5o**) substituent attached to the piperazine ring were observed in the range of 6.81–7.69, 6.65–8.14 and 6.67–8.44 ppm, respectively.

The –CH_3_ group protons of compounds **5f**, **5g**, **5h**, **5ı** and **5j** on phenyl ring were observed at 3.82–3.87 ppm ppm as a singlet. The -OC_2_H_5_ group of compounds **5k**, **5l**, **5m**, **5n** ve **5o** on phenyl ring, –OCH_2_ protons were observed at 4.11–4.13 ppm as a quartette while for other compounds (**5k**, **5l**,) –OCH_2_ protons were appeared as multiplet. The protons of CH_3_ group of ethyl substituent were observed 1.35–1.37 ppm as a singlet. Methylene protons between C=O and -S group were recorded as a singlet peak between 4.53–4.65 ppm.

A broad singlet due to NH proton of the benzimidazole ring was recorded at 10.70–13.50 ppm. The aromatic protons of 4-substitutedphenyl assigned at 6.90–8.09 and 8.06–8.38 as two doublets. The benzimidazole protons were visualised in the form of doublet, doublet’s doublet and one singlet in ^1^H NMR spectra at around 7.64–7.92, 7.74–8.03 and 7.89–8.22 due to H-4, H-5, and H-7 protons.

The ^13^C NMR spectra showed the peaks between 165.61–169.44 ppm due to carbonyl group (C=O). The carbons of the piperazine ring were observed at 39.11–55.91 ppm. The ^13^CNMR spectra of all the derivatives showed carbon values in the predictable regions, while the HRMS analysis confirmed the mass with the calculated values of the target compounds. In addition for structure elucidations using routine spectroscopic methods, 2 D NMR studies including HMBC, HSQC and COSY were performed for compound **5e** as seen in Table 1 and supporting information.

### 3.2. Biological activity

#### 3.2.1. Cytotoxicity assay

The benzimidazole-1,3,4-oxadiazole derivatives (**5a**-**5o**) were tested their cytotoxic effects *in vitro* using five cancer cell line A549 (lung carcinoma cell line), HeLa (cervical cell line), MCF-7 (human breast adenocarcinoma cell line), HepG2 (human liver carcinoma cell line) and C6 (rat glioma cell line) cell lines as well as NIH3T3 (mouse embryo fibroblast cell line). Doxorubicin and Hoechst 33342 were used for this study as a reference drugs. The IC_50_ values, defined as the half maximal inhibitory concentration, were summarised in Table 2.

**Table 2.** IC_50_ values (µM) of the compounds **5a**-**5o**, doxorubicin and Hoechst 33342 against A549, MCF-7, C6, HepG2, HeLa and NIH3T3.

Bileşik	A549	MCF-7	C6	HepG2	HeLa	NIH3T3
**5a**	43.256 ± 1.785	**5.704 ± 0.254**	15.614 ± 0.615	**5.695 ± 0.283**	13.960 ± 0.65	39.105 ± 1.940
**5b**	≥100	≥100	≥100	≥100	**0.698 ± 0.032**	944.360**±**7.625
**5c**	≥100	≥100	≥100	21.860**±**0.991	20.285**±**1.101	44.556**±**0.221
**5d**	≥100	**9.148 ± 0.391**	38.93**±**1.921	≥100	≥100	720.645**±**5.032
**5e**	≥100	**7.318 ± 0.352**	≥100	≥100	≥100	54.026**±**2.682
**5f**	≥100	≥100	≥100	≥100	≥100	–
**5g**	≥100	≥100	≥100	≥100	≥100	–
**5h**	≥100	≥100	≥100	≥100	≥100	–
**5ı**	≥100	≥100	≥100	≥100	≥100	–
**5j**	≥100	≥100	≥100	≥100	≥100	–
**5k**	22.254 ± 1.054	38.705**±**1.875	**3.204 ± 0.152**	≥100	11.167**±**0.467	74.630**±**3.631
**5l**	31.017 ± 1.530	≥100	≥100	≥100	**0.224 ± 0.011**	113.451**±**5.472
**5m**	≥100	≥100	32.167**±**1.552	≥100	≥100	52.264**±**2.512
**5n**	**7.388 ± 0.325**	≥100	≥100	≥100	**0.205 ± 0.010**	188.570**±**8.728
**5o**	≥100	**6.442 ± 0.287**	24.951**±**1.237	59.369**±**2.589	≥100	44.072**±**2.104
**Dox.**	12.420**±**0.521	10.525**±**0.472	28.690**±**1.228	16.482**±**0.804	14.280**±**0.704	1110.80**±**8.254
**Hoechst 33342**	0.422**±**0.020	2.404**±**0.118	1.051**±**0.307	27.815**±**1.190	0.306**±**0.015	7.388**±**0.269

Investigations of the cytotoxic activity against A549, MCF-7, C6, HepG-2 and HeLa indicated that the HeLa was the most sensitive cell line to the influence of the new derivatives. It was important to note that compounds **5l** and **5n** exhibited better antiproliferative activity than Hoechst 33342 and doxorubicin against HeLa cell line, with IC_50_ of 0.224 ± 0.011 µM and 0.205 ± 0.010 µM, respectively. The above MTT assay results confirmed our expectation that compounds **5l** and **5n** may exhibit effective activity and lower toxicity and thus possibly better therapeutic potency than Hoechst 33342. Furthermore, compound **5b** exhibited prominent activity against HeLa cell line with IC_50_ 0.698 ± 0.032 µM and shows poor activity against the other cell lines with IC_50_ ≥100 µM

Compound **5a** emerged as potential compound against both cell lines with IC_50_ 5.704 ± 0.254 µM against MCF-7 cell line and IC_50_ 5.695 ± 0.283 µM against HepG2 cell line. IC_50_ values of compounds **5d**, **5e** and **5o** for A549 cell line were determined as 9.148 ± 0.391, 7.318 ± 0.352 and 6.442 ± 0.287 µM, respectively. Only compound **5n** displayed most promising cytotoxic activity against A549 cell line with an IC_50_ value of 7.388 ± 0.325 µM. Besides, among compounds **5a**-**5o**, compound **5k** exhibited the most promising anticancer activity against C6 cell line with IC_50_ values of 3.204 ± 0.152 µM.

It is crucial that an anticancer agent affects the cancer cell line but having minimal or no side-effect on healthy cells. For this purpose, the cytotoxic effects of the active compounds on the NIH3T3 cell line were investigated (Table 2).

According to all results, it can be concluded that 4-hydroxyphenyl and 4-ethoxyphenyl enhanced anticancer activity more than 4-methoxyphenyl. The presence of methyl and ethyl group at the 4th position of the piperazine scaffold also increased anticancer activity, whereas phenyl moiety at the 4th position of the piperazine ring led to a significant drop in anticancer activity. However, compounds carrying pyridine and pyrimidine substituents have also been found to be active.

#### 3.2.2. DNA synthesis inhibition assay

According to the MTT assay, compound **5n** for A549 cell line; compounds **5a**, **5d**, **5e**, **5o** for MCF-7 cell line; compound **5k** for C6 cell line; compound **5a** for HepG2 cell line; compounds **5b**, **5l** and **5n** for HeLa cell line were selected for the DNA synthesis inhibition assay. A549, C6, MCF-7, HepG2 and HeLa cells were incubated with three different concentrations (IC_50/2_, IC_50_, and IC_50_/2) of the compounds for 24 and 48 h time periods. The tested compounds showed time-and dose-dependent inhibitory activity on DNA synthesis ofthe tumour cells. Doxorubicin was used as positive control.

**Figure 2.**
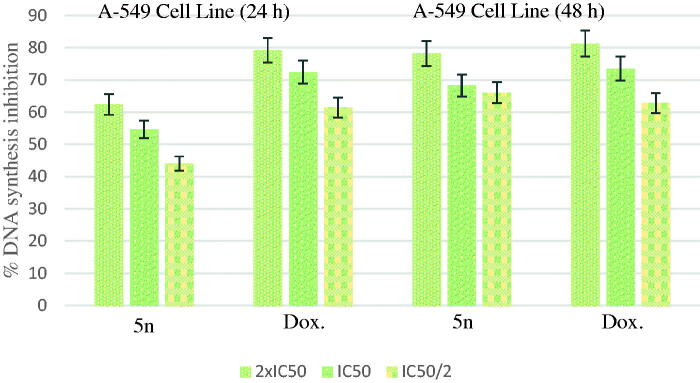
% DNA synthesis inhibition activities of compound **5n** and doxorubicin against A549 cell line.

Figure 2 shows the DNA % synthesis inhibitory activity of the compound **5n** and standard drug on A549 cells. Compound **5n** was found to have 78.17, 68.25 and 66.00% DNA synthesis inhibition at 14.77, 7.39 and 5.39 µg/mL doses after 48 h of incubation, whereas doxorubicin was found to have 81.22, 73.43 and 62.78% inhibition at 24.84, 12.42 and 6.21 µg/mL doses, respectively.

**Figure 3.**
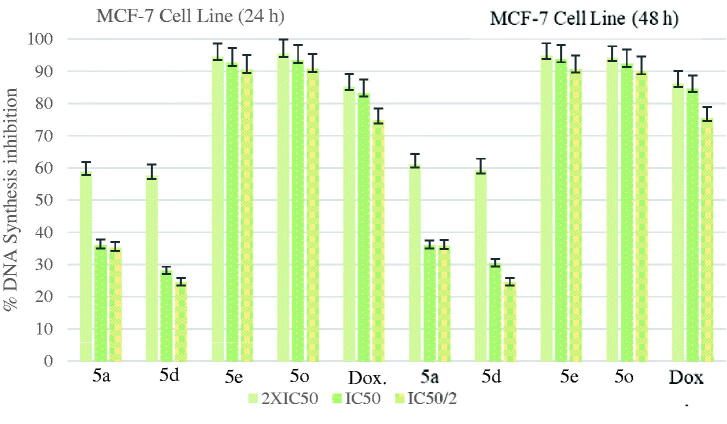
% DNA synthesis inhibition activities of compounds **5a**, **5d**, **5e**, **5o** and doxorubicin against MCF-7 cell line.

The DNA % synthesis inhibitory activity of the compounds **5a**, **5d**, **5e**, **5o**, and standard drug on MCF7 cells is seen in Figure 3. Among of the compounds, compound **5e** was determined as the most active compounds. Compound **5e** was observed to have an approximate inhibitory activity with a value of 93.81% at 7.318 µg/mL concentration whereas doxorubicin had 84.60% inhibition at 10.525 µg/mL concentration after 48-h incubation.

**Figure 4.**
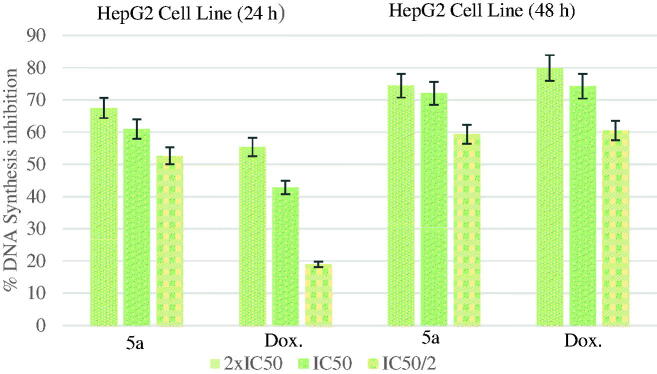
% DNA synthesis inhibition activities of compound **5a** and doxorubicin against HepG2 cell line.

Figure 4 shows the DNA % synthesis inhibitory activity of the compound **5a** and standard drug on HepG2 cells. Compound **5a** had 60.89 and 72.05% inhibitory activity after 24- and 48-h incubation, at 5.695 µg/mL whereas doxorubicin had 42.80 and 74.22% inhibitions at 16.482 µg/mL.

**Figure 5.**
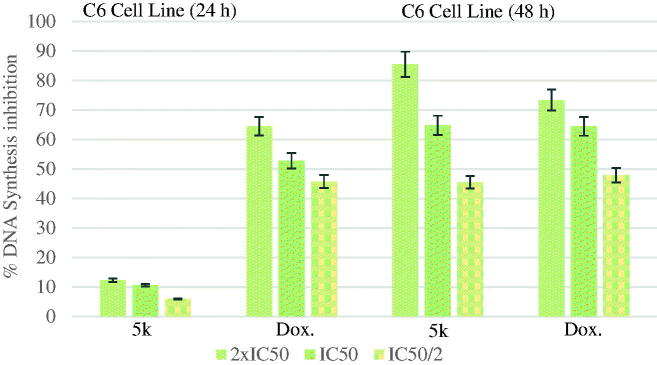
% DNA synthesis inhibition activities of compound **5k** and doxorubicin against C6 cell line.

The DNA % synthesis inhibitory activity of the compound **5k** and standard drug on C6 cells is seen Figure 5. DNA % inhibition was increased with the increasing incubation period (24 and 48 h) for the compound **5k** and doxorubicin. This increase can be clearly appreciated in particular for the compound **5k**. Measurement of the inhibition for the compound **5k** at lower dose (IC_50_/2) after 48 h incubation was eight times higher than at 24 h of measurement.

**Figure 6.**
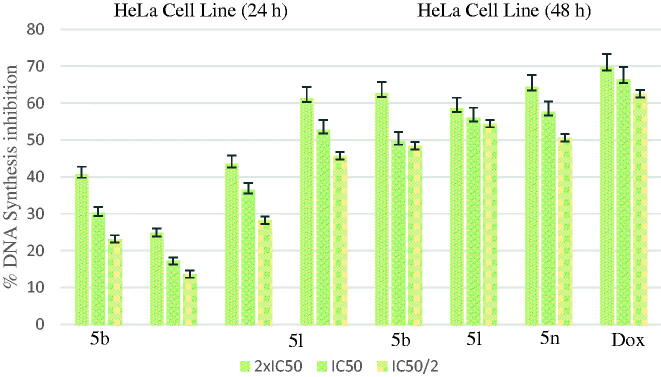
% DNA synthesis inhibition activities of compounds 5 b, 5 l, 5n and doxorubicin against HeLa cell line.

Figure 6 shows the DNA % synthesis inhibitory activity of the compounds **5b**, **5l**, **5n** and standard drug on HeLa cells. DNA % inhibition was increased with the increasing incubation period (24 and 48 h) for all of the compounds. Compounds **5b**, **5l**, **5n** were found to have 49.70, 56.01 and 57.65% DNA synthesis inhibition at 0.698, 0.224 and 0.205 µg/mL doses after 48 h of incubation whereas doxorubicin was found to have 66.48% inhibition at 14.280 µg/mL doses, respectively.

#### 3.2.3. Flow cytometric analysis

**Figure 7.**
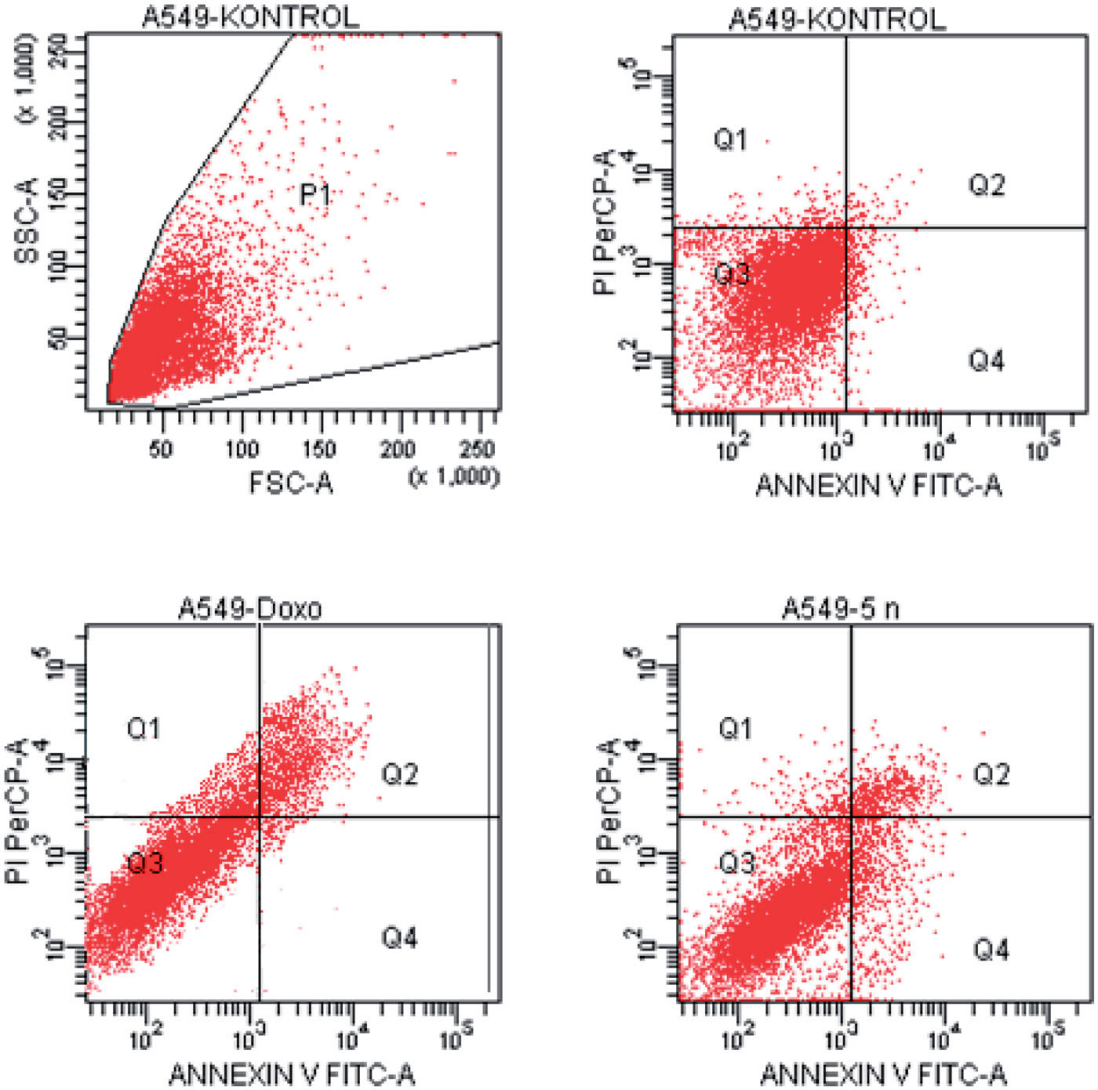
The flow cytometric analysis diagram of compound **5n** and doxorubicin for A549 cell line.

**Figure 8.**
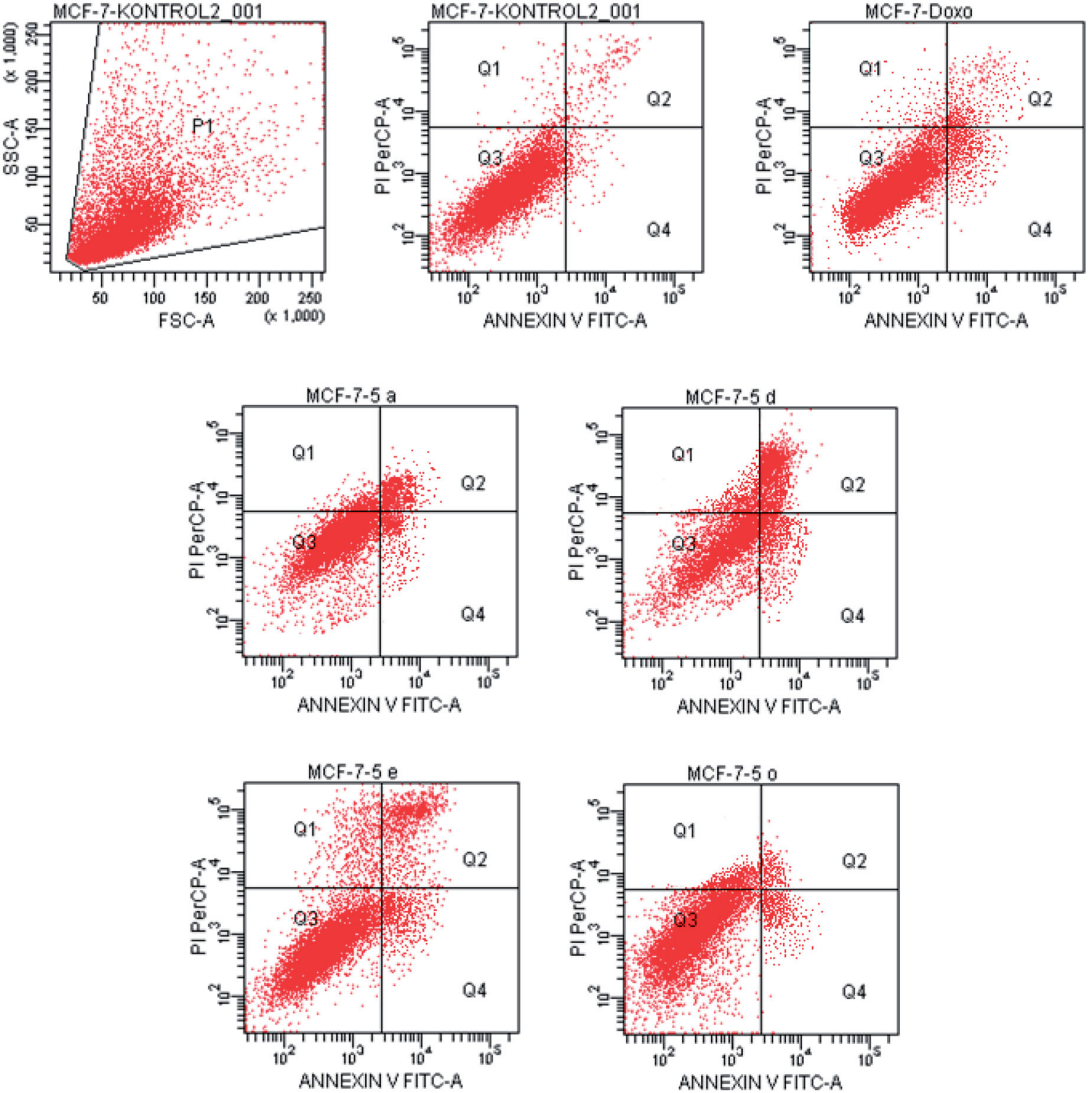
The flow cytometric analysis diagram of compounds **5d, 5e, 5o** and doxorubicin for MCF-7 cell line.

**Figure 9.**
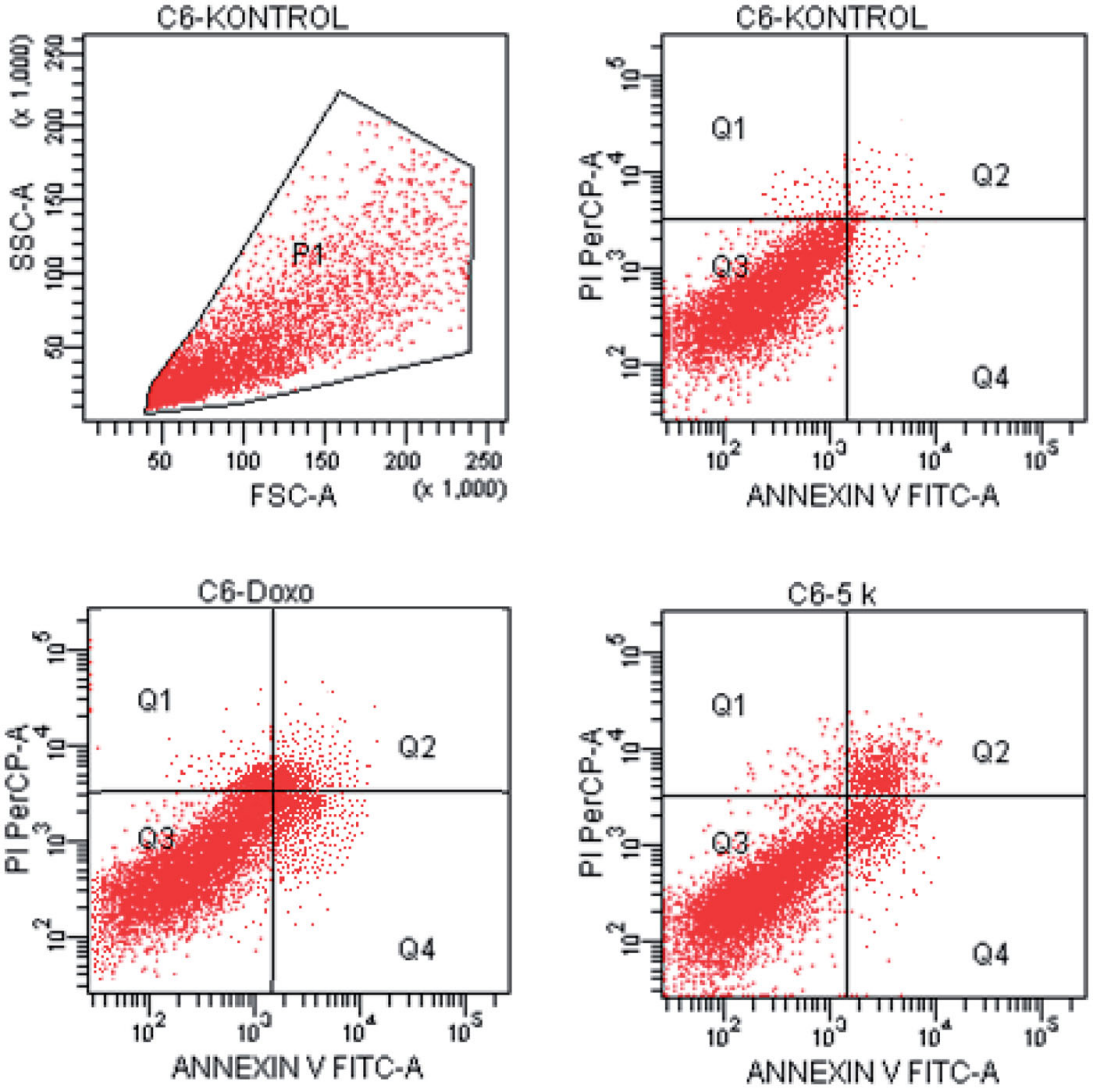
The flow cytometric analysis diagram of compound **5k** and doxorubicin for C6 cell line.

**Figure 10.**
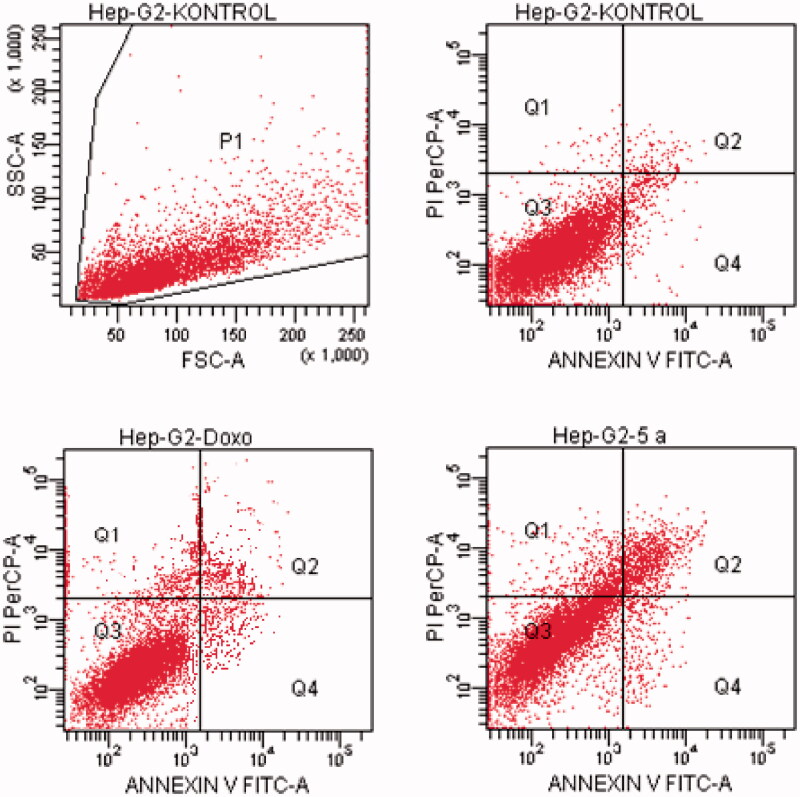
The flow cytometric analysis diagram of compound **5a** and doxorubicin for HepG2 cell line.

**Figure 11.**
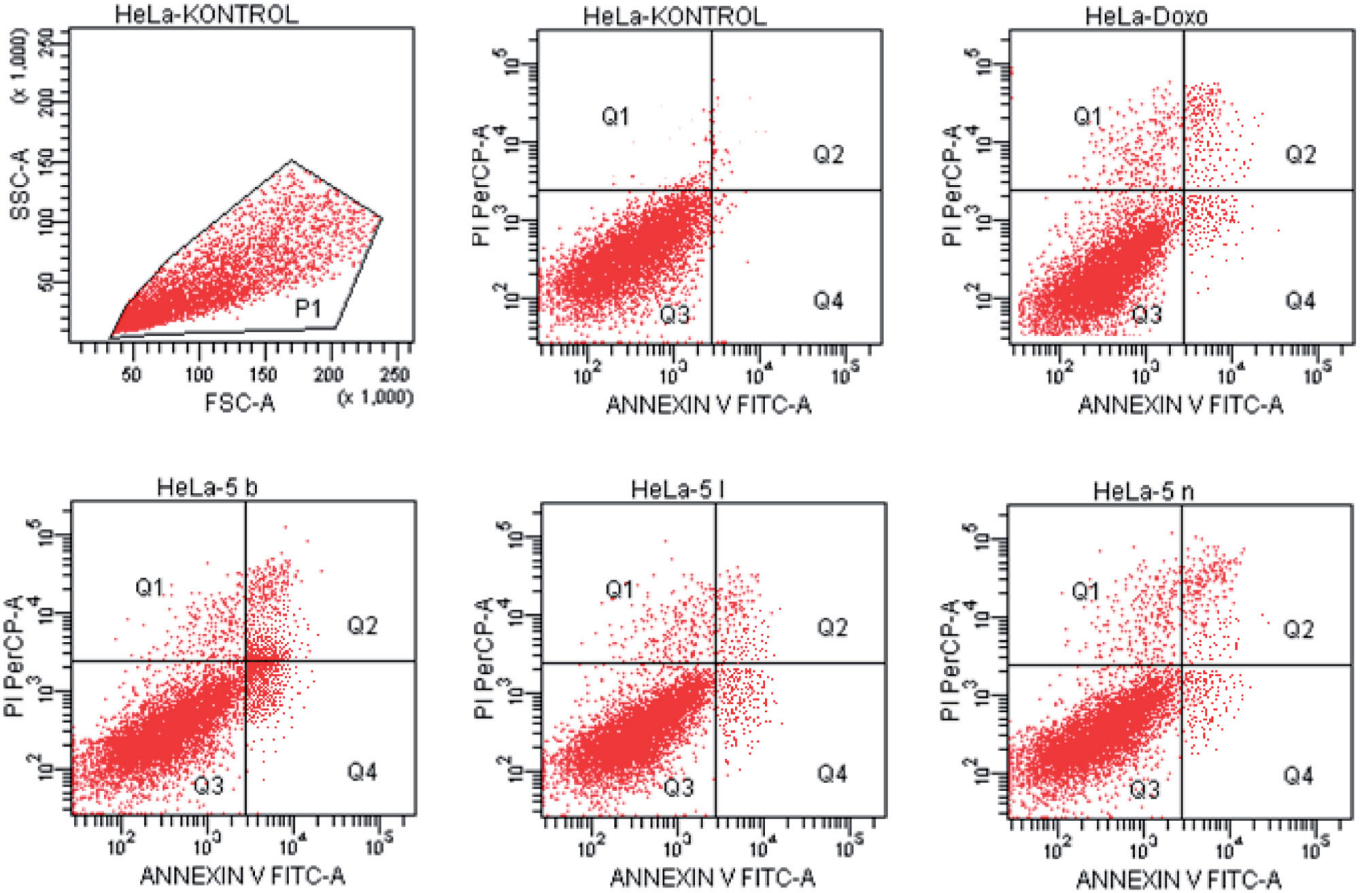
The flow cytometric analysis diagram of compounds **5 b, 5 l, 5n** and doxorubicin for HeLa cell line.

Annexin V/PI staining on tested cancer cells treated with the active compounds was performed to investigate induction of apoptosis. Usually, four quadrant images were generated by flow cytometry: Q1 shows damaged cells due to any technical factor rather than compound, Q2 area shows late apoptotic cells, Q3 denotes normal cells while Q4 illustrates early apoptotic cells. According to the MTT assay, compound **5n** for A549 cell line; compounds **5a**, **5d**, **5e**, **5o** for MCF-7 cell line; compound **5k** for C6 cell line; compound **5a** for HepG2 cell line; compounds **5b**, **5l** and **5n** for HeLa cell line were selected for the flow cytometric analysis. Furthermore, flow cytometry studies were performed for reference drug doxorubicin. Flow cytometric analysis diagrams of tested compounds and doxorubicin at IC_50/2_, IC_50_, and 2xIC_50_ concentrations on cancer cells were presented in Figures 7–11.

Flow cytometric analysis diagram of doxorubicin with compound **5n** was presented in Figure 7 for A549 cell line. Accordingly, the percentage of apoptotic cells at IC_50_ concentrations was calculated as 19.2% for doxorubicin and 13% for compound 5n.

Flow cytometric analysis diagram of doxorubicin with compounds **5a**, **5d**, **5e** and **5o** in MCF-7 cell line was presented in Figure 8. Compound **5d** caused the highest percentage of apoptosis (early + late apoptotic cells) with 22.3% whereas doxorubicin caused 12.7% on MCF-7 cells. The other compounds **5a**, **5e** and **5o** possessed 15.4, 13.1 and 8.7% apoptotic cell percentages.

Flow cytometric analysis diagram of doxorubicin with compound **5k** was presented in Figure 9 for C6 cell line. Accordingly, the percentage of apoptotic cells at IC_50_ concentrations was calculated as 13.7% for doxorubicin and 12.3% for compound **5k**.

For HepG2 cell line, the percentage of apoptotic cells at IC_50_ concentrations was calculated as 9.4% for doxorubicin and 13.2% for compound **5a** (Figure 10). As a result, it is observed that compound **5a** induces apoptotic cell death more than doxorubicin in HepG2 cell line in IC_50_ concentrations and 24 h incubation time.

Flow cytometric analysis diagram of doxorubicin and compounds **5b**, **5l**, **5n** on HeLa cell line was presented in Figure 11. For HeLa cells, compound **5b** showed the highest percentage of 17.4% whereas doxorubicin caused 17.4% apoptotic cells. The other tested compounds **5l** and **5n** followed it with 8.6 and 15.2% percentages.

#### 3.2.4. DNA topoisomerase I assay

To determine whether the DNA intercalation properties of compounds **5a**, **5b**, **5d**, **5e**, **5k**, **5l**, **5n** and **5o** might result in Topo I inhibition, agarose gel electrophores is assay was performed to evaluate the Topo I inhibitory activity of them via agarose-gel electrophoresis and CPT was used as the positive control. The TopoGEN Topoisomerase I Drug Screening Kit were optimised and implemented to accomplish this goal.

The topo I DNA relaxation data demonstrated that the supercoiled kDNA migrates faster on the agarose gel than the relaxed topoisomers (nicked) caused by the full activity of topo I. Therefore, the appearance of relaxed topoisomers along with residual supercoiled form indicates the partial activity of topoisomerase I and thus its inhibition by the test compounds. In a previous studies, it was said that the compounds poisoning topoisomerase caused the formation of nick bands in such gel appearances, while the formation of Supercoiled bands was said to be caused by catalytic inhibitors that bound to either DNA or enzyme^40^^,^^41^.

**Figure 12.**
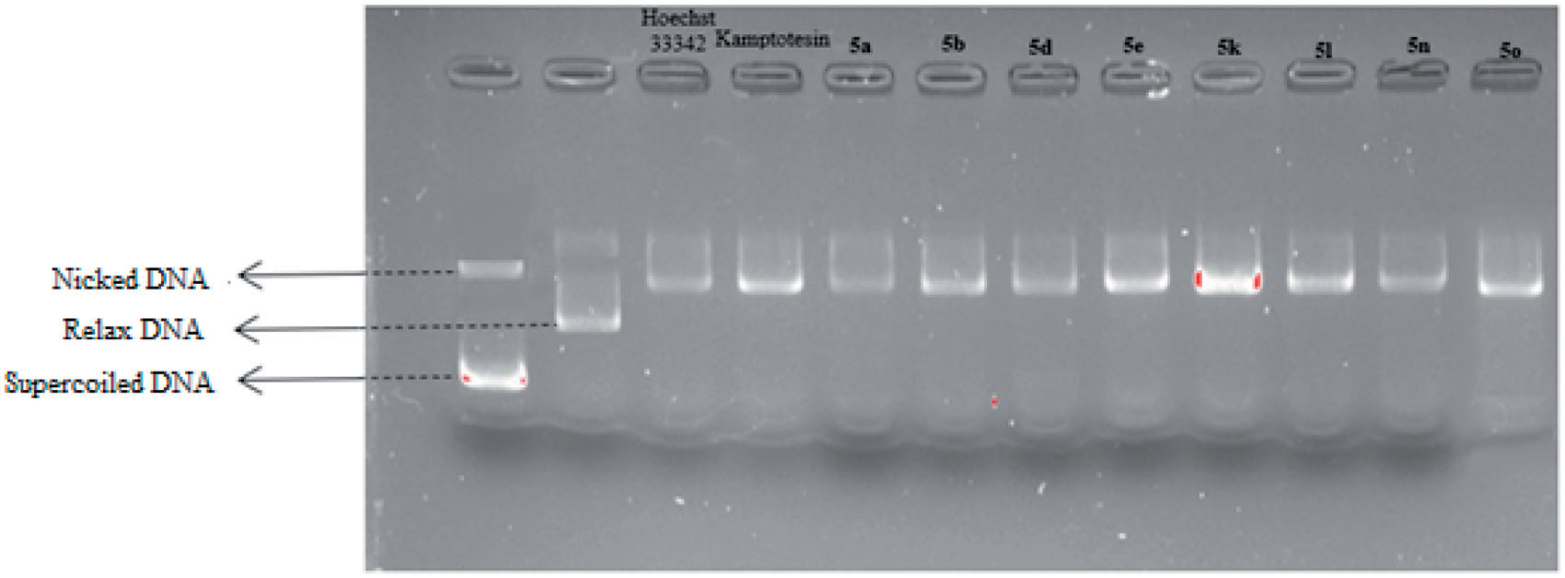
Topo I activity of compounds **5a**, **5 b**, **5d**, **5e**, **5k**, **5 l**, **5n**, **5o**, Hoechst 33342 and camptothecin.

The results in Figure 12 indicated that compounds **5a**, **5b**, **5d**, **5e**, **5k**, **5l**, **5n** and **5o** exhibited potent topo I inhibition activity. By the comparison of the effective inhibited concentrations of camptothecin with these compounds, it could be concluded that these compounds exhibited similar topo I inhibition activity with camptothecin.

### 3.3. Molecular docking

In order to determine the possible interactions of compound, showed high activity, docking studies have been performed by using high-resolution crystal structure of the DNA-Topoisomerase I enzyme complex (PDB Code: 1T8I)^35^^,^^42–45^. Hoechst 33342 molecule was included in the docking study with the relevant crystal structure before performing the analysis of binding modes of selected compound which has displayed significant topoisomerase I enzyme activity (**5n**). Hence, the docking protocol was verified by examining and comparing binding modes and interactions of Hoechst 33342.

**Figure 13.**
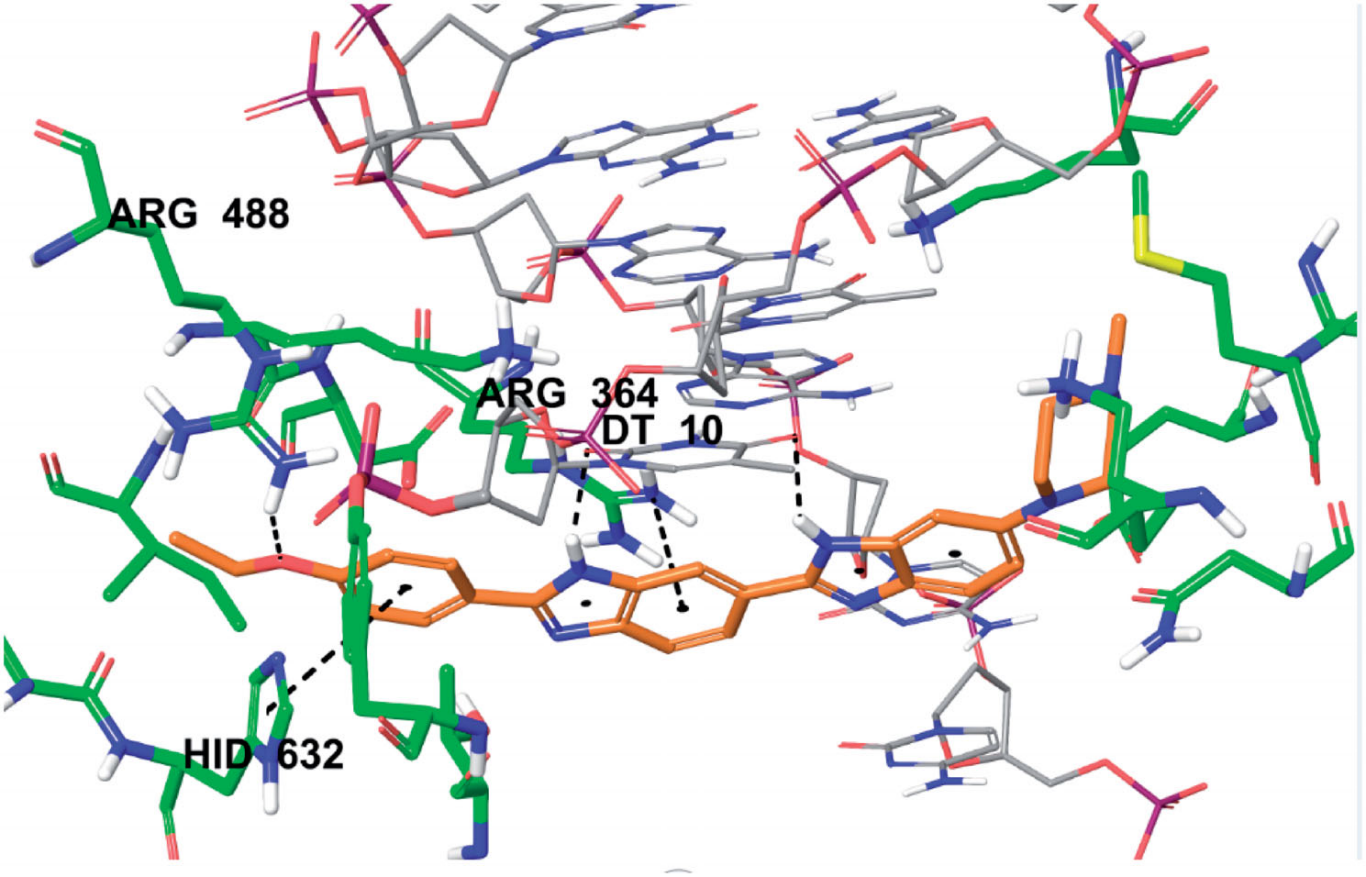
Three-dimensional interaction of Hoechst 33342 with the DNA-Topoisomerase I enzyme complex active site.

Two-dimensional interaction pose of Hoechst 33342 with DNA-Topoisomerase I enzyme complex active region is given in Figure 13. The oxygen atom in the ethoxy group in the structure forms a hydrogen bond with amino Arg488. The phenyl ring near to ethoxy group establishes π-π interaction with imidazole ring of Hid632. The benzimidazole ring adjacent to phenyl ring has been found to be very important for both polar and apolar interactions. Benzene and imidazole structures of benzimidazole ring forms cation-π interactions separately with amino of Arg364. Each of the nitrogen atoms located in the same position of successive benzimidazole rings in the structure of Hoechst 33342 creates a hydrogen bond with carbonyl of thymine (DT10) in the D chain of DNA-Topoisomerase enzyme complex. In addition, these interactions analyse of Hoechst 33342 were found to be similar and compatible with the literature data^3^.

**Figure 14.**
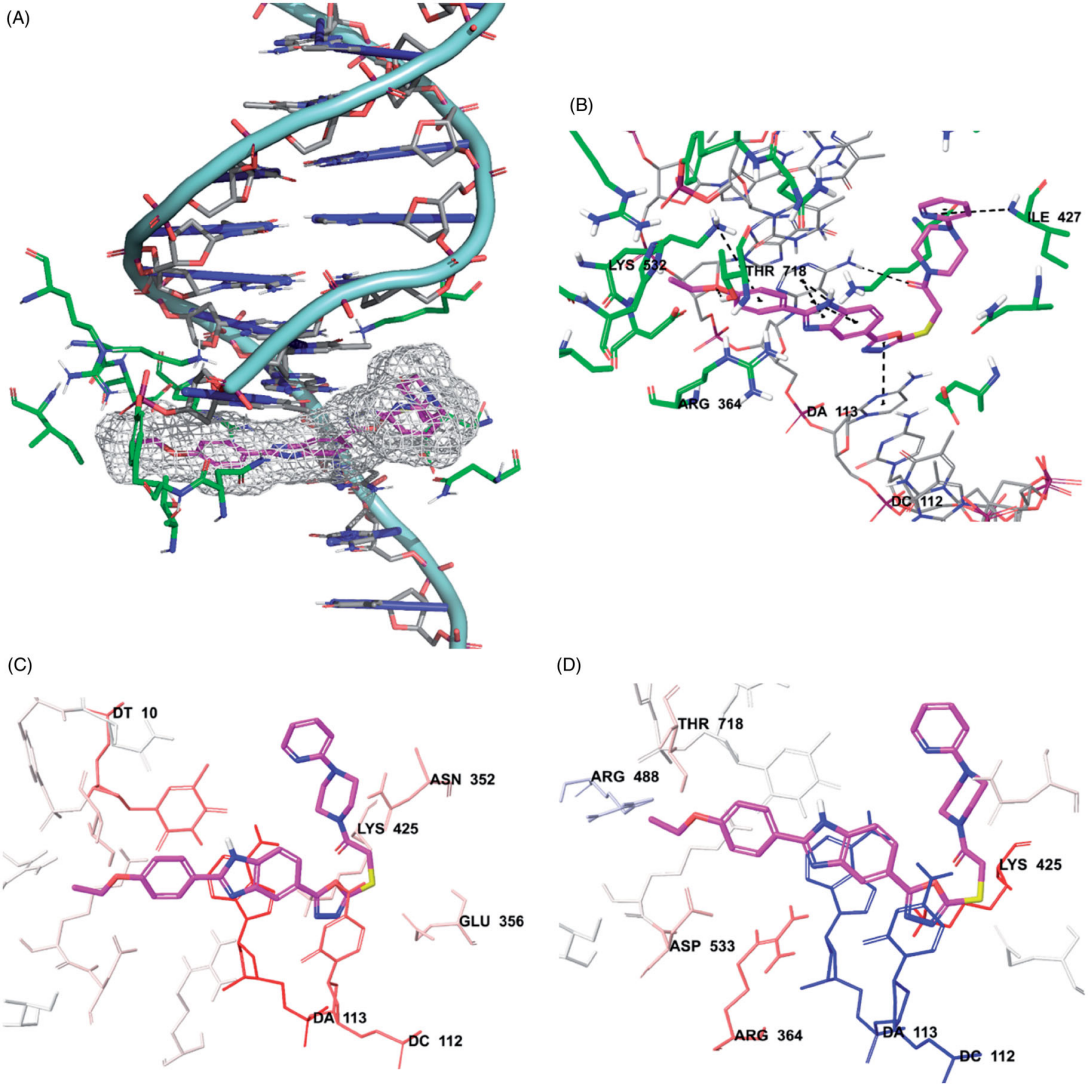
Docking poses of compound **5n**. (**A)** Placement of the compound **5n** on the DNA-Topoisomerase I enzyme complex active site. (**B)** The interacting mode of compound **5n** in the active region of DNA-Topoisomerase I enzyme. (**C)** Van der Waals interaction of DNA-Topoisomerase I enzyme complex active site of compound **5n**. (D) Electrostatic interaction of DNA-Topoisomerase I enzyme complex active site of compound **5n**.

The docking poses of compound **5n** was given in Figure 14. When the docking poses of this compound was examined, it was seen that this compound interact with both amino acids in the enzyme active region and certain regions of the DNA contained in the complex crystal.

Scheme 1.Synthesis way of the compounds **5a-5o.**

**Figure:**
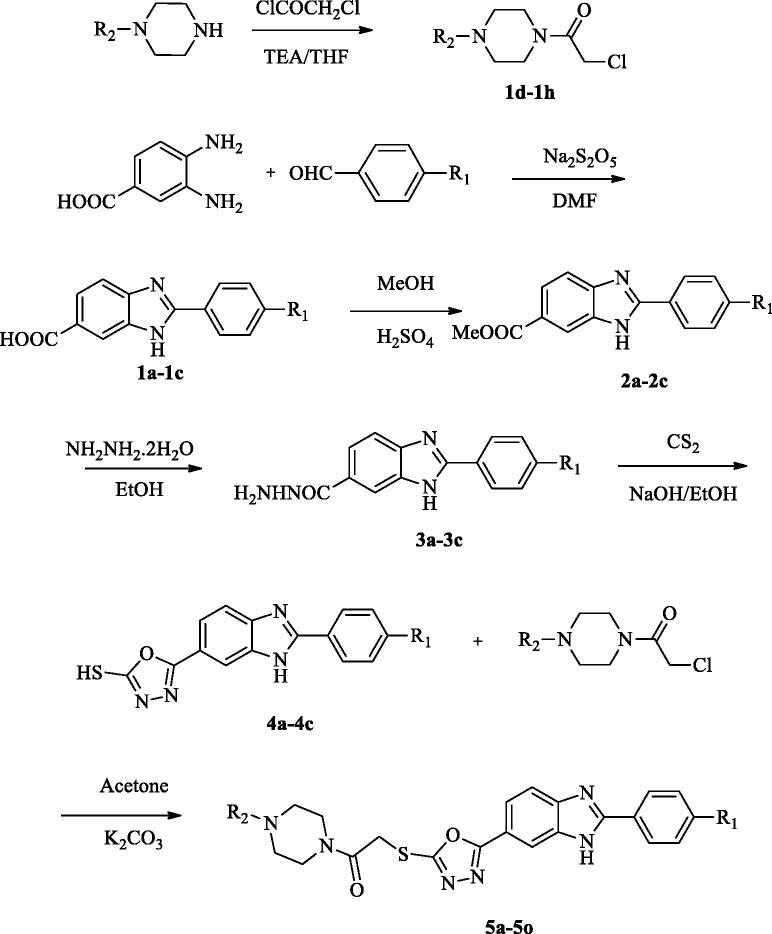

Thus, it was observed that it cause damage in DNA chain and prevent the binding of topoisomerase enzyme to DNA (Figure 14(A)). Also, when looking at the chemical structure of the compound, it has been seen that benzimidazole, oxadiazole and pyridine structures show similar interactions. Benzimidazole ring system has attracted attention in terms of polar and apolar interactions. π–π interactions were observed separately between purine ring of adenine (DA113) in the DNA chain and benzene and imidazole rings of benzimidazole in the compound **5n**. Also, in this compound, phenyl ring of benzimidazole forms π–π interaction with Arg364. The nitrogen atom in the benzimidazole ring is found to be important for polar interactions.

The oxadiazole ring in the structure of compound **5n** has been shown to contribute to binding to DNA-Topoisomerase I enzyme complex active site (Figure 14(B)). The oxadiazole ring forms π–π interaction with pyrimidine of cytosine (DC112) in DNA. It has been determined that carbonyl group in the chemical structures of selected compound is especially important in binding to the DNA chain (Figure 14(C)). In compound **5n**, carbonyl group forms a hydrogen bond with amino group in the purine ring of adenine (DA113) of DNA. The oxygen atom of ethoxy group in the para position for compound 5n forms a hydrogen bond with hydroxyl of Thr718 in both compounds. An additional cation–π interaction is observed between amino group of Lys532 and para-ethoxypheny. It is observed that the chemical structure of selected compound whose molecular docking studies are carried out include pyridine ring adjacent to the piperazine ring. It has been observed that nitrogen atoms in this ring systems are very effective and important in binding to related region. As it is known, nitrogen atom in these ring systems have the ability to make hydrogen bonds by acting as an electron donor by keeping the unpaired electron pairs on them outside the ring without including them in the ring electron system. They strengthen the binding profiles of the compound by forming hydrogen bonds with the active region thanks to their properties. Hydrogen bond formation with amino group of Ile427 is observed in the compound **5n**. Van der Waals and electrostatic interactions of selected compound with DNA-Topoisomerase I enzyme complex active region is given in Figure 14(D). According to the docking analysis procedure followed in Van der Waals interactions, red and pink coloured amino acids and nucleic acids indicate strong van der Waals interactions. Hereunder, it is seen that Asn352, Glu356, Lys425, Lys532, Asp533 and Ile535 amino acids and DT10, DC112 and DA113 nucleic acids in DNA play an important role in terms of van der Waals interactions. Strong electrostatic interactions are similarly represented by red- and blue-coloured amino acids and nucleic acids according to the docking analysis procedure. In terms of electrostatic interactions, it is seen that Glu356, Arg364, Lys425, Arg488, Lys532, Asp533 and Thr718 amino acids and DT10, DC112 and DA113 nucleic acids in DNA play an important role.

Table 3 presents the binding affinity and interacting residues of compound **5n** and Hoechst. The binding affinity was calculated according to the parameters of docking score, Glide gscore and Glide emodel, and is as follow: for compound **5n** −7.409, −7.889, −100.082 kcal/mol and for Hoechst −4.373, −5.989, −66.563 kcal/mol. As can be seen from this table, compound **5n** showed more potent binding affinity than Hoechst.

**Table 3.** Binding affinity and interacting residues of compound **5n** and Hoechst.

Compounds	Binding affinity (Kcal/mol)			Interacting residues (According to Van der Waals, coulomb, hydrogen-bonding energies)
	Docking score	Glide gscore	Glide emodel	
**5n**	−7.409	−7.889	−100.082	Asn352, Glu356, Arg364, Lys374, Lys425, Arg488, Lys493, Gly531, Lys532, Asp533, Ile535, Lys587, Arg590, Hid632, Gly717, Thr718, Asn722, DA7, DC8, DT9, DT10, DC111, DC112, DA113, DA114, DG115, DT116
**Hoechst**	−4.373	−5.989	−66.563	Asn352, Glu356, Phe361, Arg364, Lys374, Gln421, Ser423, Lys425, Arg488, Lys493, Lys532, Asp533, Lys587, Arg590, Hid632, Gly717, Thr718, Leu721, Asn722, DA7, DC8, DT9, DT10, DC111, DC112, DA113, DA114, DG115, DT116

## 4. Conclusion

In conclusion, 15 novel benzimidazole-1,3,4-oxadiazole derivatives were synthesised and evaluated for their anticancer effects on A549, MCF-7, C6, HepG2 and HeLa cell lines. The results of this preliminary cytotoxicity screening using the MTT assay suggested that many of them (**5a**, **5b**, **5d**, **5e**, **5k**, **5l**, **5n** and **5o**) displayed potent antitumor activity in different cancer cell lines. Especially, compounds **5l** (0.224 µM) and **5n** (0.205 µM) displayed potent and selective anticancer activity against HeLa cell line compared to doxorubicin (14.280 µM) and Hoechst 33342 (0.306 µM). Further detailed biological studies including flow cytometric analysis, DNA synthesis inhibition assay and Topo I inhibition studies delivered promising results. Docking studies of compound **5n** and Hoechst 33342 were performed and probable interactions in the DNA-Topo I enzyme complex was determined. According to all *in vitro* and in silico studies, novel benzimidazole-1,3,4-oxadiazole derivatives could be served as a novel promising scaffold for the development of new chemotherapeutic agent.

## Supplementary Material

Supplemental Material
