# Supplementary material for: Synthesis, anticancer evaluation and molecular docking studies of new benzimidazole- 1,3,4-oxadiazole derivatives as human topoisomerase types I poison
Source: J Enzyme Inhib Med Chem. 2020 Aug 19;35(1):1657–73. doi: 10.1080/14756366.2020.1806831 (PMC7470102; doi:10.1080/14756366.2020.1806831)
Supplement: Supplemental Material [file IENZ_A_1806831_SM5293.pdf]

# Synthesis, anticancer evaluation and molecular docking studies of new benzimidazole-1,3,4-oxadiazole derivatives as human topoisomerase types I poison

**Ulviye Acar Çevik[a,b*], Begüm Nurpelin Sağlık[a,b], Derya Osmaniye[a,b], Serkan Levent[a,b], Betül Kaya Çavuşoğlu[c], Abdullah Burak Karaduman[d], Özlem Atlı Eklioğlu[d], Yusuf Özkay[a,b*], Zafer Asım Kaplancıklı[a]**

[a] *Department of Pharmaceutical Chemistry, Faculty of Pharmacy, Anadolu University, 26470 Eskişehir, Turkey*

[b] *Doping and Narcotic Compounds Analysis Laboratory, Faculty of Pharmacy, Anadolu University, 26470 Eskişehir, Turkey*

[c] *Department of Pharmaceutical Chemistry, Faculty of Pharmacy, Zonguldak Bülent Ecevit University, 67600 Zonguldak, Turkey*

[d] *Department of Pharmaceutical Toxicology, Faculty of Pharmacy, Anadolu University, 26470 Eskişehir, Turkey*

* Corresponding author.

*E-mail address*: uacar@anadolu.edu.tr

*Tel*: +90-222-3350580/3774 *Fax*: +90-222-3350750.

*Address*: Anadolu University, Faculty of Pharmacy, Department of Pharmaceutical Chemistry, 26470, Eskişehir, Turkey.

DOPNALAB

| | Item | Value |
|---|---|---|
| | Acquired Date&Time | 5.02.2019 14:11:07 |
| | Acquired by | System Administrator |
| | Filename | C:\Users\dopnalab\Desktop\derya\OP\op-oh-ester1.ispd |
| | Spectrum name | op-oh-ester1 |
| | Sample name | oh-ester |
| | Sample ID | |
| | Option | |
| | Comment | |
| | No. of Scans | 10 |
| | Resolution | 4 [cm-1] |
| | Apodization | Happ-Genzel |

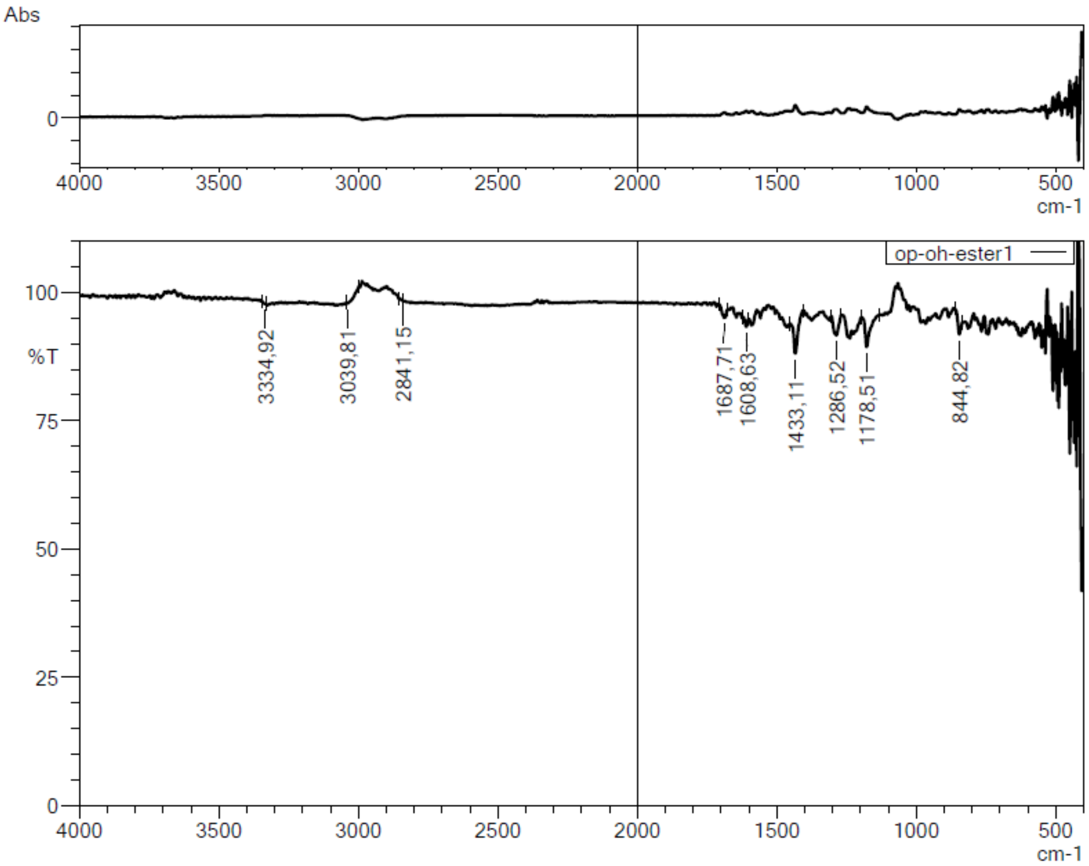


**Figure 1.** IR spectrum of compound **2a**

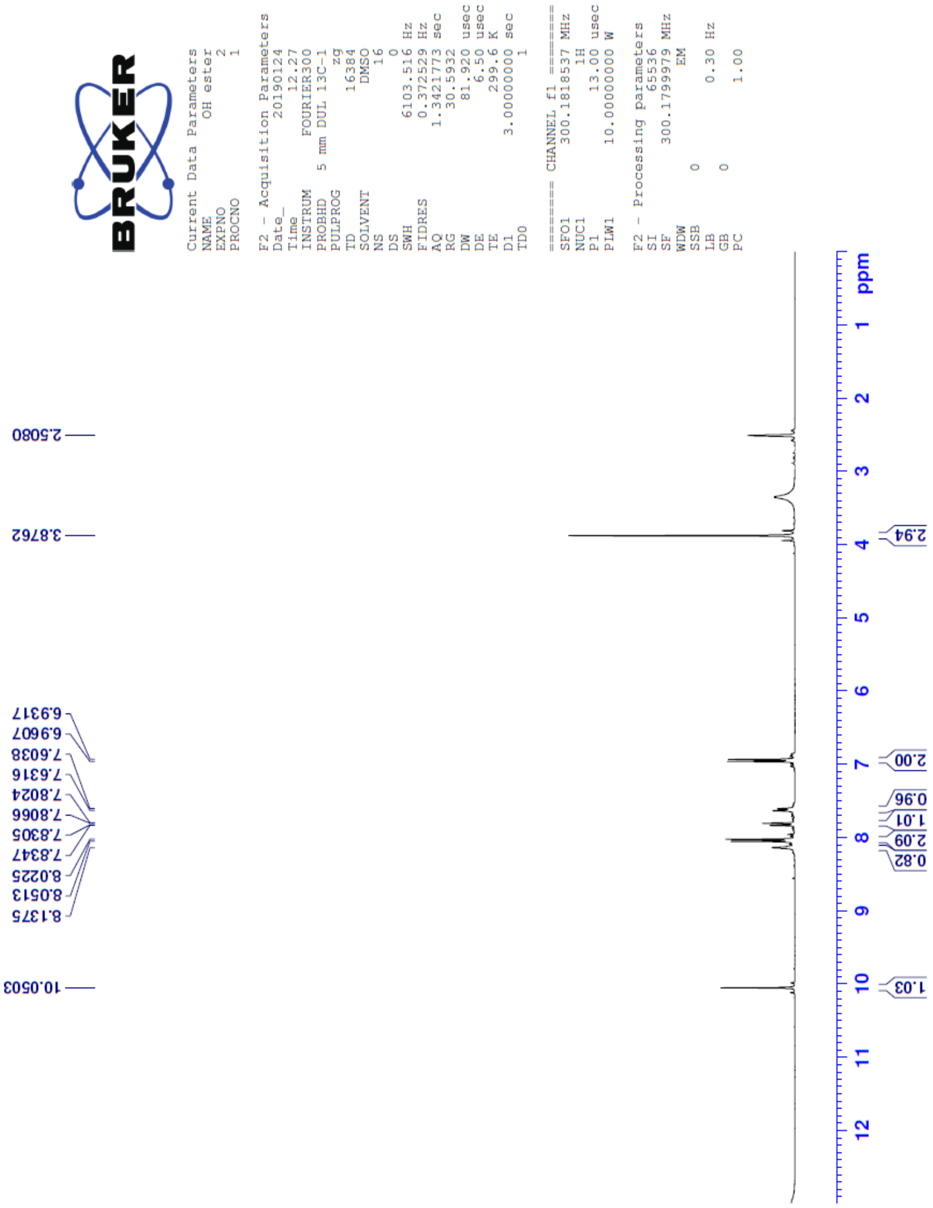

**Figure 2.** $^{1}$H-NMR spectra of compound **2a**

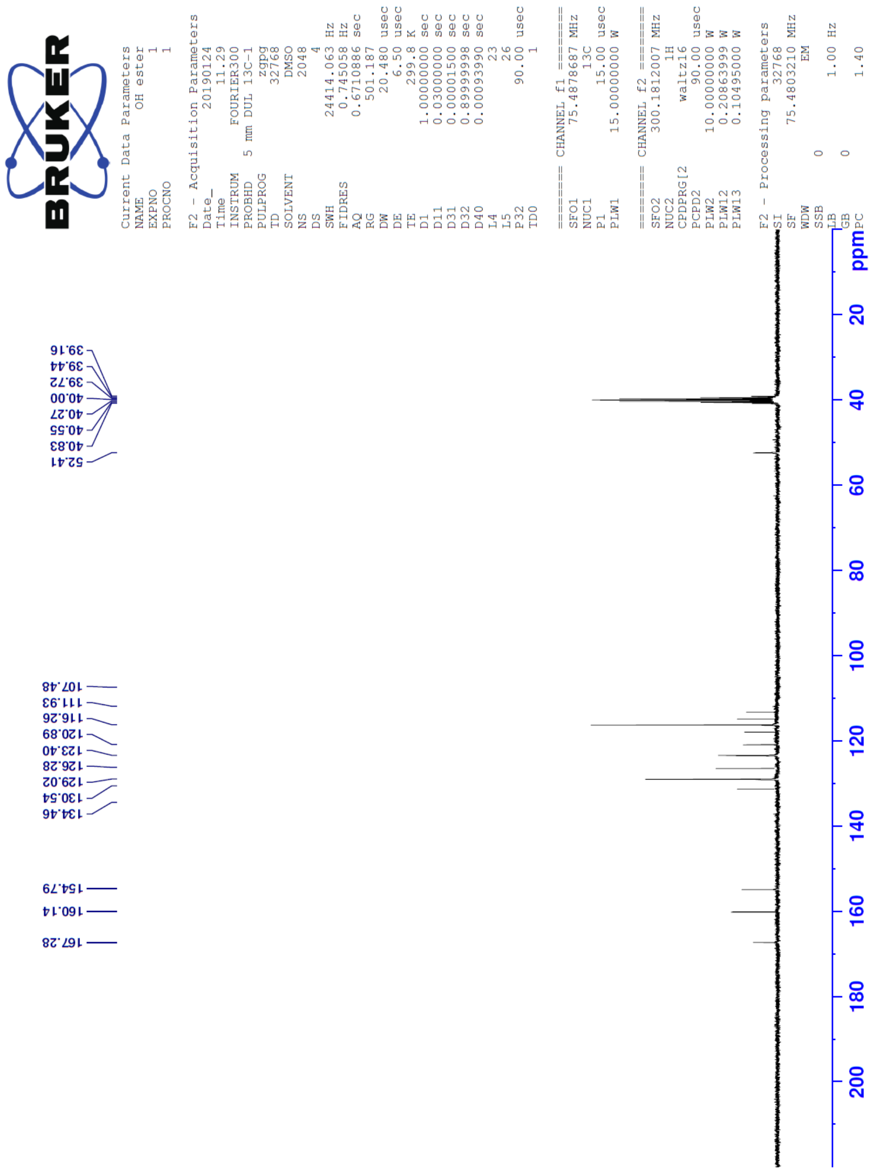


**Figure 3.** $^{13}$C-NMR spectra of compound **2a**

Formula Predictor Report - OH-E_3.lcd Page 1 of 1

Data File: C:\LabSolutions\Data\Analiz\uac\OH-E_3.lcd

| Elmt | Val. | Min | Max | Elmt | Val. | Min | Max | Elmt | Val. | Min | Max | Elmt | Val. | Min | Max | Use Adduct |
|---|---|---|---|---|---|---|---|---|---|---|---|---|---|---|---|---|
| H | 1 | 10 | 40 | O | 2 | 3 | 5 | S | 2 | 0 | 2 | Ru | 2 | 0 | 0 | H |
| C | 4 | 15 | 30 | F | 1 | 0 | 0 | Cl | 1 | 0 | 0 | Pd | 2 | 0 | 0 | |
| N | 3 | 0 | 6 | P | 3 | 0 | 0 | Br | 1 | 0 | 0 | I | 3 | 0 | 0 | |

Error Margin (ppm): 15
HC Ratio: unlimited
Max Isotopes: 3
MSn Iso RI (%): 10.00

DBE Range: 5.0 - 22.0
Apply N Rule: yes
Isotope RI (%): 1.00
MSn Logic Mode: AND

Electron Ions: both
Use MSn Info: yes
Isotope Res: 9000
Max Results: 500

Event#: 1 MS(E+) Ret. Time : 5.213 Scan# : 783

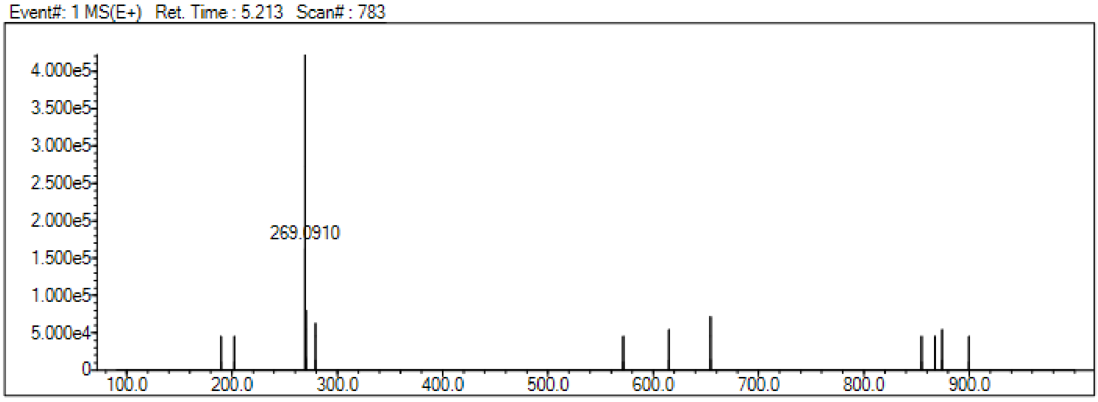


Measured region for 269.0910 m/z

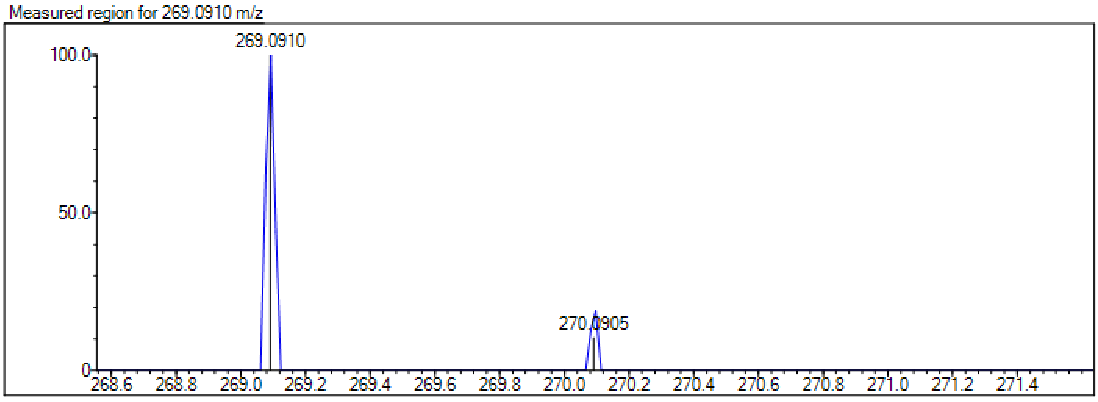


C15 H12 N2 O3 [M+H]+ : Predicted region for 269.0921 m/z

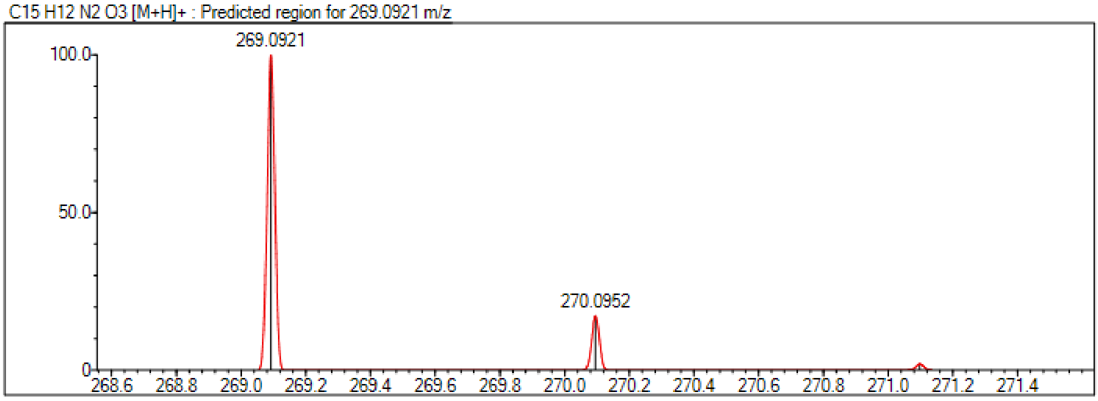


| Rank | Score | Formula (M) | Ion | Meas. m/z | Pred. m/z | Df. (mDa) | Df. (ppm) | Iso | DBE |
|---|---|---|---|---|---|---|---|---|---|
| 1 | 76.27 | C15 H12 N2 O3 | [M+H]+ | 269.0910 | 269.0921 | -1.1 | -4.09 | 82.66 | 11.0 |

**Figure 4.** Mass spectrum of compound **2a**

DOPNALAB

| | Item | Value |
|---|---|---|
| | Acquired Date&Time | 5.02.2019 14:13:17 |
| | Acquired by | System Administrator |
| | Filename | C:\Users\dopnalab\Desktop\derya\OP\op-ome-ester1.ispd |
| | Spectrum name | op-ome-ester1 |
| | Sample name | ome-ester |
| | Sample ID | |
| | Option | |
| | Comment | |
| | No. of Scans | 10 |
| | Resolution | 4 [cm-1] |
| | Apodization | Happ-Genzel |

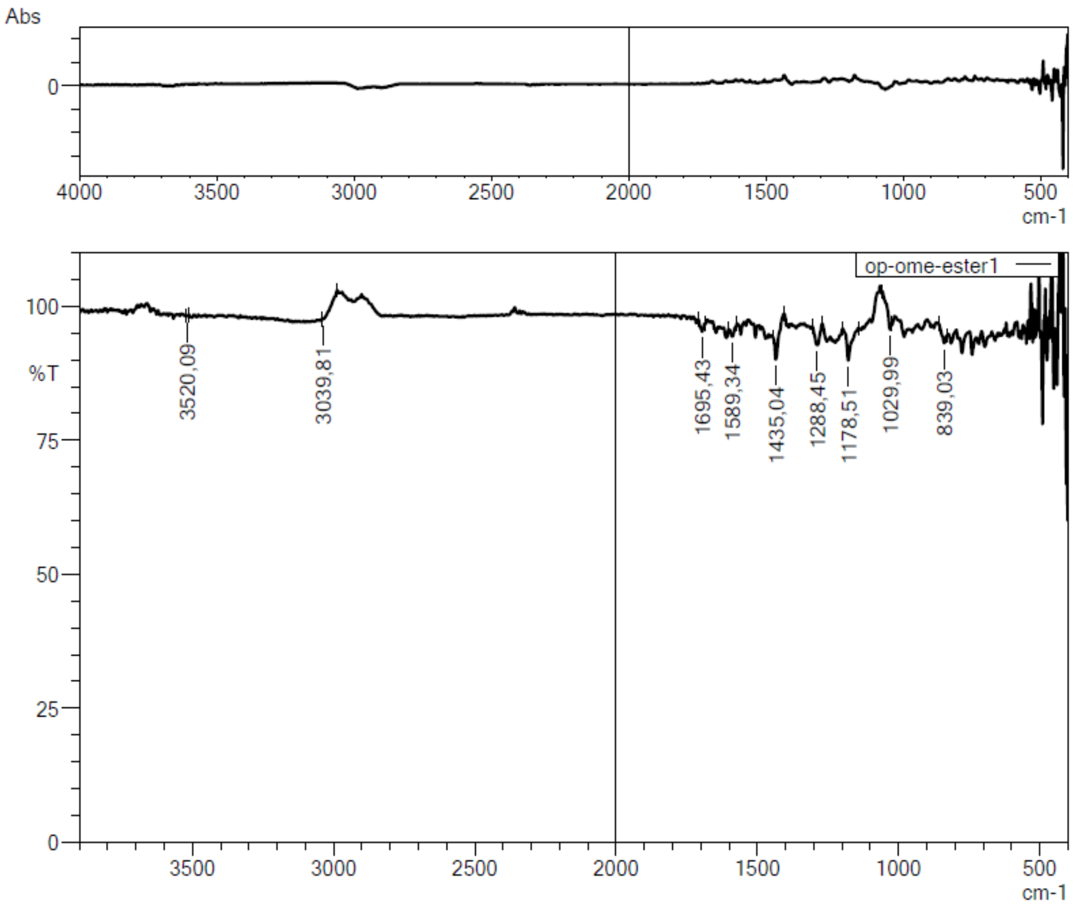


**Figure 5.** IR spectrum of compound **2b**

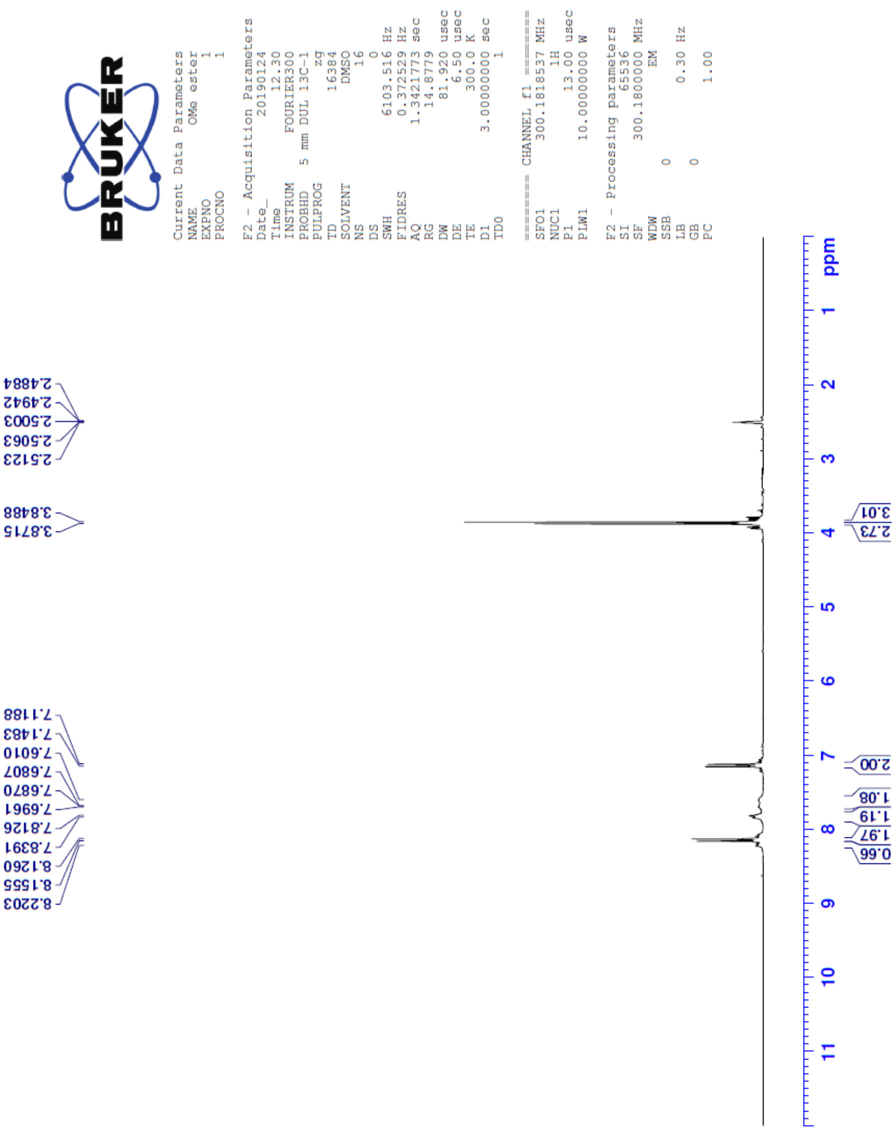

**Figure 6.** $^{1}$H-NMR spectra of compound **2b**

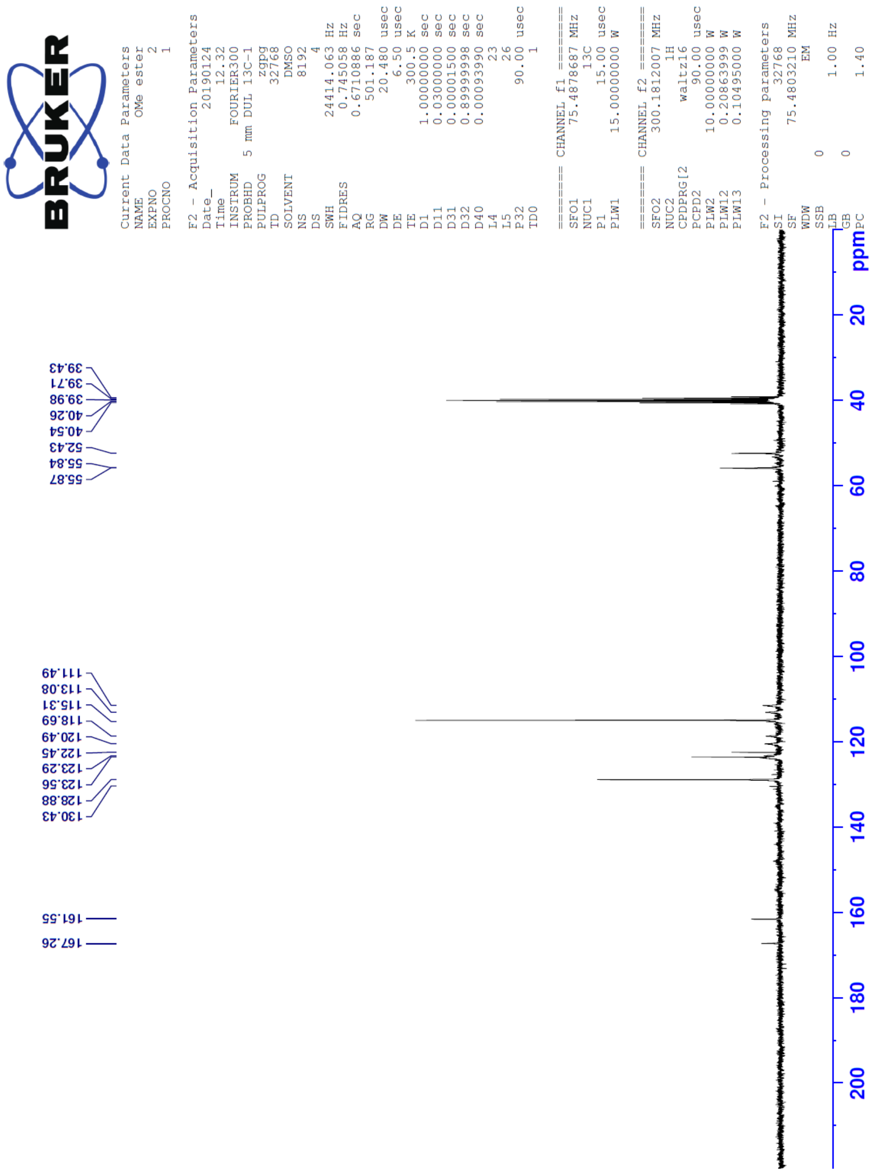


**Figure 7.** $^{13}$C-NMR spectra of compound **2b**

Data File: C:\LabSolutions\Data\Analiz\uac\OM-E_4.lcd

| Elmt | Val. | Min | Max | Elmt | Val. | Min | Max | Elmt | Val. | Min | Max | Elmt | Val. | Min | Max | Use Adduct |
|---|---|---|---|---|---|---|---|---|---|---|---|---|---|---|---|---|
| H | 1 | 10 | 40 | O | 2 | 2 | 5 | S | 2 | 0 | 2 | Ru | 2 | 0 | 0 | H |
| C | 4 | 15 | 30 | F | 1 | 0 | 0 | Cl | 1 | 0 | 0 | Pd | 2 | 0 | 0 | |
| N | 3 | 0 | 6 | P | 3 | 0 | 0 | Br | 1 | 0 | 0 | I | 3 | 0 | 0 | |

Error Margin (ppm): 5
HC Ratio: unlimited
Max Isotopes: 3
MSn Iso RI (%): 10.00

DBE Range: 5.0 - 22.0
Apply N Rule: yes
Isotope RI (%): 1.00
MSn Logic Mode: AND

Electron Ions: both
Use MSn Info: yes
Isotope Res: 9000
Max Results: 500

Event#: 1 MS(E+) Ret. Time : 4.813 -> 4.813 Scan# : 723 -> 723

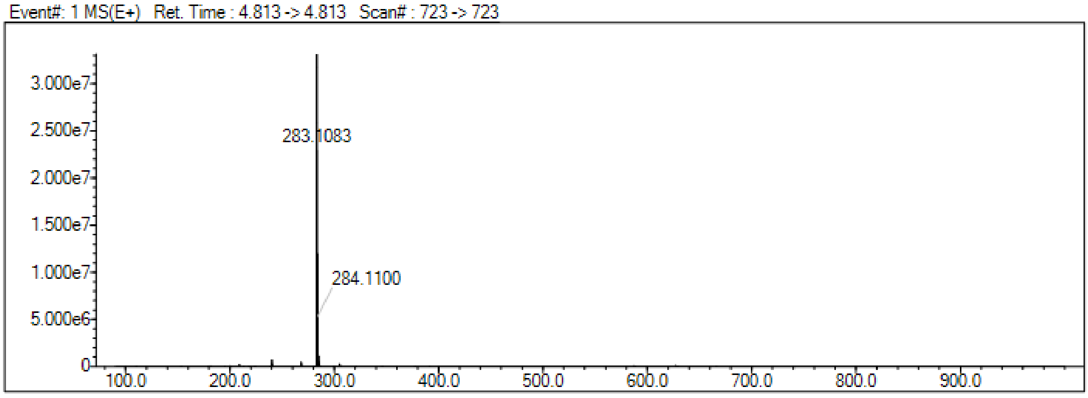


Measured region for 283.1083 m/z

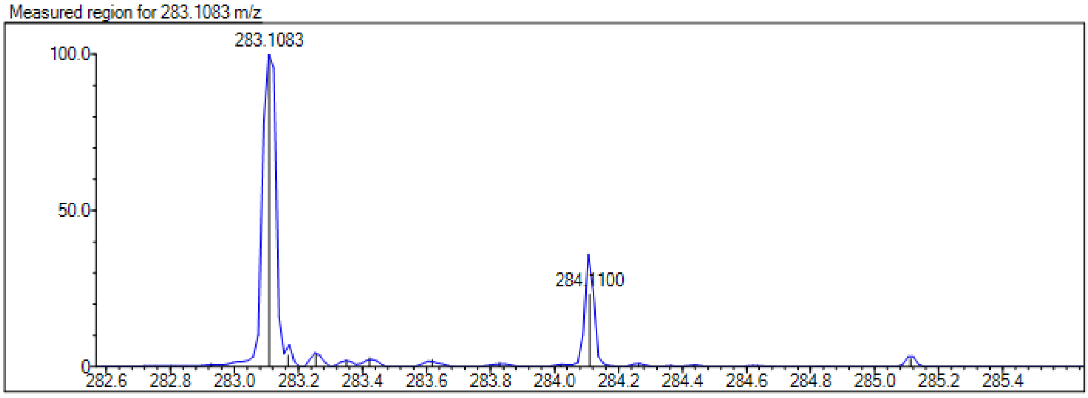


C16 H14 N2 O3 [M+H]+ : Predicted region for 283.1077 m/z

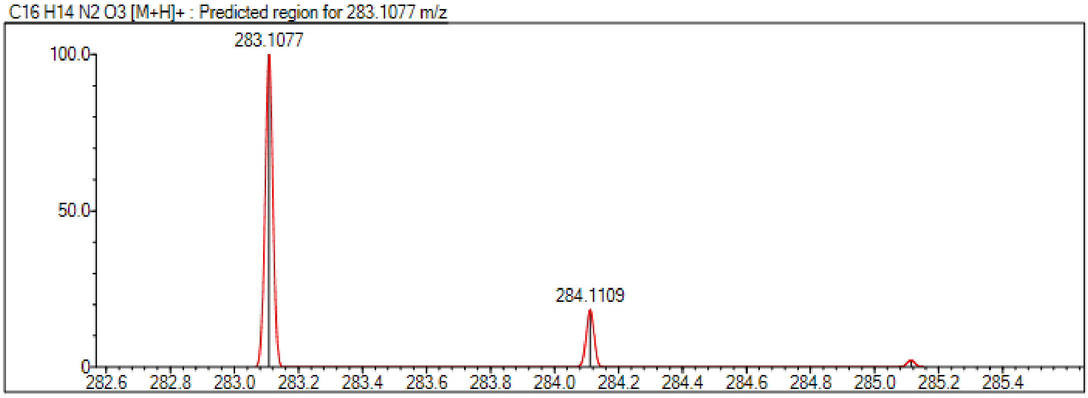


| Rank | Score | Formula (M) | Ion | Meas. m/z | Pred. m/z | Df. (mDa) | Df. (ppm) | Iso | DBE |
|---|---|---|---|---|---|---|---|---|---|
| 1 | 64.36 | C16 H14 N2 O3 | [M+H]+ | 283.1083 | 283.1077 | 0.6 | 2.12 | 66.21 | 11.0 |

**Figure 8.** Mass spectrum of compound **2b**

DOPNALAB

| | Item | Value |
|---|---|---|
| | Acquired Date&Time | 5.02.2019 14:09:09 |
| | Acquired by | System Administrator |
| | Filename | C:\Users\dopnalab\Desktop\derya\OP\op-oet-ester1.ispd |
| | Spectrum name | op-oet-ester1 |
| | Sample name | oet-ester |
| | Sample ID | |
| | Option | |
| | Comment | |
| | No. of Scans | 10 |
| | Resolution | 4 [cm-1] |
| | Apodization | Happ-Genzel |

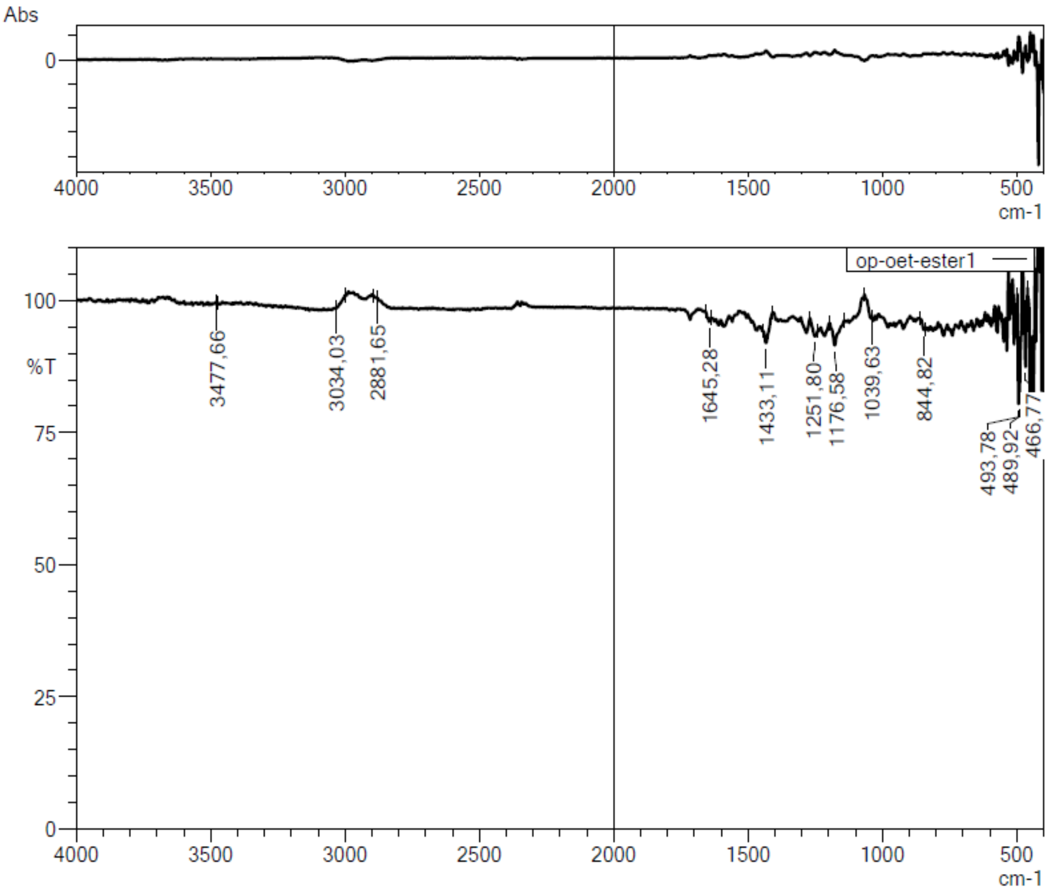


**Figure 9.** IR spectrum of compound **2c**

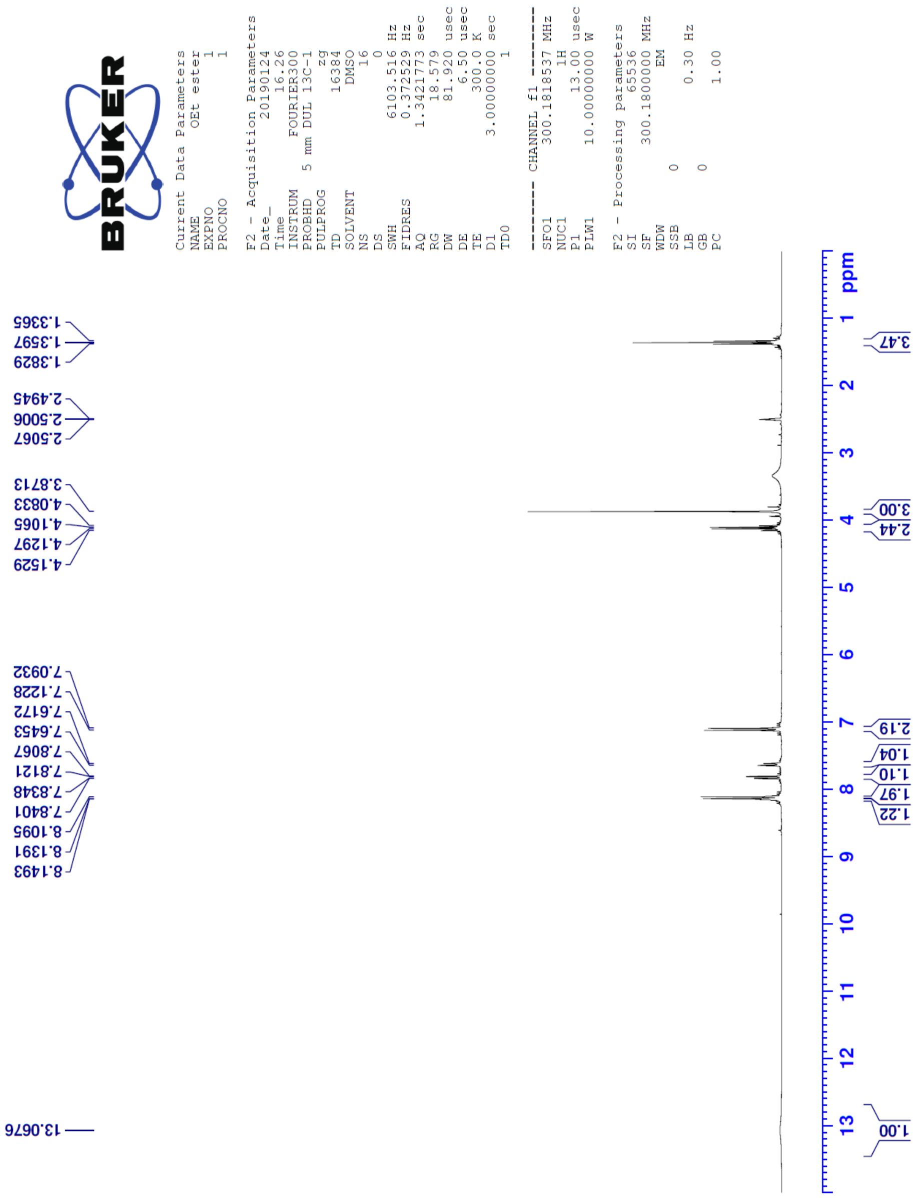


**Figure 10.** $^{1}$H-NMR spectra of compound **2c**

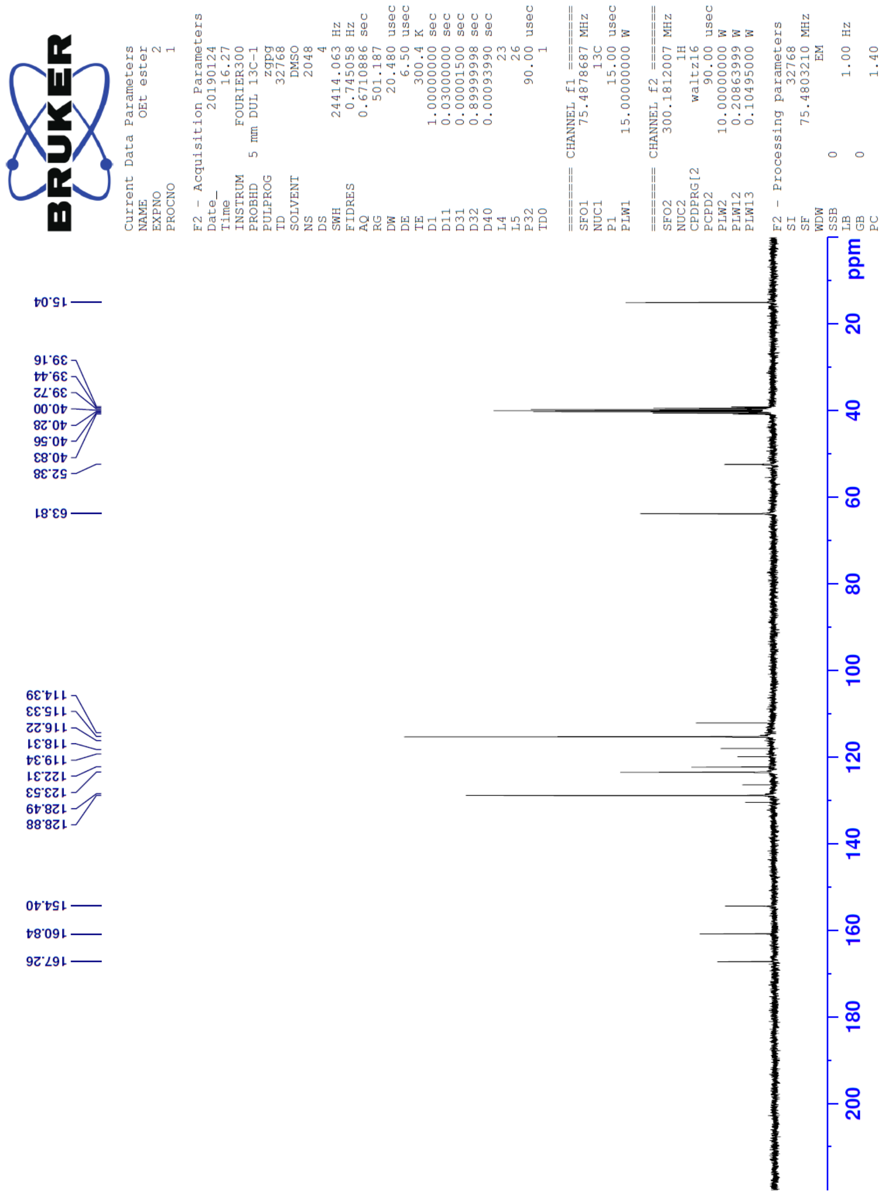


**Figure 11.** $^{13}$C-NMR spectra of compound **2c**

Formula Predictor Report - ET-E_4.lcd 

Data File: C:\LabSolutions\Data\Analiz\uac\ET-E_4.lcd

| Elmt | Val. | Min | Max | Elmt | Val. | Min | Max | Elmt | Val. | Min | Max | Elmt | Val. | Min | Max | Use Adduct |
|---|---|---|---|---|---|---|---|---|---|---|---|---|---|---|---|---|
| H | 1 | 10 | 40 | O | 2 | 3 | 5 | S | 2 | 0 | 2 | Ru | 2 | 0 | 0 | H |
| C | 4 | 15 | 30 | F | 1 | 0 | 0 | Cl | 1 | 0 | 0 | Pd | 2 | 0 | 0 | |
| N | 3 | 0 | 6 | P | 3 | 0 | 0 | Br | 1 | 0 | 0 | I | 3 | 0 | 0 | |

Error Margin (ppm): 15
HC Ratio: unlimited
Max Isotopes: 3
MSn Iso RI (%): 10.00

DBE Range: 5.0 - 22.0
Apply N Rule: yes
Isotope RI (%): 1.00
MSn Logic Mode: AND

Electron Ions: both
Use MSn Info: yes
Isotope Res: 9000
Max Results: 500

Event#: 1 MS(E+) Ret. Time : 5.947 Scan# : 893

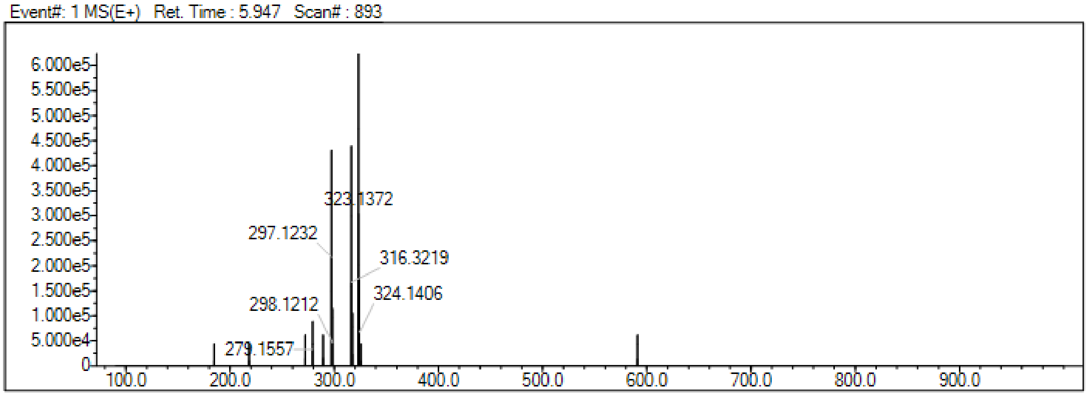


Measured region for 297.1232 m/z

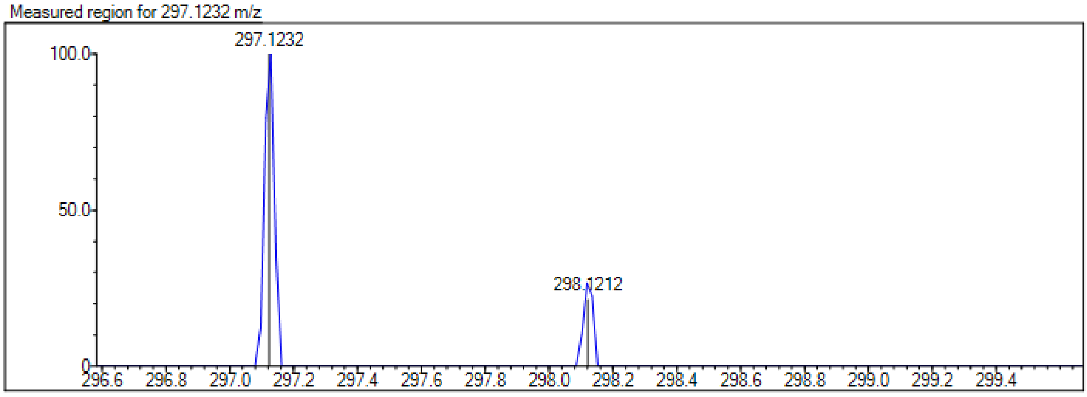


C17 H16 N2 O3 [M+H]+ : Predicted region for 297.1234 m/z

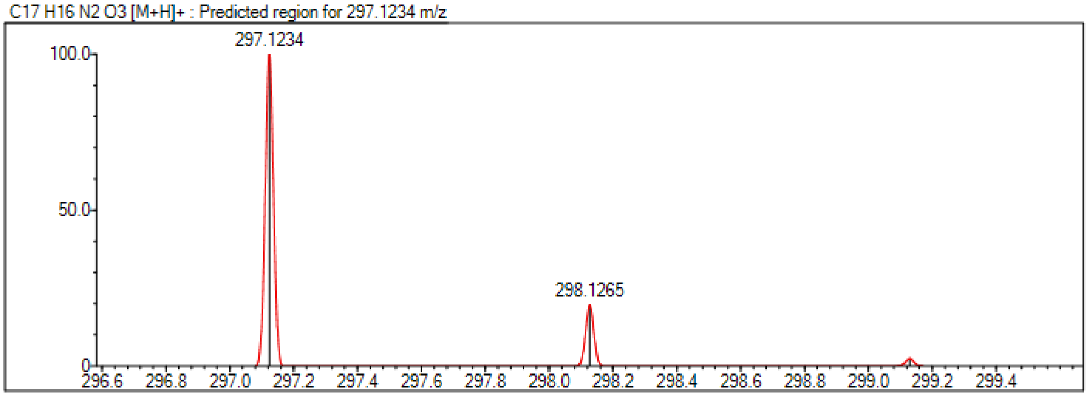


| Rank | Score | Formula (M) | Ion | Meas. m/z | Pred. m/z | Df. (mDa) | Df. (ppm) | Iso | DBE |
|---|---|---|---|---|---|---|---|---|---|
| 1 | 52.20 | C17 H16 N2 O3 | [M+H]+ | 297.1232 | 297.1234 | -0.2 | -0.67 | 52.20 | 11.0 |

**Figure 12.** Mass spectrum of compound **2c**

DOPNALAB

| | Item | Value |
|---|---|---|
| | Acquired Date&Time | 5.02.2019 14:06:58 |
| | Acquired by | System Administrator |
| | Filename | C:\Users\dopnalab\Desktop\derya\OP\op-oh-oks1.ispd |
| | Spectrum name | op-oh-oks1 |
| | Sample name | oh-oks |
| | Sample ID | |
| | Option | |
| | Comment | |
| | No. of Scans | 10 |
| | Resolution | 4 [cm-1] |
| | Apodization | Happ-Genzel |

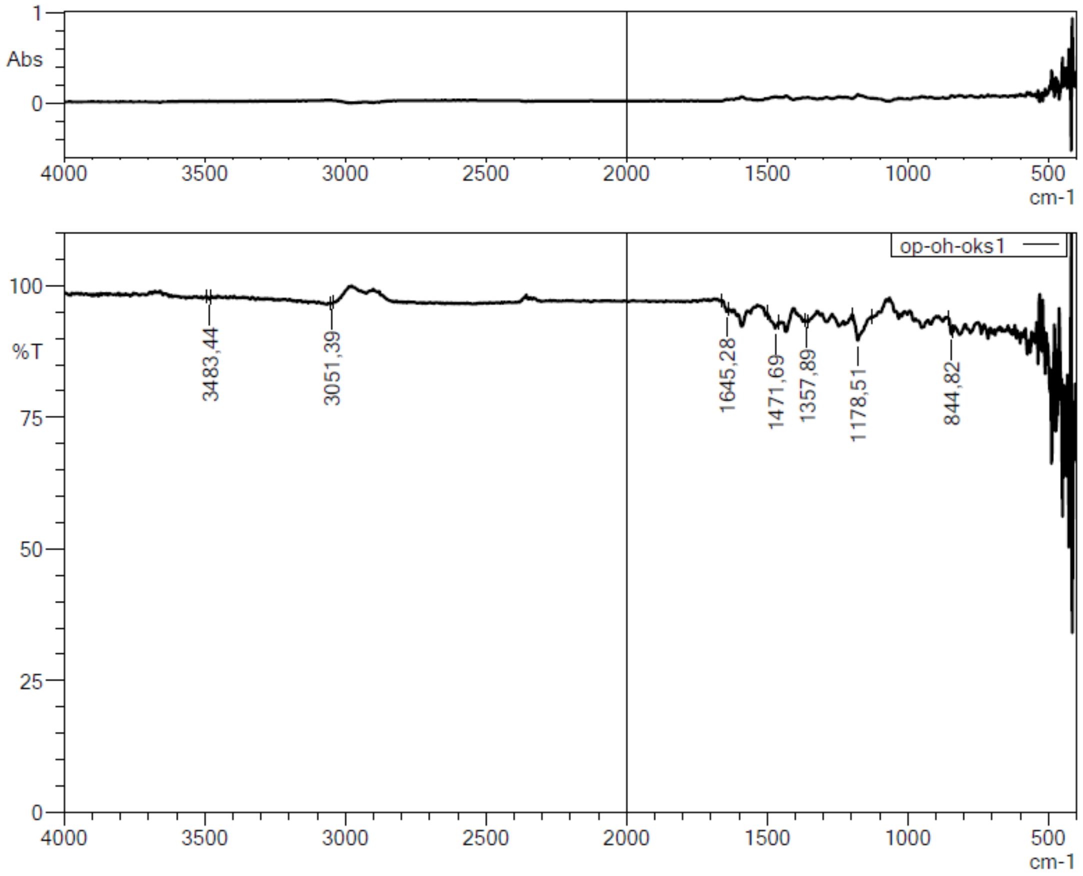


**Figure 13.** IR spectrum of compound **4a**

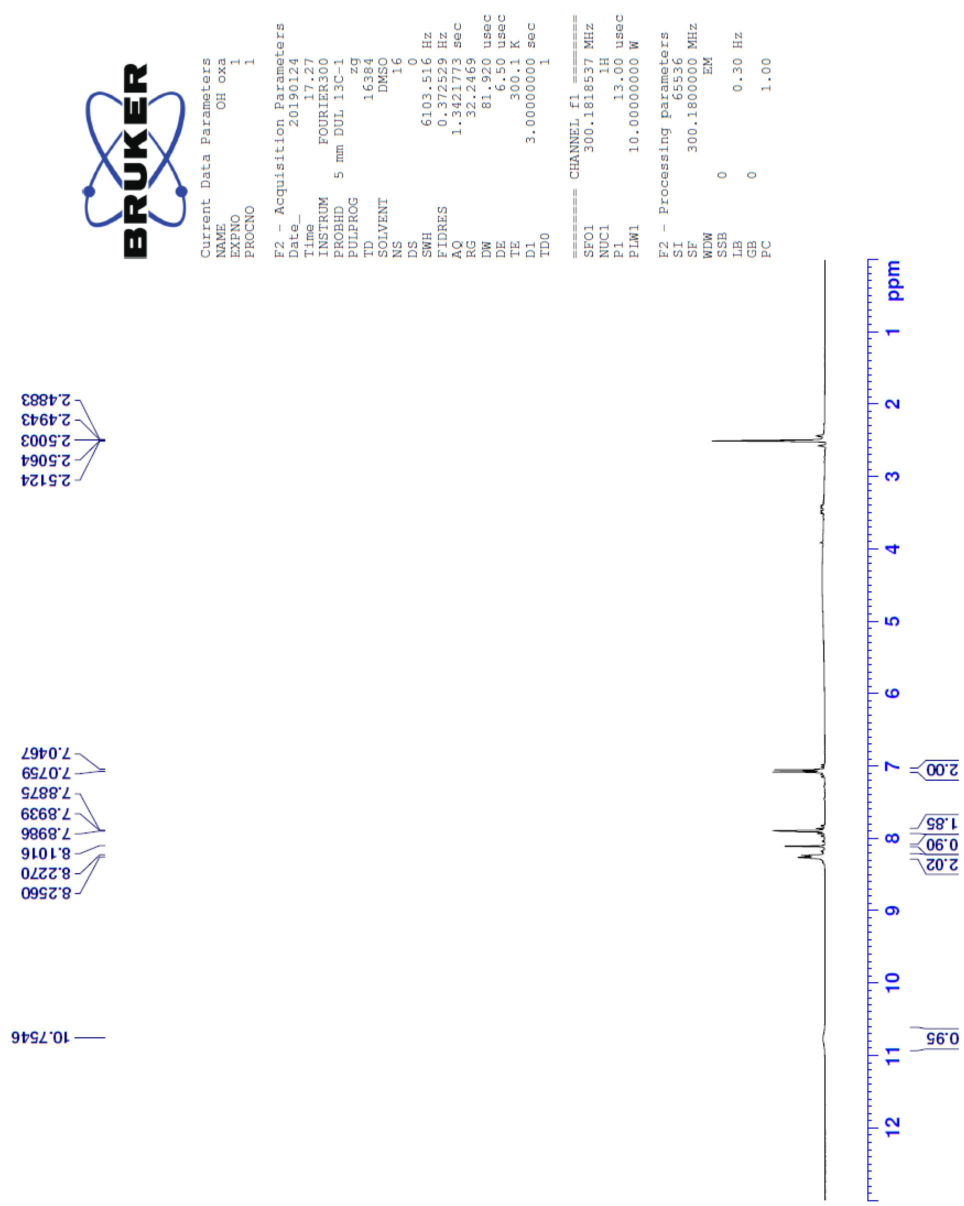


**Figure 14.** $^1$H-NMR spectra of compound **4a**

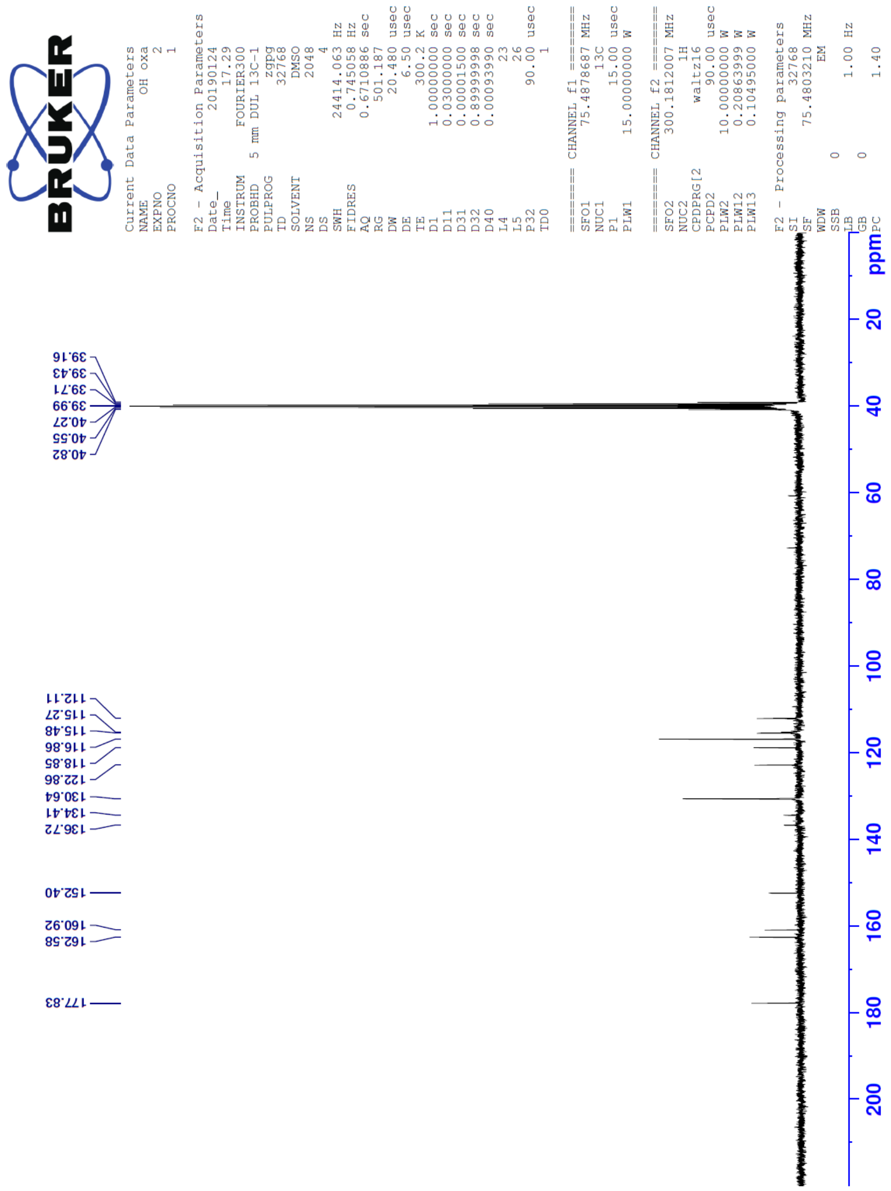


**Figure 15.** $^{13}$C-NMR spectra of compound **4a**

Formula Predictor Report - OH-OKS_4.lcd Page 1 of 1

Data File: C:\LabSolutions\Data\Analiz\uac\OH-OKS_4.lcd

| Elmt | Val. | Min | Max | Elmt | Val. | Min | Max | Elmt | Val. | Min | Max | Elmt | Val. | Min | Max | Use Adduct |
|---|---|---|---|---|---|---|---|---|---|---|---|---|---|---|---|---|
| H | 1 | 10 | 40 | O | 2 | 2 | 5 | S | 2 | 0 | 2 | Ru | 2 | 0 | 0 | H |
| C | 4 | 15 | 30 | F | 1 | 0 | 0 | Cl | 1 | 0 | 0 | Pd | 2 | 0 | 0 | |
| N | 3 | 0 | 6 | P | 3 | 0 | 0 | Br | 1 | 0 | 0 | I | 3 | 0 | 0 | |

Error Margin (ppm): 15
HC Ratio: unlimited
Max Isotopes: 3
MSn Iso RI (%): 10.00

DBE Range: 5.0 - 22.0
Apply N Rule: yes
Isotope RI (%): 1.00
MSn Logic Mode: AND

Electron Ions: both
Use MSn Info: yes
Isotope Res: 9000
Max Results: 500

Event#: 1 MS(E+) Ret. Time : 4.253 -> 4.253 Scan# : 639 -> 639

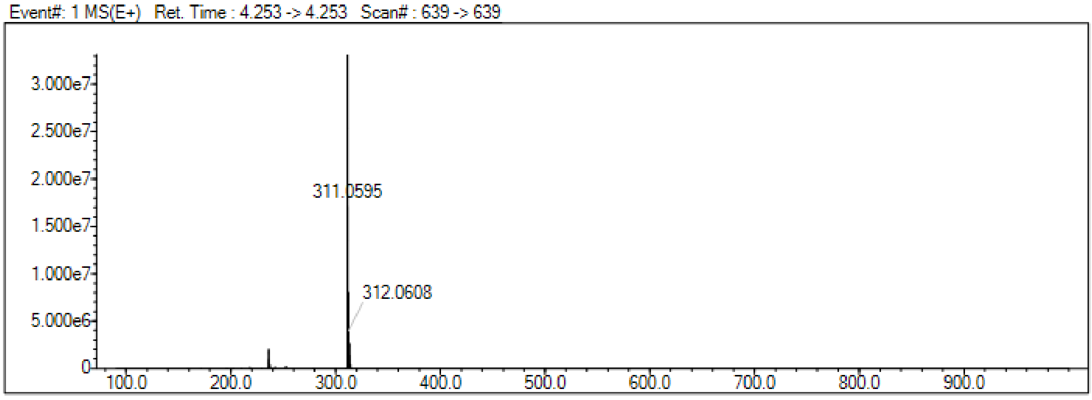


Measured region for 311.0595 m/z

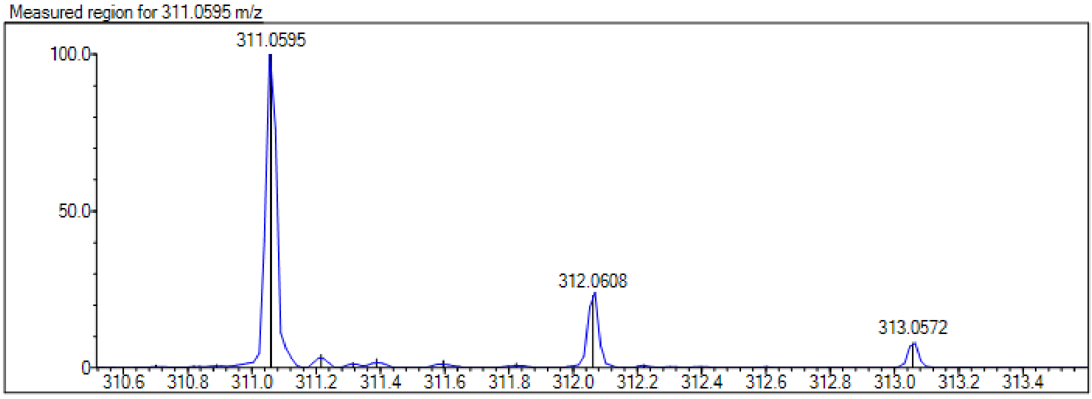


C15 H10 N4 O2 S [M+H]+ : Predicted region for 311.0597 m/z

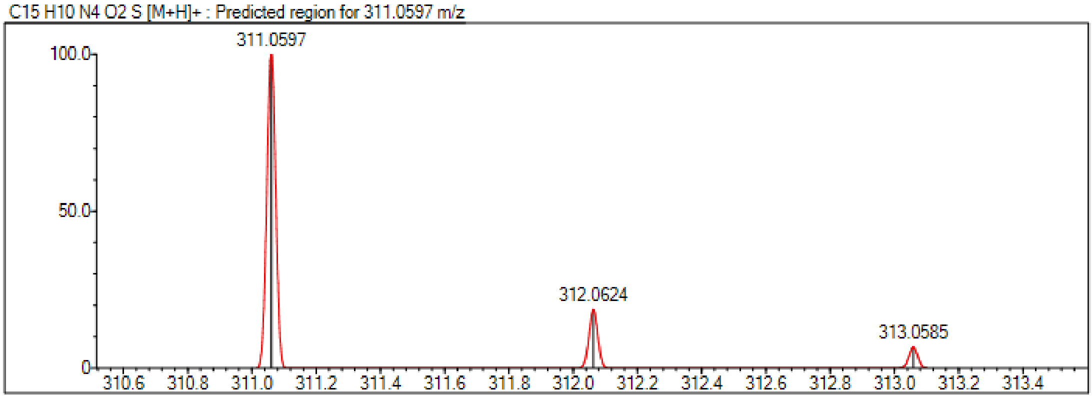


| Rank | Score | Formula (M) | Ion | Meas. m/z | Pred. m/z | Df. (mDa) | Df. (ppm) | Iso | DBE |
|---|---|---|---|---|---|---|---|---|---|
| 1 | 85.30 | C15 H10 N4 O2 S | [M+H]+ | 311.0595 | 311.0597 | -0.2 | -0.64 | 85.30 | 13.0 |

**Figure 16.** Mass spectrum of compound **4a**

DOPNALAB

| | Item | Value |
|---|---|---|
| | Acquired Date&Time | 5.02.2019 14:01:22 |
| | Acquired by | System Administrator |
| | Filename | C:\Users\dopnalab\Desktop\derya\OP\op-ome-oks1.ispd |
| | Spectrum name | op-ome-oks1 |
| | Sample name | ome-oks |
| | Sample ID | |
| | Option | |
| | Comment | |
| | No. of Scans | 10 |
| | Resolution | 4 [cm-1] |
| | Apodization | Happ-Genzel |

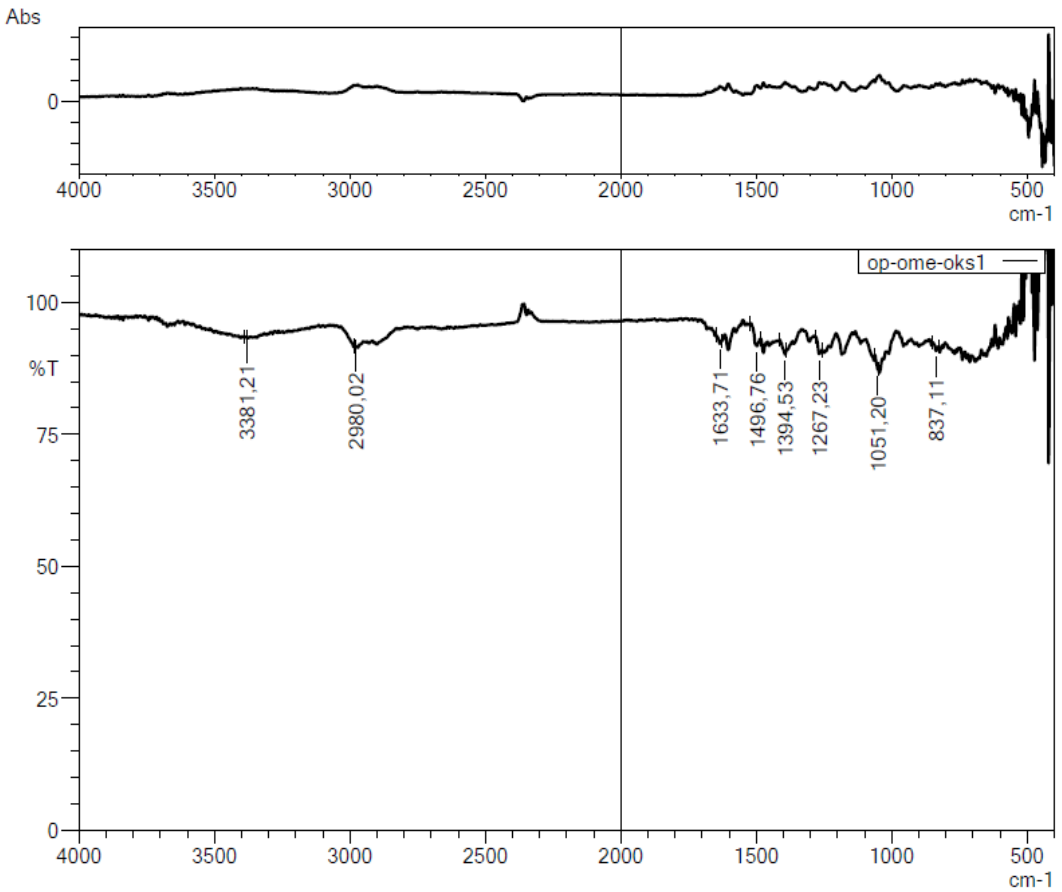


**Figure 17.** IR spectrum of compound **4b**

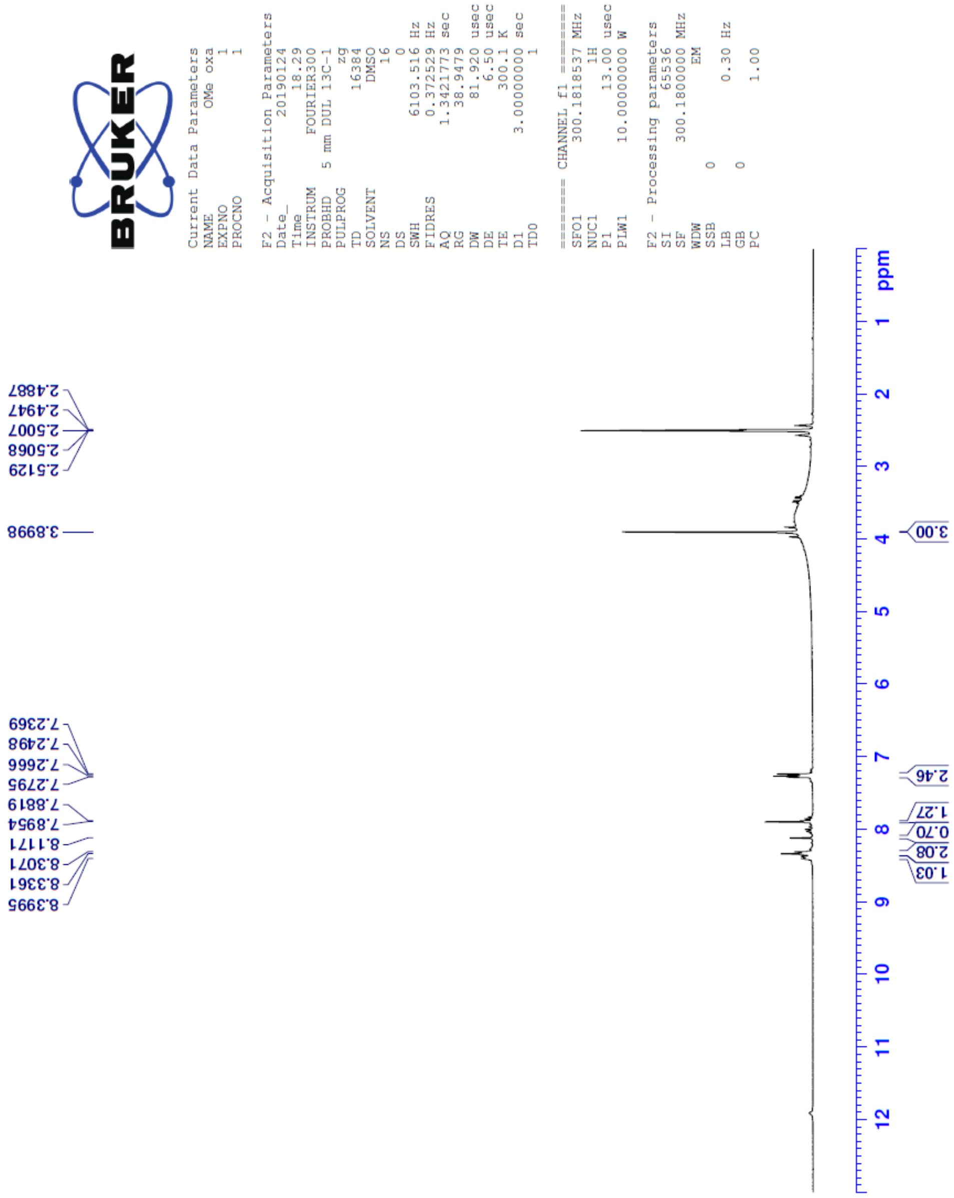


**Figure 18.** $^{1}$H-NMR spectra of compound **4b**

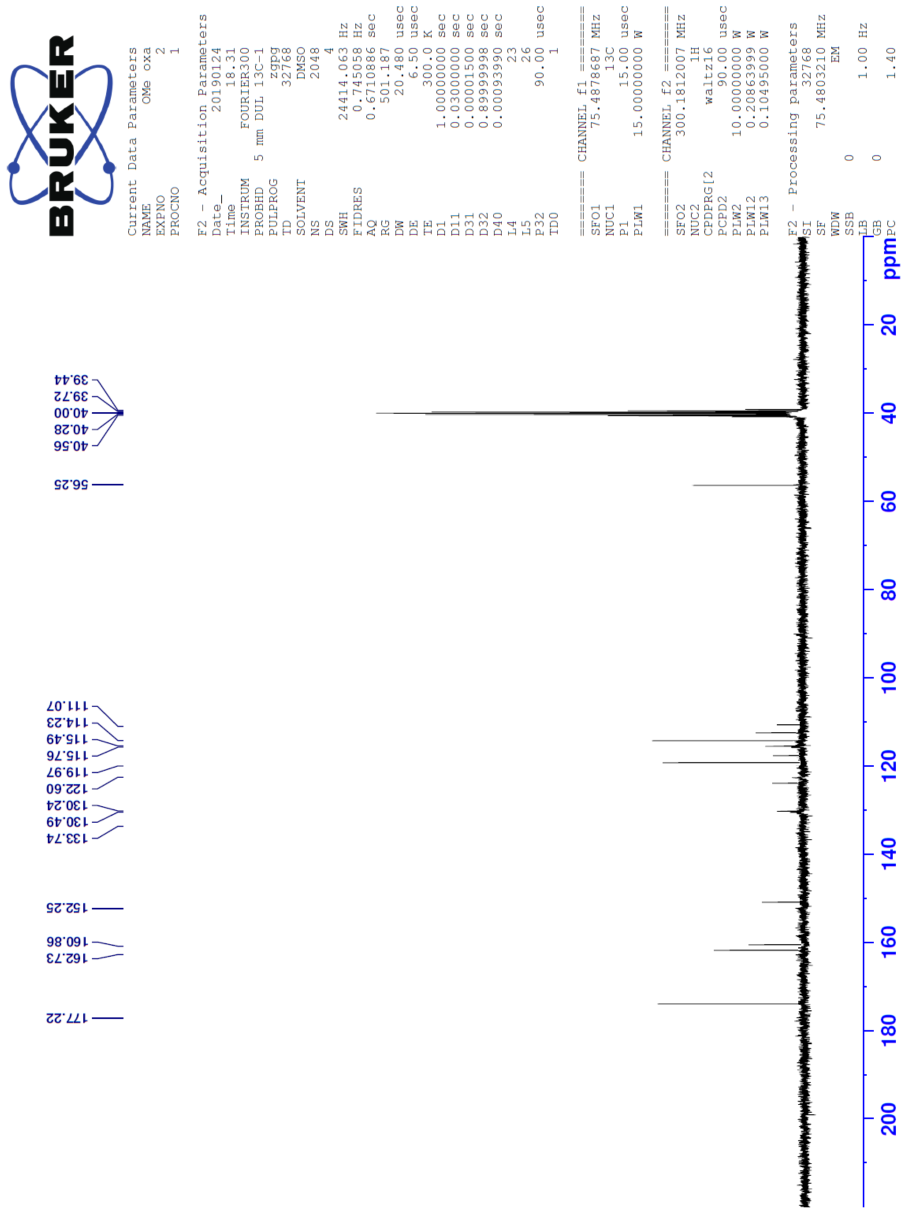


**Figure 19.** $^{13}$C-NMR spectra of compound **4b**

Data File: C:\LabSolutions\Data\Analiz\uac\OM2_7.lcd

| Elmt | Val. | Min | Max | Elmt | Val. | Min | Max | Elmt | Val. | Min | Max | Elmt | Val. | Min | Max | Use Adduct |
|---|---|---|---|---|---|---|---|---|---|---|---|---|---|---|---|---|
| H | 1 | 10 | 40 | O | 2 | 2 | 5 | S | 2 | 0 | 2 | Ru | 2 | 0 | 0 | H |
| C | 4 | 15 | 30 | F | 1 | 0 | 0 | Cl | 1 | 0 | 0 | Pd | 2 | 0 | 0 | |
| N | 3 | 0 | 6 | P | 3 | 0 | 0 | Br | 1 | 0 | 0 | I | 3 | 0 | 0 | |

| | | | | | |
|---|---|---|---|---|---|
| Error Margin (ppm): | 15 | DBE Range: | 5.0 - 22.0 | Electron Ions: | both |
| HC Ratio: | unlimited | Apply N Rule: | yes | Use MSn Info: | yes |
| Max Isotopes: | 3 | Isotope RI (%): | 1.00 | Isotope Res: | 9000 |
| MSn Iso RI (%): | 10.00 | MSn Logic Mode: | AND | Max Results: | 500 |

Event#: 1 MS(E+) Ret. Time : 5.253 Scan# : 789

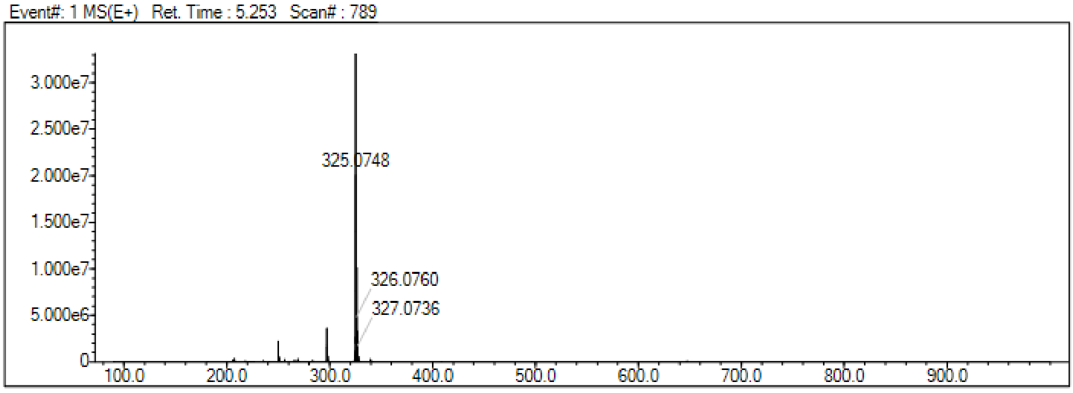


Measured region for 325.0748 m/z

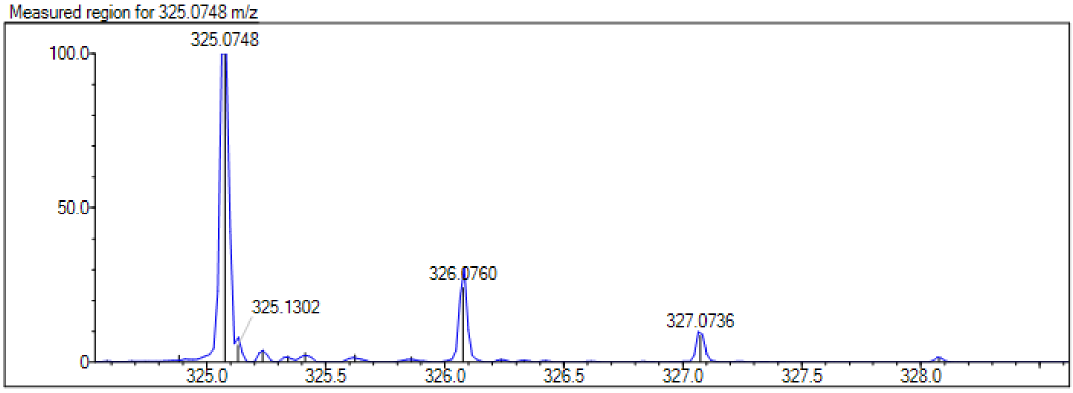


C16 H12 N4 O2 S [M+H]+ : Predicted region for 325.0754 m/z

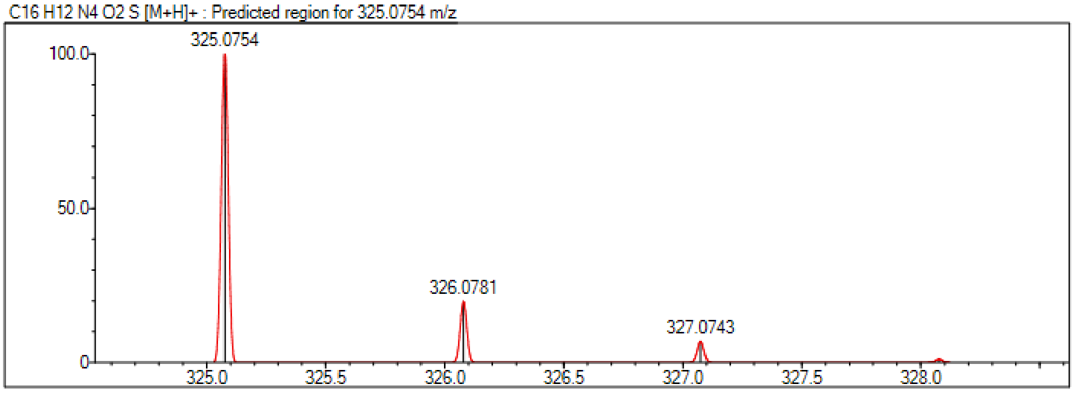


| Rank | Score | Formula (M) | Ion | Meas. m/z | Pred. m/z | Df. (mDa) | Df. (ppm) | Iso | DBE |
|---|---|---|---|---|---|---|---|---|---|
| 1 | 80.05 | C16 H12 N4 O2 S | [M+H]+ | 325.0748 | 325.0754 | -0.6 | -1.85 | 81.79 | 13.0 |

**Figure 20.** Mass spectrum of compound **4b**

DOPNALAB

| | Item | Value |
|---|---|---|
| | Acquired Date&Time | 5.02.2019 14:04:57 |
| | Acquired by | System Administrator |
| | Filename | C:\Users\dopnalab\Desktop\derya\OP\op-oet-oks1.ispd |
| | Spectrum name | op-oet-oks1 |
| | Sample name | oet-oks |
| | Sample ID | |
| | Option | |
| | Comment | |
| | No. of Scans | 10 |
| | Resolution | 4 [cm-1] |
| | Apodization | Happ-Genzel |

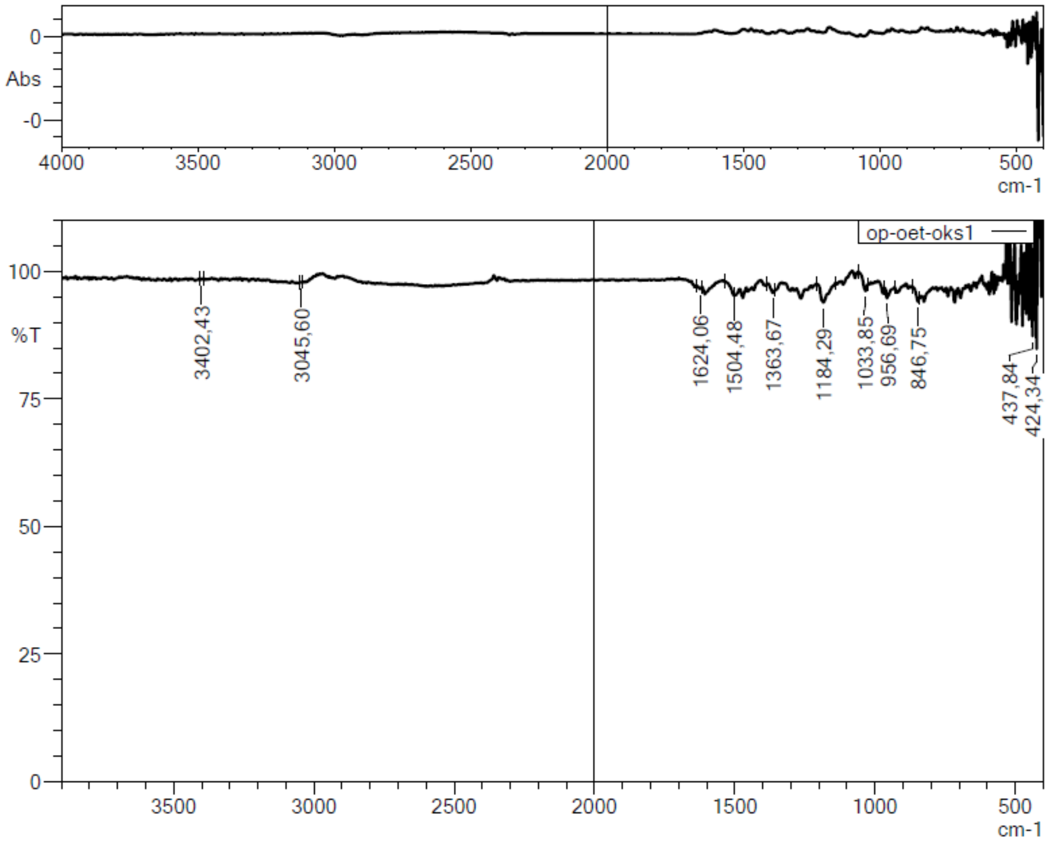


**Figure 21.** IR spectrum of compound **4c**

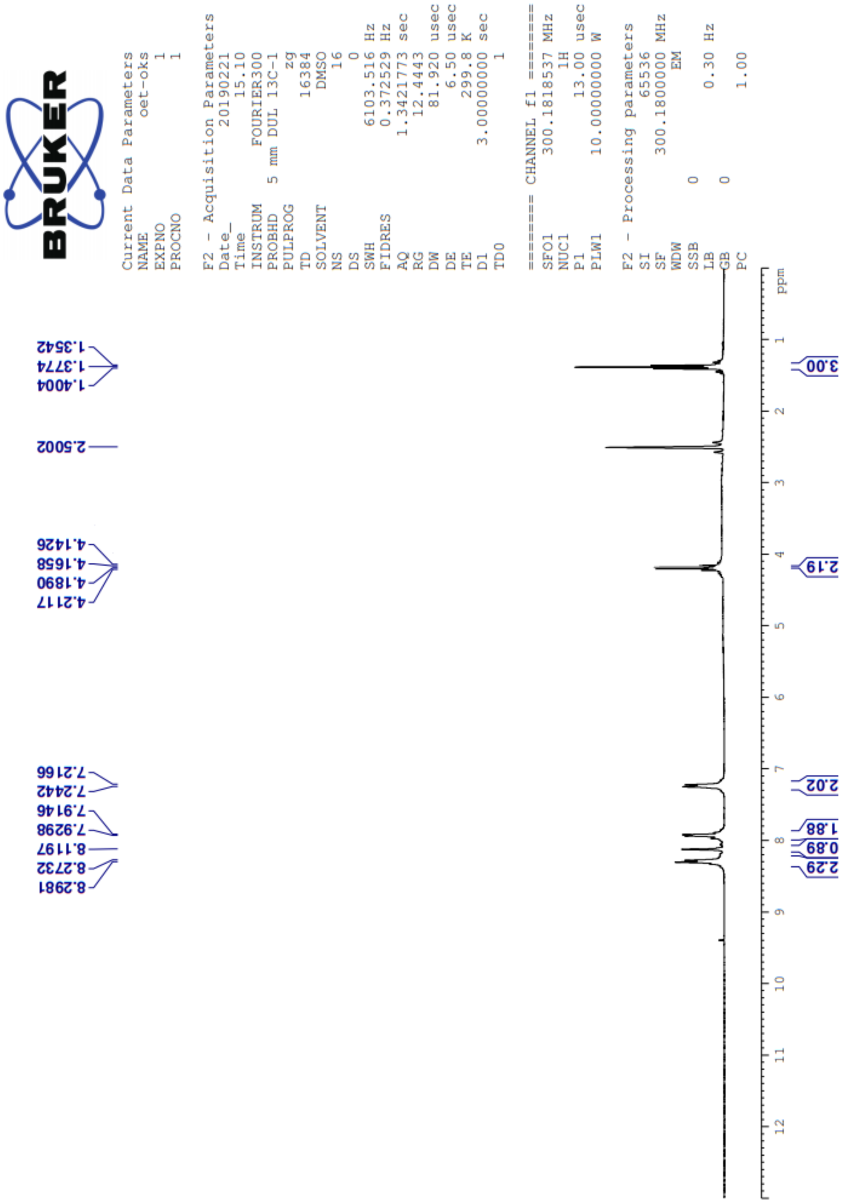


**Figure 22.** $^{1}$H-NMR spectra of compound **4c**

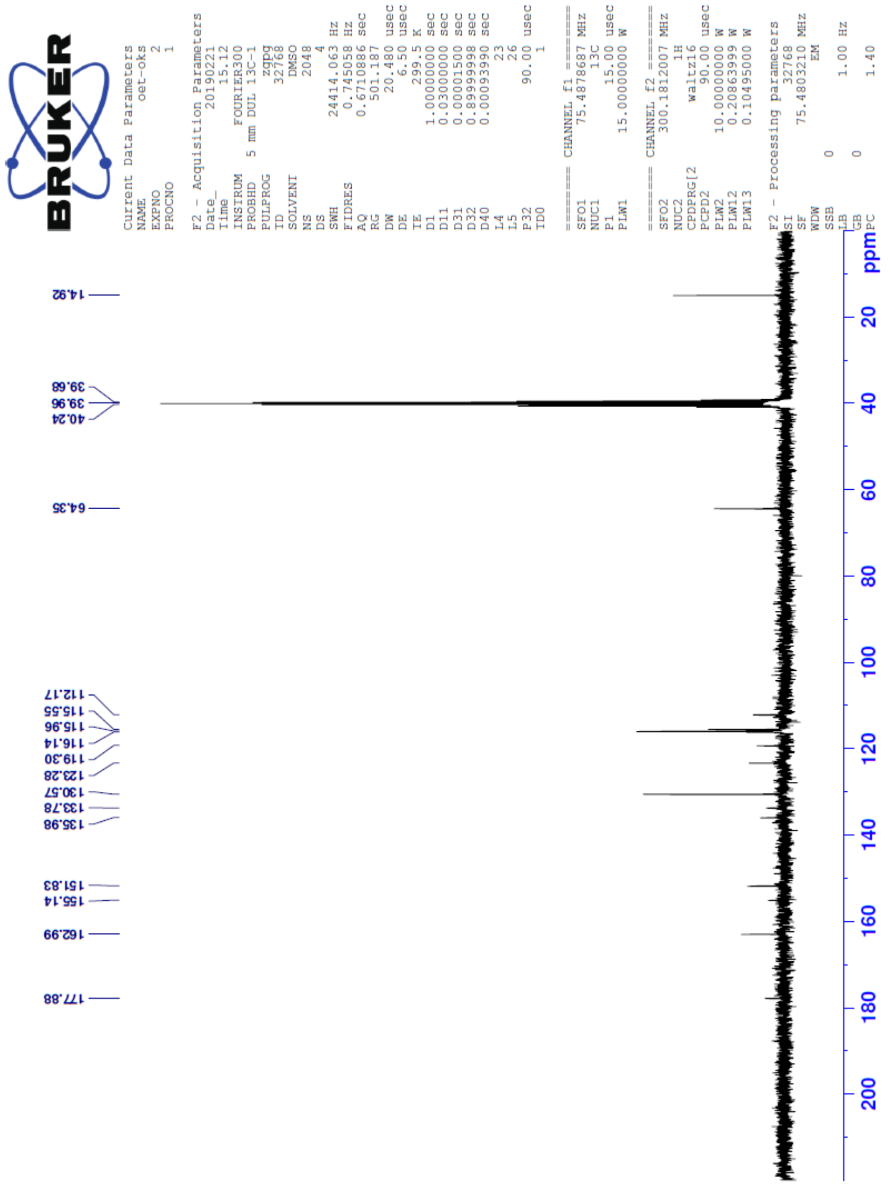


**Figure 23.** $^{13}$C-NMR spectra of compound **4c**

Data File: C:\LabSolutions\Data\Analiz\uac\ETO_6.lcd

| Elmt | Val. | Min | Max | Elmt | Val. | Min | Max | Elmt | Val. | Min | Max | Elmt | Val. | Min | Max | Use Adduct |
|---|---|---|---|---|---|---|---|---|---|---|---|---|---|---|---|---|
| H | 1 | 10 | 40 | O | 2 | 2 | 5 | S | 2 | 0 | 2 | Ru | 2 | 0 | 0 | H |
| C | 4 | 15 | 30 | F | 1 | 0 | 0 | Cl | 1 | 0 | 0 | Pd | 2 | 0 | 0 | |
| N | 3 | 0 | 6 | P | 3 | 0 | 0 | Br | 1 | 0 | 0 | I | 3 | 0 | 0 | |

Error Margin (ppm): 5
HC Ratio: unlimited
Max Isotopes: 3
MSn Iso RI (%): 10.00

DBE Range: 5.0 - 22.0
Apply N Rule: yes
Isotope RI (%): 1.00
MSn Logic Mode: AND

Electron Ions: both
Use MSn Info: yes
Isotope Res: 9000
Max Results: 500

Event#: 1 MS(E+) Ret. Time : 5.693 Scan# : 855

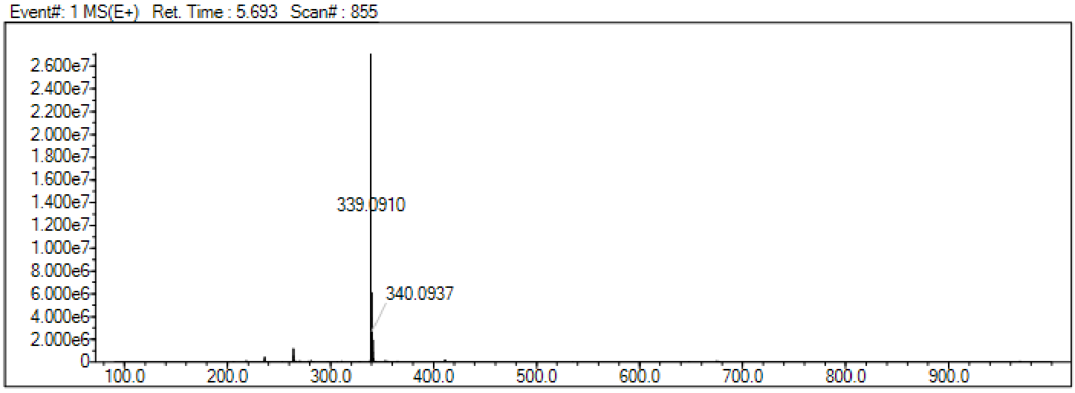


Measured region for 339.0910 m/z

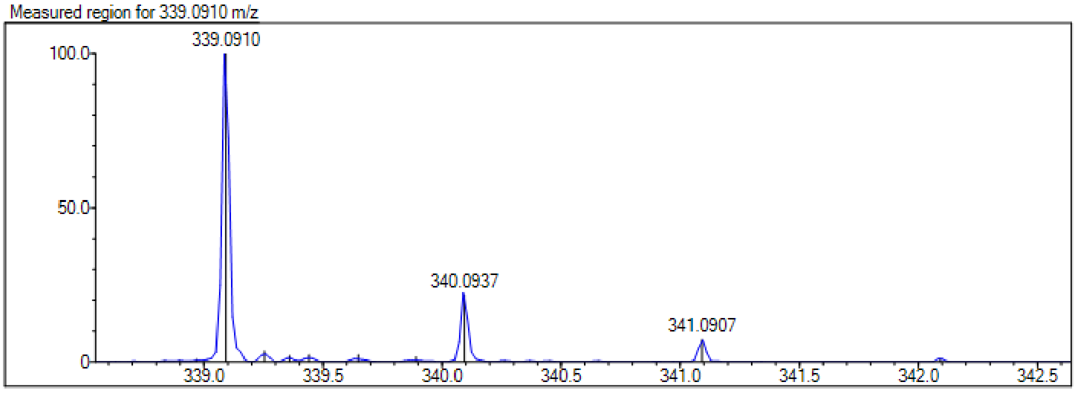


C17 H14 N4 O2 S [M+H]+ : Predicted region for 339.0910 m/z

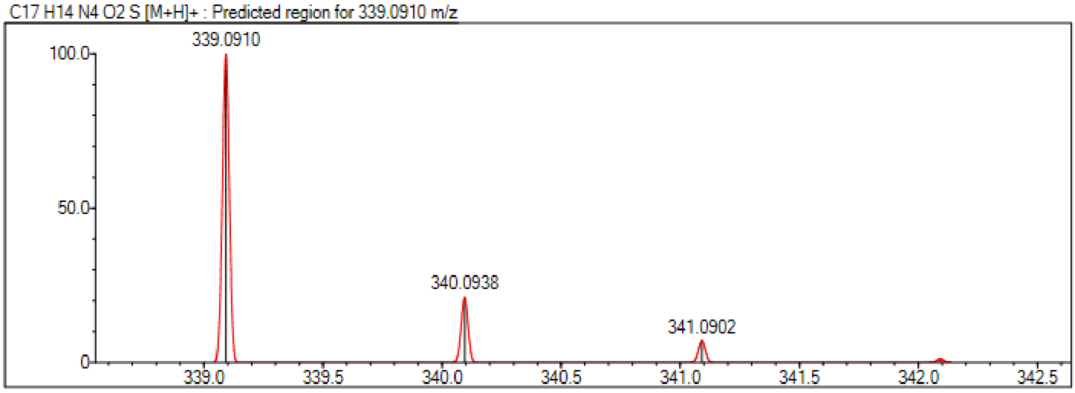


| Rank | Score | Formula (M) | Ion | Meas. m/z | Pred. m/z | Df. (mDa) | Df. (ppm) | Iso | DBE |
|---|---|---|---|---|---|---|---|---|---|
| 1 | 80.01 | C17 H14 N4 O2 S | [M+H]+ | 339.0910 | 339.0910 | -0.0 | 0.00 | 80.01 | 13.0 |

**Figure 24.** Mass spectrum of compound **4c**

DOPNALAB

| Item | Value |
|---|---|
| Acquired Date&Time | 5.02.2019 12:08:37 |
| Acquired by | System Administrator |
| Filename | C:\Users\dopnalab\Desktop\derya\OP\op-81.ispd |
| Spectrum name | op-81 |
| Sample name | OP-8 |
| Sample ID | |
| Option | |
| Comment | |
| No. of Scans | 10 |
| Resolution | 4 [cm-1] |
| Apodization | Happ-Genzel |

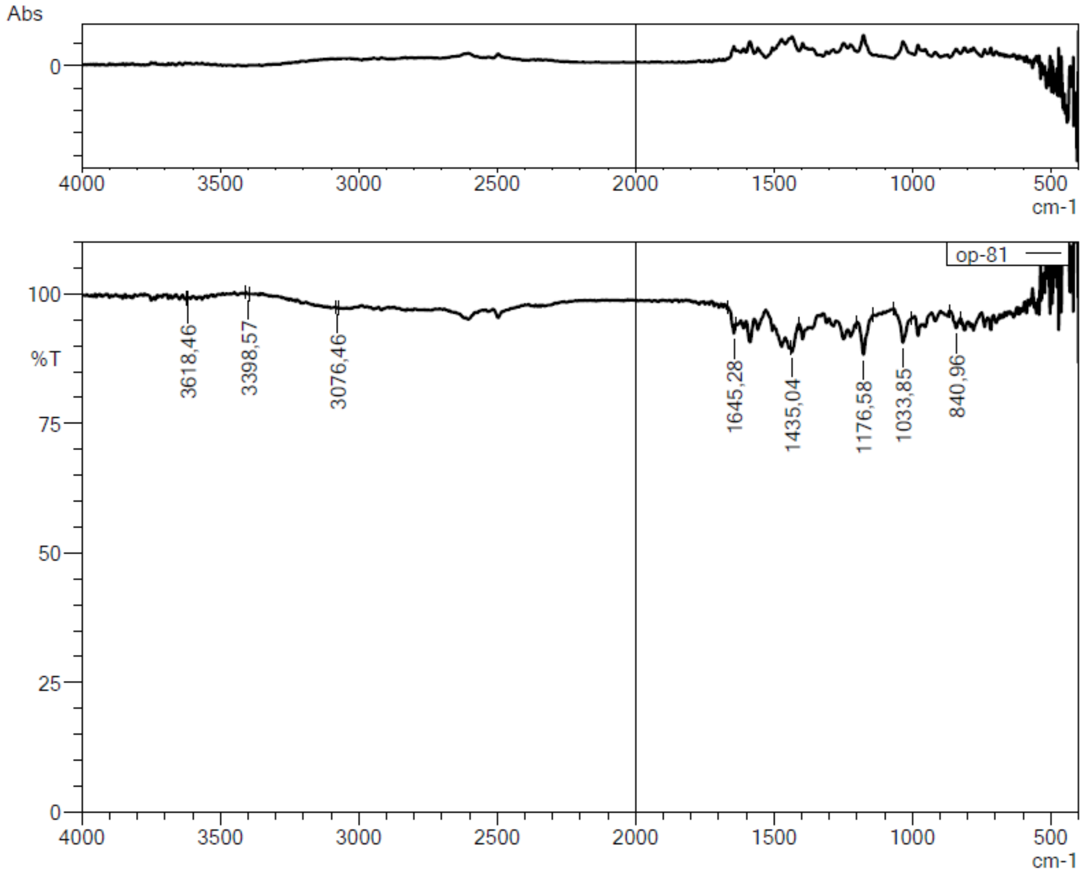


**Figure 25.** IR spectrum of compound **5a**

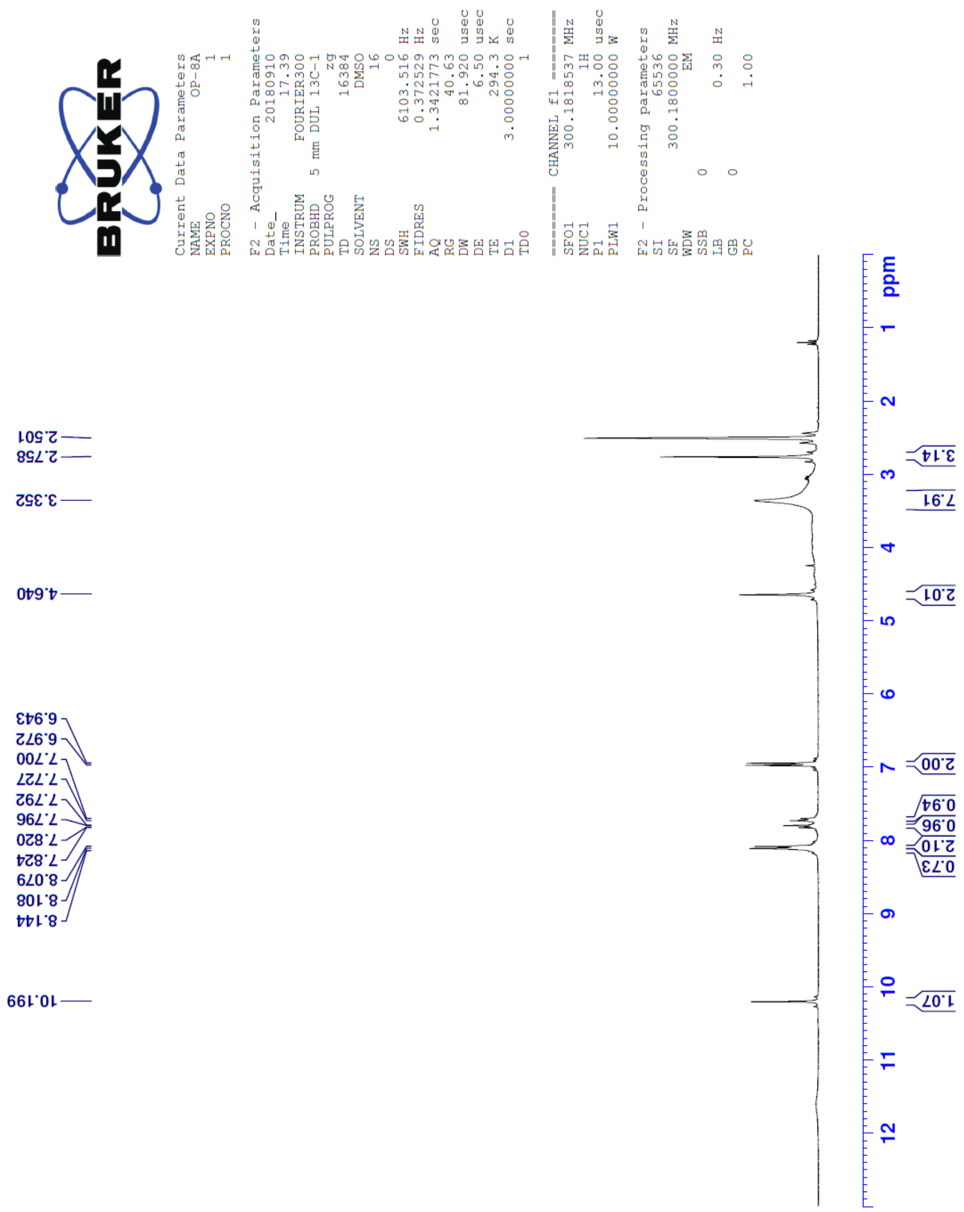


**Figure 26.** $^{1}$H-NMR spectra of compound **5a**

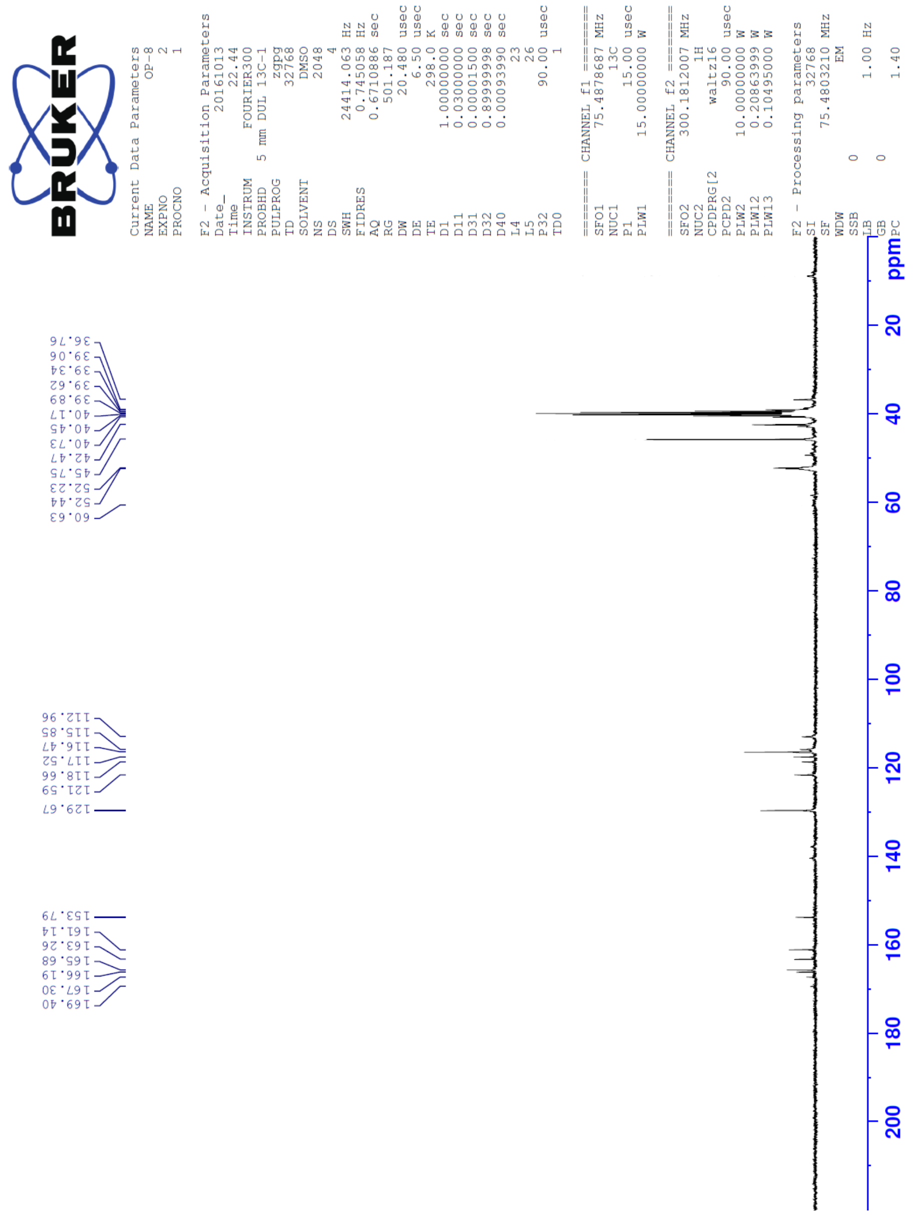


**Figure 27.** $^{13}$C-NMR spectra of compound **5a**

Data File: C:\LabSolutions\Data\Analiz\uac\OP-8_3.lcd

| Elmt | Val. | Min | Max | Elmt | Val. | Min | Max | Elmt | Val. | Min | Max | Elmt | Val. | Min | Max | Use Adduct |
|---|---|---|---|---|---|---|---|---|---|---|---|---|---|---|---|---|
| H | 1 | 20 | 40 | O | 2 | 1 | 5 | S | 2 | 1 | 3 | Ru | 2 | 0 | 0 | H |
| C | 4 | 15 | 30 | F | 1 | 0 | 0 | Cl | 1 | 0 | 0 | Pd | 2 | 0 | 0 | |
| N | 3 | 6 | 8 | P | 3 | 0 | 0 | Br | 1 | 0 | 1 | I | 3 | 0 | 0 | |

Error Margin (ppm): 15
HC Ratio: unlimited
Max Isotopes: 3
MSn Iso RI (%): 10.00

DBE Range: 12.0 - 20.0
Apply N Rule: yes
Isotope RI (%): 1.00
MSn Logic Mode: AND

Electron Ions: both
Use MSn Info: yes
Isotope Res: 9000
Max Results: 500

Event#: 1 MS(E+) Ret. Time : 1.907 Scan# : 287

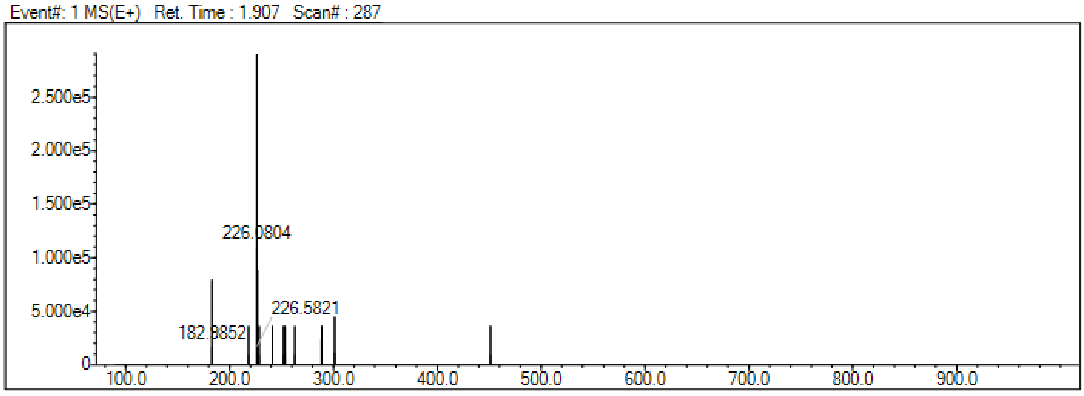


Measured region for 226.0804 m/z

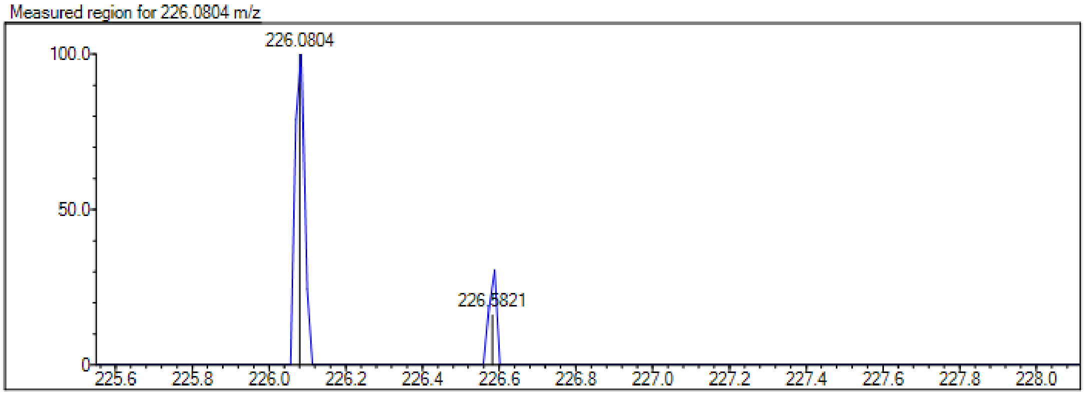


C22 H22 N6 O3 S [M+2H]2+ : Predicted region for 226.0810 m/z

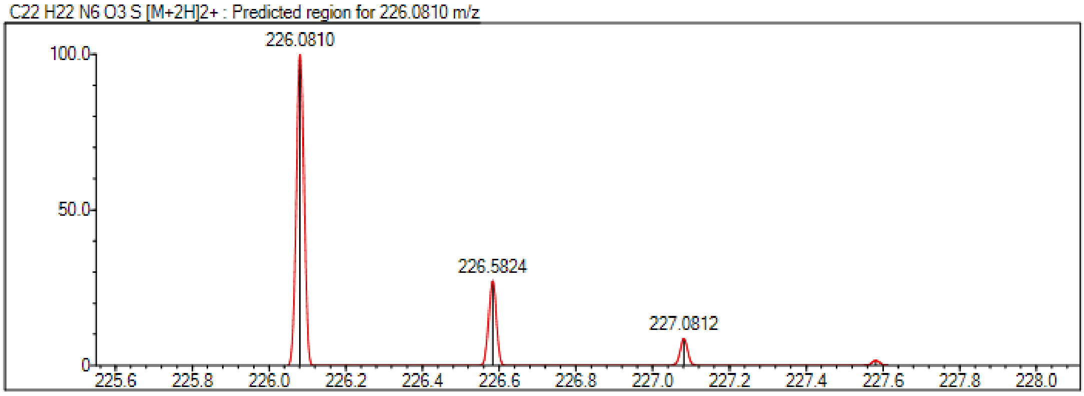


| Rank | Score | Formula (M) | Ion | Meas. m/z | Pred. m/z | Df. (mDa) | Df. (ppm) | Iso | DBE |
|---|---|---|---|---|---|---|---|---|---|
| 1 | 0.00 | C22 H22 N6 O3 S | [M+2H]2+ | 226.0804 | 226.0810 | -0.6 | -2.65 | 0.00 | 15.0 |

**Figure 28.** Mass spectrum of compound **5a**

DOPNALAB

| | Item | Value |
|---|---|---|
| | Acquired Date&Time | 5.02.2019 13:51:36 |
| | Acquired by | System Administrator |
| | Filename | C:\Users\dopnalab\Desktop\derya\OP\op-331.ispd |
| | Spectrum name | op-331 |
| | Sample name | OP-33 |
| | Sample ID | |
| | Option | |
| | Comment | |
| | No. of Scans | 10 |
| | Resolution | 4 [cm-1] |
| | Apodization | Happ-Genzel |

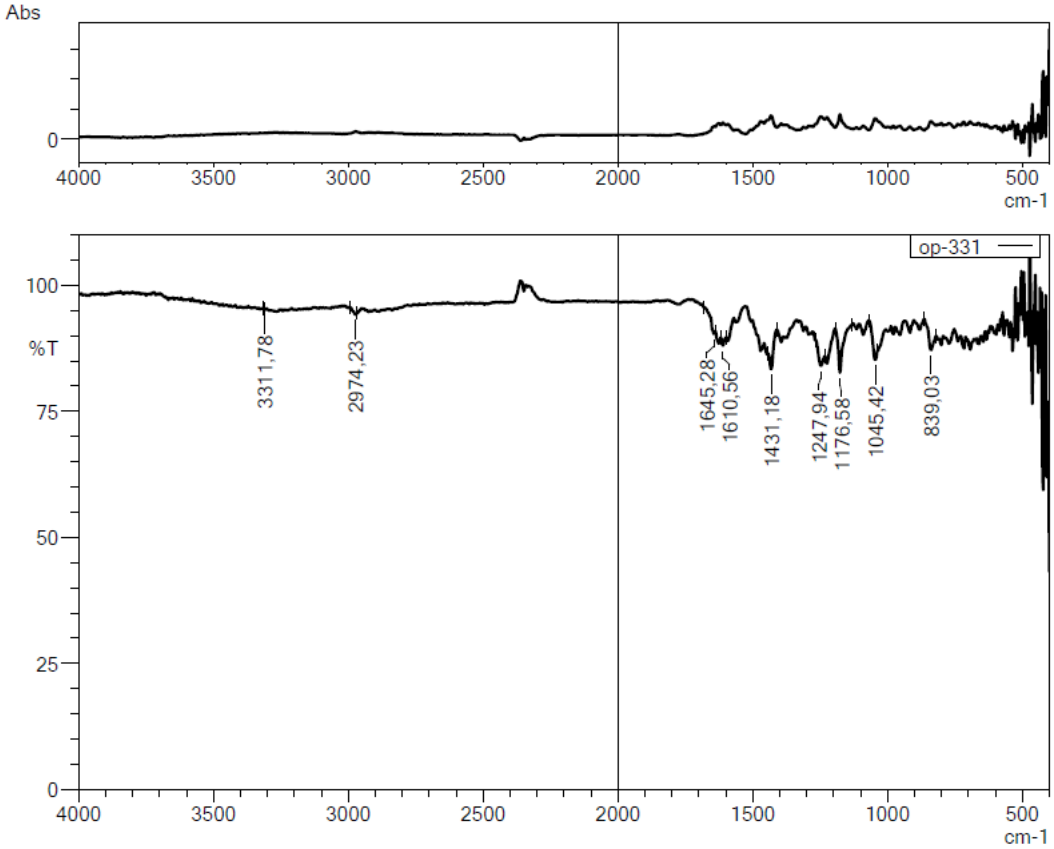


**Figure 29.** IR spectrum of compound **5b**

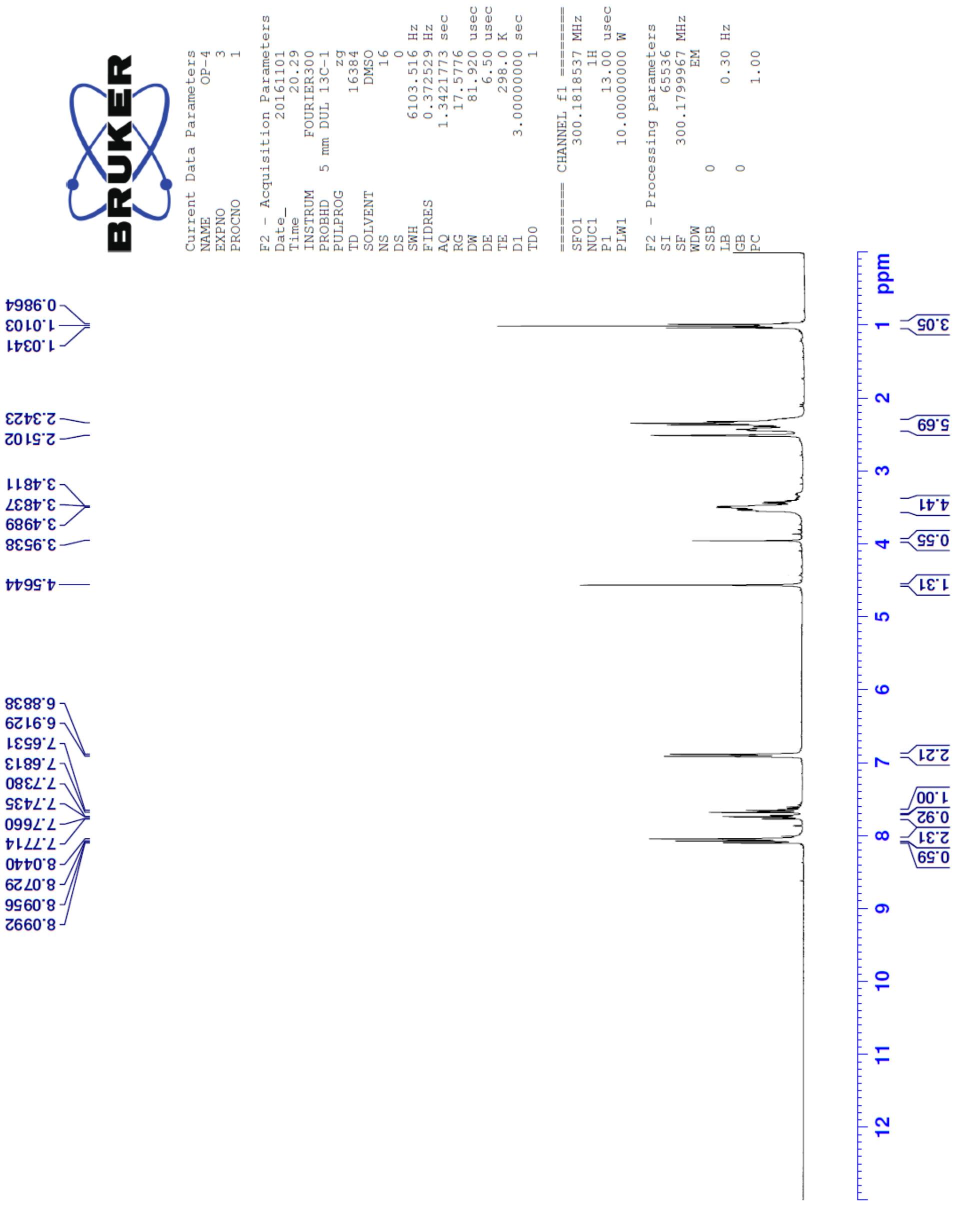


**Figure 30.** $^{1}$H-NMR spectra of compound **5b**

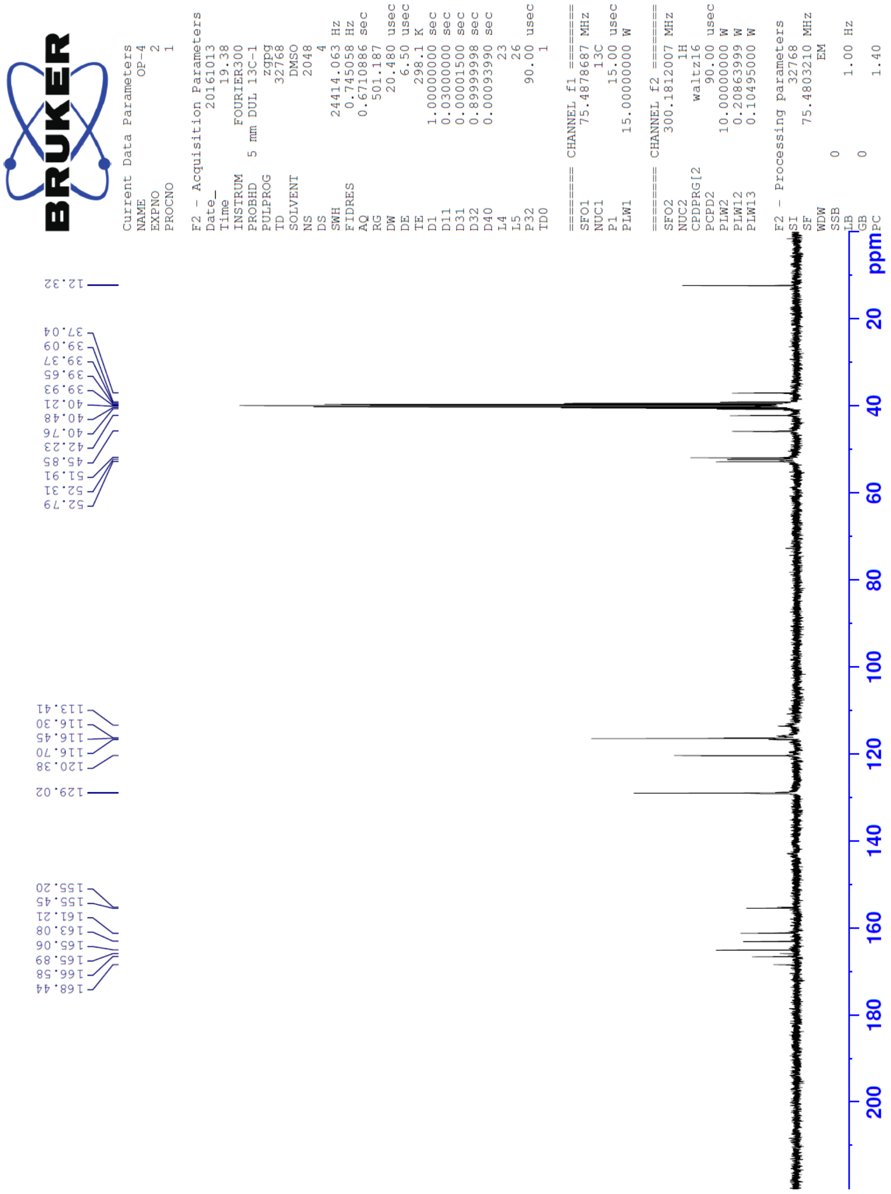


**Figure 31.** $^{13}$C-NMR spectra of compound **5b**

Data File: C:\LabSolutions\Data\Analiz\uac\OP-4_5.lcd

| Elmt | Val. | Min | Max | Elmt | Val. | Min | Max | Elmt | Val. | Min | Max | Elmt | Val. | Min | Max | Use Adduct |
|---|---|---|---|---|---|---|---|---|---|---|---|---|---|---|---|---|
| H | 1 | 20 | 40 | O | 2 | 1 | 5 | S | 2 | 1 | 3 | Ru | 2 | 0 | 0 | H |
| C | 4 | 15 | 30 | F | 1 | 0 | 1 | Cl | 1 | 0 | 1 | Pd | 2 | 0 | 0 | |
| N | 3 | 6 | 6 | P | 3 | 0 | 0 | Br | 1 | 0 | 1 | I | 3 | 0 | 0 | |

Error Margin (ppm): 5
HC Ratio: unlimited
Max Isotopes: 3
MSn Iso RI (%): 10.00

DBE Range: 12.0 - 20.0
Apply N Rule: yes
Isotope RI (%): 1.00
MSn Logic Mode: AND

Electron Ions: both
Use MSn Info: yes
Isotope Res: 9000
Max Results: 500

Event#: 1 MS(E+) Ret. Time : 1.133 -> 1.240 Scan# : 171 -> 187

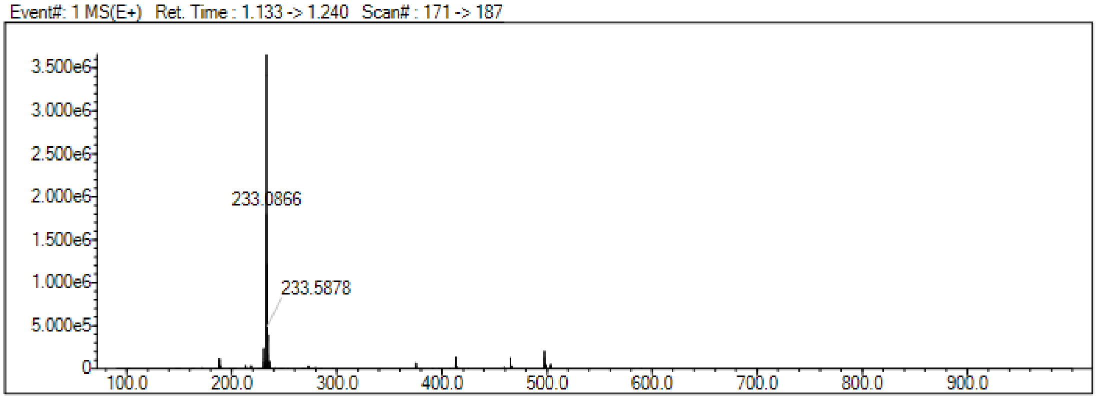


Measured region for 465.1692 m/z

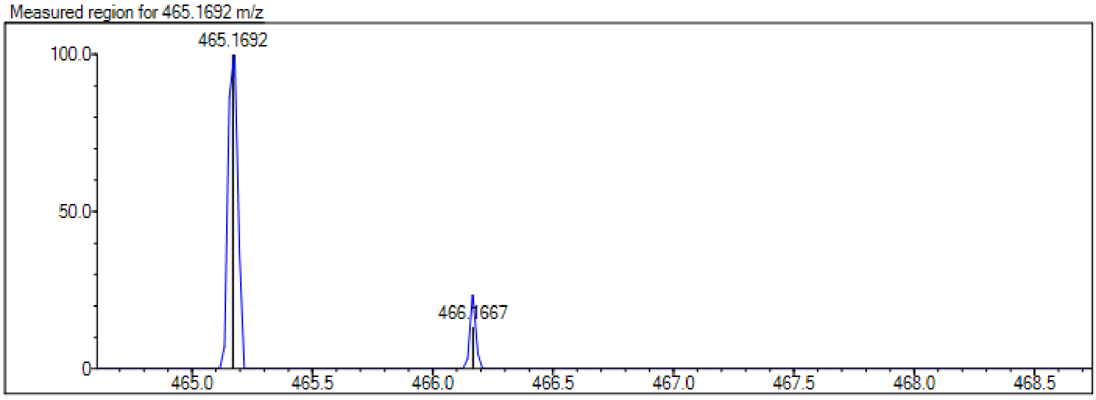


C23 H24 N6 O3 S [M+H]+ : Predicted region for 465.1703 m/z

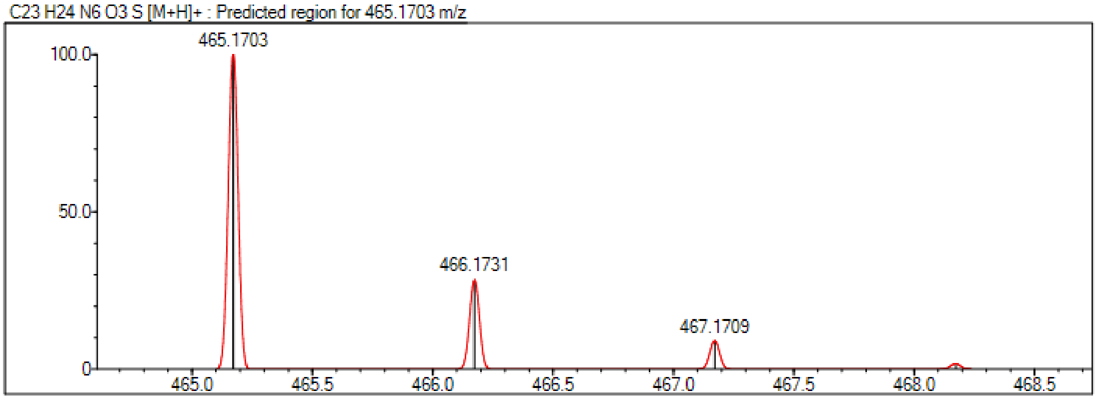


| Rank | Score | Formula (M) | Ion | Meas. m/z | Pred. m/z | Df. (mDa) | Df. (ppm) | Iso | DBE |
|---|---|---|---|---|---|---|---|---|---|
| 1 | 0.00 | C23 H24 N6 O3 S | [M+H]+ | 465.1692 | 465.1703 | -1.1 | -2.36 | 0.00 | 15.0 |

**Figure 32.** Mass spectrum of compound **5b**

DOPNALAB

| | Item | Value |
|---|---|---|
| | Acquired Date&Time | 5.02.2019 12:20:47 |
| | Acquired by | System Administrator |
| | Filename | C:\Users\dopnalab\Desktop\derya\OP\op-101.ispd |
| | Spectrum name | op-101 |
| | Sample name | OP-10 |
| | Sample ID | |
| | Option | |
| | Comment | |
| | No. of Scans | 10 |
| | Resolution | 4 [cm-1] |
| | Apodization | Happ-Genzel |

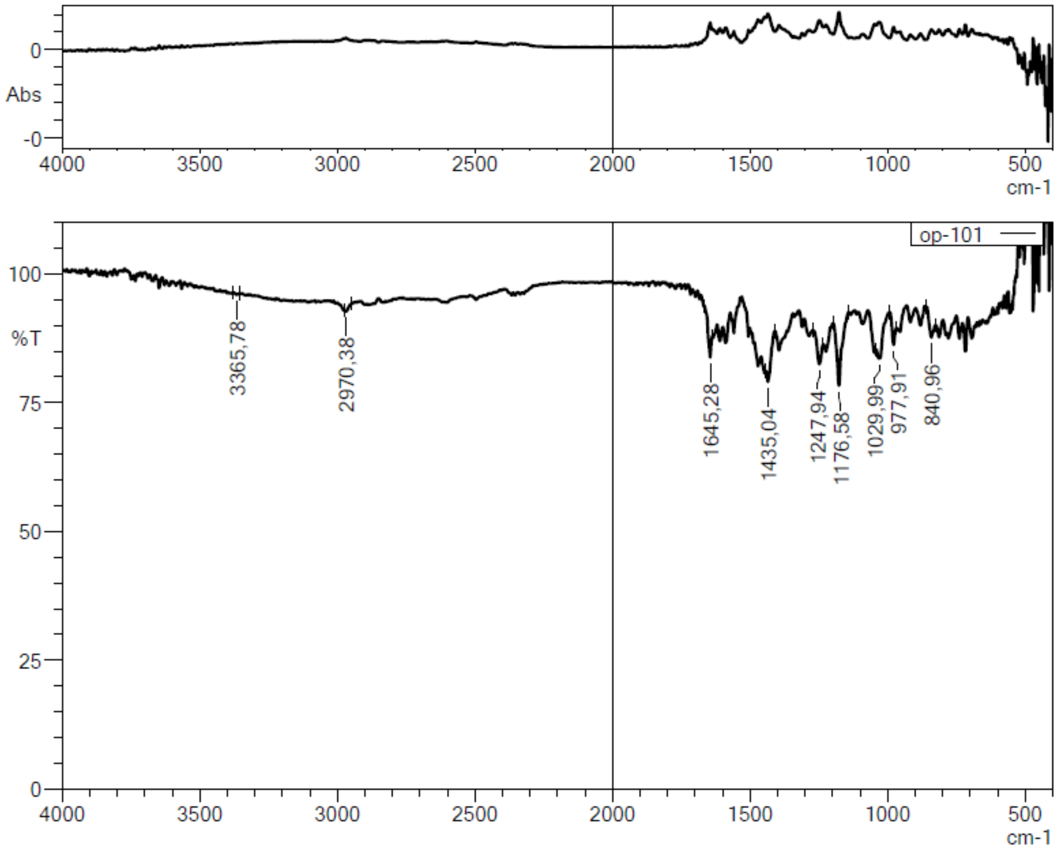


**Figure 33.** IR spectrum of compound **5c**

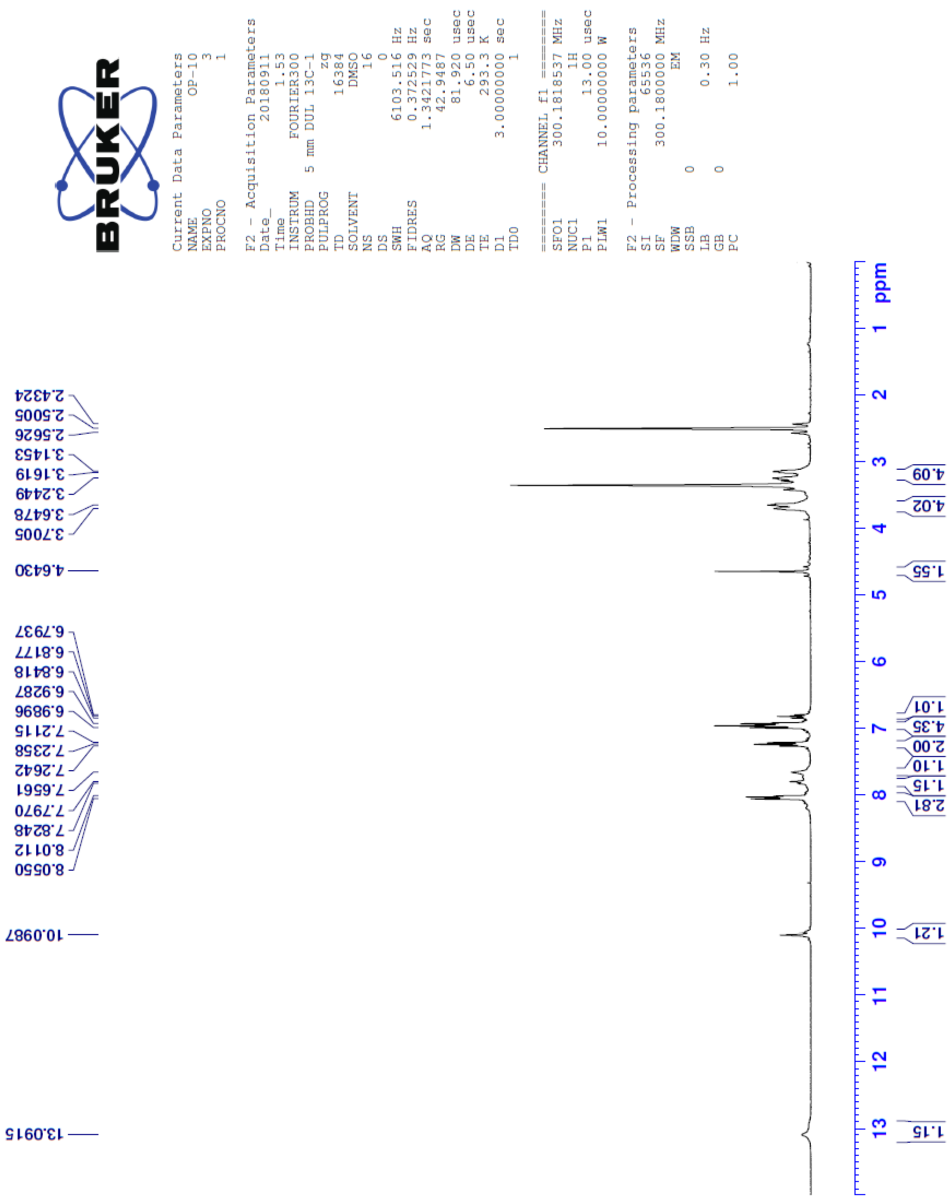


**Figure 34.** $^{1}$H-NMR spectra of compound **5c**

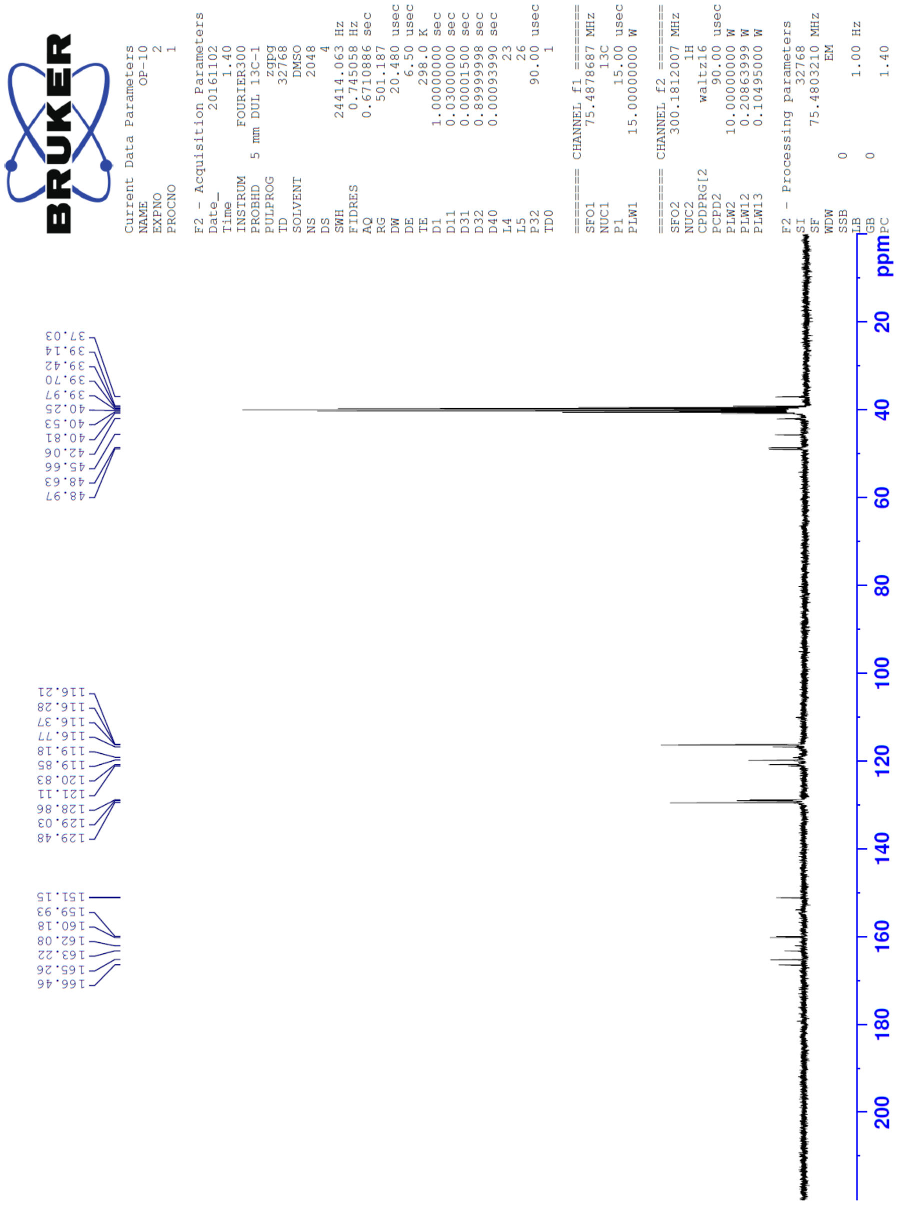

**Figure 35.** $^{13}$C-NMR spectra of compound **5c**

Formula Predictor Report - OP-10_14.lcd Page 1 of 1

Data File: C:\LabSolutions\Data\Analiz\BKaya\OP-10_14.lcd

| Elmt | Val. | Min | Max | Elmt | Val. | Min | Max | Elmt | Val. | Min | Max | Elmt | Val. | Min | Max | Use Adduct |
|---|---|---|---|---|---|---|---|---|---|---|---|---|---|---|---|---|
| H | 1 | 20 | 40 | O | 2 | 1 | 5 | S | 2 | 1 | 1 | Ru | 2 | 0 | 0 | H |
| C | 4 | 15 | 30 | F | 1 | 0 | 1 | Cl | 1 | 0 | 1 | Pd | 2 | 0 | 0 | |
| N | 3 | 6 | 8 | P | 3 | 0 | 0 | Br | 1 | 0 | 1 | I | 3 | 0 | 0 | |

Error Margin (ppm): 15
HC Ratio: unlimited
Max Isotopes: 3
MSn Iso RI (%): 10.00

DBE Range: 16.0 - 20.0
Apply N Rule: yes
Isotope RI (%): 1.00
MSn Logic Mode: AND

Electron Ions: both
Use MSn Info: yes
Isotope Res: 9000
Max Results: 500

Event#: 1 MS(E+) Ret. Time : 4.840 Scan# : 727

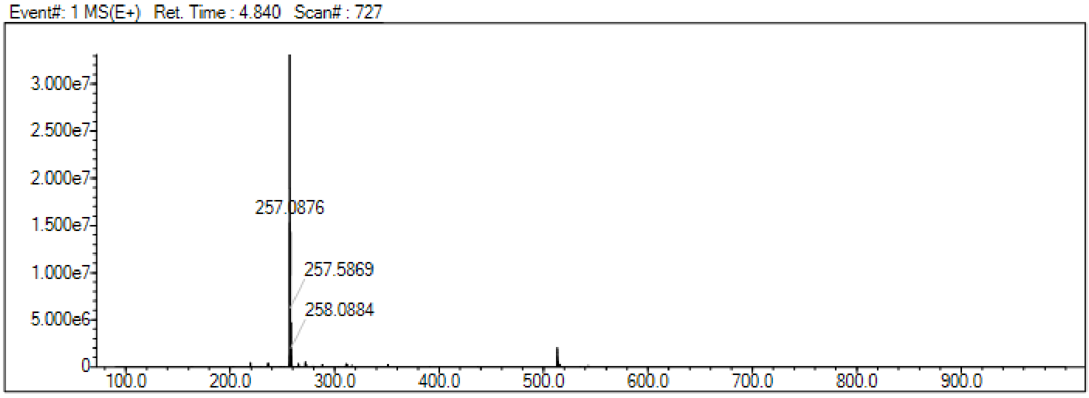


Measured region for 513.1699 m/z

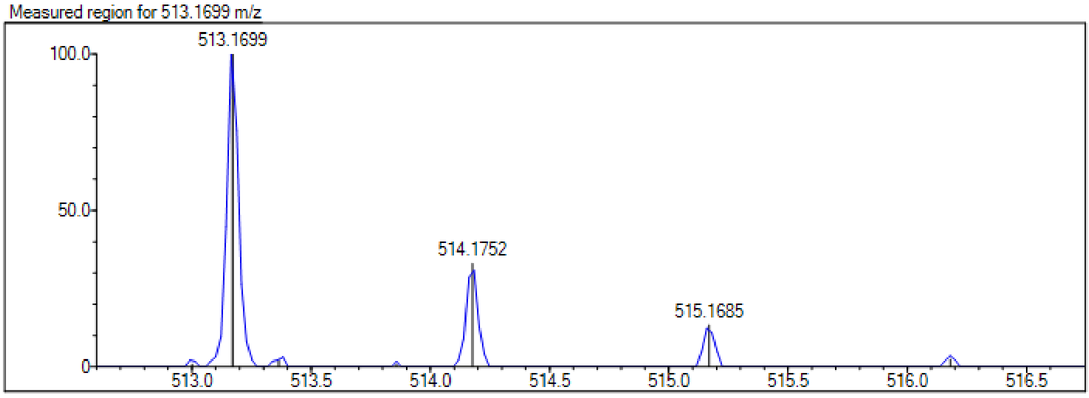


C27 H24 N6 O3 S [M+H]+ : Predicted region for 513.1703 m/z

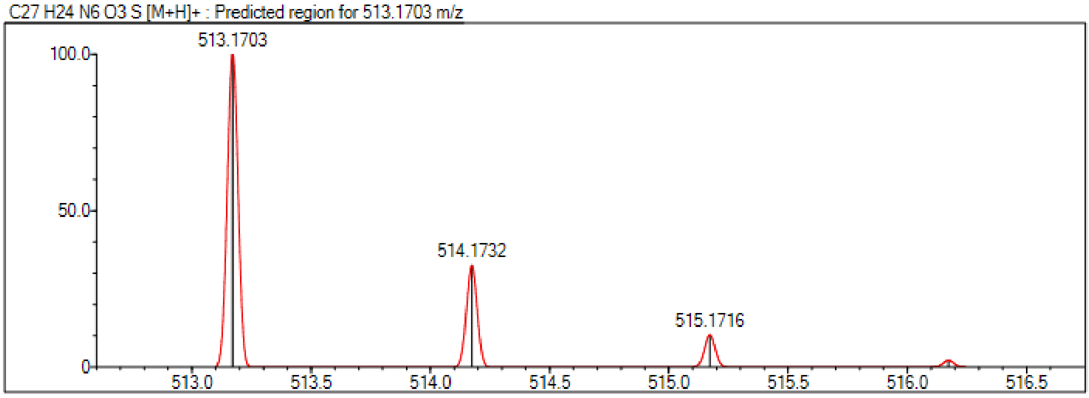


| Rank | Score | Formula (M) | Ion | Meas. m/z | Pred. m/z | Df. (mDa) | Df. (ppm) | Iso | DBE |
|---|---|---|---|---|---|---|---|---|---|
| 1 | 77.73 | C27 H24 N6 O3 S | [M+H]+ | 513.1699 | 513.1703 | -0.4 | -0.78 | 77.73 | 19.0 |

**Figure 36.** Mass spectrum of compound **5c**

DOPNALAB

| | Item | Value |
|---|---|---|
| | Acquired Date&Time | 5.02.2019 11:41:35 |
| | Acquired by | System Administrator |
| | Filename | C:\Users\dopnalab\Desktop\derya\OP\OP-61.ispd |
| | Spectrum name | OP-61 |
| | Sample name | OP-6 |
| | Sample ID | |
| | Option | |
| | Comment | |
| | No. of Scans | 10 |
| | Resolution | 4 [cm-1] |
| | Apodization | Happ-Genzel |

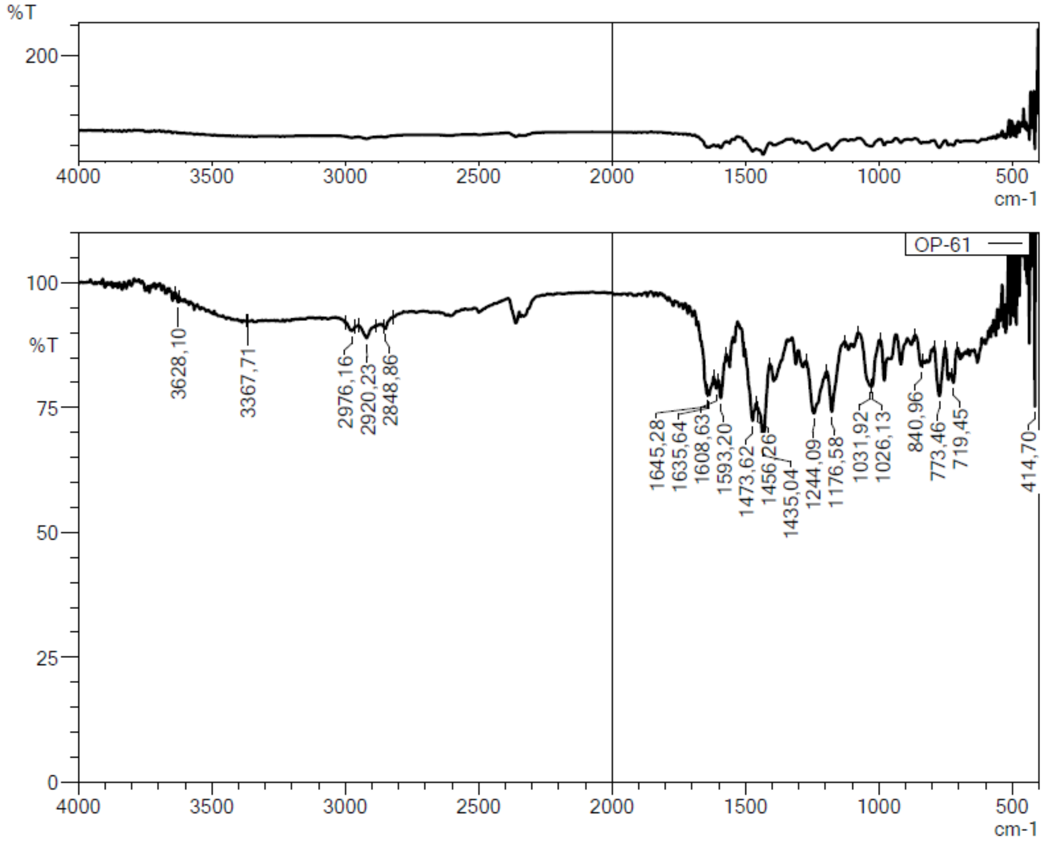


**Figure 37.** IR spectrum of compound **5d**

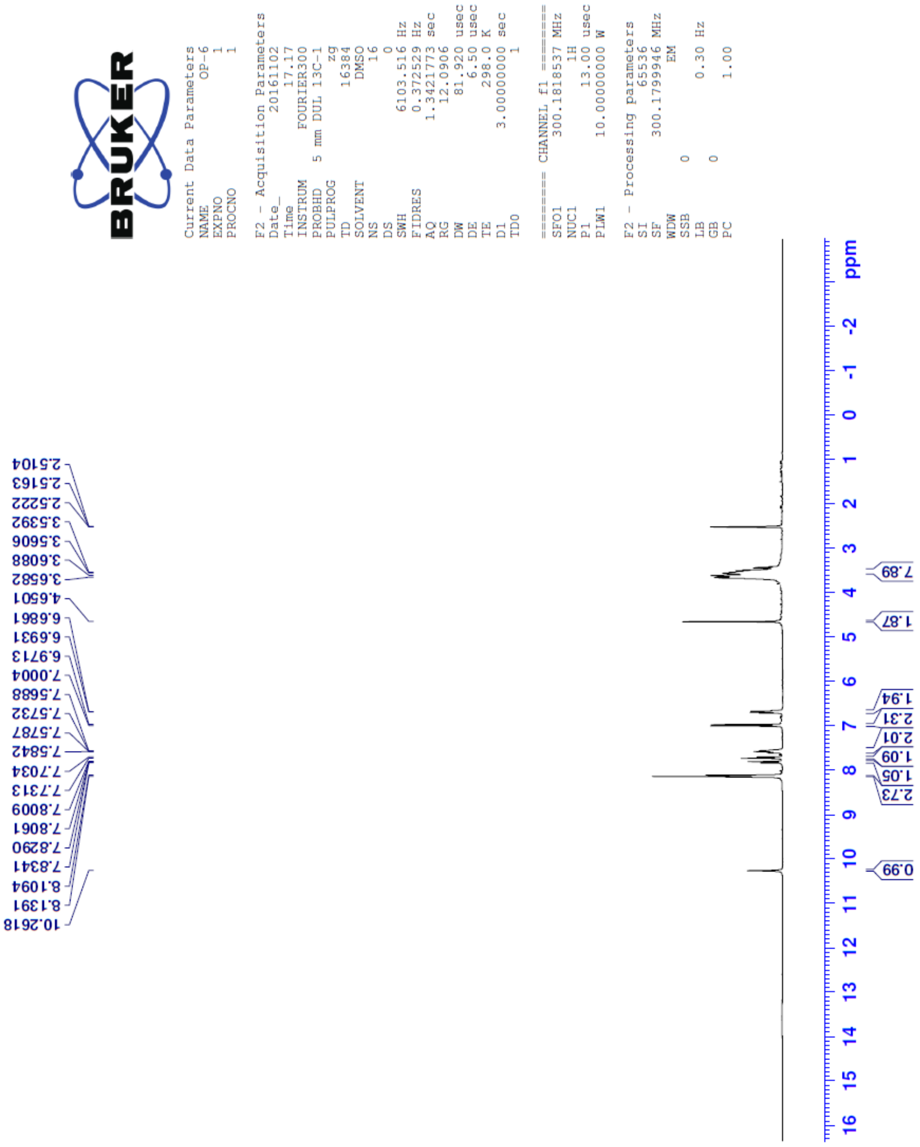

**Figure 38.** $^{1}$H-NMR spectra of compound **5d**

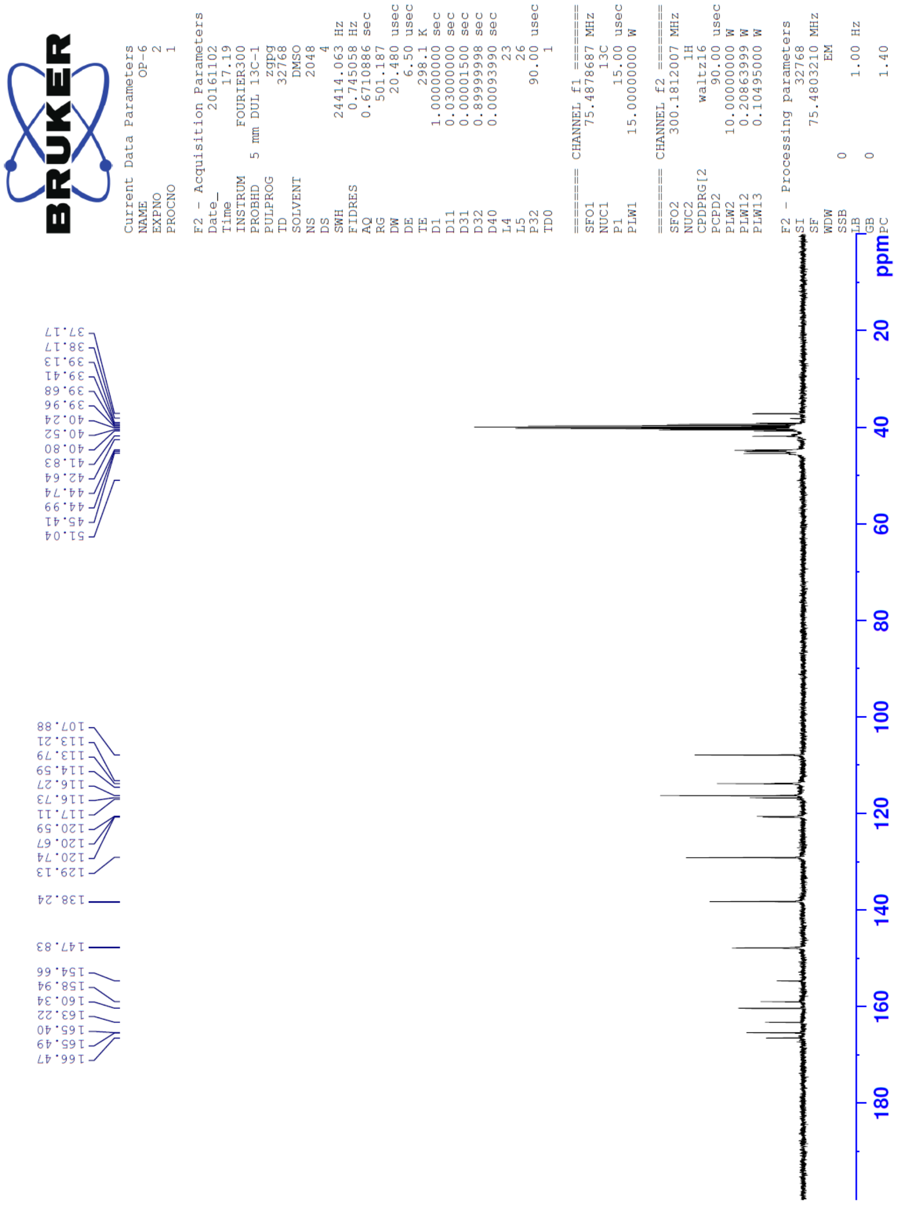


**Figure 39.** $^{13}$C-NMR spectra of compound **5d**

Data File: C:\LabSolutions\Data\Analiz\uac\OP-6_5.lcd

| Elmt | Val. | Min | Max | Elmt | Val. | Min | Max | Elmt | Val. | Min | Max | Elmt | Val. | Min | Max | Use Adduct |
|---|---|---|---|---|---|---|---|---|---|---|---|---|---|---|---|---|
| H | 1 | 20 | 40 | O | 2 | 1 | 5 | S | 2 | 1 | 3 | Ru | 2 | 0 | 0 | H |
| C | 4 | 15 | 30 | F | 1 | 0 | 1 | Cl | 1 | 0 | 1 | Pd | 2 | 0 | 0 | |
| N | 3 | 6 | 7 | P | 3 | 0 | 0 | Br | 1 | 0 | 1 | I | 3 | 0 | 0 | |

| | | | | | |
|---|---|---|---|---|---|
| Error Margin (ppm): | 5 | DBE Range: | 12.0 - 20.0 | Electron Ions: | both |
| HC Ratio: | unlimited | Apply N Rule: | yes | Use MSn Info: | yes |
| Max Isotopes: | 3 | Isotope RI (%): | 1.00 | Isotope Res: | 9000 |
| MSn Iso RI (%): | 10.00 | MSn Logic Mode: | AND | Max Results: | 500 |

Event#: 1 MS(E+) Ret. Time : 2.560 -> 2.560 Scan# : 385 -> 385

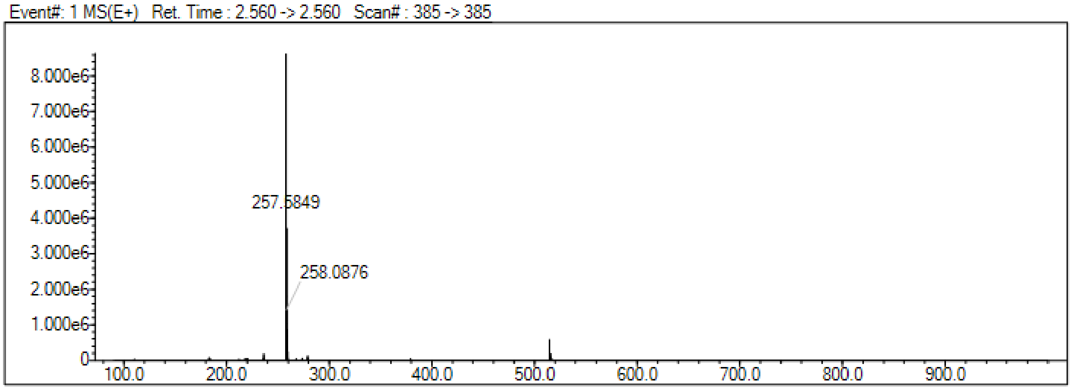


Measured region for 514.1636 m/z

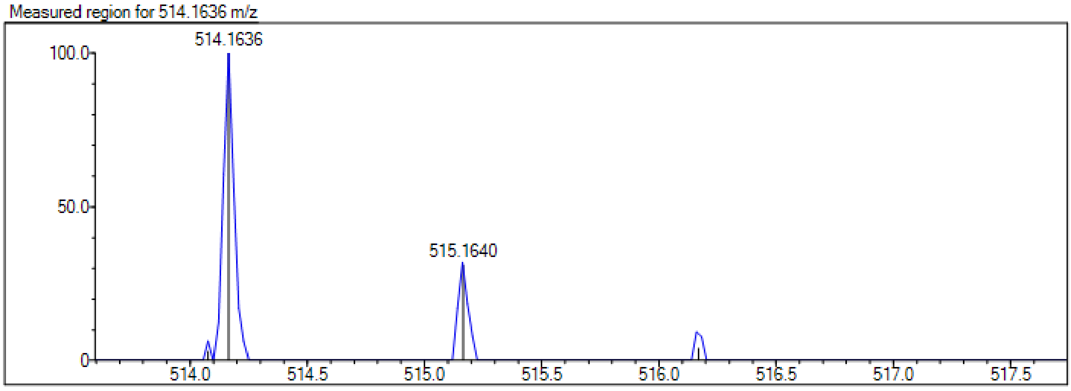


C26 H23 N7 O3 S [M+H]+ : Predicted region for 514.1656 m/z

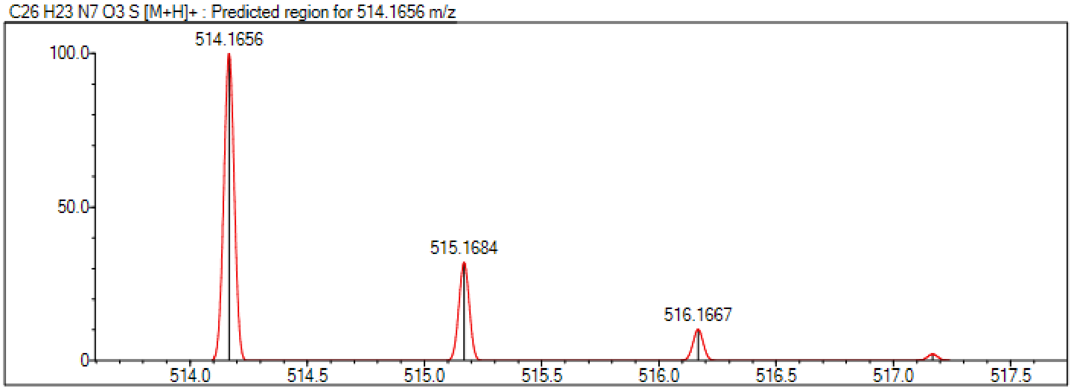


| Rank | Score | Formula (M) | Ion | Meas. m/z | Pred. m/z | Df. (mDa) | Df. (ppm) | Iso | DBE |
|---|---|---|---|---|---|---|---|---|---|
| 1 | 72.50 | C26 H23 N7 O3 S | [M+H]+ | 514.1636 | 514.1656 | -2.0 | -3.89 | 78.14 | 19.0 |

**Figure 40.** Mass spectrum of compound **5d**

DOPNALAB

| | Item | Value |
|---|---|---|
| | Acquired Date&Time | 5.02.2019 12:02:18 |
| | Acquired by | System Administrator |
| | Filename | C:\Users\dopnalab\Desktop\derya\OP\op-7b2.ispd |
| | Spectrum name | op-7b2 |
| | Sample name | OP-7B |
| | Sample ID | |
| | Option | |
| | Comment | |
| | No. of Scans | 10 |
| | Resolution | 4 [cm-1] |
| | Apodization | Happ-Genzel |

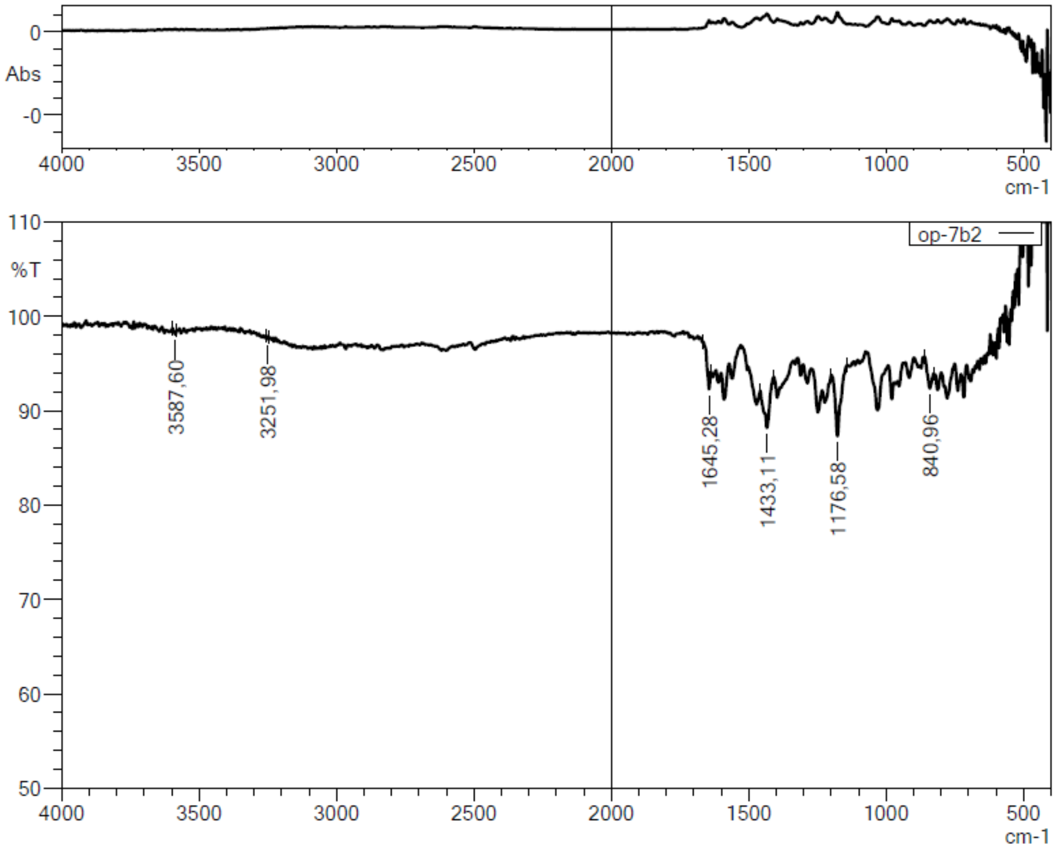


**Figure 41.** IR spectrum of compound **5e**

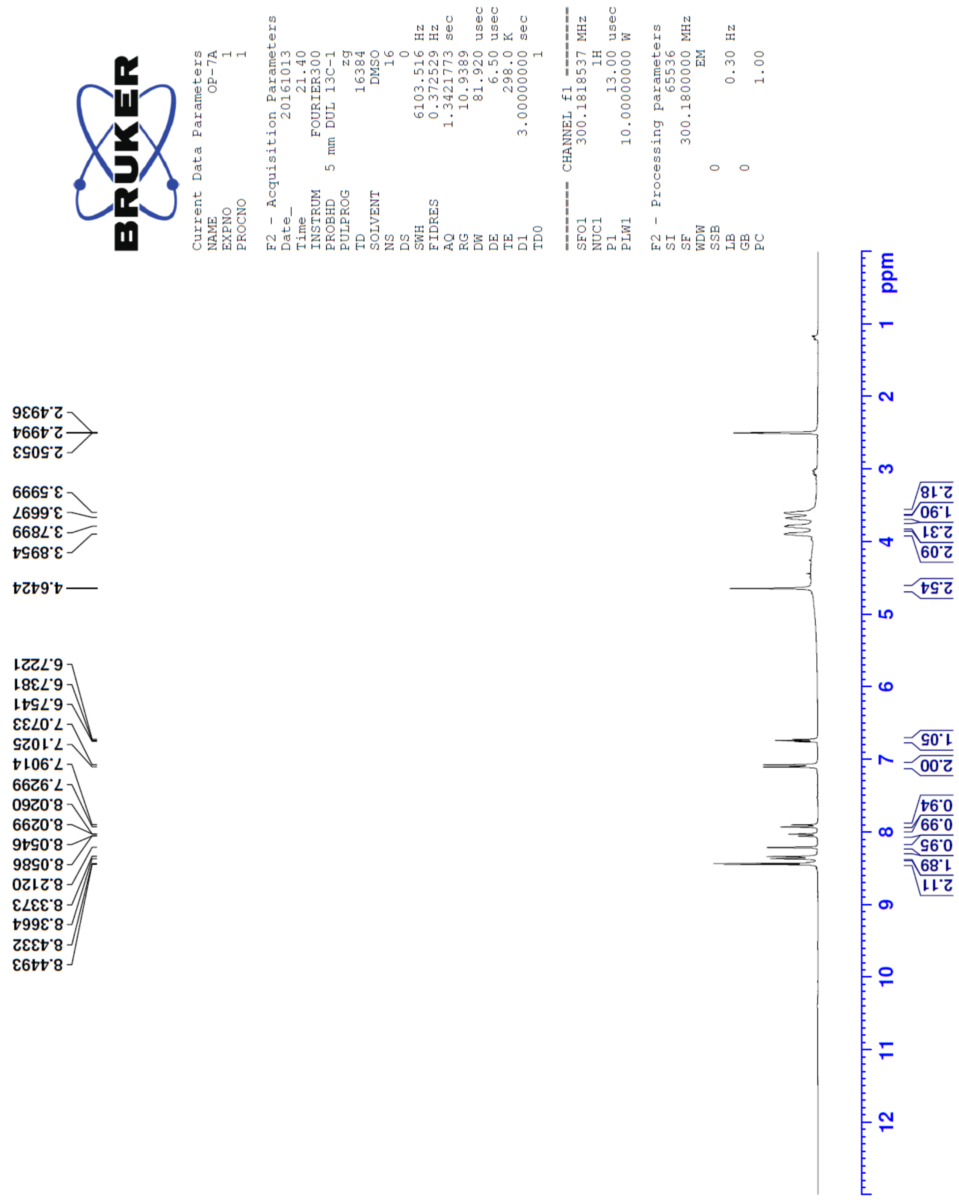


**Figure 42.** $^{1}$H-NMR spectra of compound **5e**

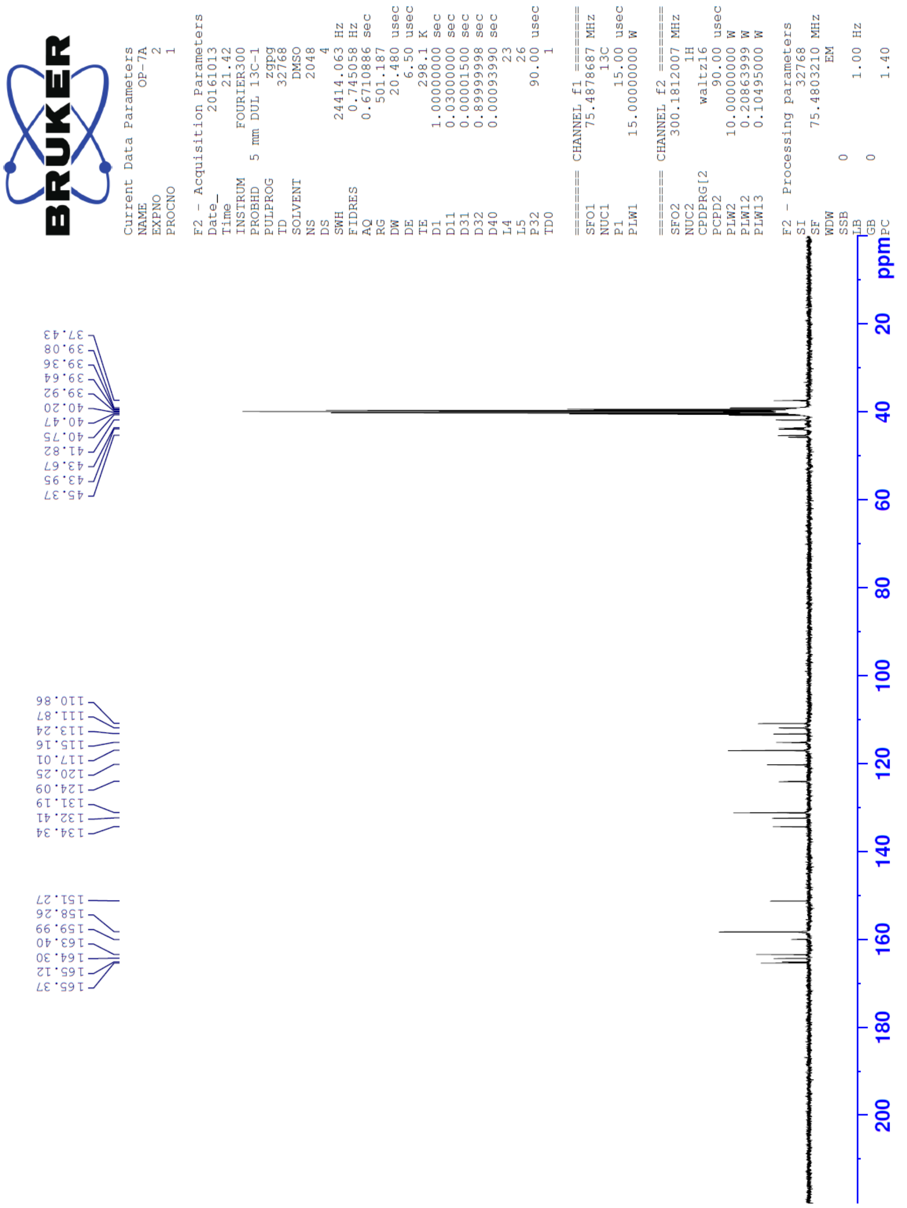


**Figure 43.** $^{13}$C-NMR spectra of compound **5e**

Data File: C:\LabSolutions\Data\Analiz\uac\OP-7_3.lcd

| Elmt | Val. | Min | Max | Elmt | Val. | Min | Max | Elmt | Val. | Min | Max | Elmt | Val. | Min | Max | Use Adduct |
|---|---|---|---|---|---|---|---|---|---|---|---|---|---|---|---|---|
| H | 1 | 20 | 40 | O | 2 | 1 | 5 | S | 2 | 1 | 3 | Ru | 2 | 0 | 0 | H |
| C | 4 | 15 | 30 | F | 1 | 0 | 0 | Cl | 1 | 0 | 0 | Pd | 2 | 0 | 0 | |
| N | 3 | 6 | 8 | P | 3 | 0 | 0 | Br | 1 | 0 | 1 | I | 3 | 0 | 0 | |

Error Margin (ppm): 5
HC Ratio: unlimited
Max Isotopes: 3
MSn Iso RI (%): 10.00

DBE Range: 12.0 - 20.0
Apply N Rule: yes
Isotope RI (%): 1.00
MSn Logic Mode: AND

Electron Ions: both
Use MSn Info: yes
Isotope Res: 9000
Max Results: 500

Event#: 1 MS(E+) Ret. Time : 4.253 -> 4.253 Scan# : 639 -> 639

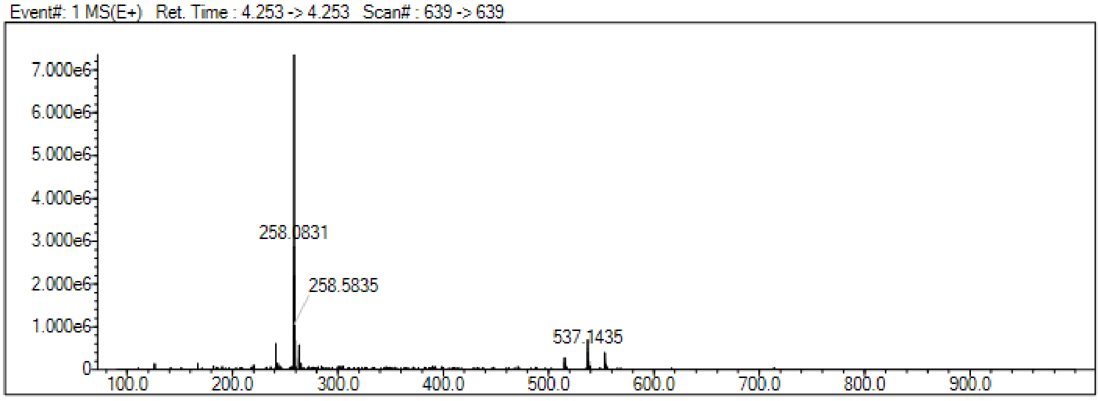


Measured region for 515.1610 m/z

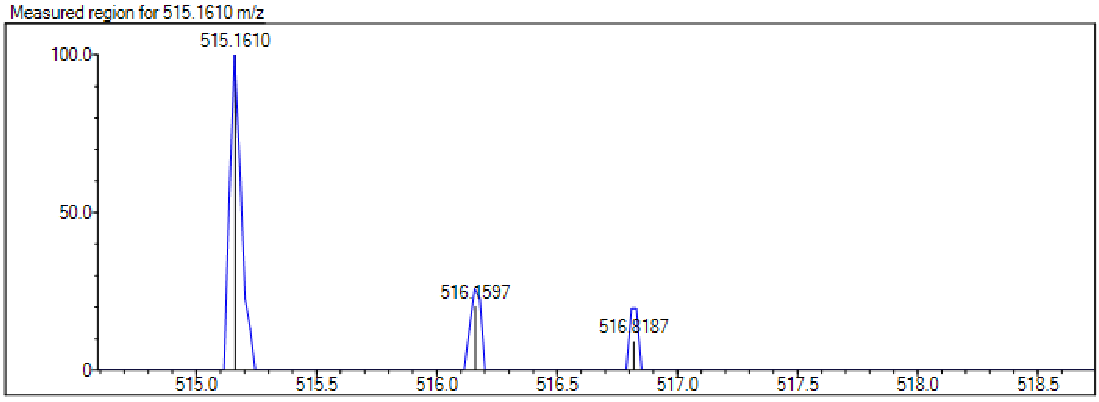


C25 H22 N8 O3 S [M+H]+ : Predicted region for 515.1608 m/z

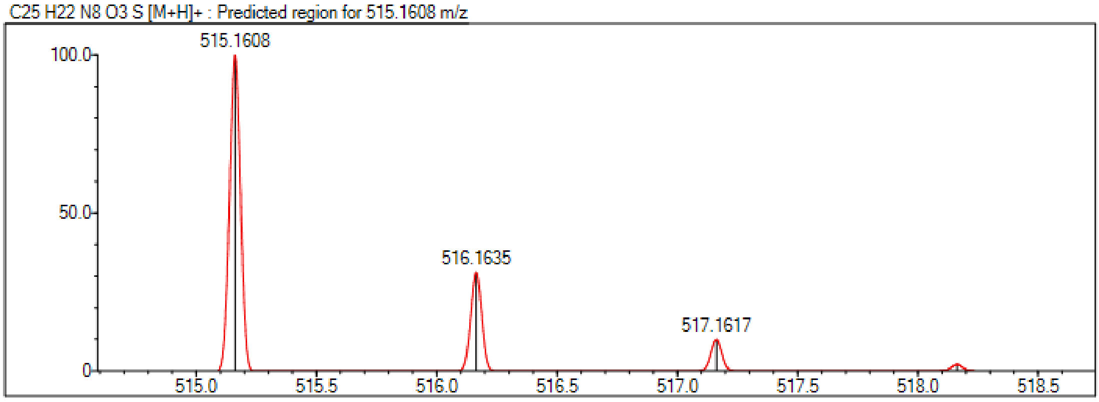


| Rank | Score | Formula (M) | Ion | Meas. m/z | Pred. m/z | Df. (mDa) | Df. (ppm) | Iso | DBE |
|---|---|---|---|---|---|---|---|---|---|
| 1 | 0.00 | C25 H22 N8 O3 S | [M+H]+ | 515.1610 | 515.1608 | 0.2 | 0.39 | 0.00 | 19.0 |

**Figure 44.** Mass spectrum of compound **5e**

DOPNALAB

| | Item | Value |
|---|---|---|
| | Acquired Date&Time | 5.02.2019 12:16:02 |
| | Acquired by | System Administrator |
| | Filename | C:\Users\dopnalab\Desktop\derya\OP\op-191.ispd |
| | Spectrum name | op-191 |
| | Sample name | OP-19 |
| | Sample ID | |
| | Option | |
| | Comment | |
| | No. of Scans | 10 |
| | Resolution | 4 [cm-1] |
| | Apodization | Happ-Genzel |

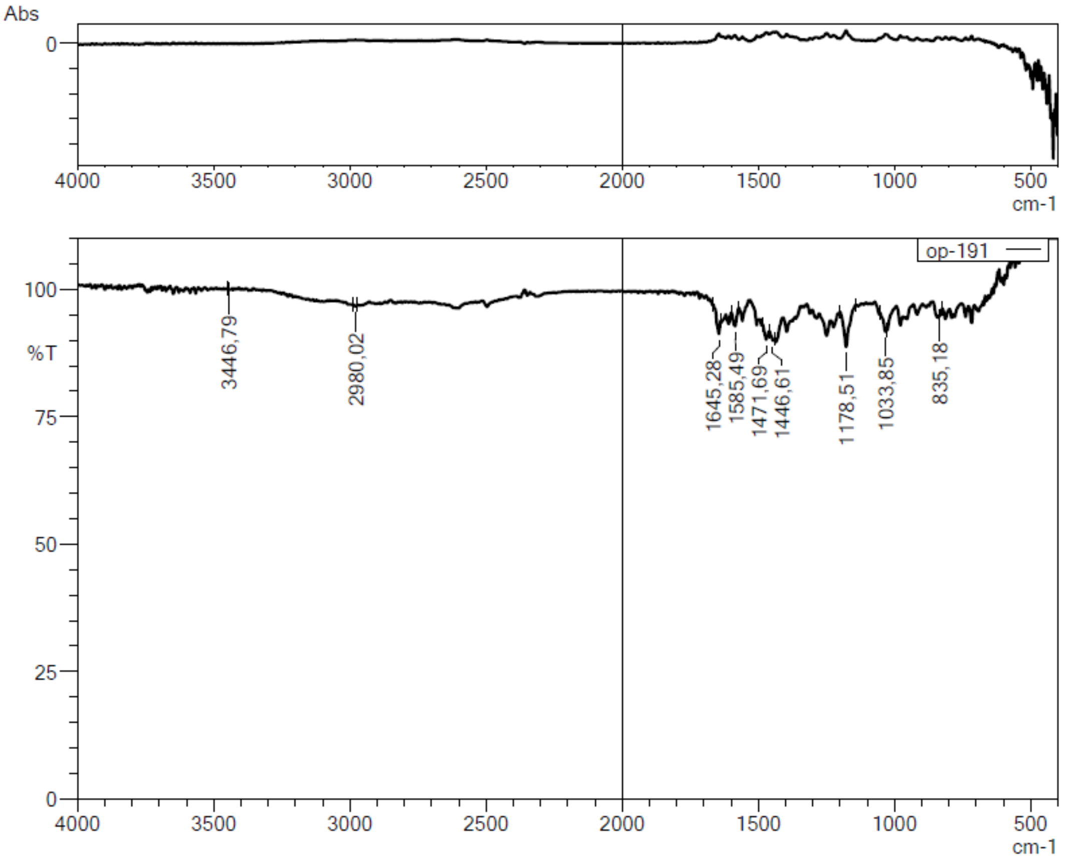


**Figure 45.** IR spectrum of compound **5f**

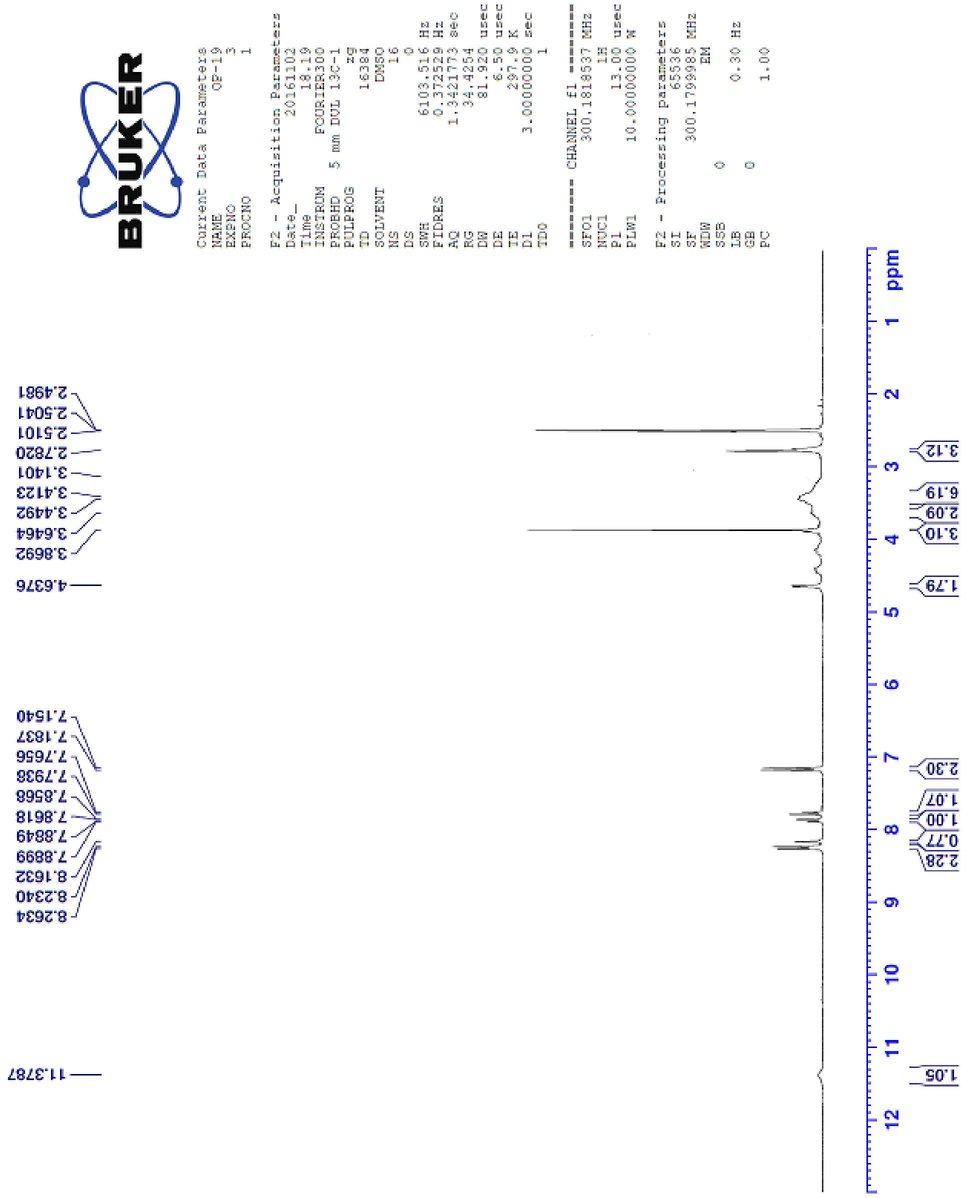

**Figure 46.** $^{1}$H-NMR spectra of compound **5f**

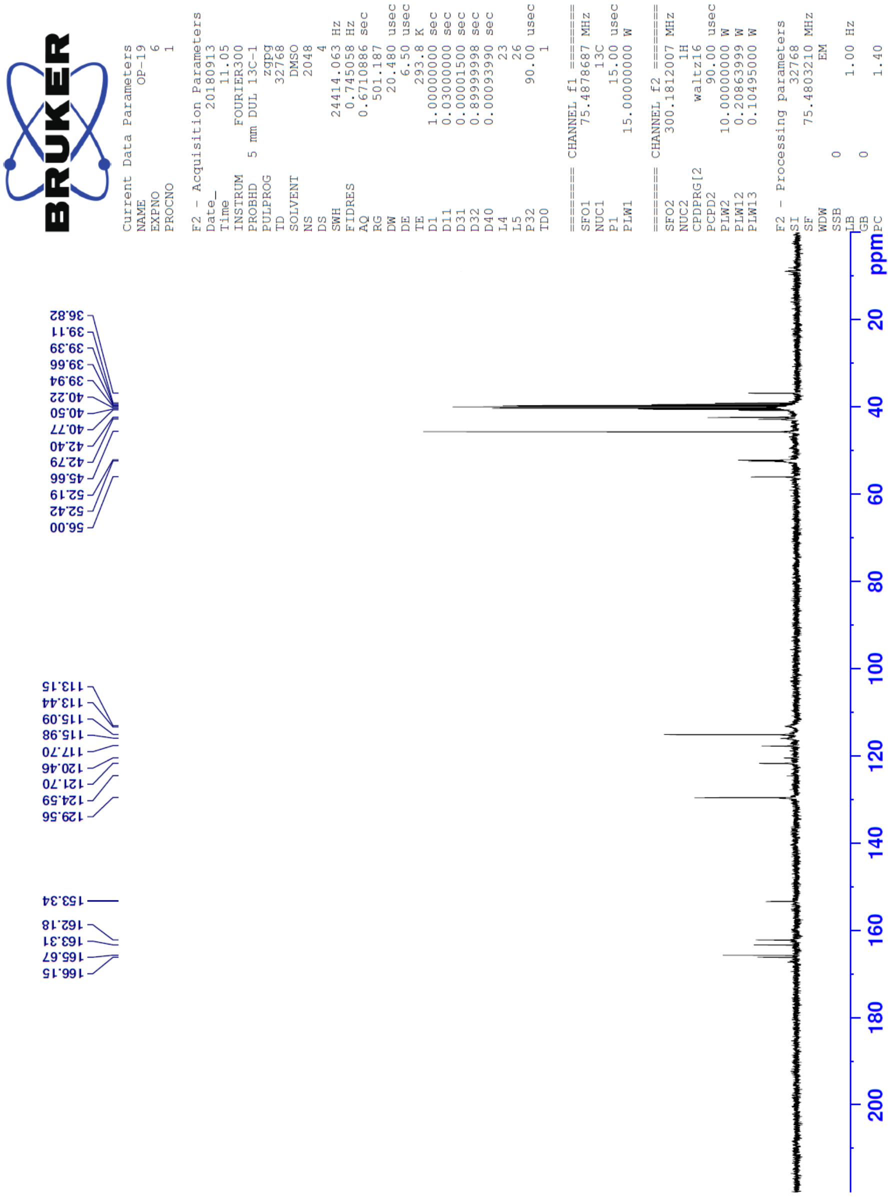

**Figure 47.** $^{13}$C-NMR spectra of compound **5f**

Data File: C:\LabSolutions\Data\Analiz\uac\OP-19_7.lcd

| Elmt | Val. | Min | Max | Elmt | Val. | Min | Max | Elmt | Val. | Min | Max | Elmt | Val. | Min | Max | Use Adduct |
|---|---|---|---|---|---|---|---|---|---|---|---|---|---|---|---|---|
| H | 1 | 20 | 40 | O | 2 | 1 | 5 | S | 2 | 1 | 1 | Ru | 2 | 0 | 0 | H |
| C | 4 | 15 | 30 | F | 1 | 0 | 0 | Cl | 1 | 0 | 1 | Pd | 2 | 0 | 0 | |
| N | 3 | 6 | 8 | P | 3 | 0 | 0 | Br | 1 | 0 | 1 | I | 3 | 0 | 0 | |

Error Margin (ppm): 5
HC Ratio: unlimited
Max Isotopes: 3
MSn Iso RI (%): 10.00

DBE Range: 12.0 - 20.0
Apply N Rule: yes
Isotope RI (%): 1.00
MSn Logic Mode: AND

Electron Ions: both
Use MSn Info: yes
Isotope Res: 9000
Max Results: 500

Event#: 1 MS(E+) Ret. Time : 2.893 -> 2.893 Scan# : 435 -> 435

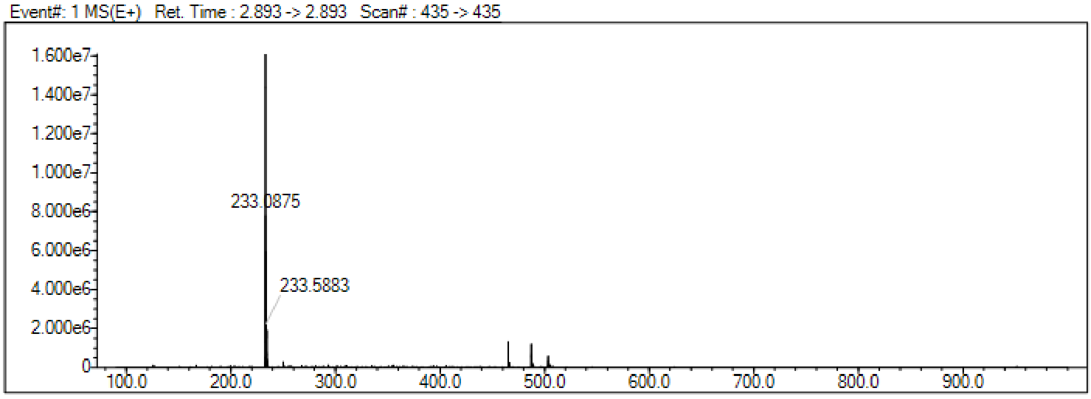


Measured region for 465.1703 m/z

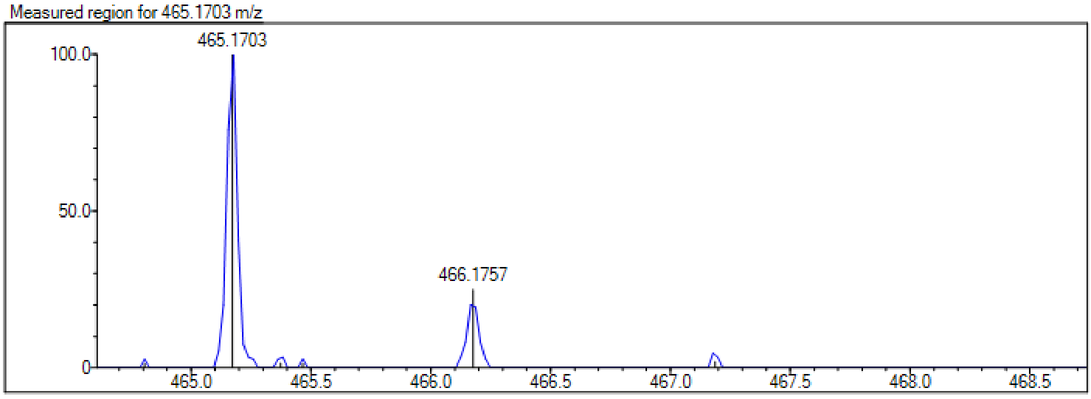


C23 H24 N6 O3 S [M+H]+ : Predicted region for 465.1703 m/z

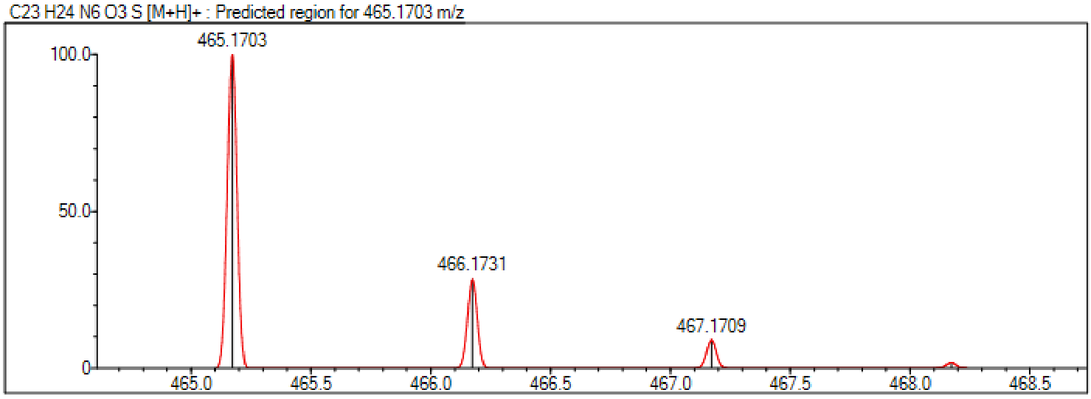


| Rank | Score | Formula (M) | Ion | Meas. m/z | Pred. m/z | Df. (mDa) | Df. (ppm) | Iso | DBE |
|---|---|---|---|---|---|---|---|---|---|
| 1 | 41.53 | C23 H24 N6 O3 S | [M+H]+ | 465.1703 | 465.1703 | -0.0 | 0.00 | 41.53 | 15.0 |

**Figure 48.** Mass spectrum of compound **5f**

## DOPNALAB

| | Item | Value |
|---|---|---|
| | Acquired Date&Time | 5.02.2019 12:18:12 |
| | Acquired by | System Administrator |
| | Filename | C:\Users\dopnalab\Desktop\derya\OP\op-181.ispd |
| | Spectrum name | op-181 |
| | Sample name | OP-18 |
| | Sample ID | |
| | Option | |
| | Comment | |
| | No. of Scans | 10 |
| | Resolution | 4 [cm-1] |
| | Apodization | Happ-Genzel |

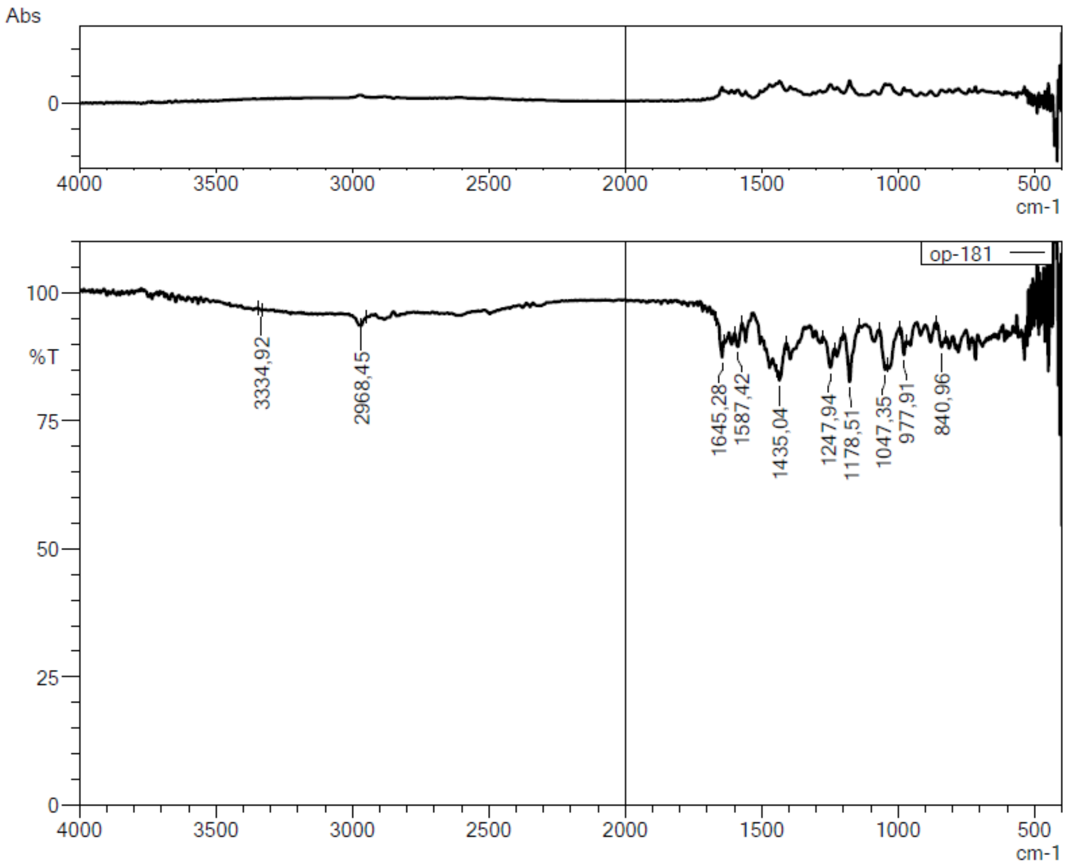


**Figure 49.** IR spectrum of compound **5g**

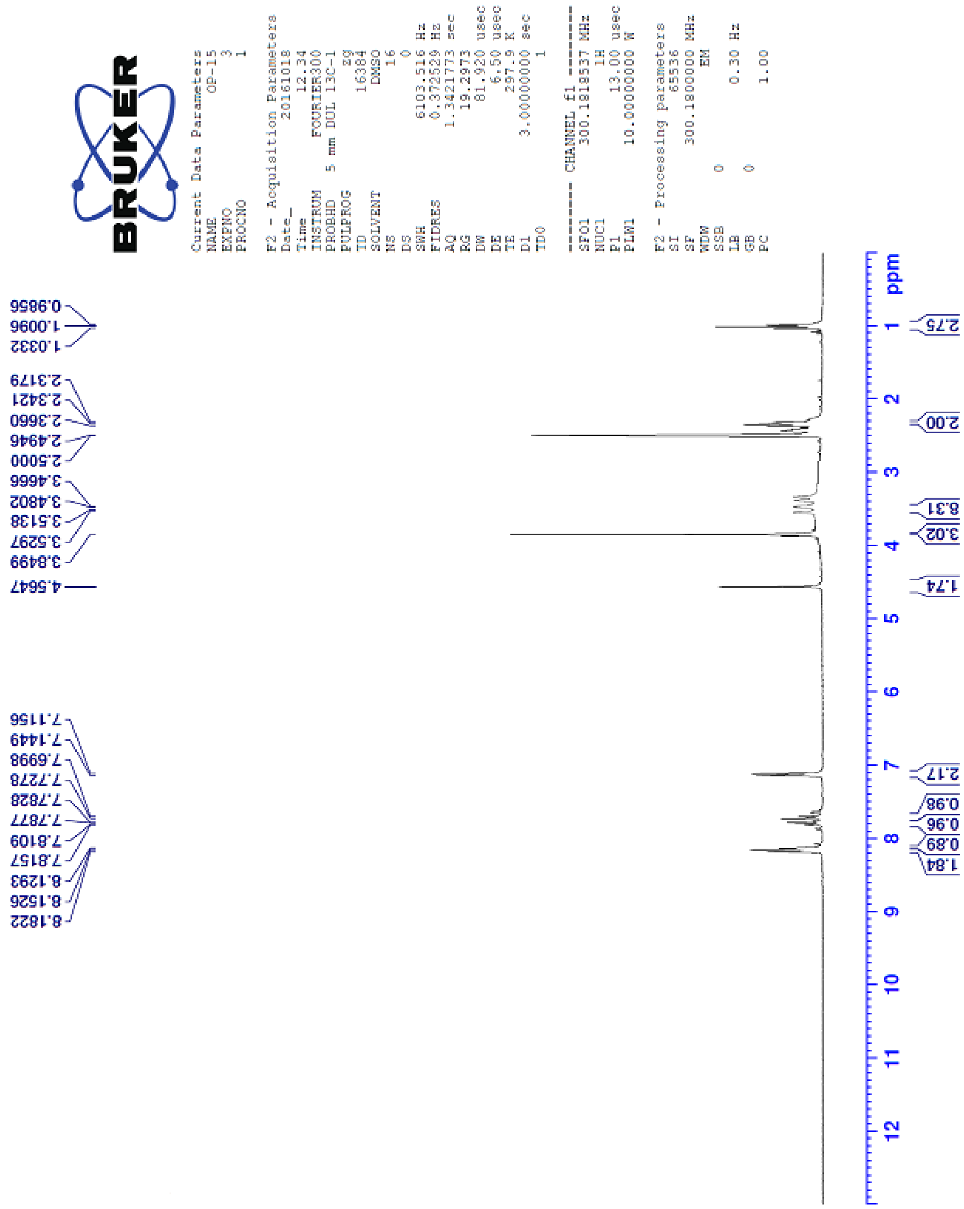

**Figure 50.** $^{1}$H-NMR spectra of compound **5g**

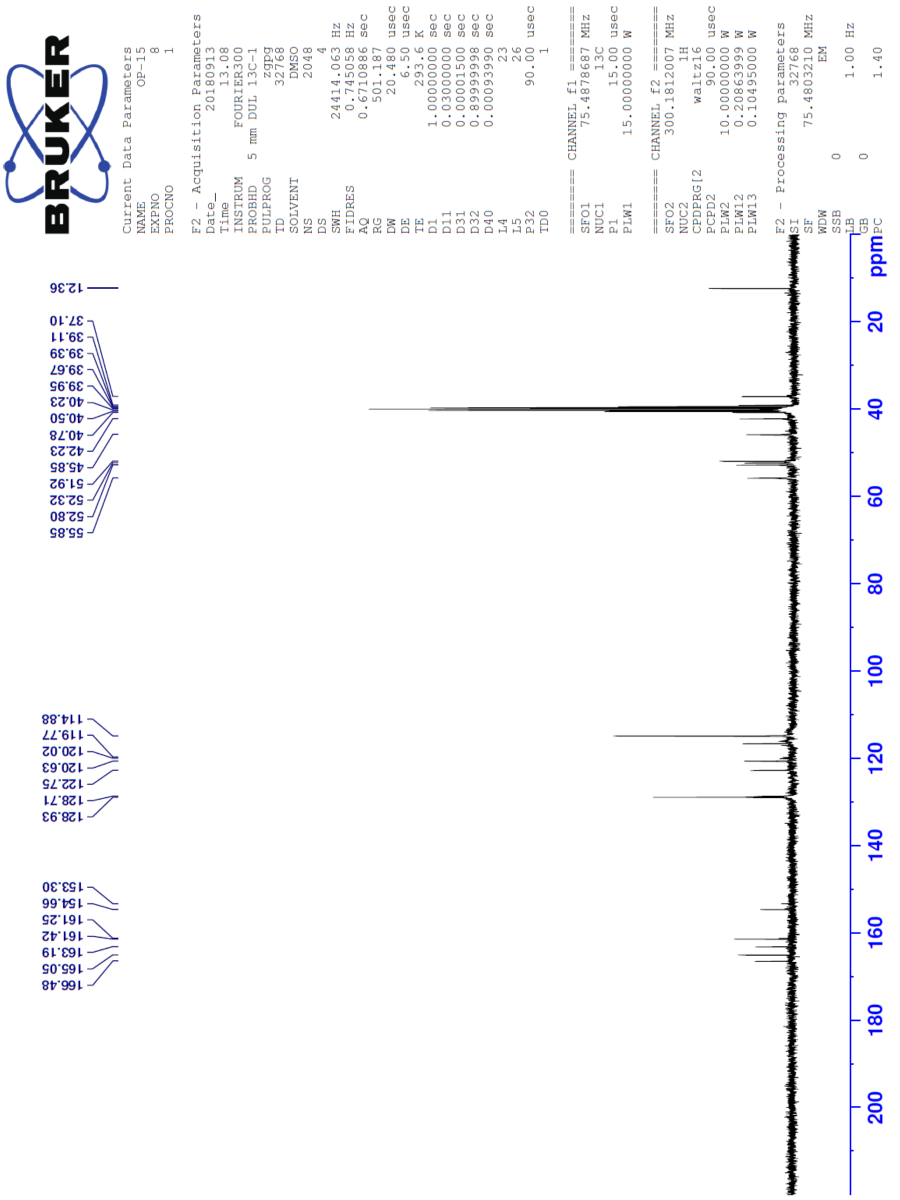


**Figure 51.** $^{13}$C-NMR spectra of compound **5g**

Data File: C:\LabSolutions\Data\Analiz\uac\OP-15_8.lcd

| Elmt | Val. | Min | Max | Elmt | Val. | Min | Max | Elmt | Val. | Min | Max | Elmt | Val. | Min | Max | Use Adduct |
|---|---|---|---|---|---|---|---|---|---|---|---|---|---|---|---|---|
| H | 1 | 20 | 40 | O | 2 | 1 | 5 | S | 2 | 1 | 3 | Ru | 2 | 0 | 0 | H |
| C | 4 | 15 | 30 | F | 1 | 0 | 0 | Cl | 1 | 0 | 1 | Pd | 2 | 0 | 0 | |
| N | 3 | 6 | 8 | P | 3 | 0 | 0 | Br | 1 | 0 | 1 | I | 3 | 0 | 0 | |

Error Margin (ppm): 5
HC Ratio: unlimited
Max Isotopes: 3
MSn Iso RI (%): 10.00

DBE Range: 12.0 - 20.0
Apply N Rule: yes
Isotope RI (%): 1.00
MSn Logic Mode: AND

Electron Ions: both
Use MSn Info: yes
Isotope Res: 9000
Max Results: 500

Event#: 1 MS(E+) Ret. Time : 2.773 Scan# : 417

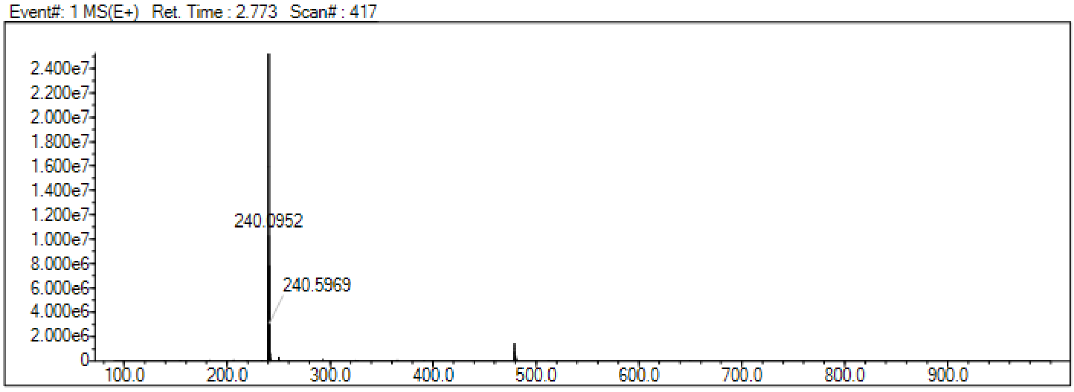


Measured region for 479.1872 m/z

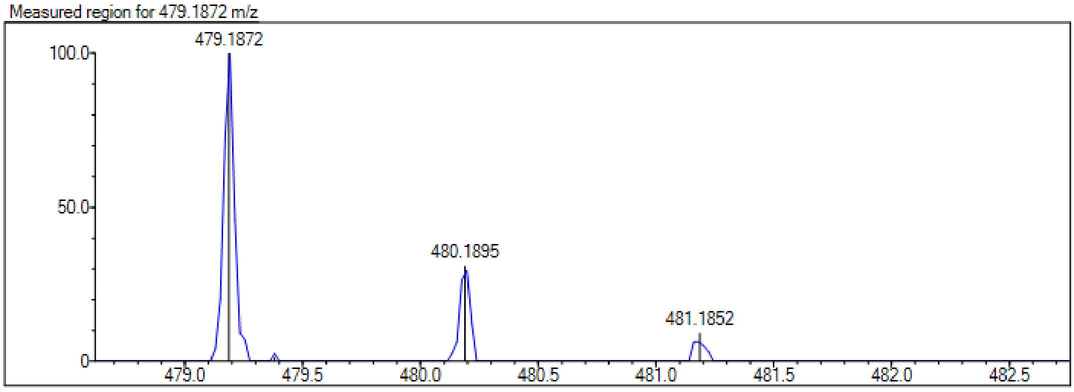


C24 H26 N6 O3 S [M+H]+ : Predicted region for 479.1860 m/z

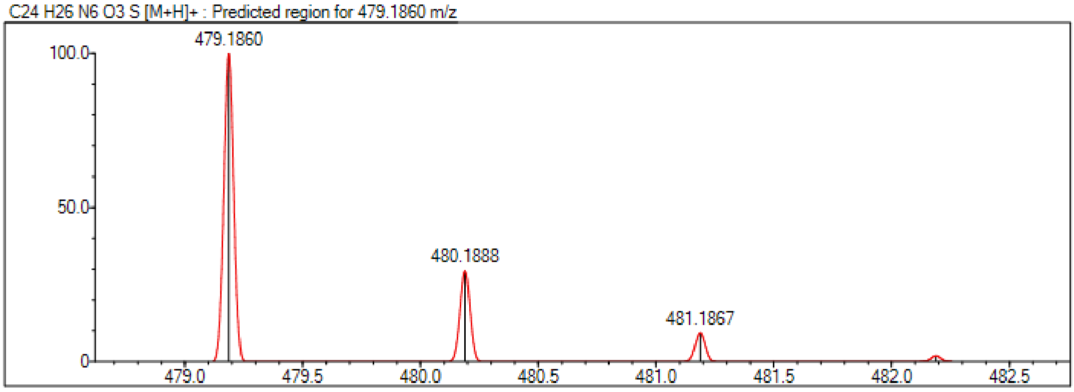


| Rank | Score | Formula (M) | Ion | Meas. m/z | Pred. m/z | Df. (mDa) | Df. (ppm) | Iso | DBE |
|---|---|---|---|---|---|---|---|---|---|
| 1 | 62.55 | C24 H26 N6 O3 S | [M+H]+ | 479.1872 | 479.1860 | 1.2 | 2.50 | 64.99 | 15.0 |

**Figure 52.** Mass spectrum of compound **5g**

DOPNALAB

| | Item | Value |
|---|---|---|
| | Acquired Date&Time | 5.02.2019 12:22:15 |
| | Acquired by | System Administrator |
| | Filename | C:\Users\dopnalab\Desktop\derya\OP\op-201.ispd |
| | Spectrum name | op-201 |
| | Sample name | OP-20 |
| | Sample ID | |
| | Option | |
| | Comment | |
| | No. of Scans | 10 |
| | Resolution | 4 [cm-1] |
| | Apodization | Happ-Genzel |

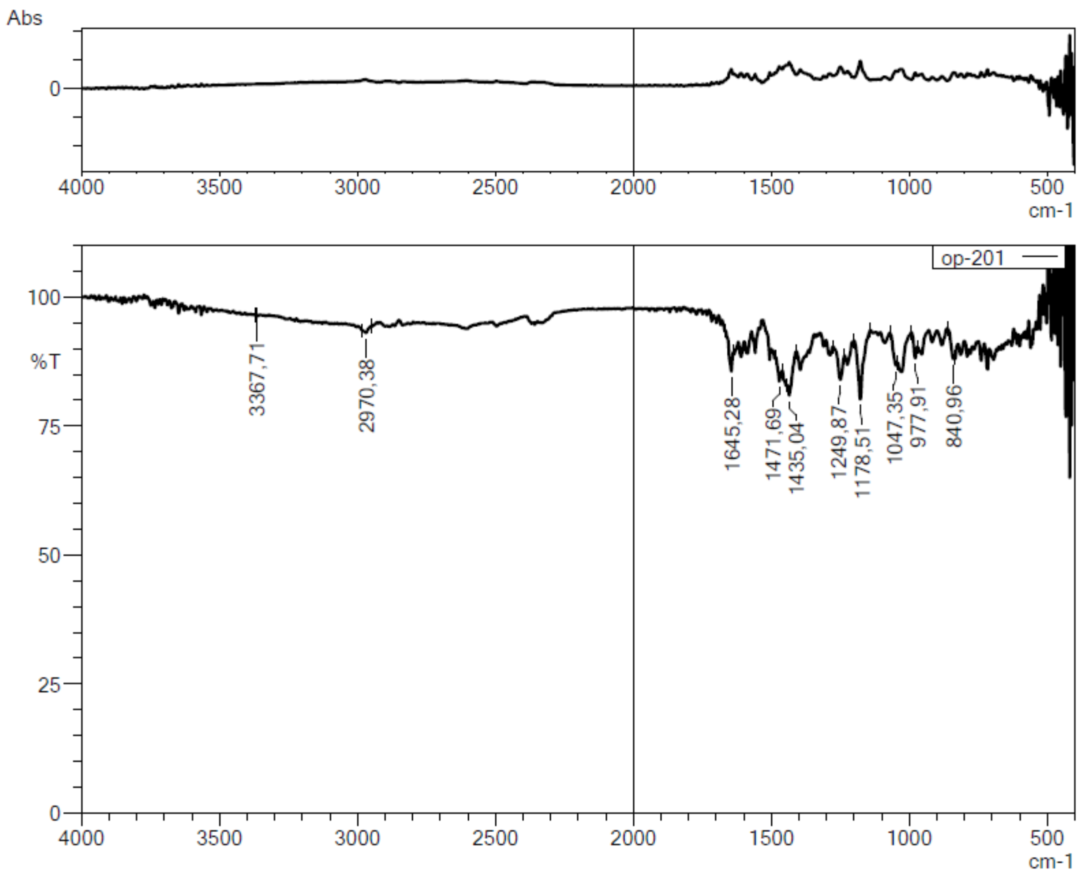


**Figure 53.** IR spectrum of compound **5h**

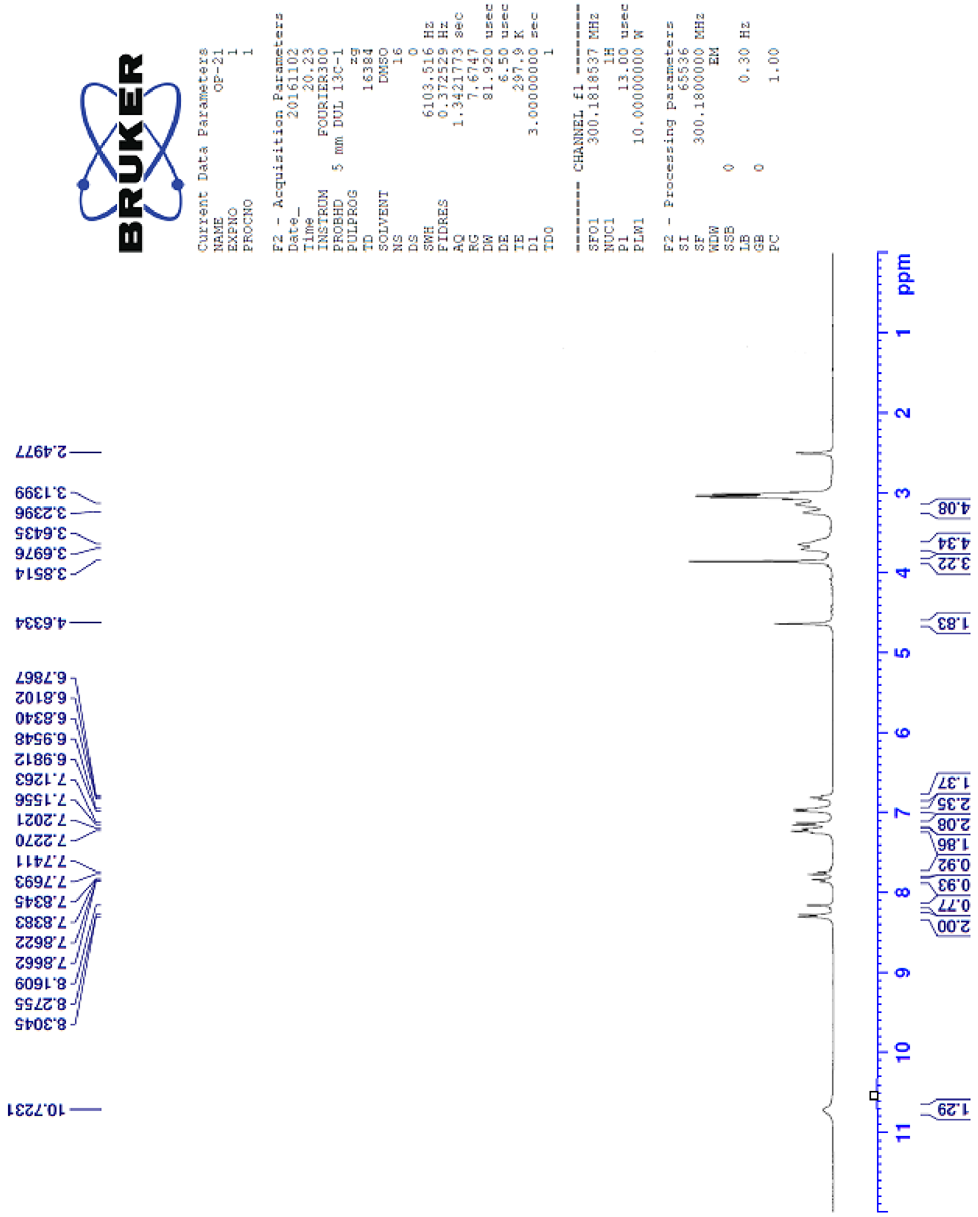


**Figure 54.** $^1$H-NMR spectra of compound **5h**

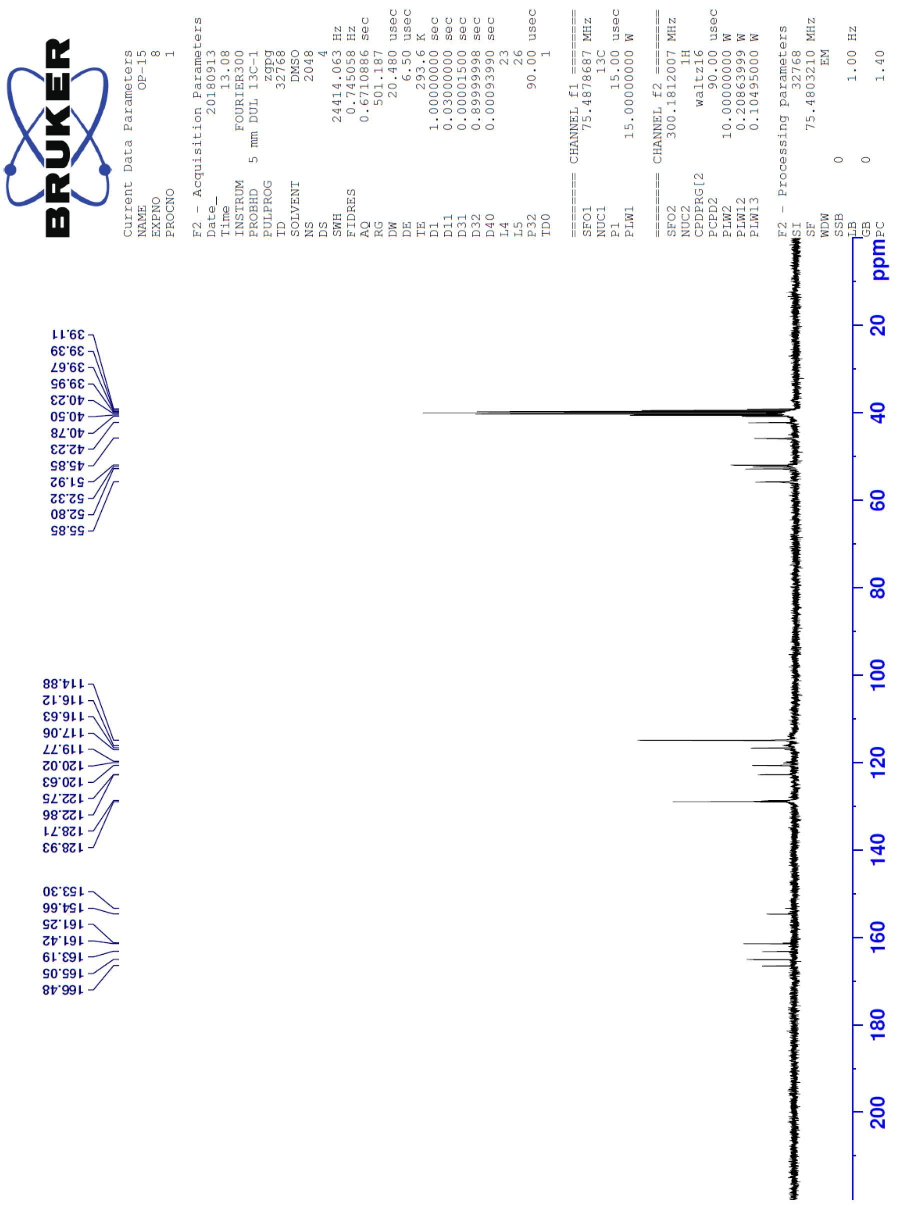


**Figure 55.** $^{13}$C-NMR spectra of compound **5h**

Formula Predictor Report - OP-21_7.lcd Page 1 of 1

Data File: C:\LabSolutions\Data\Analiz\uac\OP-21_7.lcd

| Elmt | Val. | Min | Max | Elmt | Val. | Min | Max | Elmt | Val. | Min | Max | Elmt | Val. | Min | Max | Use Adduct |
|---|---|---|---|---|---|---|---|---|---|---|---|---|---|---|---|---|
| H | 1 | 20 | 40 | O | 2 | 1 | 5 | S | 2 | 1 | 1 | Ru | 2 | 0 | 0 | H |
| C | 4 | 15 | 30 | F | 1 | 0 | 0 | Cl | 1 | 0 | 1 | Pd | 2 | 0 | 0 | |
| N | 3 | 6 | 8 | P | 3 | 0 | 0 | Br | 1 | 0 | 1 | I | 3 | 0 | 0 | |

Error Margin (ppm): 15
HC Ratio: unlimited
Max Isotopes: 3
MSn Iso RI (%): 10.00

DBE Range: 16.0 - 20.0
Apply N Rule: yes
Isotope RI (%): 1.00
MSn Logic Mode: AND

Electron Ions: both
Use MSn Info: yes
Isotope Res: 9000
Max Results: 500

Event#: 1 MS(E+) Ret. Time : 5.613 Scan# : 843

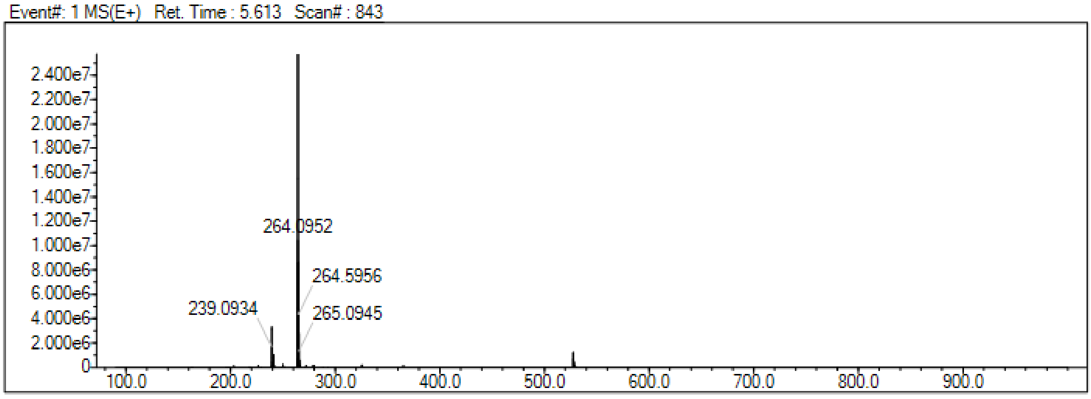


Measured region for 527.1849 m/z

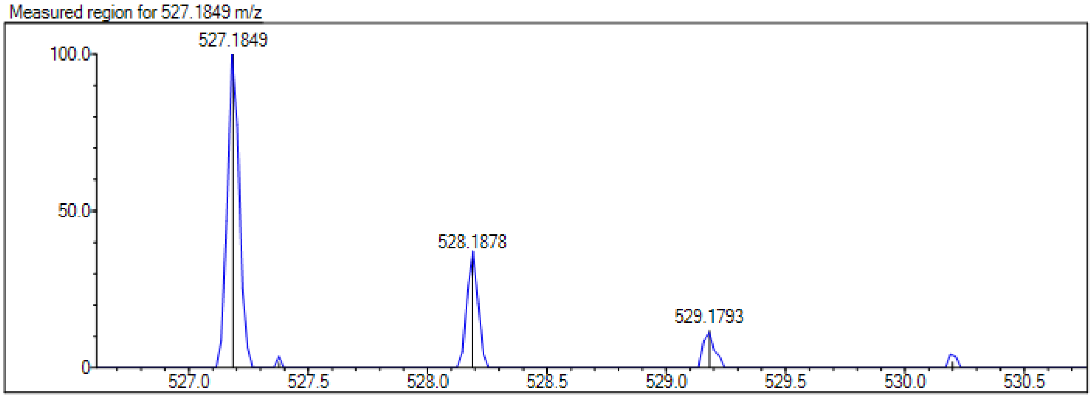


C28 H26 N6 O3 S [M+H]+ : Predicted region for 527.1860 m/z

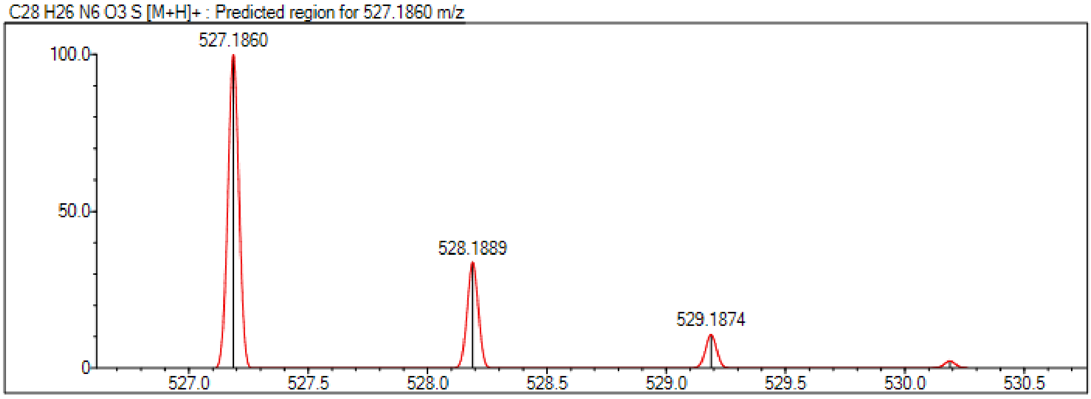


| Rank | Score | Formula (M) | Ion | Meas. m/z | Pred. m/z | Df. (mDa) | Df. (ppm) | Iso | DBE |
|---|---|---|---|---|---|---|---|---|---|
| 1 | 62.15 | C28 H26 N6 O3 S | [M+H]+ | 527.1849 | 527.1860 | -1.1 | -2.09 | 63.89 | 19.0 |

**Figure 56.** Mass spectrum of compound **5h**

DOPNALAB

| Item | Value |
|---|---|
| Acquired Date&Time | 5.02.2019 13:59:03 |
| Acquired by | System Administrator |
| Filename | C:\Users\dopnalab\Desktop\derya\OP\op-111.ispd |
| Spectrum name | op-111 |
| Sample name | OP-11 |
| Sample ID | |
| Option | |
| Comment | |
| No. of Scans | 10 |
| Resolution | 4 [cm-1] |
| Apodization | Happ-Genzel |

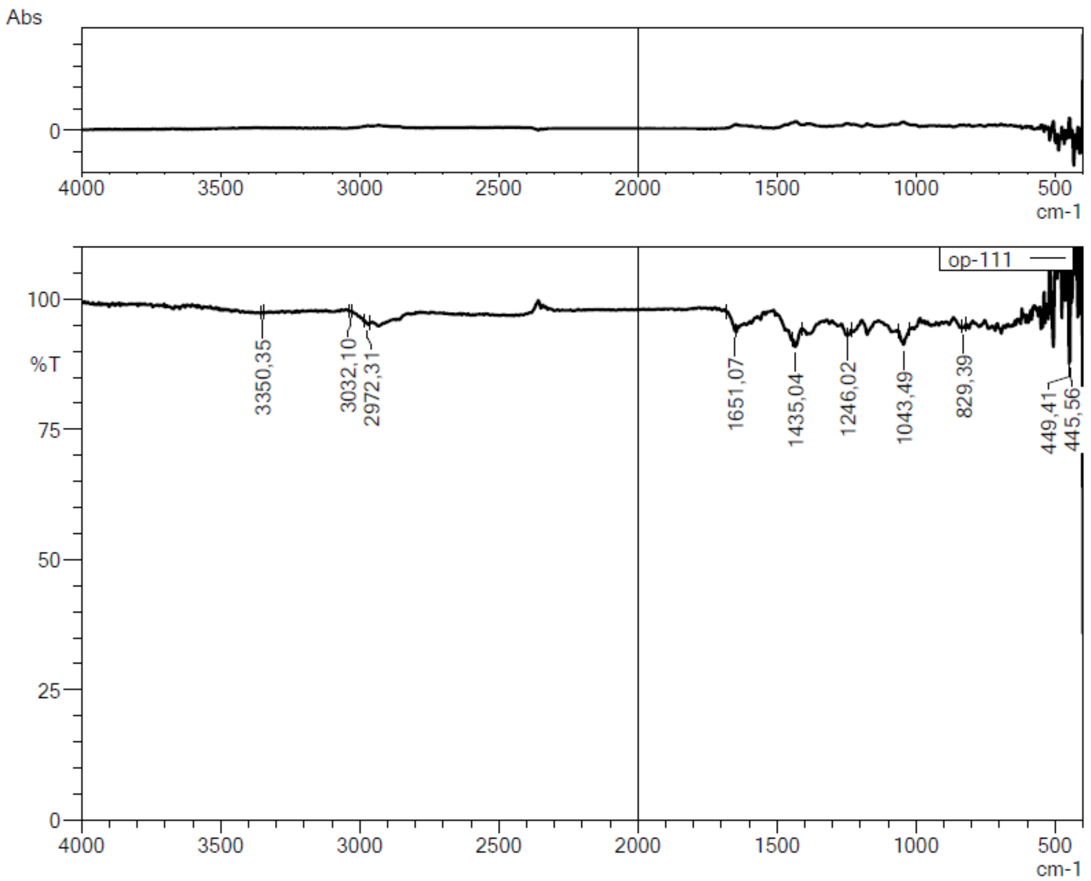


**Figure 57.** IR spectrum of compound **5ı**

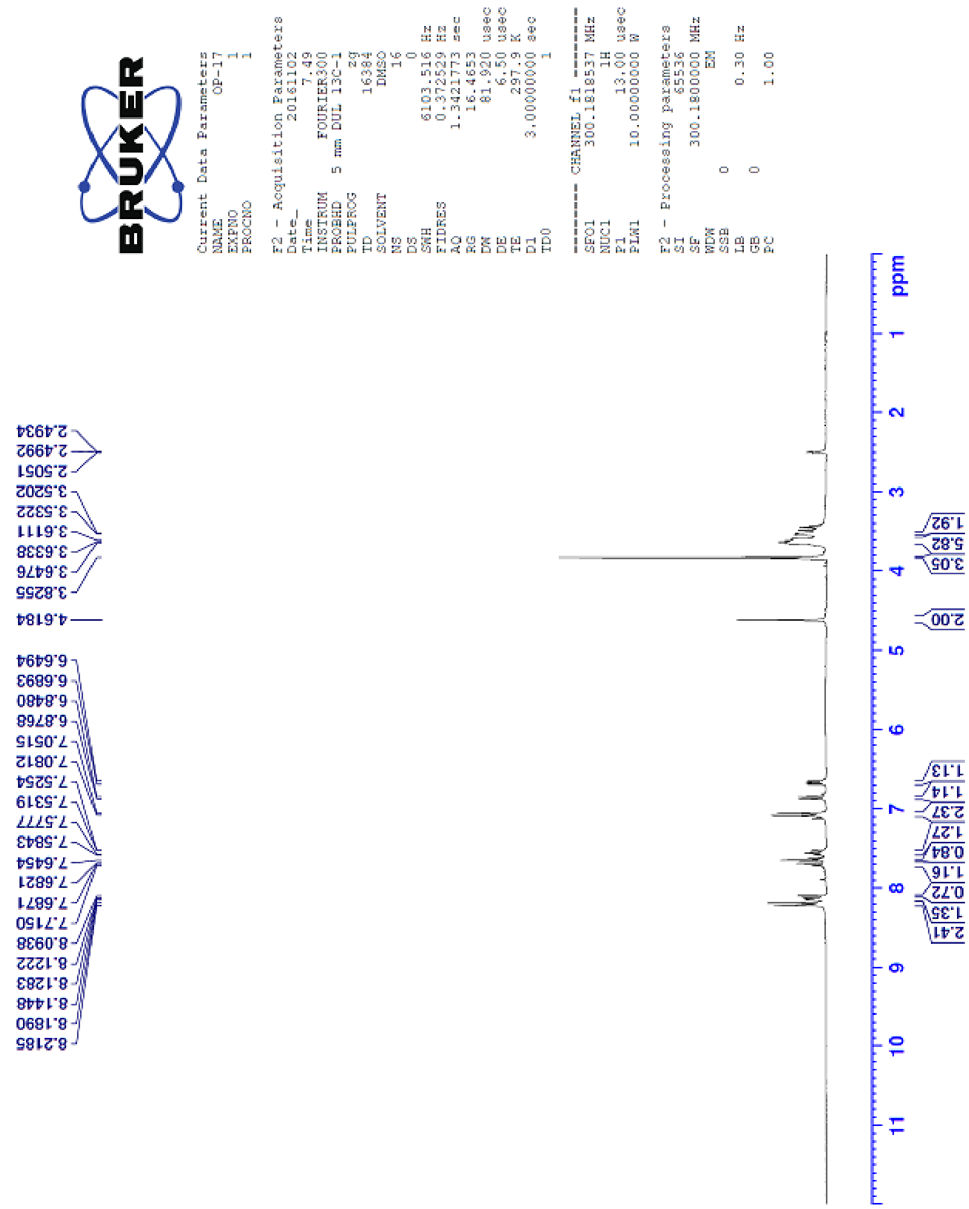


**Figure 58.** $^{1}$H-NMR spectra of compound **5ı**

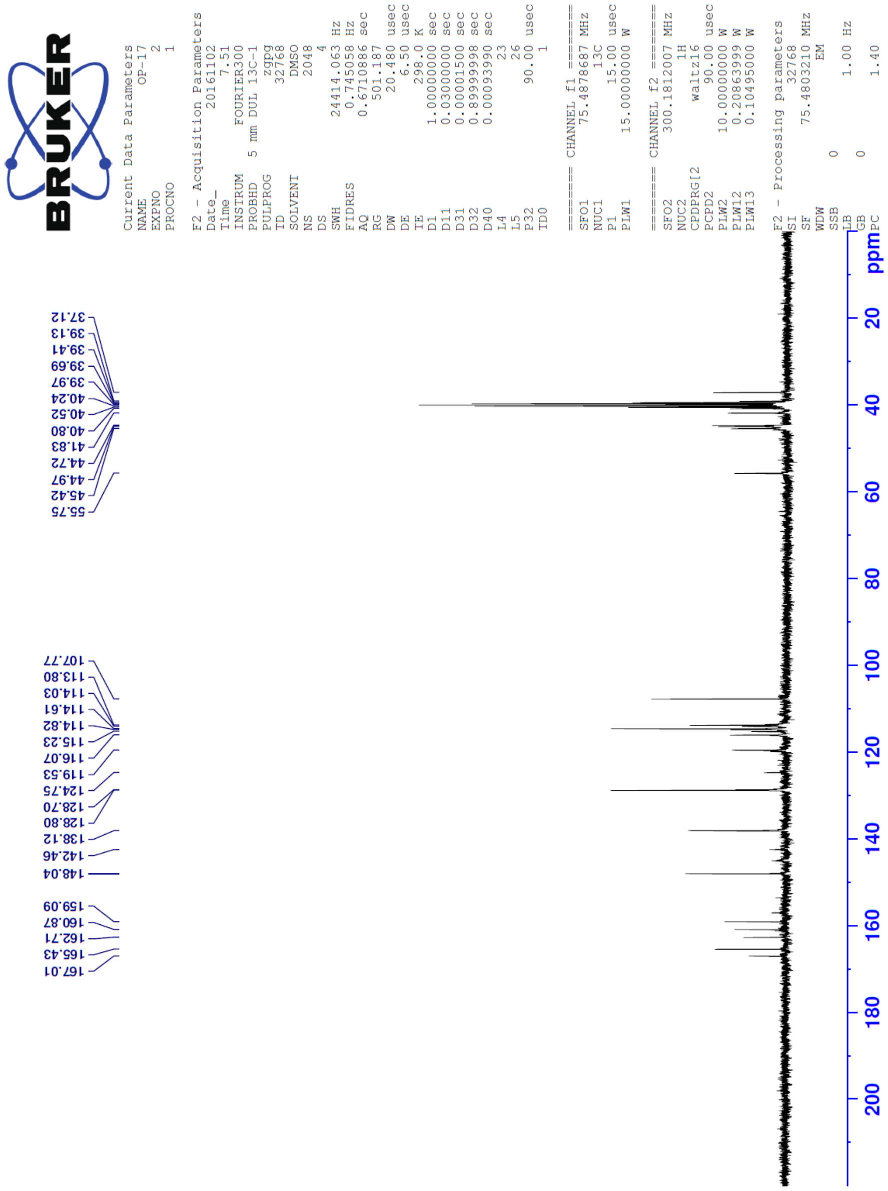


**Figure 59.** $^{13}$C-NMR spectra of compound **5ı**

Data File: C:\LabSolutions\Data\Analiz\uac\OP-17_6.lcd

| Elmt | Val. | Min | Max | Elmt | Val. | Min | Max | Elmt | Val. | Min | Max | Elmt | Val. | Min | Max | Use Adduct |
|---|---|---|---|---|---|---|---|---|---|---|---|---|---|---|---|---|
| H | 1 | 20 | 40 | O | 2 | 1 | 5 | S | 2 | 1 | 1 | Ru | 2 | 0 | 0 | H |
| C | 4 | 15 | 30 | F | 1 | 0 | 0 | Cl | 1 | 0 | 1 | Pd | 2 | 0 | 0 | |
| N | 3 | 6 | 8 | P | 3 | 0 | 0 | Br | 1 | 0 | 1 | I | 3 | 0 | 0 | |

Error Margin (ppm): 5
HC Ratio: unlimited
Max Isotopes: 3
MSn Iso RI (%): 10.00

DBE Range: 12.0 - 20.0
Apply N Rule: yes
Isotope RI (%): 1.00
MSn Logic Mode: AND

Electron Ions: both
Use MSn Info: yes
Isotope Res: 9000
Max Results: 500

Event#: 1 MS(E+) Ret. Time : 3.653 -> 3.653 Scan# : 549 -> 549

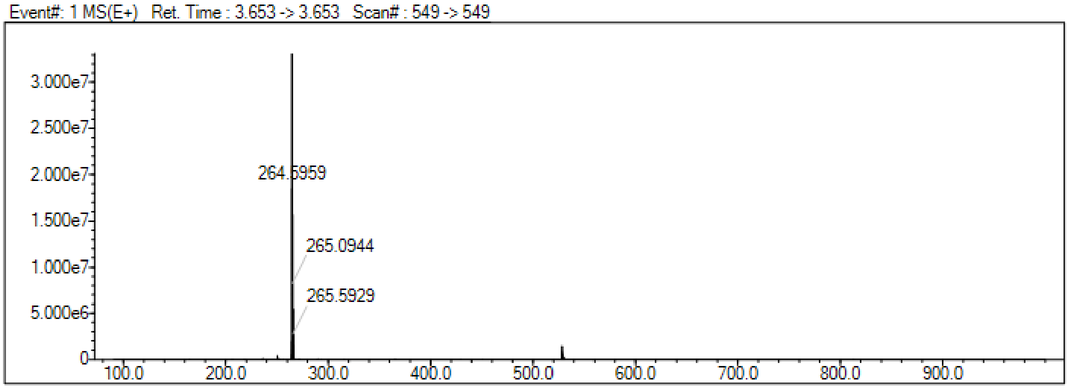


Measured region for 528.1825 m/z

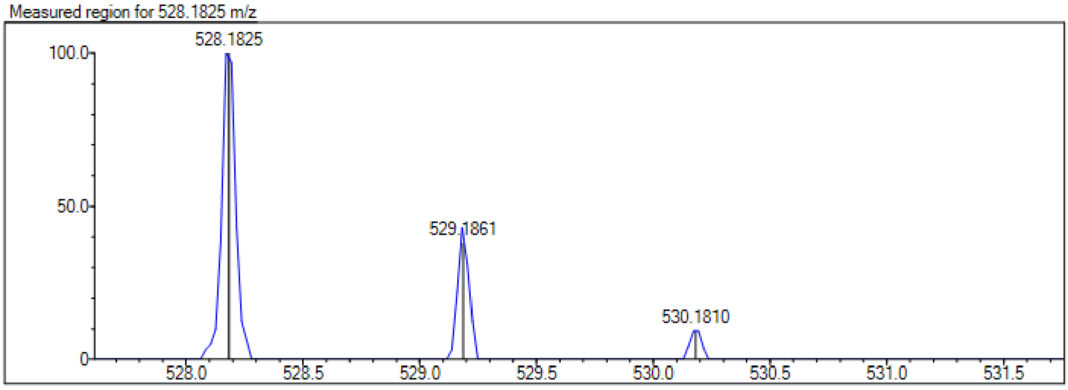


C27 H25 N7 O3 S [M+H]+ : Predicted region for 528.1812 m/z

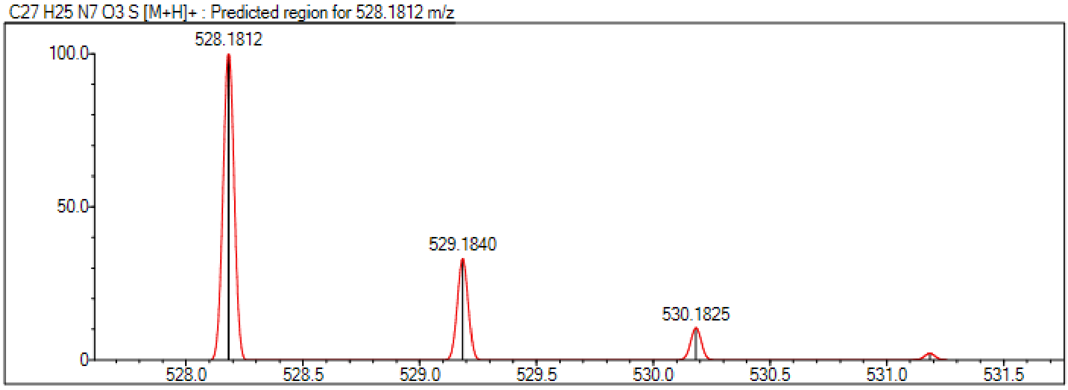


| Rank | Score | Formula (M) | Ion | Meas. m/z | Pred. m/z | Df. (mDa) | Df. (ppm) | Iso | DBE |
|---|---|---|---|---|---|---|---|---|---|
| 1 | 71.96 | C27 H25 N7 O3 S | [M+H]+ | 528.1825 | 528.1812 | 1.3 | 2.46 | 74.69 | 19.0 |

**Figure 60.** Mass spectrum of compound **5ı**

DOPNALAB

| | Item | Value |
|---|---|---|
| | Acquired Date&Time | 5.02.2019 13:53:06 |
| | Acquired by | System Administrator |
| | Filename | C:\Users\dopnalab\Desktop\derya\OP\op-7a1.ispd |
| | Spectrum name | op-7a1 |
| | Sample name | OP-7A |
| | Sample ID | |
| | Option | |
| | Comment | |
| | No. of Scans | 10 |
| | Resolution | 4 [cm-1] |
| | Apodization | Happ-Genzel |

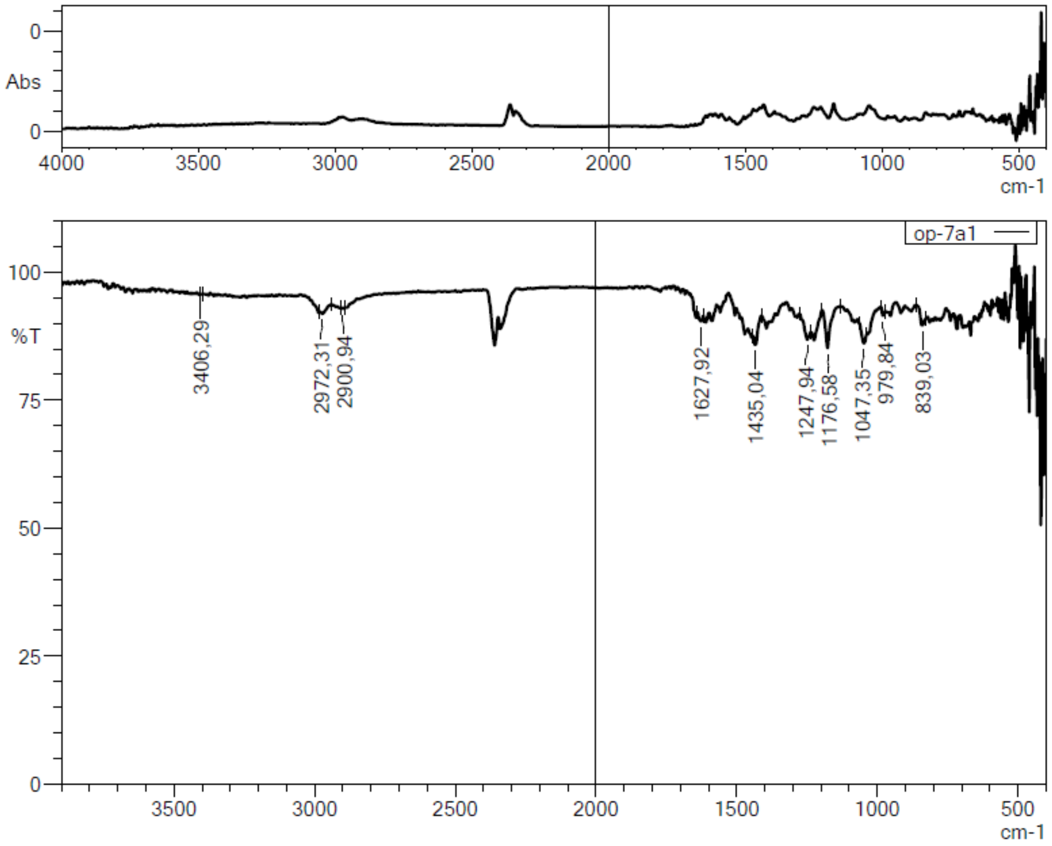


**Figure 61.** IR spectrum of compound **5j**

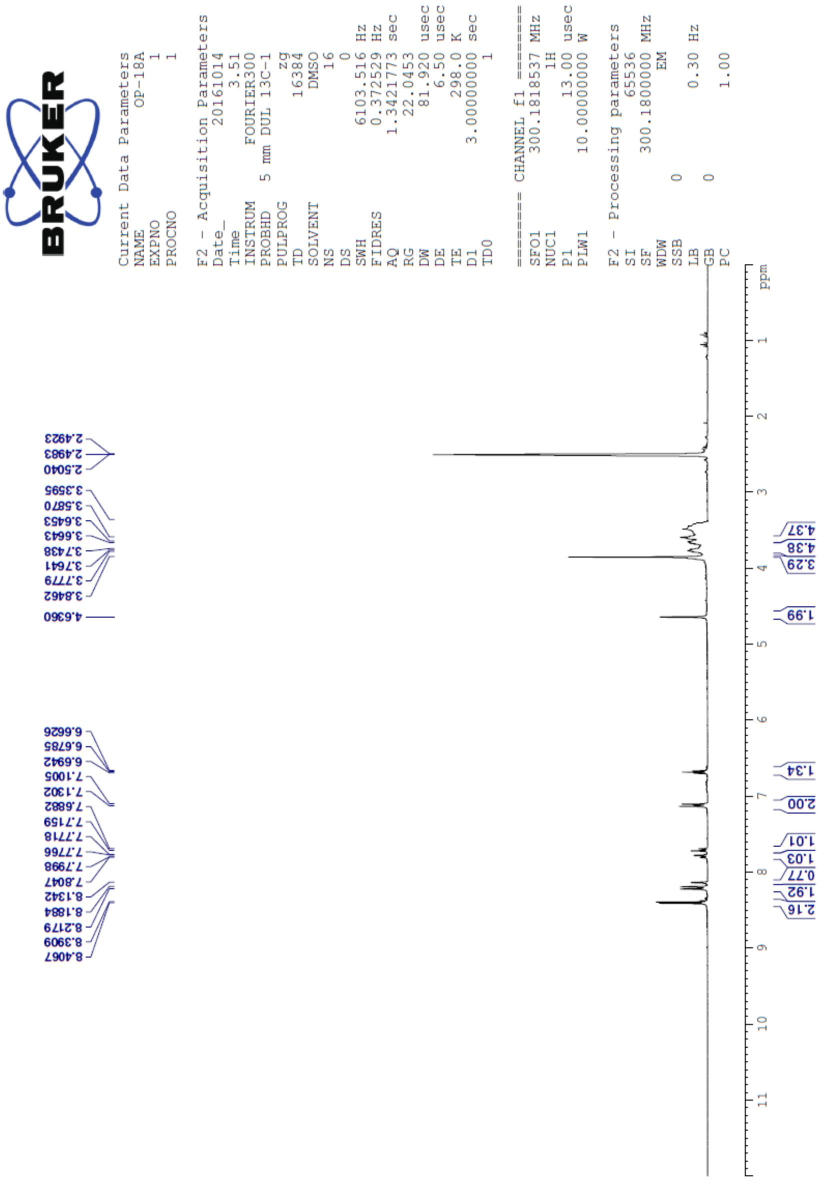

**Figure 62.** $^{1}$H-NMR spectra of compound **5j**

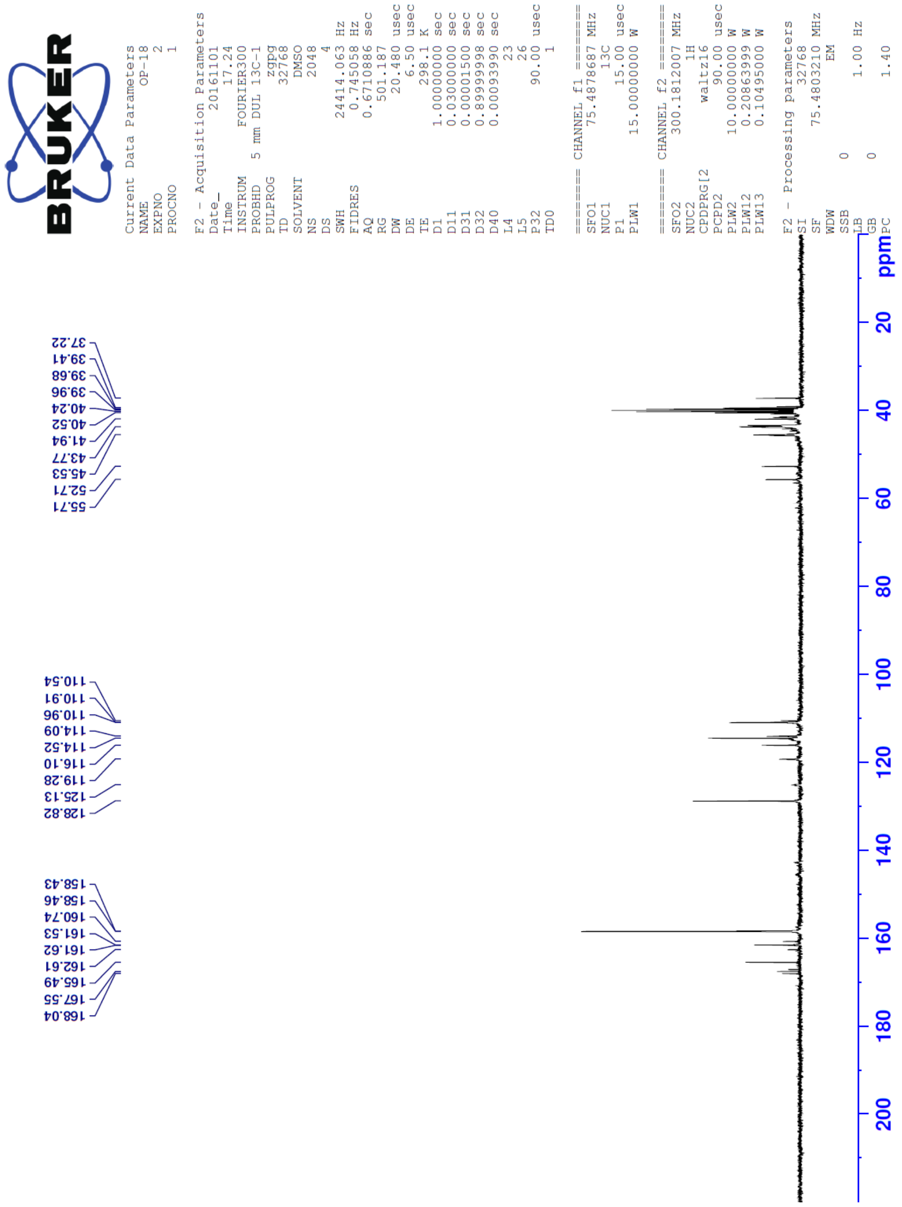

**Figure 63.** $^{13}$C-NMR spectra of compound **5j**

Formula Predictor Report - OP-18_2.lcd Page 1 of 1

Data File: C:\LabSolutions\Data\Analiz\uac\OP-18_2.lcd

| Elmt | Val. | Min | Max | Elmt | Val. | Min | Max | Elmt | Val. | Min | Max | Elmt | Val. | Min | Max | Use Adduct |
|---|---|---|---|---|---|---|---|---|---|---|---|---|---|---|---|---|
| H | 1 | 20 | 40 | O | 2 | 1 | 5 | S | 2 | 1 | 1 | Ru | 2 | 0 | 0 | H |
| C | 4 | 15 | 30 | F | 1 | 0 | 0 | Cl | 1 | 0 | 1 | Pd | 2 | 0 | 0 | |
| N | 3 | 6 | 8 | P | 3 | 0 | 0 | Br | 1 | 0 | 1 | I | 3 | 0 | 0 | |

| | | | | | |
|---|---|---|---|---|---|
| Error Margin (ppm): | 5 | DBE Range: | 12.0 - 20.0 | Electron Ions: | both |
| HC Ratio: | unlimited | Apply N Rule: | yes | Use MSn Info: | yes |
| Max Isotopes: | 3 | Isotope RI (%): | 1.00 | Isotope Res: | 9000 |
| MSn Iso RI (%): | 10.00 | MSn Logic Mode: | AND | Max Results: | 500 |

Event#: 1 MS(E+) Ret. Time : 4.827 -> 4.827 Scan# : 725 -> 725

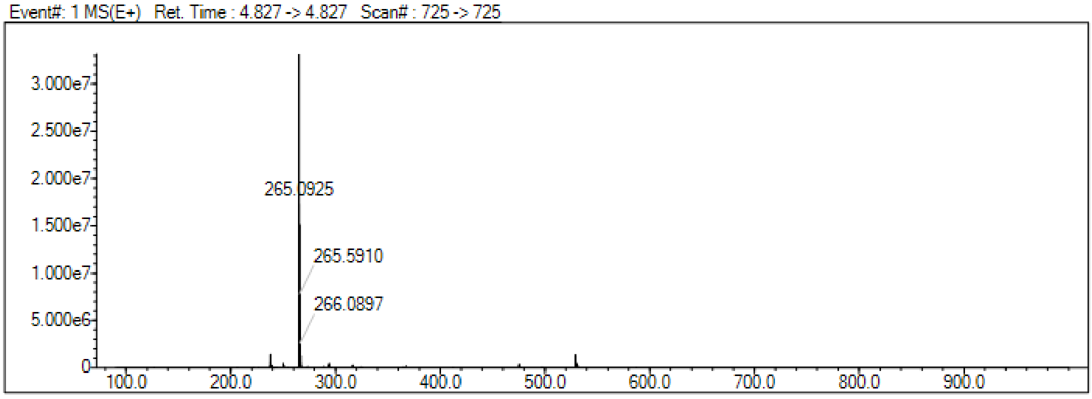


Measured region for 529.1755 m/z

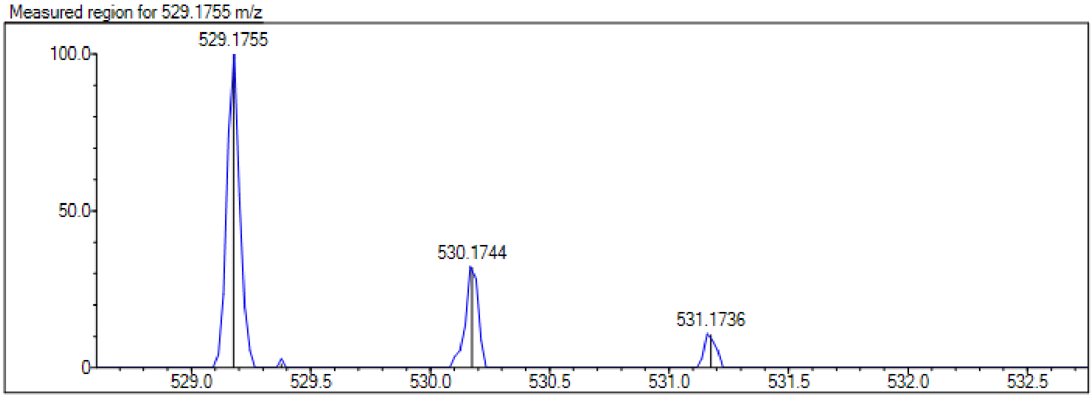


C26 H24 N8 O3 S [M+H]+ : Predicted region for 529.1765 m/z

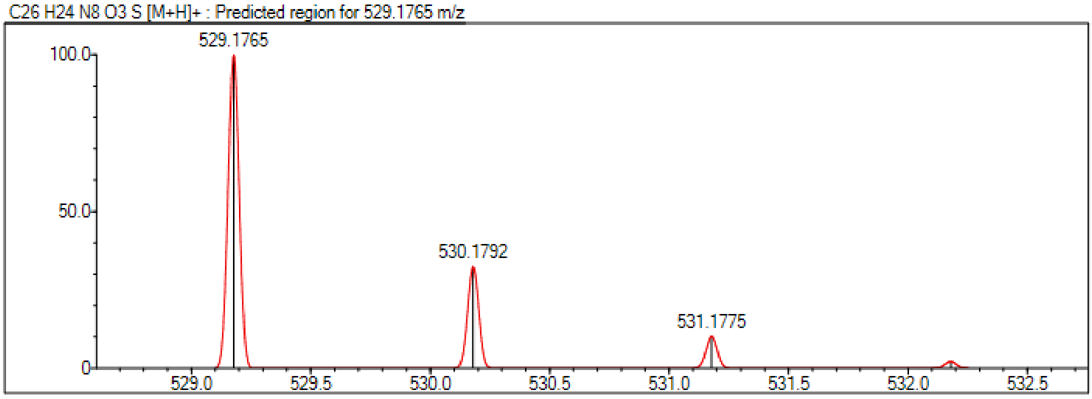


| Rank | Score | Formula (M) | Ion | Meas. m/z | Pred. m/z | Df. (mDa) | Df. (ppm) | Iso | DBE |
|---|---|---|---|---|---|---|---|---|---|
| 1 | 76.98 | C26 H24 N8 O3 S | [M+H]+ | 529.1755 | 529.1765 | -1.0 | -1.89 | 78.73 | 19.0 |

**Figure 64.** Mass spectrum of compound **5j**

# DOPNALAB

| | Item | Value |
|---|---|---|
| | Acquired Date&Time | 5.02.2019 12:14:00 |
| | Acquired by | System Administrator |
| | Filename | C:\Users\dopnalab\Desktop\derya\OP\op-301.ispd |
| | Spectrum name | op-301 |
| | Sample name | OP-30 |
| | Sample ID | |
| | Option | |
| | Comment | |
| | No. of Scans | 10 |
| | Resolution | 4 [cm-1] |
| | Apodization | Happ-Genzel |

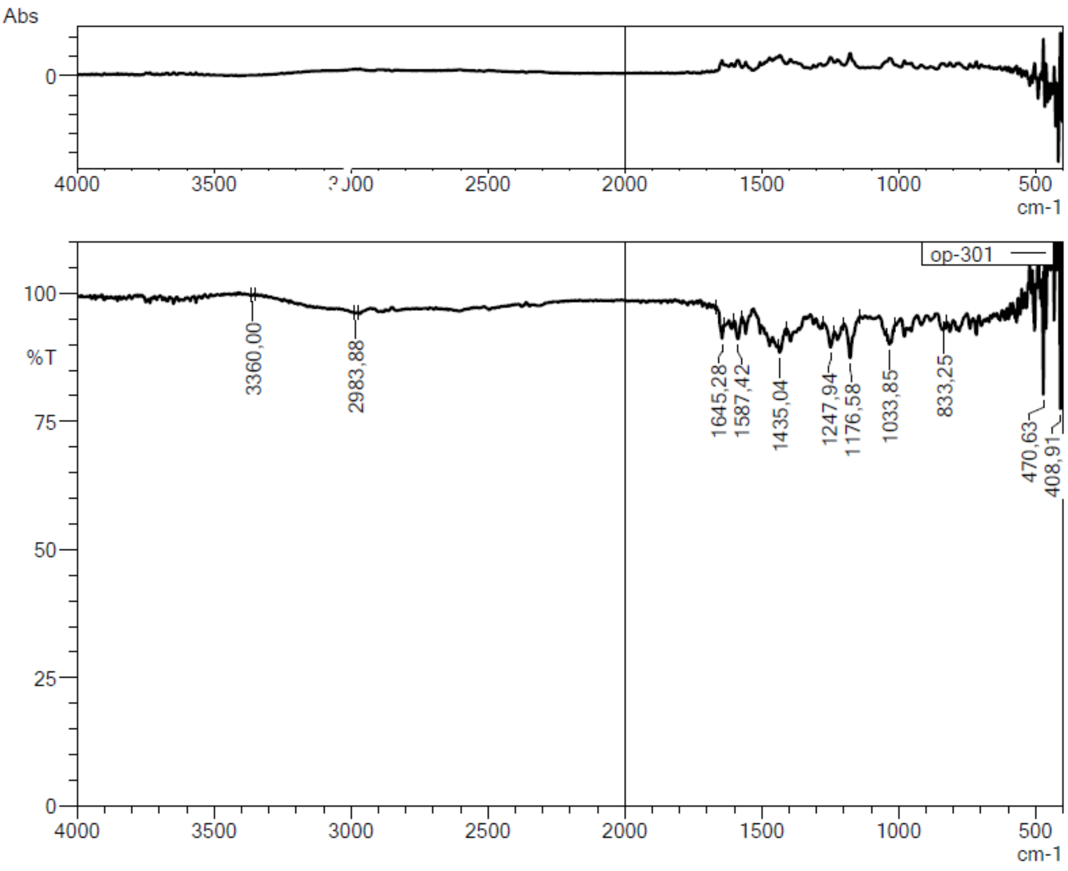


**Figure 65.** IR spectrum of compound **5k**

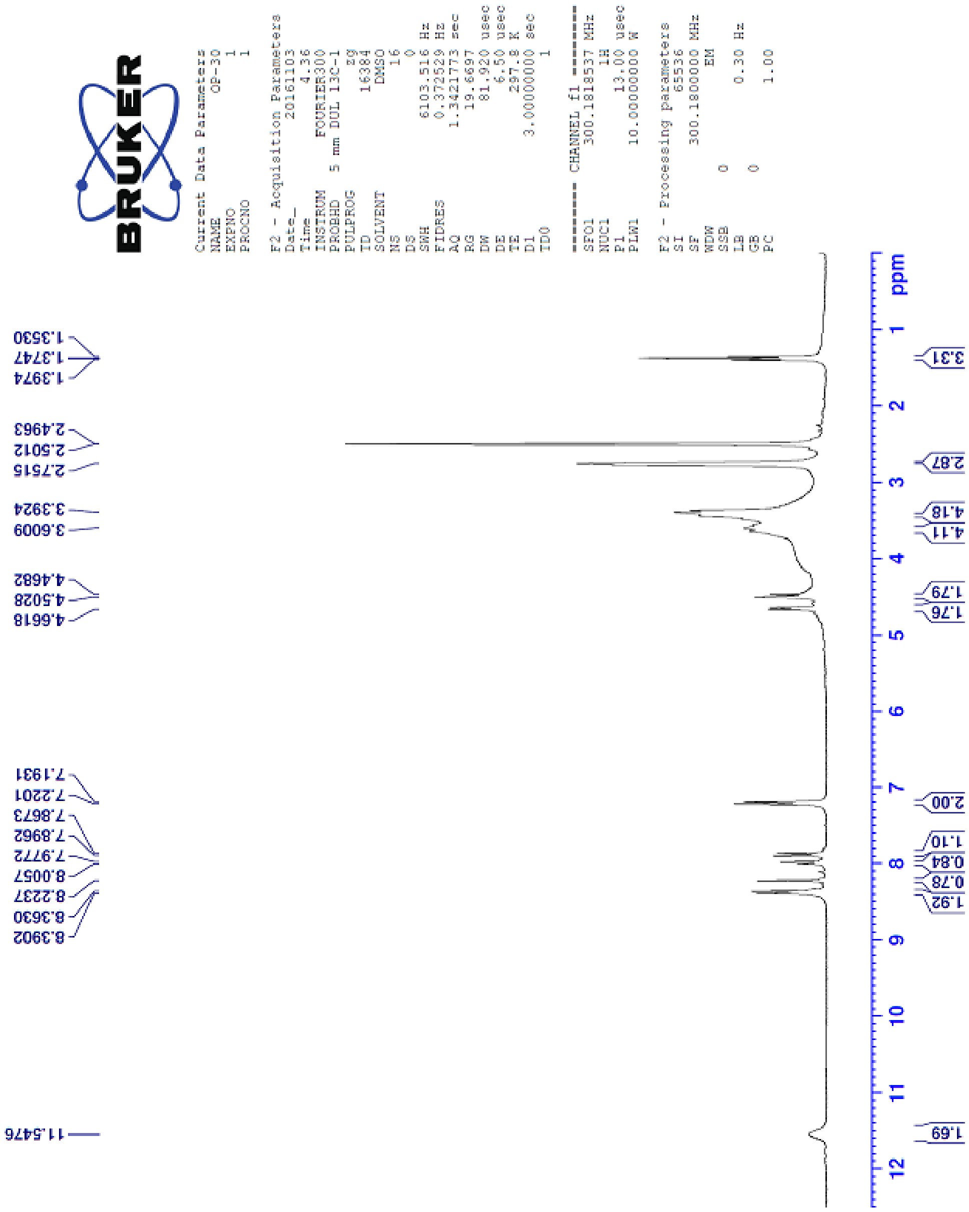


**Figure 66.** $^{1}$H-NMR spectra of compound **5k**

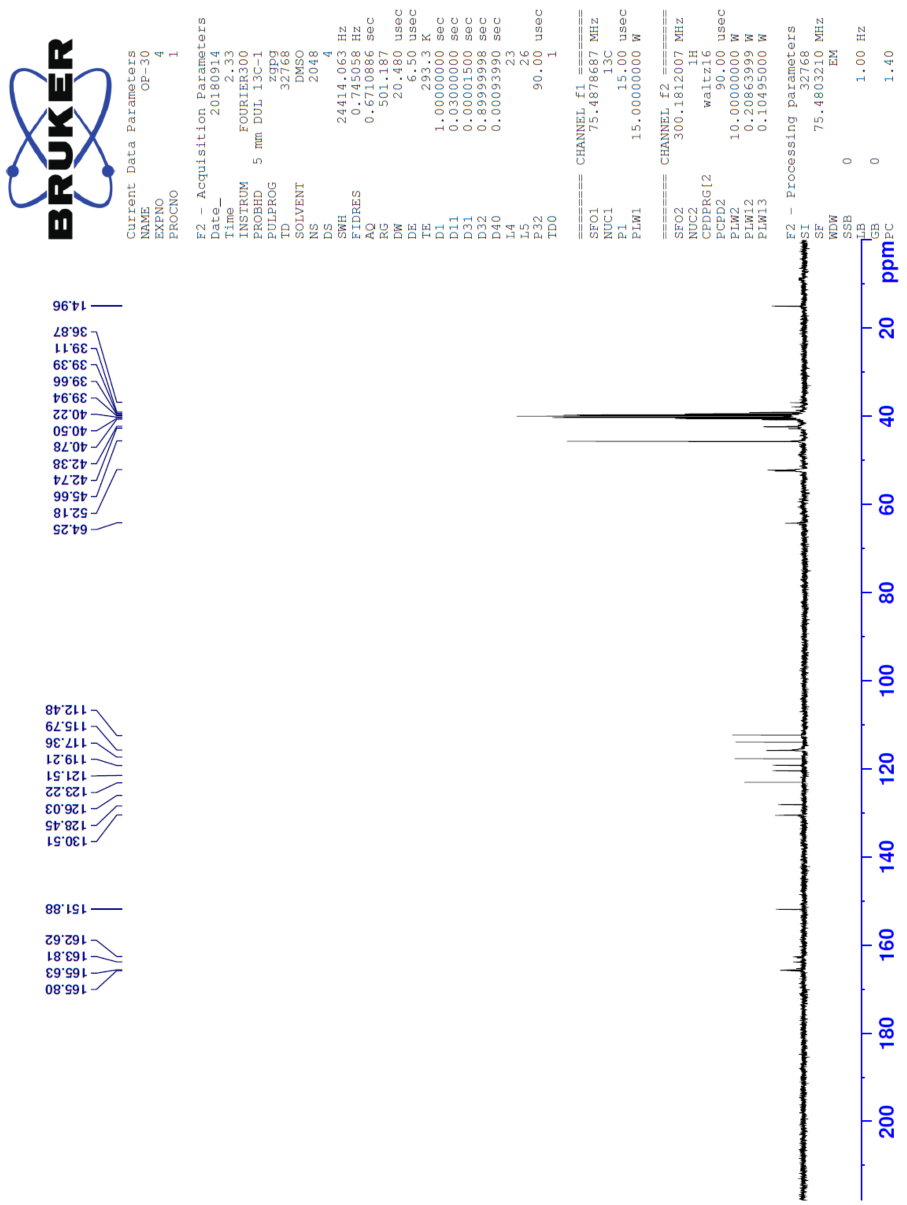


**Figure 67.** $^{13}$C-NMR spectra of compound **5k**

Data File: C:\LabSolutions\Data\Analiz\uac\OP-30_8.lcd

| Elmt | Val. | Min | Max | Elmt | Val. | Min | Max | Elmt | Val. | Min | Max | Elmt | Val. | Min | Max | Use Adduct |
|---|---|---|---|---|---|---|---|---|---|---|---|---|---|---|---|---|
| H | 1 | 20 | 40 | O | 2 | 1 | 5 | S | 2 | 1 | 1 | Ru | 2 | 0 | 0 | H |
| C | 4 | 15 | 30 | F | 1 | 0 | 1 | Cl | 1 | 0 | 1 | Pd | 2 | 0 | 0 | |
| N | 3 | 6 | 8 | P | 3 | 0 | 0 | Br | 1 | 0 | 1 | I | 3 | 0 | 0 | |

| | | | | | |
|---|---|---|---|---|---|
| Error Margin (ppm): | 5 | DBE Range: | 12.0 - 20.0 | Electron Ions: | both |
| HC Ratio: | unlimited | Apply N Rule: | yes | Use MSn Info: | yes |
| Max Isotopes: | 3 | Isotope RI (%): | 1.00 | Isotope Res: | 9000 |
| MSn Iso RI (%): | 10.00 | MSn Logic Mode: | AND | Max Results: | 500 |

Event#: 1 MS(E+) Ret. Time : 3.133 -> 3.133 Scan# : 471 -> 471

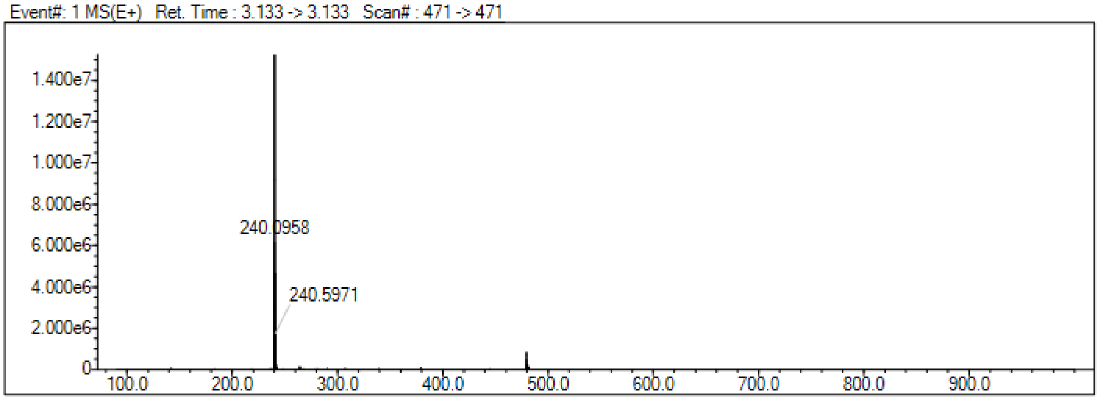


Measured region for 479.1873 m/z

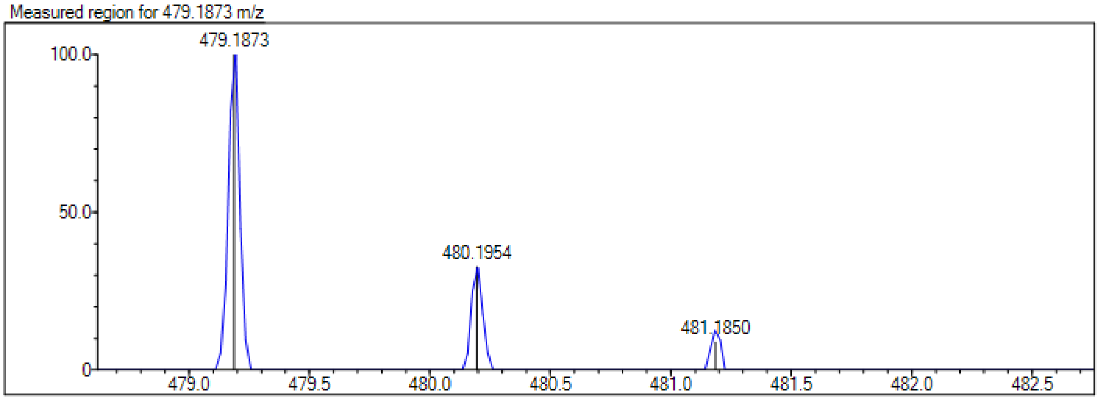


C24 H26 N6 O3 S [M+H]+ : Predicted region for 479.1860 m/z

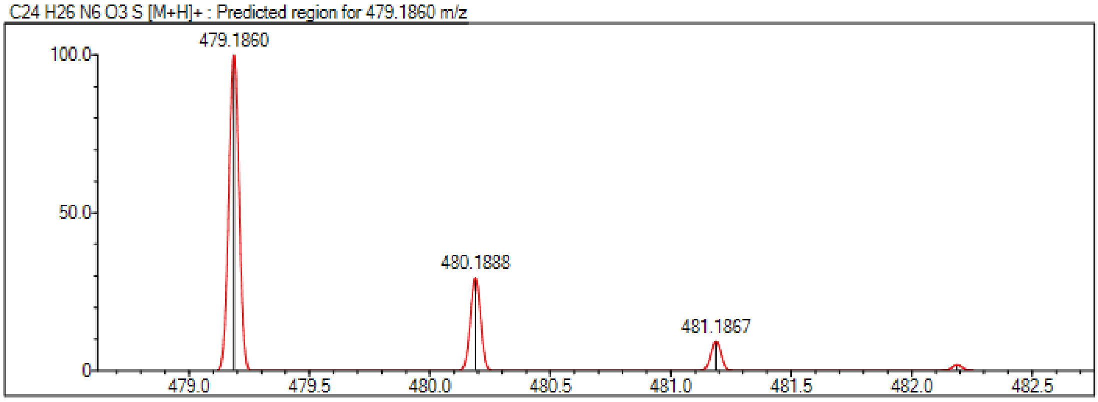


| Rank | Score | Formula (M) | Ion | Meas. m/z | Pred. m/z | Df. (mDa) | Df. (ppm) | Iso | DBE |
|---|---|---|---|---|---|---|---|---|---|
| 1 | 69.10 | C24 H26 N6 O3 S | [M+H]+ | 479.1873 | 479.1860 | 1.3 | 2.71 | 72.19 | 15.0 |

**Figure 68.** Mass spectrum of compound **5k**

## DOPNALAB

| Item | Value |
|---|---|
| Acquired Date&Time | 5.02.2019 11:35:11 |
| Acquired by | System Administrator |
| Filename | C:\Users\dopnalab\Desktop\derya\OP\OP-262.ispd |
| Spectrum name | OP-262 |
| Sample name | OP-26 |
| Sample ID | |
| Option | |
| Comment | |
| No. of Scans | 10 |
| Resolution | 4 [cm-1] |
| Apodization | Happ-Genzel |

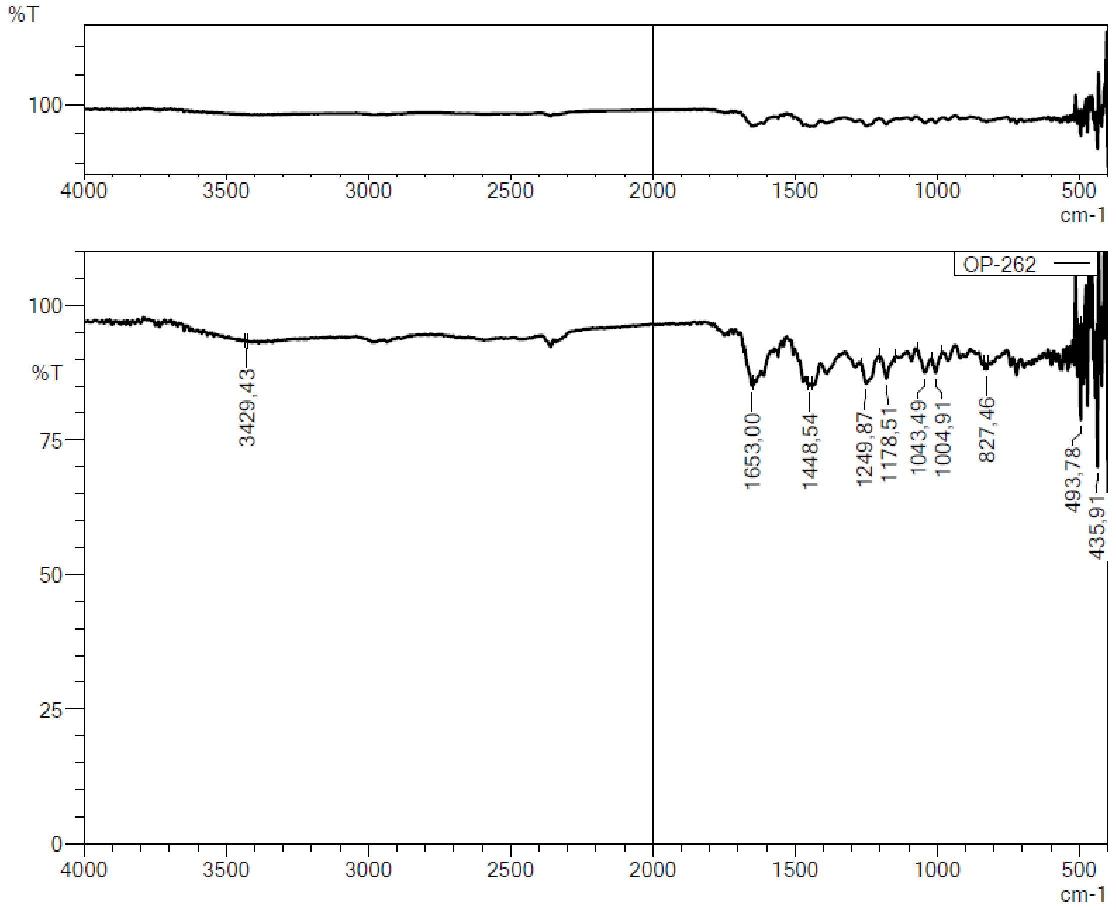


**Figure 69.** IR spectrum of compound **5l**

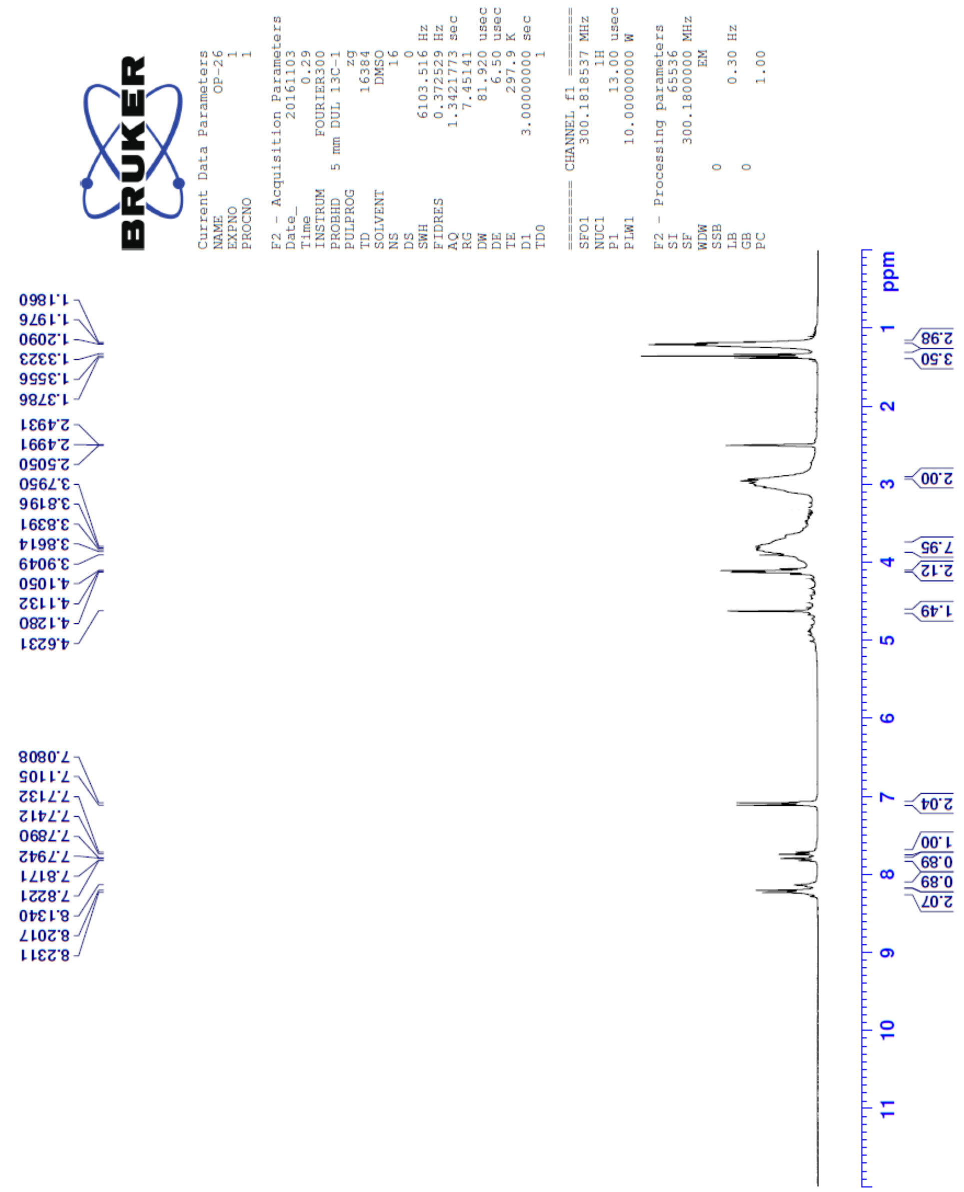


**Figure 70.** $^{1}$H-NMR spectra of compound **5l**

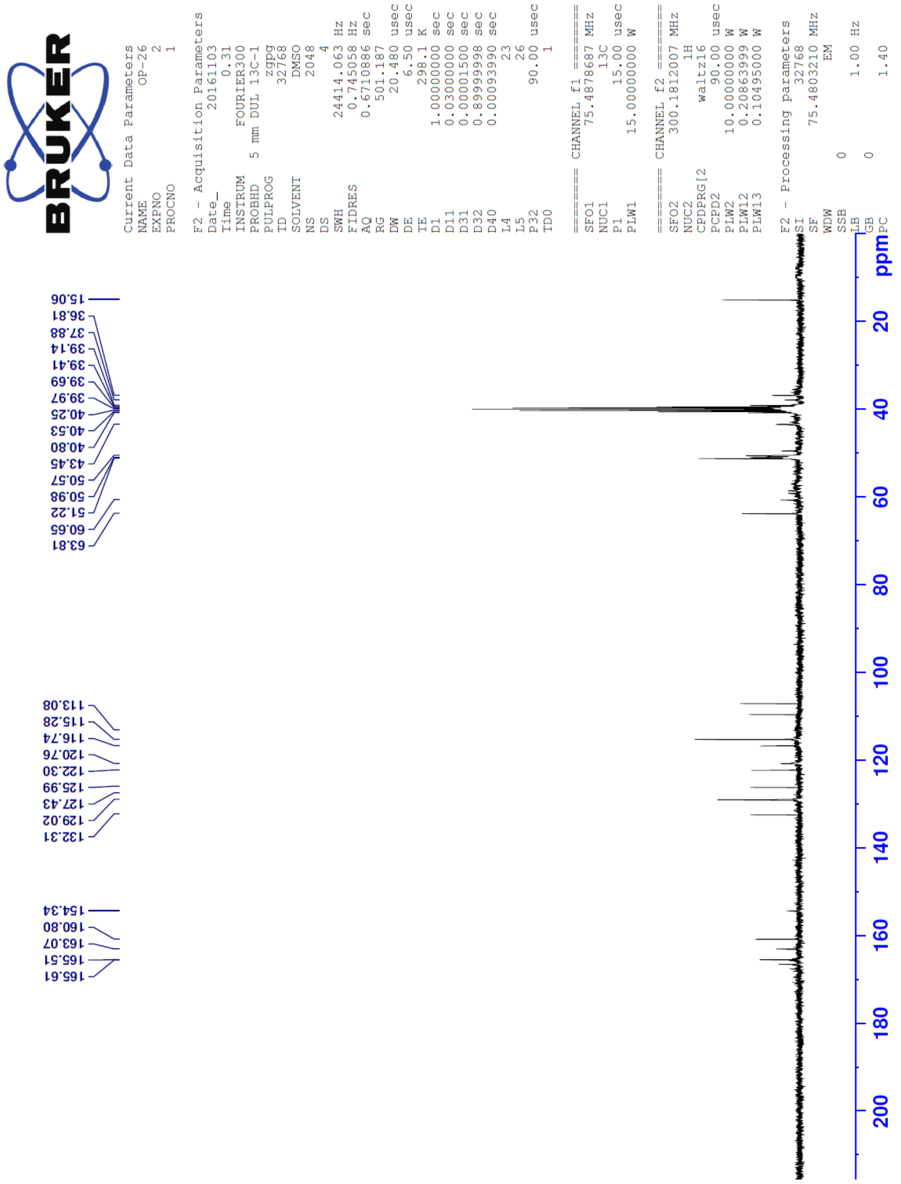

**Figure 71.** $^{13}$C-NMR spectra of compound **5l**

Data File: C:\LabSolutions\Data\Analiz\uac\OP-26_6.lcd

| Elmt | Val. | Min | Max | Elmt | Val. | Min | Max | Elmt | Val. | Min | Max | Elmt | Val. | Min | Max | Use Adduct |
|---|---|---|---|---|---|---|---|---|---|---|---|---|---|---|---|---|
| H | 1 | 20 | 40 | O | 2 | 1 | 5 | S | 2 | 1 | 1 | Ru | 2 | 0 | 0 | H |
| C | 4 | 15 | 30 | F | 1 | 0 | 1 | Cl | 1 | 0 | 1 | Pd | 2 | 0 | 0 | |
| N | 3 | 6 | 8 | P | 3 | 0 | 0 | Br | 1 | 0 | 1 | I | 3 | 0 | 0 | |

Error Margin (ppm): 15
HC Ratio: unlimited
Max Isotopes: 3
MSn Iso RI (%): 10.00

DBE Range: 12.0 - 20.0
Apply N Rule: yes
Isotope RI (%): 1.00
MSn Logic Mode: AND

Electron Ions: both
Use MSn Info: yes
Isotope Res: 9000
Max Results: 500

Event#: 1 MS(E+) Ret. Time : 3.307 -> 3.307 Scan# : 497 -> 497

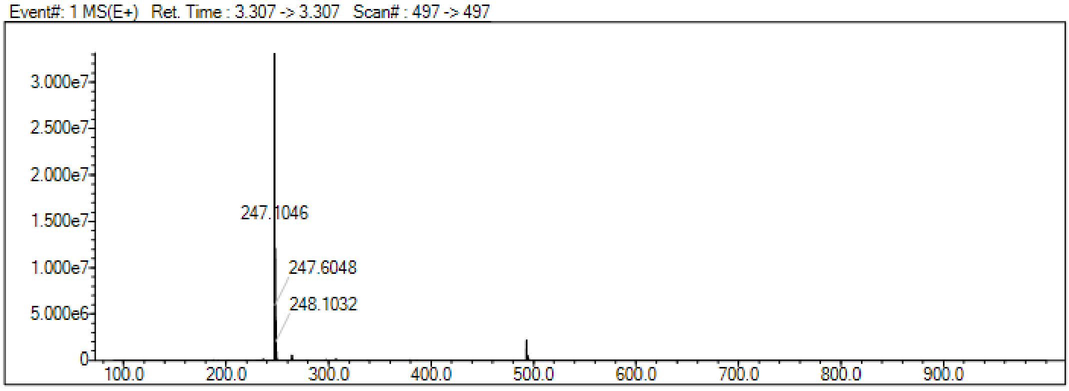


Measured region for 493.2019 m/z

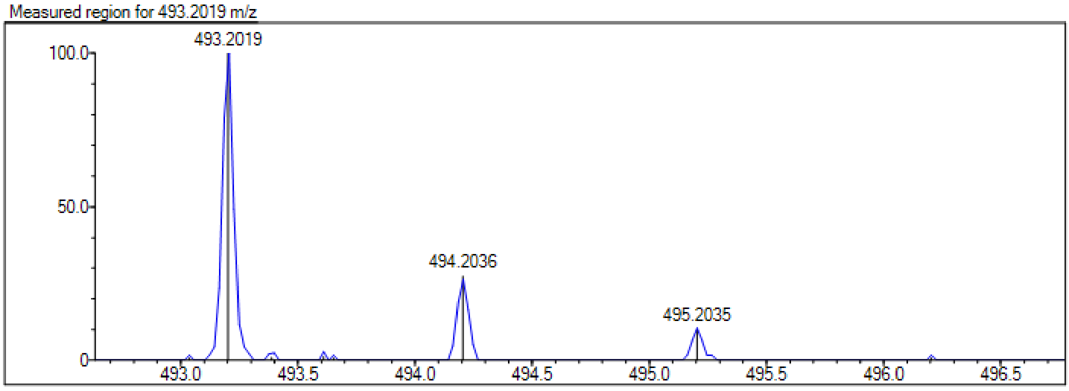


C25 H28 N6 O3 S [M+H]+ : Predicted region for 493.2016 m/z

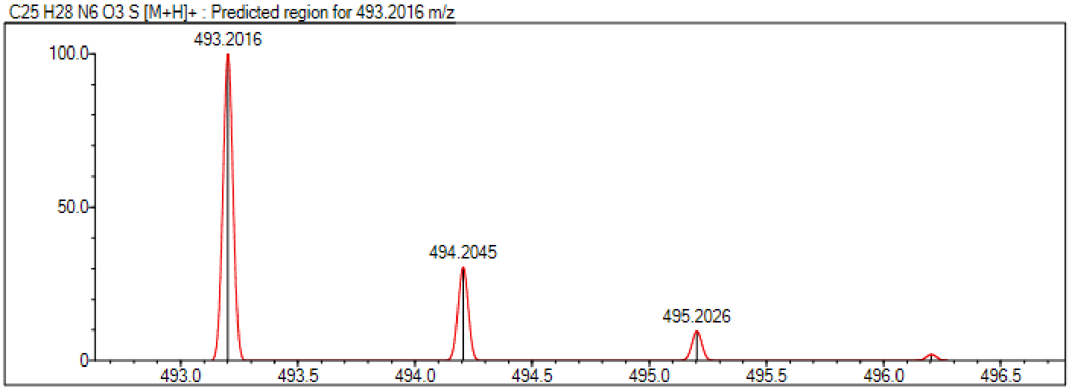


| Rank | Score | Formula (M) | Ion | Meas. m/z | Pred. m/z | Df. (mDa) | Df. (ppm) | Iso | DBE |
|---|---|---|---|---|---|---|---|---|---|
| 1 | 91.18 | C25 H28 N6 O3 S | [M+H]+ | 493.2019 | 493.2016 | 0.3 | 0.61 | 91.18 | 15.0 |

**Figure 72.** Mass spectrum of compound **5l**

DOPNALAB

| | Item | Value |
|---|---|---|
| | Acquired Date&Time | 5.02.2019 12:22:15 |
| | Acquired by | System Administrator |
| | Filename | C:\Users\dopnalab\Desktop\derya\OP\op-201.ispd |
| | Spectrum name | op-201 |
| | Sample name | OP-20 |
| | Sample ID | |
| | Option | |
| | Comment | |
| | No. of Scans | 10 |
| | Resolution | 4 [cm-1] |
| | Apodization | Happ-Genzel |

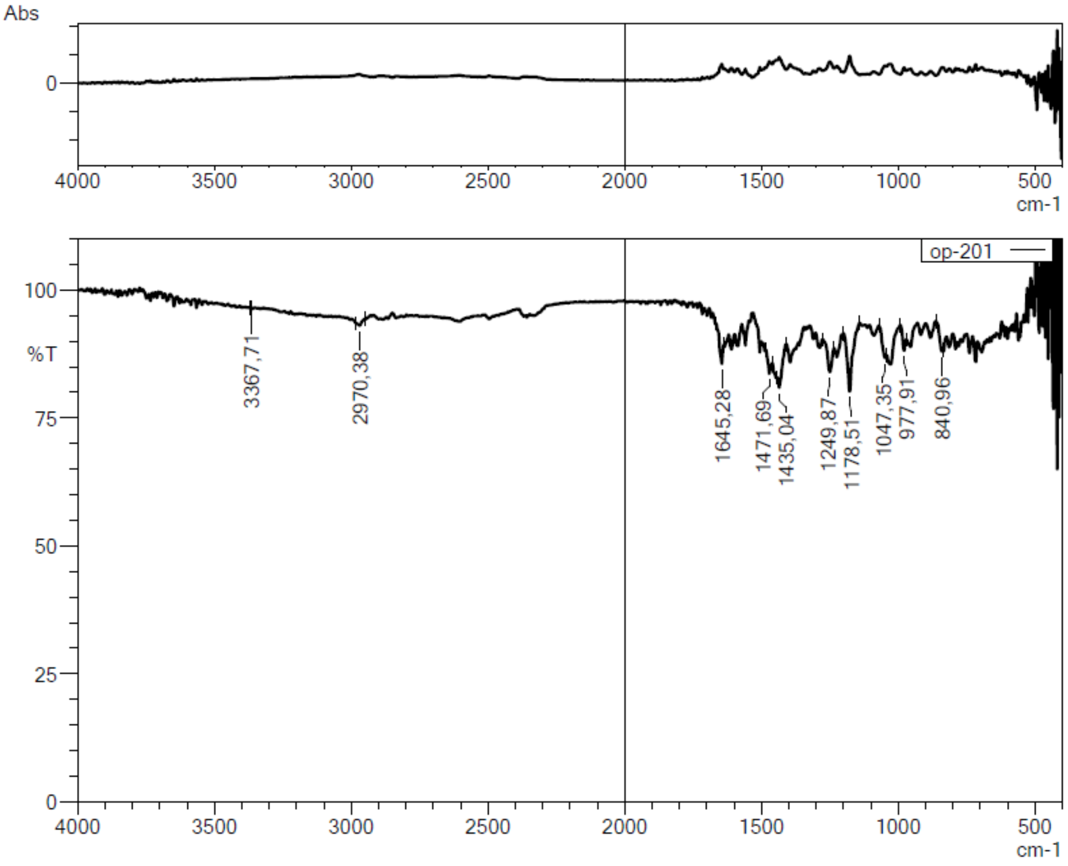


**Figure 73.** IR spectrum of compound **5m**

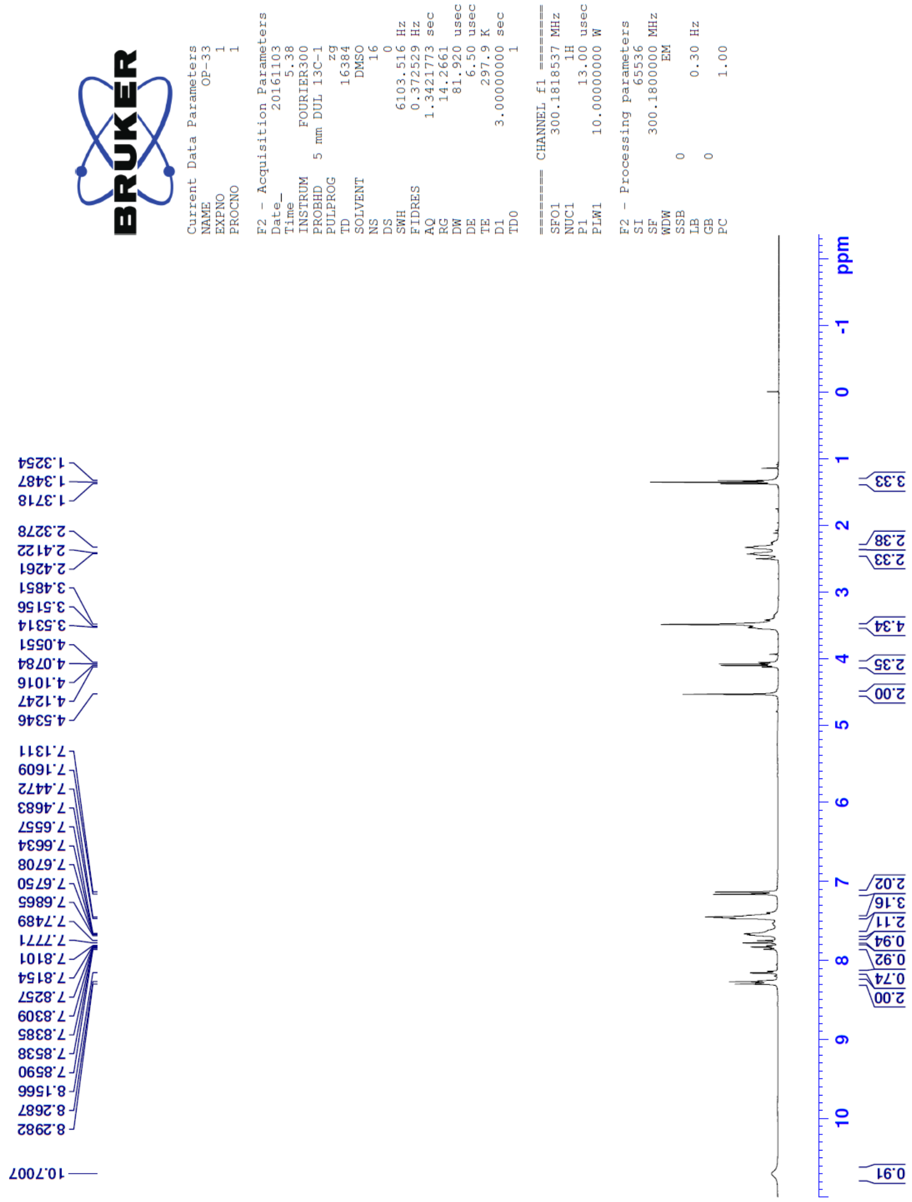

**Figure 74.** $^{1}$H-NMR spectra of compound **5m**

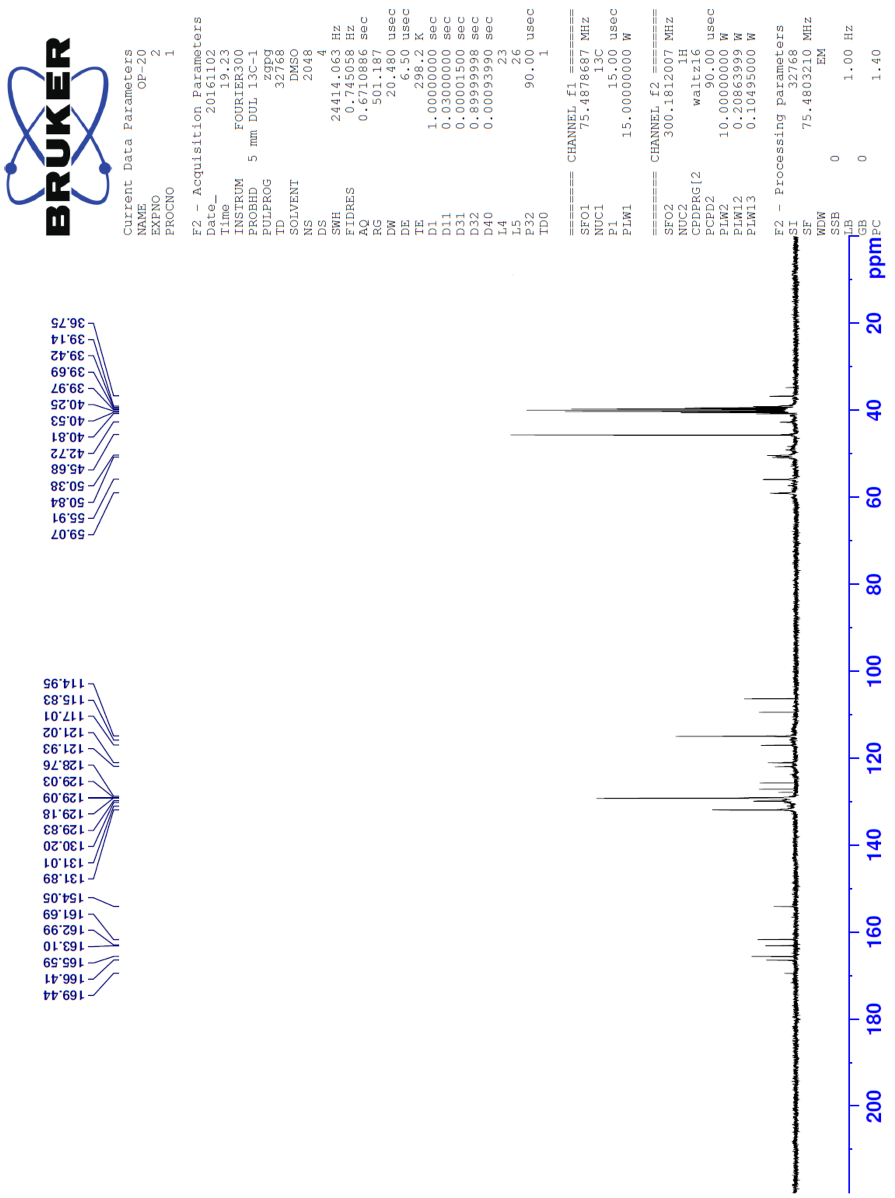

**Figure 75.** $^{13}$C-NMR spectra of compound **5m**

Data File: C:\LabSolutions\Data\Analiz\uac\OP-20_1.lcd

| Elmt | Val. | Min | Max | Elmt | Val. | Min | Max | Elmt | Val. | Min | Max | Elmt | Val. | Min | Max | Use Adduct |
|---|---|---|---|---|---|---|---|---|---|---|---|---|---|---|---|---|
| H | 1 | 20 | 40 | O | 2 | 1 | 5 | S | 2 | 1 | 1 | Ru | 2 | 0 | 0 | H |
| C | 4 | 15 | 30 | F | 1 | 0 | 0 | Cl | 1 | 0 | 1 | Pd | 2 | 0 | 0 | |
| N | 3 | 6 | 8 | P | 3 | 0 | 0 | Br | 1 | 0 | 1 | I | 3 | 0 | 0 | |

Error Margin (ppm): 5
HC Ratio: unlimited
Max Isotopes: 3
MSn Iso RI (%): 10.00

DBE Range: 16.0 - 20.0
Apply N Rule: yes
Isotope RI (%): 1.00
MSn Logic Mode: AND

Electron Ions: both
Use MSn Info: yes
Isotope Res: 9000
Max Results: 500

Event#: 1 MS(E+) Ret. Time : 3.693 -> 3.693 Scan# : 555 -> 555

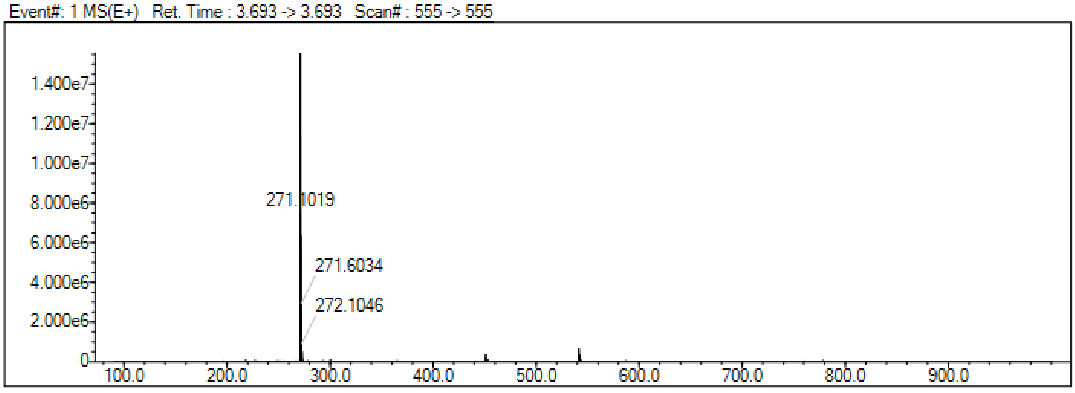


Measured region for 541.2002 m/z

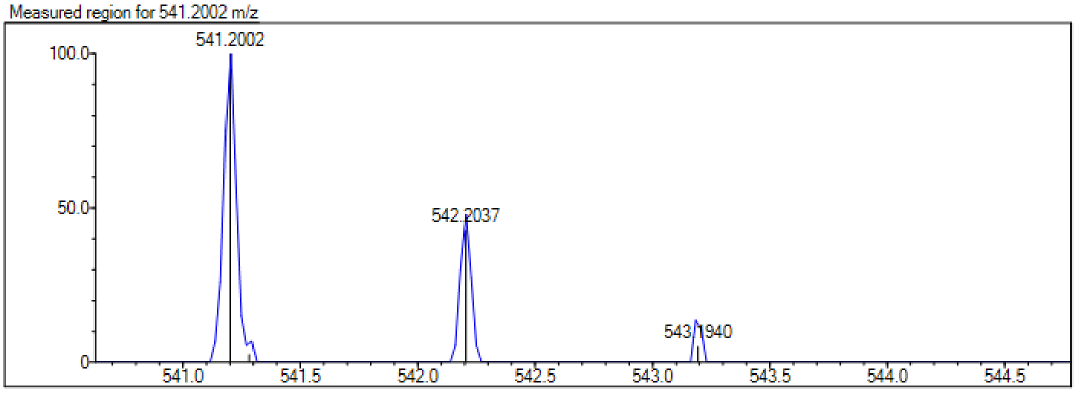


C29 H28 N6 O3 S [M+H]+ : Predicted region for 541.2016 m/z

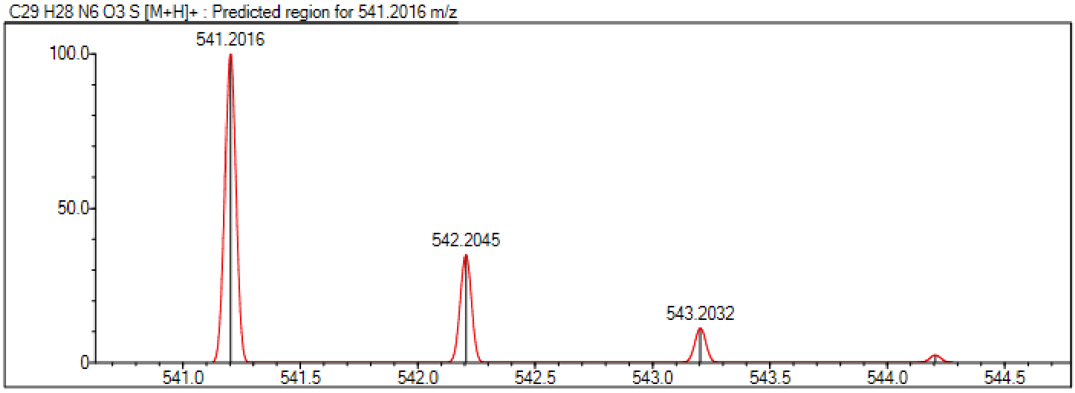


| Rank | Score | Formula (M) | Ion | Meas. m/z | Pred. m/z | Df. (mDa) | Df. (ppm) | Iso | DBE |
|---|---|---|---|---|---|---|---|---|---|
| 1 | 45.66 | C29 H28 N6 O3 S | [M+H]+ | 541.2002 | 541.2016 | -1.4 | -2.59 | 47.55 | 19.0 |

**Figure 76.** Mass spectrum of compound **5m**

DOPNALAB

| Item | Value |
|---|---|
| Acquired Date&Time | 5.02.2019 11:39:48 |
| Acquired by | System Administrator |
| Filename | C:\Users\dopnalab\Desktop\derya\OP\OP-281.ispd |
| Spectrum name | OP-281 |
| Sample name | OP-28 |
| Sample ID | |
| Option | |
| Comment | |
| No. of Scans | 10 |
| Resolution | 4 [cm-1] |
| Apodization | Happ-Genzel |

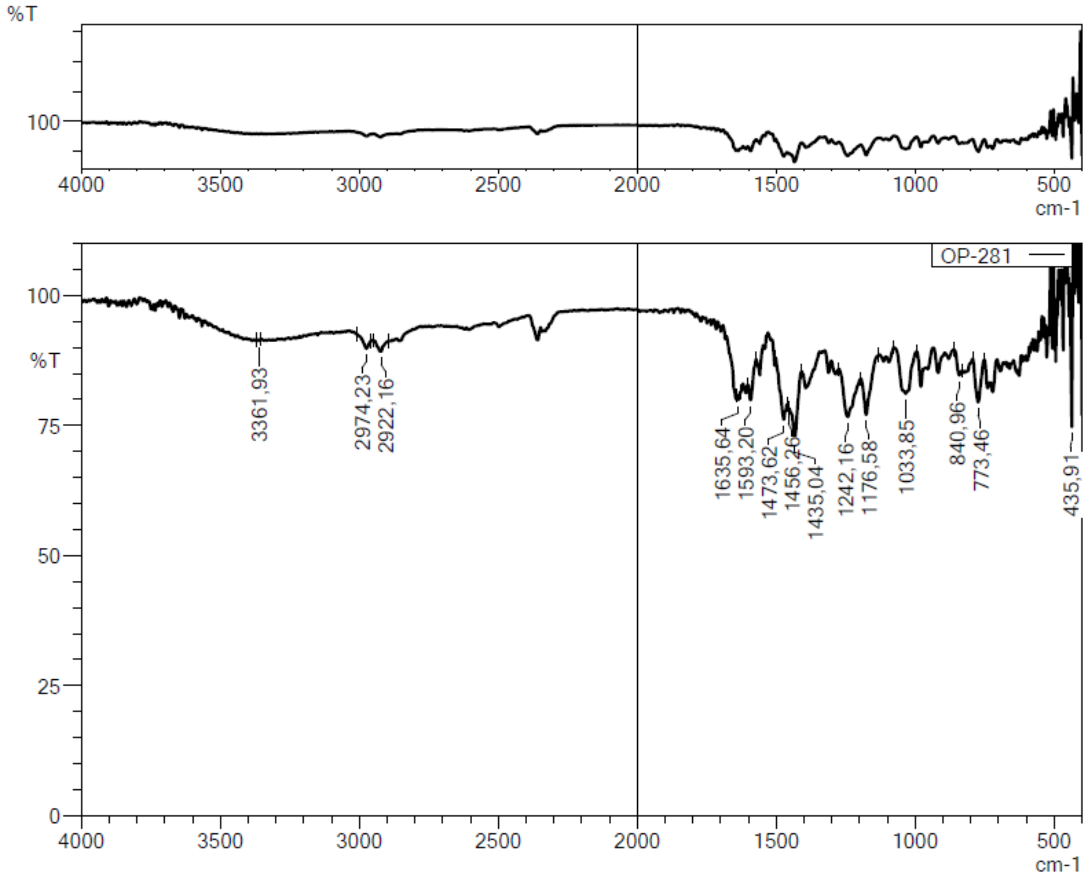


**Figure 77.** IR spectrum of compound **5n**

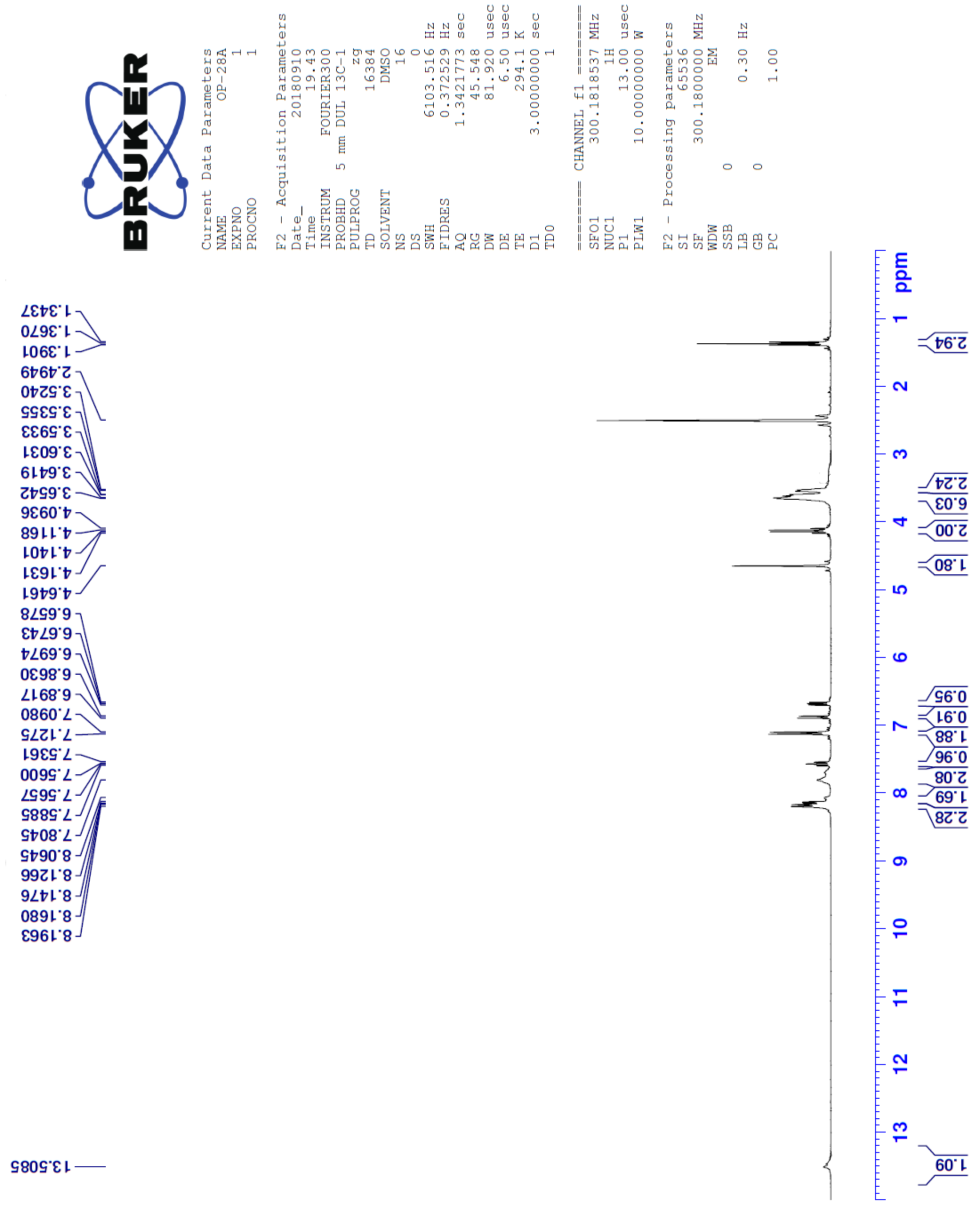


**Figure 78.** $^{1}$H-NMR spectra of compound **5n**

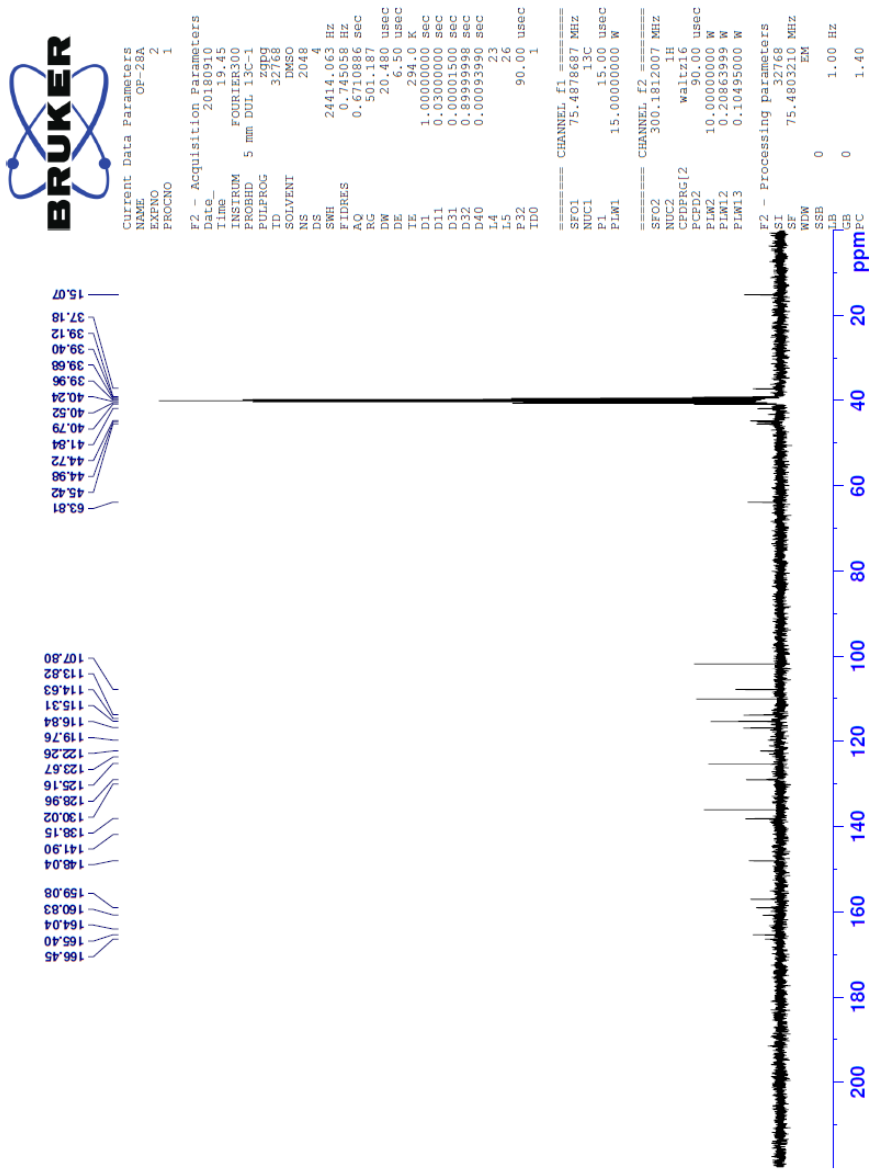


**Figure 79.** $^{13}$C-NMR spectra of compound **5n**

Formula Predictor Report - OP-28_11.lcd Page 1 of 1

Data File: C:\LabSolutions\Data\Analiz\uac\OP-28_11.lcd

| Elmt | Val. | Min | Max | Elmt | Val. | Min | Max | Elmt | Val. | Min | Max | Elmt | Val. | Min | Max | Use Adduct |
|---|---|---|---|---|---|---|---|---|---|---|---|---|---|---|---|---|
| H | 1 | 20 | 40 | O | 2 | 1 | 5 | S | 2 | 1 | 1 | Ru | 2 | 0 | 0 | H |
| C | 4 | 15 | 30 | F | 1 | 0 | 1 | Cl | 1 | 0 | 1 | Pd | 2 | 0 | 0 | |
| N | 3 | 6 | 8 | P | 3 | 0 | 0 | Br | 1 | 0 | 1 | I | 3 | 0 | 0 | |

Error Margin (ppm): 5
HC Ratio: unlimited
Max Isotopes: 3
MSn Iso RI (%): 10.00

DBE Range: 16.0 - 20.0
Apply N Rule: yes
Isotope RI (%): 1.00
MSn Logic Mode: AND

Electron Ions: both
Use MSn Info: yes
Isotope Res: 9000
Max Results: 500

Event#: 1 MS(E+) Ret. Time : 3.880 -> 3.880 Scan# : 583 -> 583

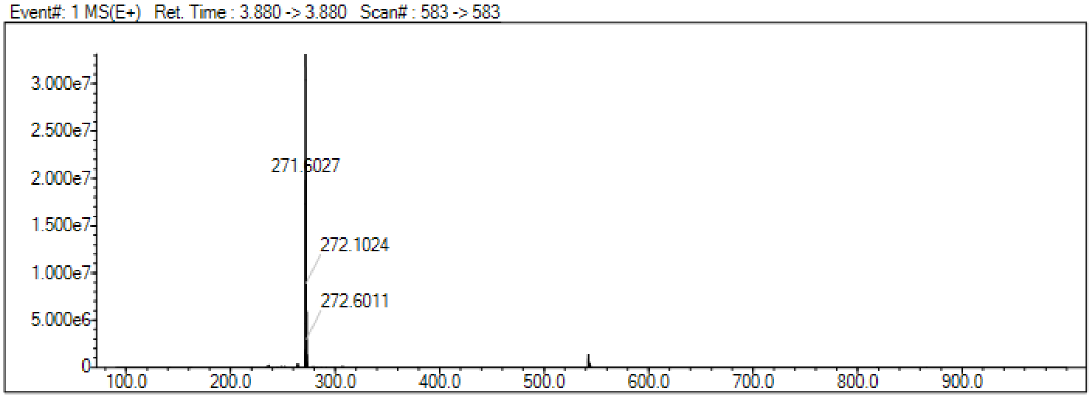


Measured region for 542.1982 m/z

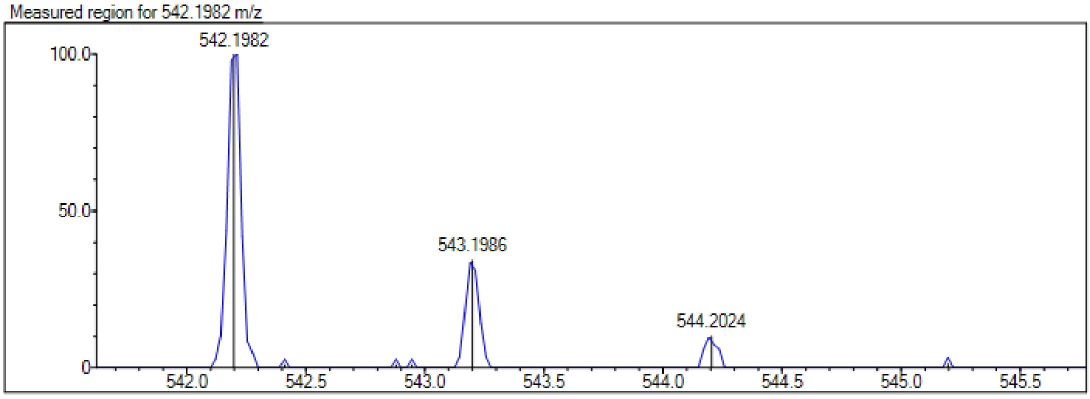


C28 H27 N7 O3 S [M+H]+ : Predicted region for 542.1969 m/z

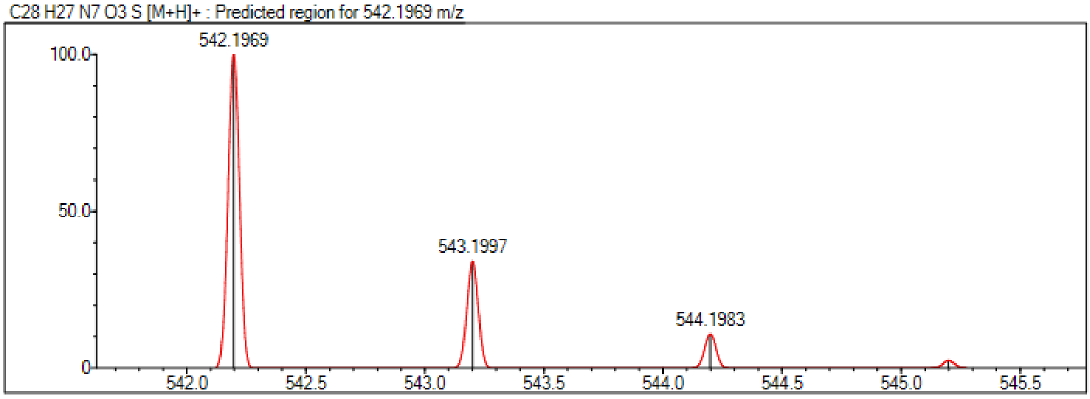


| Rank | Score | Formula (M) | Ion | Meas. m/z | Pred. m/z | Df. (mDa) | Df. (ppm) | Iso | DBE |
|---|---|---|---|---|---|---|---|---|---|
| 1 | 95.38 | C28 H27 N7 O3 S | [M+H]+ | 542.1982 | 542.1969 | 1.3 | 2.40 | 98.84 | 19.0 |

**Figure 80.** Mass spectrum of compound **5n**

DOPNALAB

| | Item | Value |
|---|---|---|
| | Acquired Date&Time | 5.02.2019 12:11:29 |
| | Acquired by | System Administrator |
| | Filename | C:\Users\dopnalab\Desktop\derya\OP\op-291.ispd |
| | Spectrum name | op-291 |
| | Sample name | OP-29 |
| | Sample ID | |
| | Option | |
| | Comment | |
| | No. of Scans | 10 |
| | Resolution | 4 [cm-1] |
| | Apodization | Happ-Genzel |

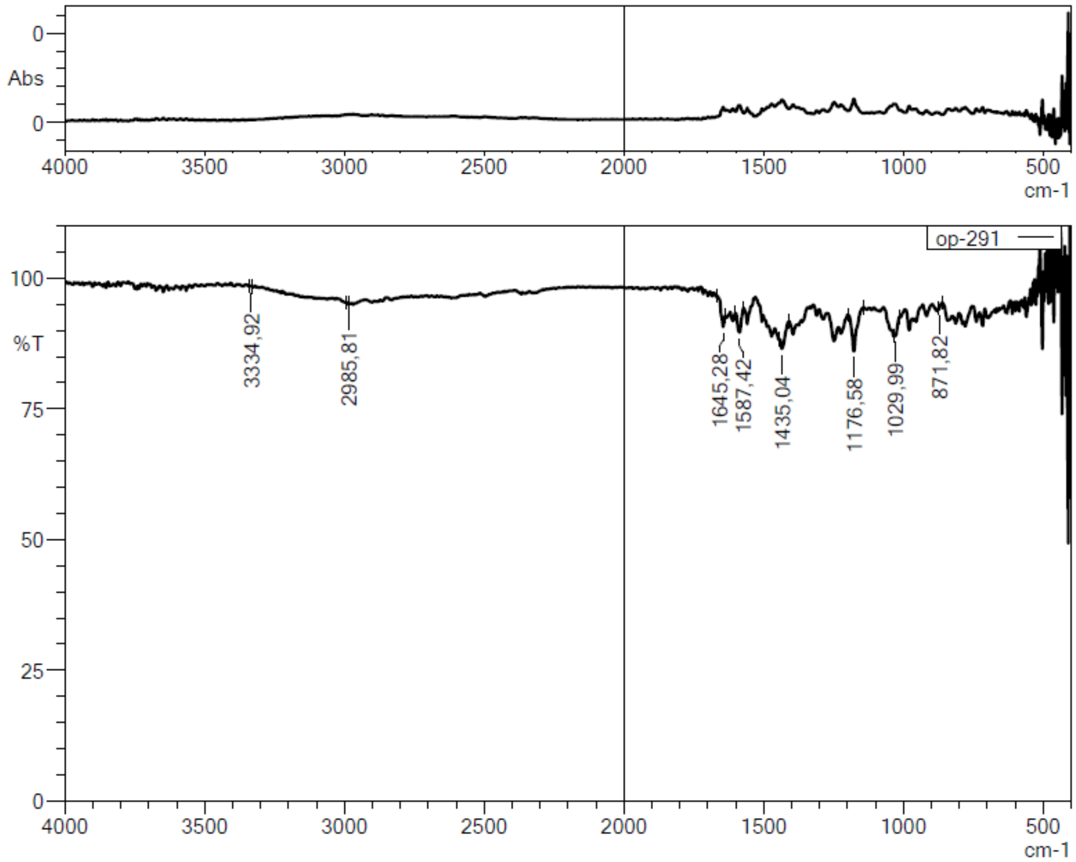


**Figure 81.** IR spectrum of compound **5o**

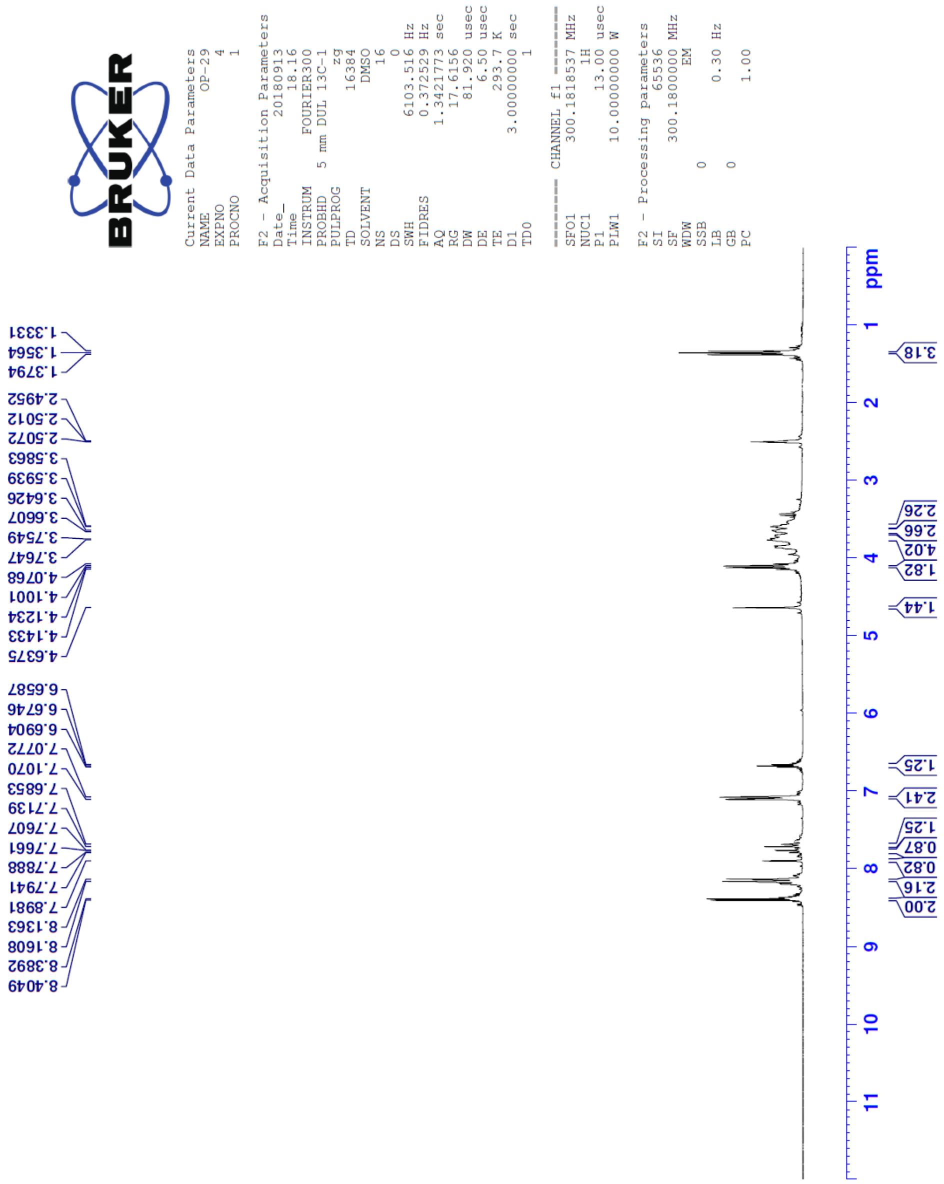


**Figure 82.** $^{1}$H-NMR spectra of compound **5o**

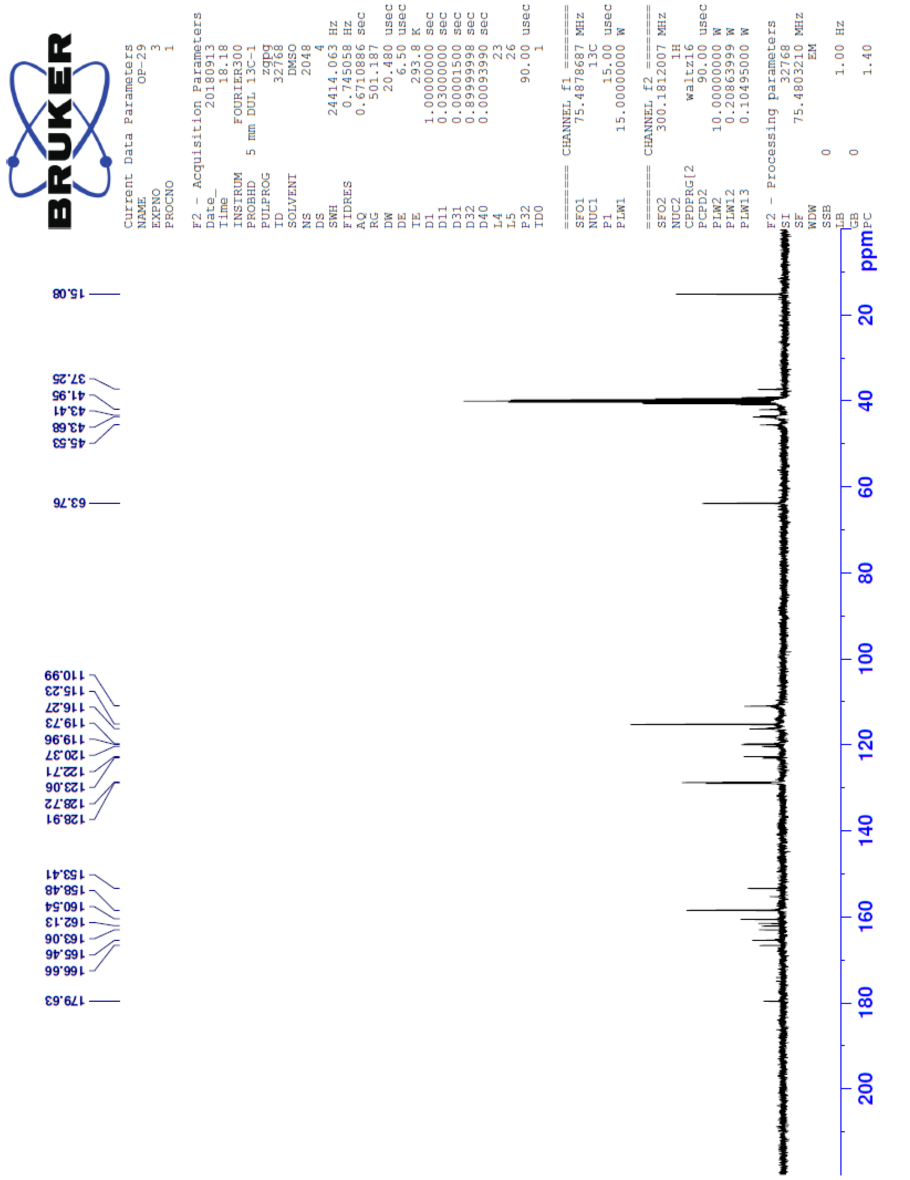


**Figure 83.** $^{13}$C-NMR spectra of compound **5o**

Formula Predictor Report - OP-29_13.lcd



Data File: C:\LabSolutions\Data\Analiz\uac\OP-29_13.lcd

| Elmt | Val. | Min | Max | Elmt | Val. | Min | Max | Elmt | Val. | Min | Max | Elmt | Val. | Min | Max | Use Adduct |
|---|---|---|---|---|---|---|---|---|---|---|---|---|---|---|---|---|
| H | 1 | 20 | 40 | O | 2 | 1 | 5 | S | 2 | 1 | 1 | Ru | 2 | 0 | 0 | H |
| C | 4 | 15 | 30 | F | 1 | 0 | 1 | Cl | 1 | 0 | 1 | Pd | 2 | 0 | 0 | |
| N | 3 | 6 | 8 | P | 3 | 0 | 0 | Br | 1 | 0 | 1 | I | 3 | 0 | 0 | |

Error Margin (ppm): 5
HC Ratio: unlimited
Max Isotopes: 3
MSn Iso RI (%): 10.00

DBE Range: 16.0 - 20.0
Apply N Rule: yes
Isotope RI (%): 1.00
MSn Logic Mode: AND

Electron Ions: both
Use MSn Info: yes
Isotope Res: 9000
Max Results: 500

Event#: 1 MS(E+) Ret. Time : 5.253 Scan# : 789

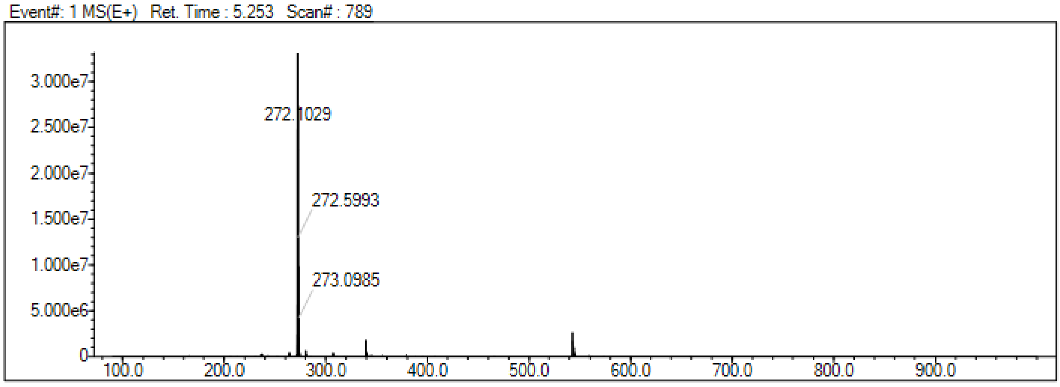


Measured region for 543.1907 m/z

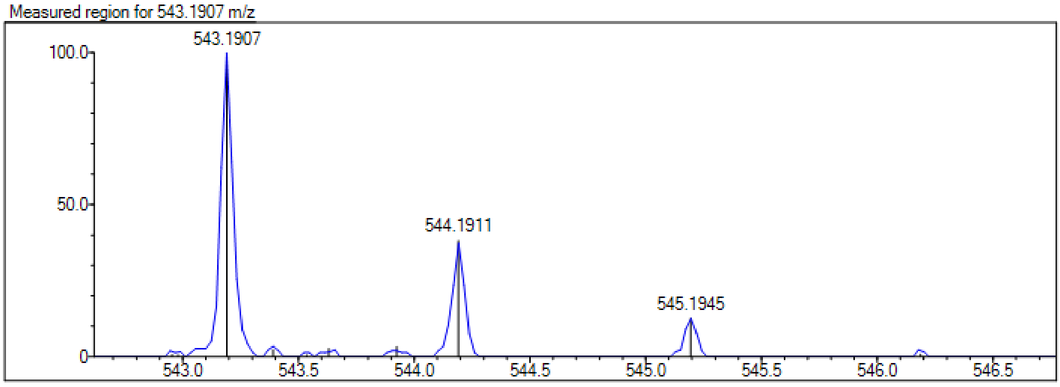


C27 H26 N8 O3 S [M+H]+ : Predicted region for 543.1921 m/z

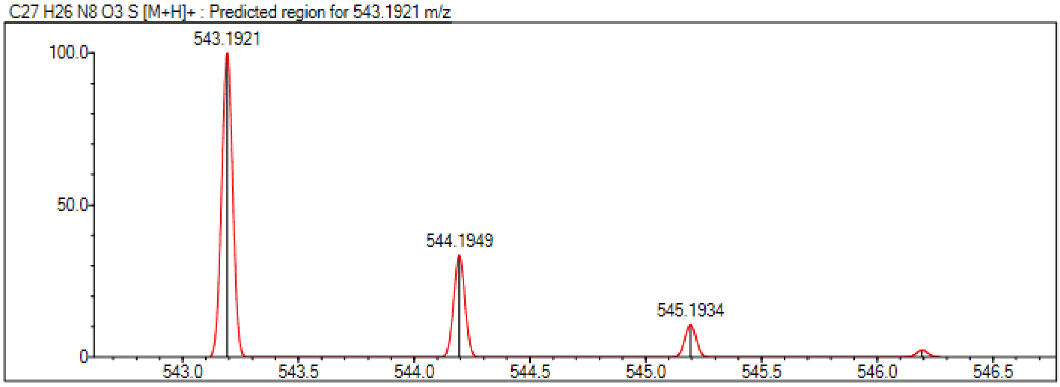


| Rank | Score | Formula (M) | Ion | Meas. m/z | Pred. m/z | Df. (mDa) | Df. (ppm) | Iso | DBE |
|---|---|---|---|---|---|---|---|---|---|
| 1 | 79.47 | C27 H26 N8 O3 S | [M+H]+ | 543.1907 | 543.1921 | -1.4 | -2.58 | 82.74 | 19.0 |

**Figure 84.** Mass spectrum of compound **5o**

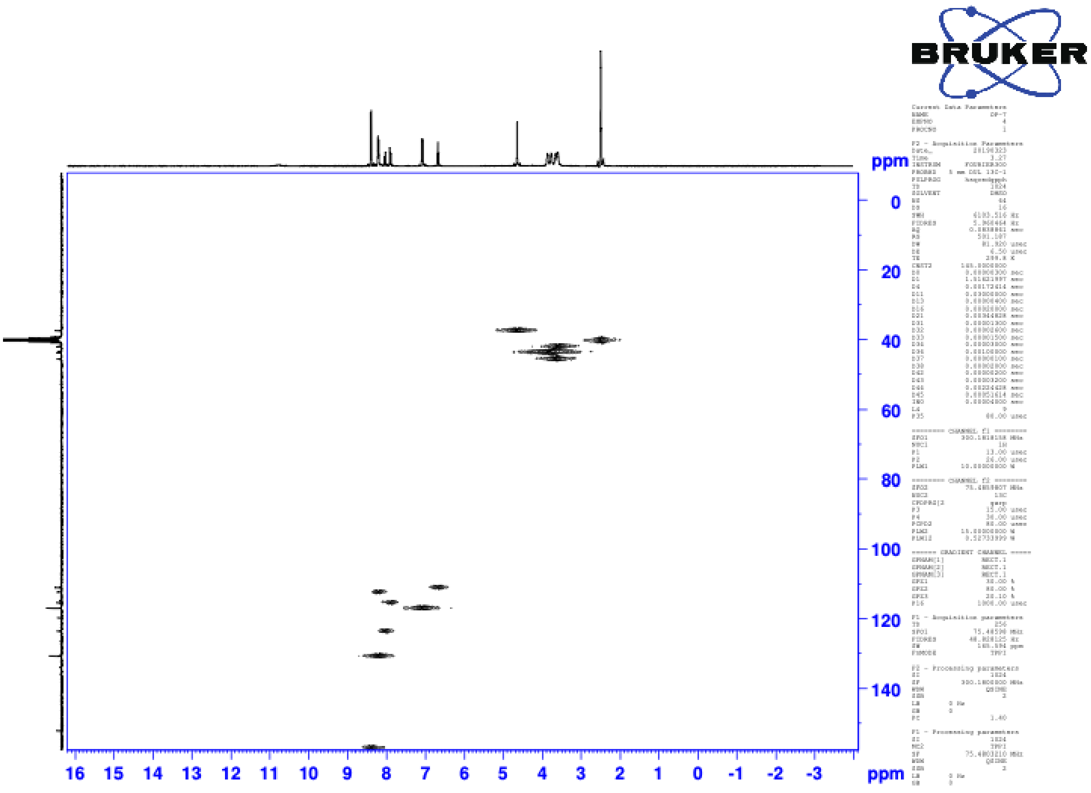


**Figure 85.** 2D HSQC spectrum of the compound **5e**

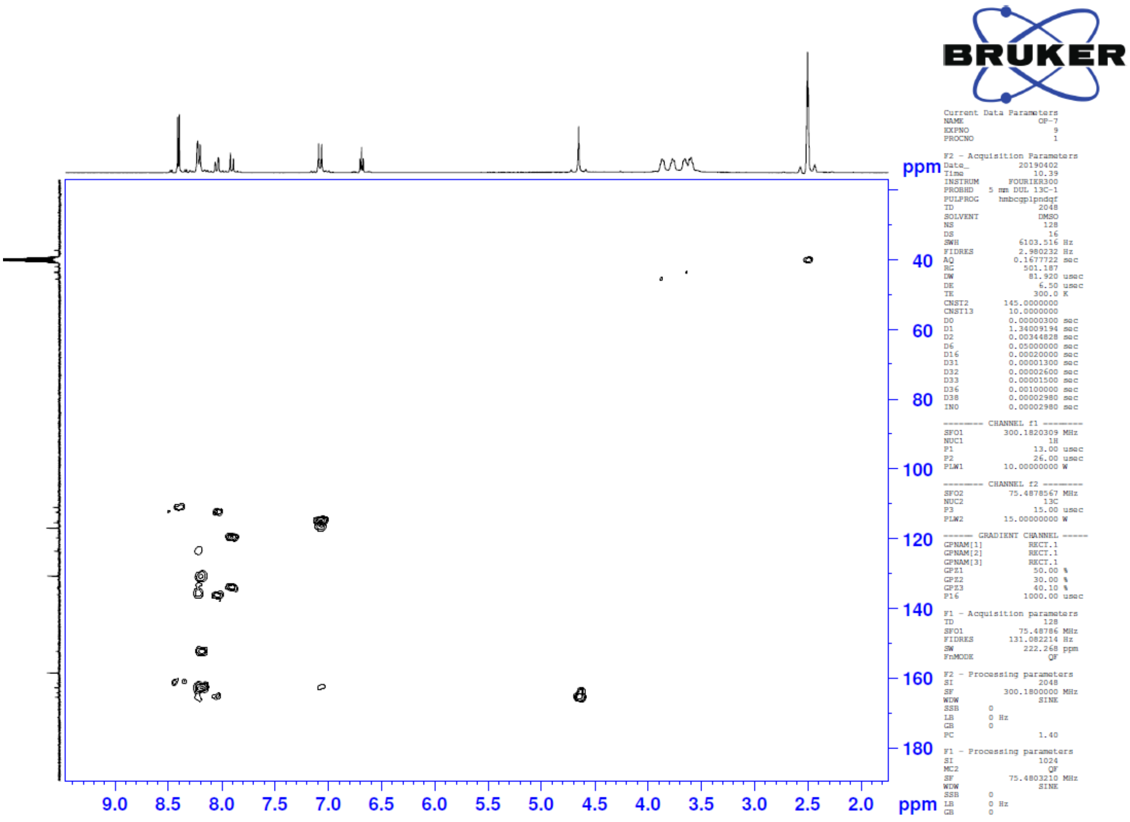


**Figure 86.** 2D HMBC spectrum of the compound **5e**

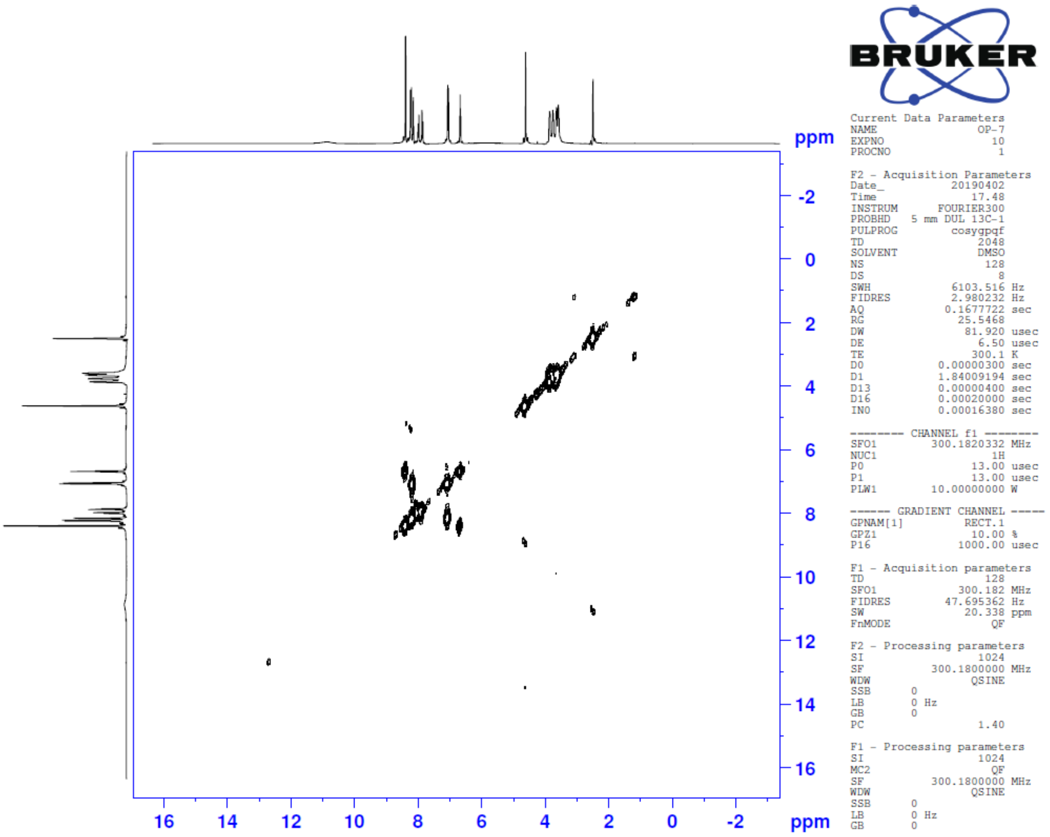


**Figure 87.** 2D COSY spectrum of the compound **5e**
